# Dual Fluorescence and Phosphorescence Emissions from Dye-Modified (*NCN*)-Bismuth Pincer Thiolate Complexes

**DOI:** 10.1021/acs.inorgchem.4c01023

**Published:** 2024-07-30

**Authors:** Marcel Geppert, Kai Jellinek, Michael Linseis, Michael Bodensteiner, Jessica Geppert, Miriam M. Unterlass, Rainer F. Winter

**Affiliations:** Fachbereich Chemie, Universität Konstanz, 78457 Konstanz, Germany

## Abstract

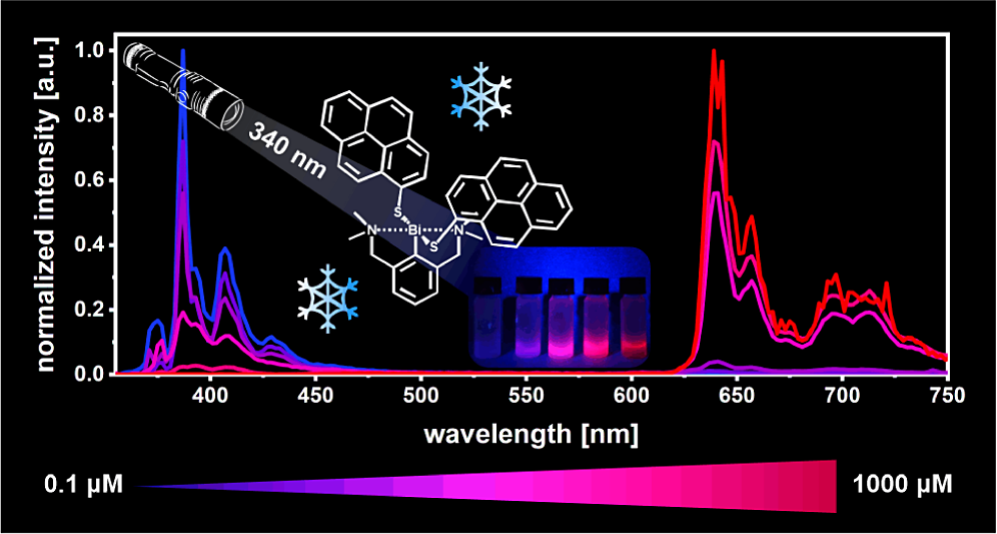

We report the synthesis, characterization, and photophysical properties of four new dye-modified (*NCN*)Bi pincer complexes with two mercaptocoumarin or mercaptopyrene ligands. Their photophysical properties were probed by UV/vis spectroscopy, photoluminescence (PL) studies, and time-dependent density functional theory (TD-DFT) calculations. Absorption spectra of the complexes are dominated by mixed pyrene or coumarin π → π*/n(pS) → pyrene or coumarin π* transitions. While unstable toward reductive elimination of the corresponding disulfide under irradiation at room temperature, the complexes provide stable emissions at 77 K. Under these conditions, coumarin complexes **2** and **4** exhibit exclusively green phosphorescence at 508 nm. In contrast, the emissive properties of pyrene complexes **1** and **3** depend on the excitation wavelength and on sample concentration. Irradiation into the lowest-energy absorption band exclusively triggers red phosphorescence from the pyrenyl residues at 640 nm. At concentrations *c* < 1 μM, excitation into higher excited electronic states results in blue pyrene fluorescence. With increasing *c* (1–100 μM), the emission profile changes to dual fluorescence and phosphorescence emission, with a steady increase of the phosphorescence intensity, until at *c* ≥ 1 mM only red phosphorescence ensues. Progressive red-shifts and broadening of steady-state excitation spectra with increasing sample concentration suggest the presence of static excimers, as we observe it for concentrated solutions of pyrene. Crystalline and powdered samples of **1** indeed show intermolecular association through π-stacking. TD-DFT calculations on model dimers and a tetramer of **1** support the idea of aggregation-induced intersystem crossing (AI-ISC) as the underlying reason for this behavior.

## Abstract 2

Four new complexes (NCN)Bi(SDye) with coumarin- or pyrene-mercapto ligands were synthesized and investigated for their emissive properties. Mercaptocoumarin complexes show efficient ISC and green phosphorescence at 509 nm at 77 K in MeTHF glass. The emission from mercaptopyrene complexes depends on concentration *c* and excitation wavelength. With increasing *c*, the emission from higher-excited electronic states changes from blue fluorescence to red phosphorescence due to aggregation-induced intersystem crossing.

## Introduction

Luminescent compounds, especially those showing phosphorescence, are of great interest due to their various applications in organic light emitting diodes (OLEDs), photodynamic therapy (PDT), bioimaging, and dye-sensitized solar cells (DSSCs).^1−16^ Phosphorescence usually emanates from an excited triplet state, which is populated through a quantum mechanically forbidden spin flip known as intersystem crossing (ISC). Possibilities to accelerate ISC are to capitalize on the so-called heavy atom effect (HAE),^17^ or on electronic transitions between different types of molecular orbitals, e.g., ^1^ππ* → ^3^nπ* or ^1^nπ* → ^3^ππ* excitations, according to the rule of El Sayed.^18^ Metal-to-ligand charge transfer (MLCT) or ligand-to-metal charge transfer (LMCT) transitions, as well as small energy differences between an excited singlet state S_*n*_ and the corresponding triplet state T_*n*_ are also known to promote ISC.^18−20^ Taking advantage of the HAE, myriads of transition metal complexes with metal ions having large spin–orbit coupling (SOC) constants like platinum, gold, iridium or rhenium were prepared and studied for their emissive properties.^20−26^ All of these noble metals however come with the drawbacks of low natural abundance and high cost, rendering these approaches poorly sustainable.

**Scheme 1:**
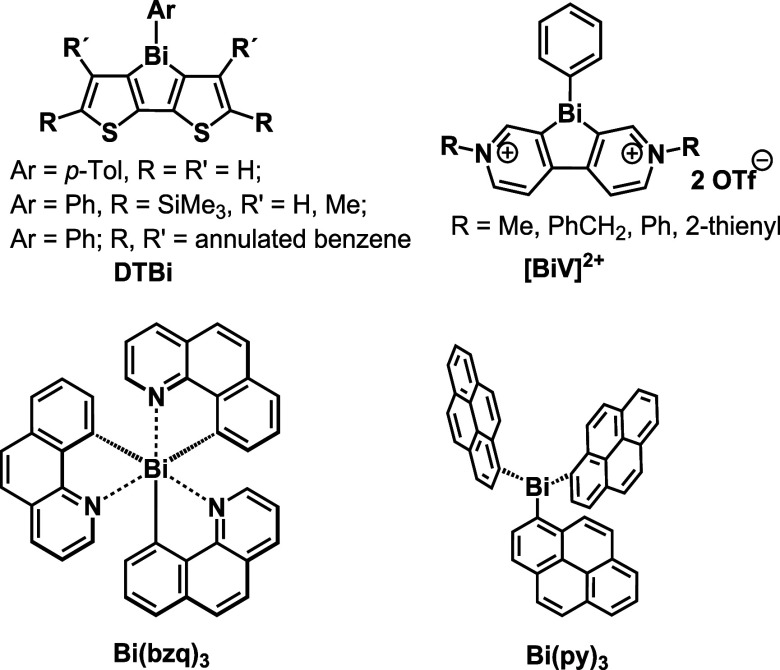
Compilation of Luminescent Bismuth Complexes

The element bismuth offers a viable alternative, as it combines the advantages of good availability, affordability, inherently low toxicity, as well as having the largest spin–orbit coupling constant of all nonradioactive elements.^27−32^ Despite several reports on luminescent bismuth complexes, the majority only show ligand-based fluorescence with attenuated intensity compared to the free ligands,^33−44^ whereas others exhibit phosphorescence in the solid state,^33,38,39,45−51^ or in frozen solvent matrix at *T* = 77 K.^33,39,43,52−55^ Phosphorescence emission of bismuth complexes in solution at room temperature is an even rarer phenomenon with only few reports in the literature.^46,51,56,57^ The examples with highest relevance to the present study are compiled in Scheme 1. In 2010, Ohshita et al. reported on four dually emissive dithienobismole complexes DTBi, which show blue fluorescence as well as red phosphorescence at room temperature in CHCl_3_ solution, albeit with only poor quantum yields ϕ_ph_ of ca. 0.2%.^46^ More recently, Ma et al. have reported on bismoviologenes (BiV^2+^), which phosphoresce in fluid CH_3_CN solution with ϕ_ph_ of up to 4.5% and in the solid state.^51^ Almost coincidently, Maurer et al. presented the bismuth benzo[*h*]quinoline complex Bi(bzq)_3_, which, in degassed solution at room temperature, exhibits cyan phosphorescence with a remarkable ϕ_ph_ of 10%. The authors have attributed efficient ISC to MLCT from the Bi 6s orbital to π* orbitals of the benzo[h]quinoline ligands. Comparison with nonphosphorescent [Bi(bzq)_2_]^+^ Br^–^, which lacks MLCT transitions, indicates that the HAE of the bismuth ion alone does not suffice to trigger efficient ISC in bzq complexes of Bi^3+^. The small absorption coefficient for the underlying HOMO–LUMO transition of only 100 M^–1^cm^–1^ as well as rapid degradation of Bi(bzq)_3_ to [Bi(bzq)_2_]^+^ upon irradiation however impose serious restrictions for its practical utility.^56^

In earlier work, Behm et al. reported on the tris(pyrenyl) pnictogens Pn(py)_3_ of the elements Pn = P, As, Sb, and Bi.^40^ All of these complexes display pyrene fluorescence at ca. 330 nm from individual molecules. A second, much broader emission was observed at 400–600 nm, whose intensity increases from P to Bi (ϕ_Bi_ = 0.44%). Based on steady-state excitation spectra, the authors assigned the latter emission to static excited-state dimers or oligomers (excimers) that result from association of individual molecules *via* their pyrenyl residues.^40^

In our present work, we have investigated Bi complexes of the 2,6-bis(dimethylaminomethyl)phenyl *NCN* pincer ligand with and without attached dye ligands and studied their photophysical properties. This we did with the hope that the HAE of the Bi^3+^ cation, augmented with intraligand or *NCN*-to-dye charge-transfer contributions to the relevant excitations, might foster ISC and trigger dye-based phosphorescence emission, as we have observed it for Pt complexes with σ-bonded dye ligands.^58−61^ Complexes **(*****NCN*****)BiX**_**2**_ (X = Cl, Br, I) are quite stable against air and water^62^ and are easily reduced to Bi(I) complexes **(*****NCN*****)Bi**. The latter readily undergo oxidative addition with disulfides to afford bis(thiolato) Bi(III) species **(*****NCN*****)Bi(SR)**_**2**_.^63,64^ This provides a straightforward access route to Bi complexes with dye ligands, even ones with functionalities that preclude their conversion into organyllithium or Grignard reagents and their use as transmetalating agents toward **(*****NCN*****)BiCl**_**2**_.^65^ We here apply this strategy to introduce pyrene and coumarin dyes into the coordination sphere of Bi^3+^.

## Results and Discussion

### Synthesis and Characterization

**Scheme 2:**
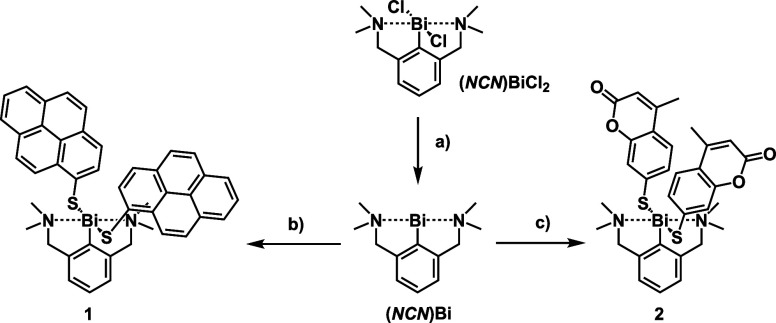
Synthesis of Complexes **1** and **2**: (a) *K*-Selectride, THF, 1 h, −78 °C; (b) **PyreneS**_**2**_, −78 °C to Room Temperature, 2 h; (c) **CoumarinS**_**2**_, −78 °C to Room Temperature, 2 h

**Scheme 3:**
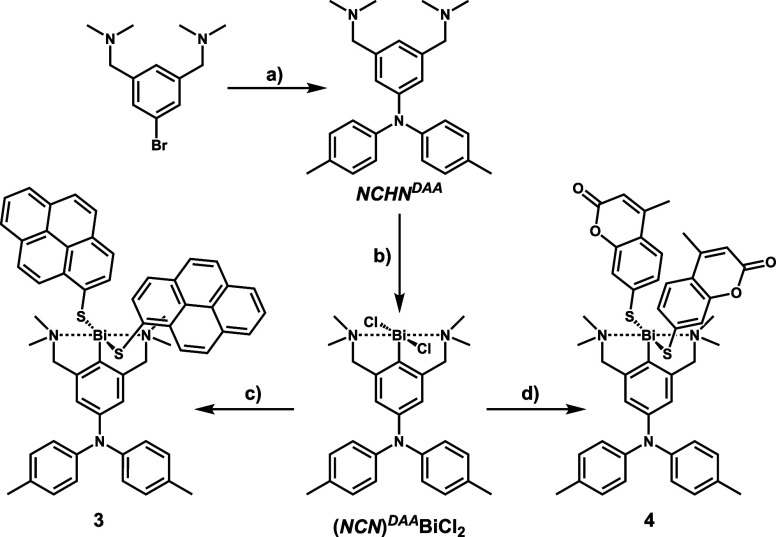
Synthesis of Complexes **3** and **4**: (a) Di(*p*-tolyl)amine, P^*t*^Bu_3_, NaO^*t*^Bu, [Pd_2_(dba)_3_], Toluene, 3 d, Reflux; (b) (i) ^*n*^BuLi, *n*-Hexane, 24 h, Reflux; (ii) BiCl_3_, Et_2_O, 1 h, −78 °C, Then 24 h Room Temperature; (c) and d) (i) *K*-Selectride, THF, 1 h, −78 °C; (ii) **CoumarinS**_**2**_ or **PyreneS**_**2**_, −78 °C to Room Temperature, 2 h

Complexes **1** and **2** were synthesized in 60% and 72% yield, respectively, by oxidative addition of pyrene or coumarin disulfide (**PyreneS**_**2**_ or **CoumarinS**_**2**_) to **(*****NCN*****)Bi**, which was generated *in situ* by reducing **(*****NCN*****)BiCl**_**2**_ with *K-*Selectride at −78 °C (Scheme 2).^62^ Complexes **3** and **4** were obtained in an identical manner starting from the new complex **(*****NCN*****)**^***DAA***^**BiCl**_**2**_ with a diarylamine- (DAA)-appended pincer ligand. Problematic purification resulted in considerably lower yields of 12% or 17% (Scheme 3). The new ligand ***NCHN*^*DAA*^** was devised with the aim of endowing complexes **3** and **4** with additional DAA-to-dye ligand-to-ligand′ charge-transfer (LL′CT) excitations that are absent in **1** and **2**. The DAA-appended ligand precursor was assembled by Buchwald–Hartwig coupling (90%) and then converted to **(*NCN*)**^***DAA***^**BiCl**_**2**_ (83%, Scheme 3).

NMR spectra of **(*NCN*)**^***DAA***^**BiCl**_**2**_ and of complexes **1**–**4** can be found in the Supporting Information (Figures S7–S18). The chemical shifts of the *N*-methyl and methylene protons in the ^1^H NMR spectrum of **(*NCN*)**^***DAA***^**BiCl**_**2**_ of 2.87 and 4.28 ppm fall close to those of 2.91 and 4.46 ppm in **(*NCN*)BiCl**_**2**_,^62^ but are shifted to significantly lower field when compared to the free ligand ***NCHN*^*DAA*^**, where they resonate at 2.20 and 3.31 ppm. The positions of all proton resonances, including those at the *p*-tolyl substituents in complexes **3** and **4**, are nearly invariant toward exchange of the chlorido for the mercapto ligands.

**Figure 1:**
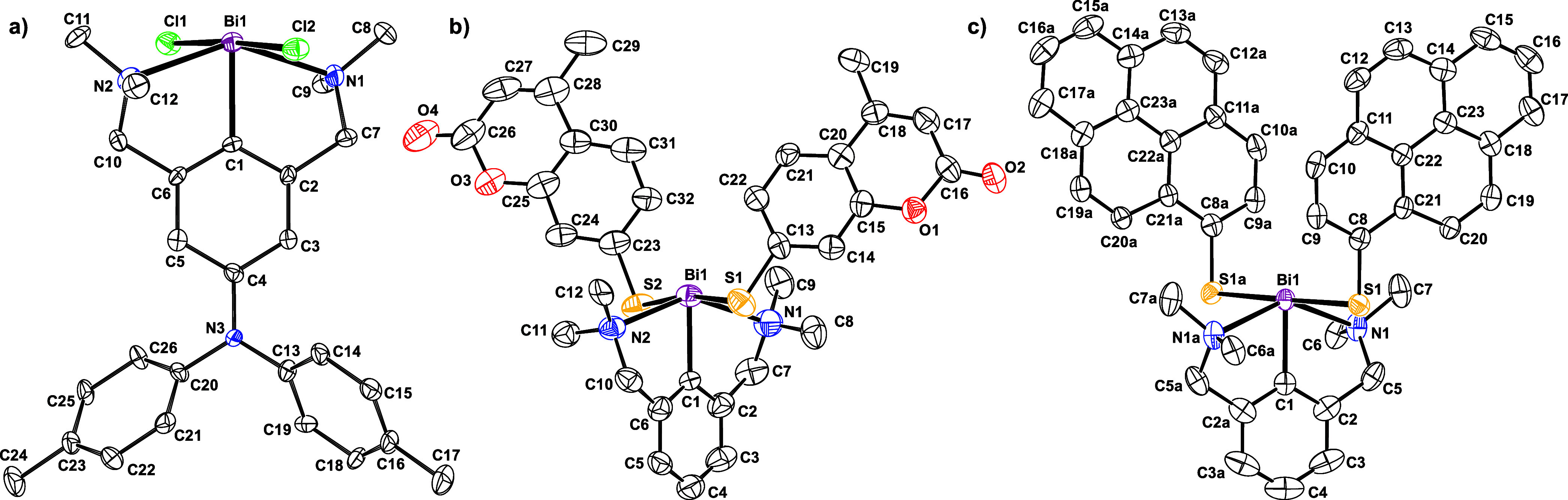
(a) Molecular structure of **(*NCN*)**^***DAA***^**BiCl**_**2**_; (b) molecular structure of complex **2**; (c) molecular structure of complex **1**. Thermal ellipsoids are drawn at the 50% probability level.

We were able to verify the structures of complexes **(*NCN*)**^***DAA***^**BiCl**_**2**_, **1** and **2** by single-crystal X-ray diffraction analysis. Figure 1 provides ORTEPs along with the atom numbering. Details to the diffraction experiments, crystal and refinement data and listings of the bond lengths, interatomic bond angles and torsion angles are provided in the Supporting Information (Tables S1–S12). Single crystals of **(*NCN***)^***DAA***^**BiCl**_**2**_ were obtained by slow diffusion of *n*-hexane into a saturated solution of the complex in CH_2_Cl_2_. The complex crystallized in the triclinic space group . Overall, the structure and metrical parameters resemble those of **(*****NCN*****)BiCl**_**2**_ closely.^62^

In the crystal lattice, molecules of **(*NCN*)**^***DAA***^**BiCl**_**2**_ pack in rows of antiparallel aligned dimers that run along the *a*-axis of the unit cell. The dimers are held together by five pairs of C–H···π interactions of 2.622(9) Å to 2.863(9) Å. Adjacent dimers associate by a pair of C–H···π interactions of 2.593(9) to 2.861(8) Å as well as C–H···Cl interactions of 2.678(6) Å to a chlorido ligand. Neighboring rows align along the *b-*axis of the unit cell and form pairwise Bi···Cl contacts of 3.500(16) Å. The molecular packing as well as relevant interatomic contacts are displayed in Figures S26 and S27.

Single crystals of **2** were obtained by slow diffusion of *n*-pentane into a saturated solution of the complex in benzene. Complex **2** crystallized in the monoclinic space group *P*2_1_ with two pairs of crystallographically unique molecules per unit cell. The structural features, including the coordination geometry and the structural distortions arising from the stereochemically active Bi lone pair and the small methylene straps, are very similar to those in the chlorido precursor **(*****NCN*****)BiCl**_**2**_ and the mercaptophenyl counterpart **(*****NCN*****)Bi(SPh)**_**2**_ (Table S13).^63^

Conspicuous differences in the structures of complex **2** and **(*****NCN*****)Bi(SPh)**_**2**_ are the orientation of the aryl substituents and the folding of the five-membered BiC_3_N chelates. The alternate positioning of atoms C(7) and C(10) above or below the N(1)–Bi-N(2) vector and the rotation of the mercaptocoumarin ligands render molecules **2** planar chiral. The investigated crystal specimen consists exclusively of one enantiomer, whereas **(*****NCN*****)Bi(SPh)**_**2**_, **(*****NCN*****)BiCl**_**2**_(62) and **(*****NCN*****)**^**DAA**^**BiCl**_**2**_ each crystallized as racemates. The molecules shown in Figure 1 correspond to the *S*_*N1*_,*S*_*N2*_ enantiomer for **2** and the *R*_*N1*_,*R*_*N2*_ enantiomer for **(*****NCN*****)***^**DAA**^***BiCl**_**2**_ and **1**, resepctively.

As is shown in Figure S28, the close to parallel alignment of the mercaptocoumarin ligands leads to an intriguing packing motif. In the crystal, molecules of complex **2** arrange in double layers. Each double layer is formed by equally oriented molecules, whereas they adopt an exactly opposite orientation in the adjacent double layers. Within each double layer we observe π-stacking interactions of 3.55(2) to 3.75(2) Å (*d* C···C) between the lactonic parts of coumarin rings. π-Stacking is complemented by a total of 14 different CH···O interactions between the O atoms of the coumarin residues and N(CH_3_) (range 2.594(15) to 2.774(12) Å) or CH protons (range 2.406(15) to 2.435(16) Å). Adjacent double layers have their coumarin rings exactly parallel aligned and associate by C–H···S interactions of 2.879(5) and 2.910(5) Å to N(CH_3_) protons as well as by C–H···O contacts of 2.651(15) and 2.665(15) Å between S(CH_3_) protons and keto O atom O(4). The network of H-bonding interactions existing within the crystal lattice is shown in Figures S29 and S30.

An extensive network of intermolecular interactions also exists in crystalline **1**. Complex **1** crystallized as a benzene disolvate by slow diffusion of *n*-pentane into a saturated solution of the complex in benzene, and as a racemate with respect to the folding of the puckered five-membered chelate ring. When viewed from the side, molecules of complex **1** have the appearance of a skewed horseshoe, with the sides defined by the *cis*-disposed pyrenyl substituents and with the pyrenyl planes tilted at an angle of 17.30(19)°. The open side of the horseshoe points away from the bismuth ion and interchelates the cyclometalating phenyl ring of the next-neighbor molecule, the latter being disposed at an angle of 9.09(19)° with respect to the encasing pyrenyl rings. This particular arrangement produces infinite chains of interlocking molecules that run along the *b-*axis of the unit cell (see Figures S31 and S32). Individual chains are held together through π-stacking interactions of 3.365(4) Å between carbon atoms of the pyrenyl substituents and the cyclometalating phenyl ring as well as short CH···S hydrogen bonds of 2.61(5) Å between one C–H proton of each pyrenyl ring and the adjacent mercapto donor atoms of the neighbor molecule. Individual chains stack one atop the other along the *a*-axis of the unit cell with alternant orientations of the open sides of the Bi(Spyr)_2_ entities. This particular packing orients the pyrenyl rings of neighboring chains in parallel, thereby allowing for short pairwise C···C contacts of 3.274(5) and 3.367(5) Å between them. The cocrystallized benzene molecules interact with four surrounding complex molecules **1**. They act as hydrogen-bond donors toward a mercapto S atom (*d*(CH···S) = 2.78(6) Å) and the pyrenyl substituent (*d*(CH···π = 2.80(5) Å) of one molecule or a pyrenyl substituent (*d*(CH···π = 2.48(5) Å) of a second neighbor molecule, and as hydrogen-bond acceptors toward a pyrenyl proton of a third molecule of **1** (*d*(CH···π) = 2.71(5) Å) or a proton of the cyclometalating phenyl ring of yet another molecule of **1** (*d*(CH···π) = 2.63(7) Å). As shown by NMR spectroscopy and combustion analysis, one benzene solvate molecule per complex unit is retained even after drying the residue for 1 day in vacuum. Moreover, the powder X-ray diffraction pattern of this material matched with that calculated based on the X-ray structure determination on a single crystal (see Figure S33).

### Electronic Absorption Spectra and TD-DFT Calculations

**Figure 2:**
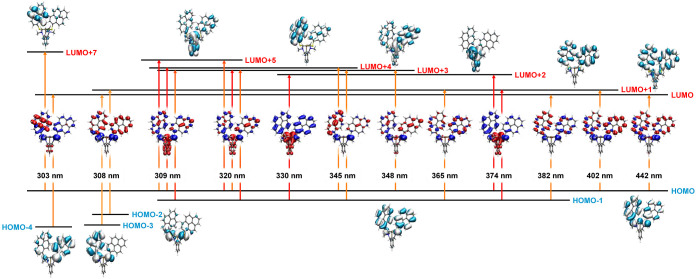
TD-DFT-calculated transitions of **1** along with the mainly contributing molecular orbitals and the electron density difference maps (EDDMs). Blue color indicates a loss and red color a gain of electron density during the corresponding excitation. LMCT/LL’CT transitions are indicated by red, and nπ*/ππ* transitions by orange arrows.

**Figure 3:**
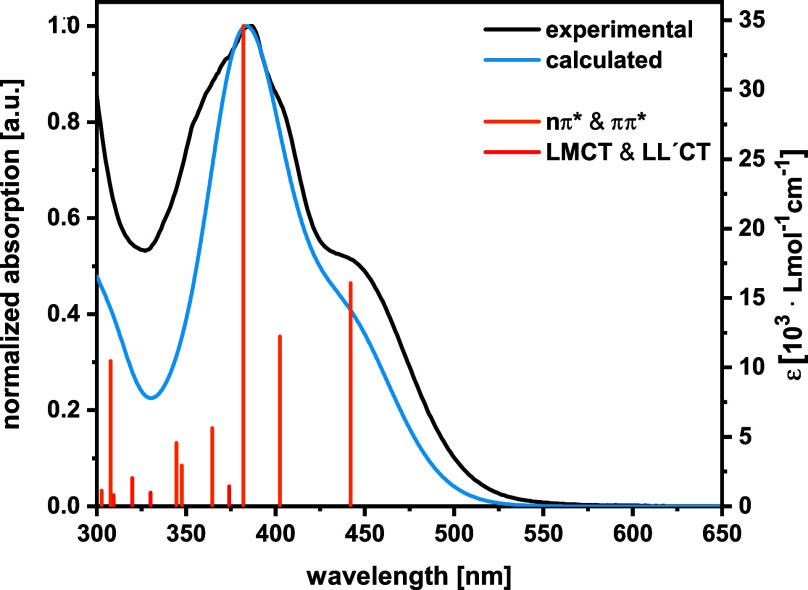
Comparison of the experimental (black line) and the TD-DFT-calculated absorption spectra of the *cisoid* conformer of complex **1** (blue line). Individual transitions are indicated by orange bars for nπ*/ππ* and red bars for CT transitions. Their heights represent the computed oscillator strengths.

In order to obtain insight into the electronic structures and the character of the electronic transitions of complexes **1**–**4**, TD-DFT calculations were carried out and amalgamated with the experimental absorption spectra. The results are shown in Figures 2 and 3 for complex **1**, and in Figures S34–S38 for complexes **2**–**4**. All complexes absorb strongly in the near UV (ε > 23,000 M^–1^ cm^–1^). A second, still fairly intense band (ε > 8,000 M^–1^ cm^–1^) is located in the visible, rendering their solutions orange (**1**, **3**) or golden yellow (**2**, **4**) in color. Both principal bands are shifted to smaller wavelengths (higher energies) in the coumarin complexes, from ca. 450 and 385 nm in **1** and **3** to ca. 415 and 345 or 368 nm in **2** and **4**. According to our quantum chemical calculations, the band envelope of the prominent UV absorption entails more than just one electronic transition (*vide infra*). An even richer structuring is noted for complexes **3** and **4** with the DAA-appended *NCN^DAA^* ligand. Relevant optical data are compiled in Table 1.

**Table 1:** Absorption and Photoluminescence Data for Complexes **1**–**4**, **CoumarinS**_**2**_, and **PyreneS**_**2**_ (**F** = Fluorescence, **P** = Phosphorescence)

compound	λ_max_/nm (ε_λ_/10^3^ M^–1^ cm^–1^)^b^	λ_Em_/nm	λ_Exc_/nm	τ_F_/ns^c^	τ_P_/μs^c^
**1**^a^	354 (27.6), 386 (34.6), 403 (29.0), 445 (17.6)	**F**: 371, 387, 407, 429;	**F**: 294, 326;	1.8 [26%], 5.3 [66%], 16 [8%]	276 [30%], 453 [70%]
		**P**: 640, 695	**P**: 291, 384, 432		
**2**^a^	344 (27.2), 359 (24.7), 414 (9.4), 350 (23.4)	**P**: 504	**P**: 346, 416	–	18 [21%], 51 [66%], 141 [13%]
**3**^a^	369 (24.9), 386 (24.4), 448 (8.2)	**F**: 377, 387, 407, 429	**F**: 361	2.4 [49%], 10 [44%], 37 [7%]	152 [27%], 485 [73%]
		**P**: 641, 699	**P**: 306, 386, 437		
**4**^a^	348 (31.0), 368 (36.3), 412 (20.3)	**P**: 509	**P**: 312, 370, 414	–	33 [43%], 79 [52%], 224 [5%]
**CoumarinS**_**2**_^a^		**F**: 402, 430, 460, 492, 606	**F**: 292, 340	1.4 [56%], 2.6 [44%]	–
**CoumarinS**_**2**_^b^		**F**: 386	**F**: 334	0.2 [58%], 1.7 [42%]	–
**PyreneS**_**2**_^a^		**F**: 376, 387, 394, 414	**F**: 337, 350, 357, 368, 376	5.7 [32%], 23 [55%], 71 [13%]	–
**PyreneS**_**2**_^b^		**F**: 387, 407, 427	**F**: 352	4.7 [82%], 14 [18%]	–

a In MeTHF at 77 K.

b In CH_2_Cl_2_ at room temperature.

c Relative contributions of individual lifetimes in %.

Our quantum chemical calculations considered two different orientations of the mercpatopyrene ligands of complexes **1** and **3**. For **1**, the *cisoid* conformer is favored slightly over the *transoid* one, whereas the opposite applies to complex **3**. Small computed energy differences between the two conformers of 1.55 and 3.85 kJ/mol suggest that both likely coexist in solution at room temperature. The mutual orientation of the pyrenyl residues does, however, not seem to have any practical implications, as the computed molecular orbitals (MOs) and electronic spectra as well as the individual excitations of the two conformers show only minor differences.

Most frontier MOs of **1** receive important contributions from the parallel disposed pyrenyl residues and are either confined to one mercaptopyrene ligand or represent in- or out-of-phase combinations of pyrene π-orbitals. HOMO and HOMO – 1 both involve antibonding interactions between pyrene π- and sulfur p-orbitals. Antibonding interactions between one lobe of a p-orbital of each thiolate donor atom and the Bi 6s orbital are noted for the HOMO and HOMO – 2. Exceptions are LUMO + 2 and LUMO + 5, which are π*-orbitals localized at the phenyl ring of the *NCN* chelate. The latter interact in a bonding or an antibonding fashion with the appropriately aligned Bi p-orbital. The same kind of MOs (and, by inference, electronic transitions) are also found for the other complexes, but are complemented by ones that are based on or receive large contributions from the appended DAA substituent in complexes **3** and **4** (see Figures S36–S38). The availability of these MOs adds additional DAA → pyrene LL′CT excitations to the manifold and accounts for the richer structuring of the electronic bands. They do however not exert any detectable influence on the emissive properties (*vide infra*).

We note an excellent agreement between the experimental and the TD-DFT computed spectra for the mercaptopyrenyl complexes **1** and **3**, as is exemplified for the *cisoid* conformer of complex **1** in Figure 3. Corresponding compilations for the *transoid* structure and both conformers of complex **3** are shown in Figures S34, S36 and S37. Larger deviations are however noted for the coumarin complexes **2** and **4**, in particular with respect to overestimated energies of the HOMO → LUMO transition. All intense electronic transitions are of mixed n(p(S)) → π*(pyrene/coumarin) and (pyrene/coumarin) π → π* character. Transitions of this kind are marked in orange color in the corresponding figures. Of considerably weaker intensity are mixed LMCT/LL′CT-type excitations from pyrenyl π-orbitals to Bi p-orbitals, in particular such with Bi–S σ* character, as well as to a π* orbital of the cyclometalating phenyl ring of the *NCN* chelate. These kind of excitations are marked in red color in the corresponding figures. DAA → pyrene/coumarin LL′CT excitations specific to complexes **3** and **4** with the *NCN*^*DAA*^ ligand are indicated by yellow color.

### Photoluminescence Studies

Complexes **1**–**4** proved to be unstable under irradiation (365 nm, 2.4 W power output) at room temperature, so that their emissive properties were explored in MeTHF at 77 K (*vide infra*). The difference in temperature also accounts for the hypsochromic shifts of the excitation spectra recorded at 77 K with respect to the absorption spectra, which pertain to room temperature conditions.

We enter the discussion of the emissive properties with coumarin complexes **2** and **4**, which, although having different *NCN* ligands, behave very similarly (see Figures S46 and S47). In glassy MeTHF at 77 K, complexes **2** and **4** show exclusively green phosphorescence at 509 nm with lifetimes in the range of 18 to 224 μs (Table 1), irrespective of the excitation wavelength and sample concentration (1 μM to 1 mM). Complete quenching of the fluorescence emission from the appended coumarin dyes demonstrates the efficacy of ISC when coordinated to the Bi^3+^ ion (Figure S48). At room temperature, solutions of both complexes in degassed CH_2_Cl_2_ are nonemissive upon excitation at λ = 420 nm and at 375 nm (Figure S49). Excitation at λ = 350 nm however gives rise to a fluorescence emission with several resolved peaks in the range of 400–500 nm and lifetimes of 0.2 and 1.7 ns (Figure S50). This emission does however not correspond with the pristine complex, but is due to **CoumarinS**_**2**_ (see Table 1 and Figure S51) as shown by comparison with an authentic sample. This indicates photoinduced decomposition by reductive elimination upon photoexcitation into higher excited states, i.e., the reversal of their synthesis from thermolabile (*NCN*)Bi^I^ and the corresponding disulfide. NMR spectra recorded on photolyzed solutions of complexes **2** and **4** confirmed the loss of the resonances of the starting complexes and the formation of **CoumarinS**_**2**_ in this process (Figure S19). The same photoinduced reactivity also prevails for mercaptopyrene complexes **1** and **3**. Solutions obtained after photolysis of **1** and **3** at λ_exc_ = 365 nm and at room temperature show the same emission and excitation spectra as well as the characteristic ^1^H NMR resonances of **PyreneS**_**2**_ (see Table 1 and Figures S52, S53, S15 and S20). All four complexes however proved stable toward irradiation at 77 K as a MeTHF glass (see Figures S48, S54 and S55).

Before entering the discussion of the emissive properties of pyrenyl complexes **1** and **3**, let us first consider the results of photophysical studies on the tris(1-pyrenyl) derivatives of the pnictides, in particular of the Bi congener Bi(py)_3_ (Scheme 1).^40^ At *c* = 11 μM in CH_2_Cl_2_ and at room temperature, compounds Pn(py)_3_ show exclusively fluorescence emission from the pyrenyl residues. It was noted that the emission and excitation spectra of the pyrenyl complexes of the heavier congeners As, Sb and Bi vary with excitation wavelength. When λ_exc_ is increased, the initially structured emission assigned to monomeric species progressively shifts red and evolves into a broader, unstructured feature. The latter gives rise to distinct bands in the excitation spectra that are likewise red-shifted from the absorptions of the monomeric species. The authors concluded that, under the conditions used in their experiments, compounds Pn(py)_3_ exist as mixtures of monomers and dimers or higher aggregates, where the latter absorb and emit at lower energies than the monomers. These so-called static excimers have to be distinguished from dynamic (or classic) excimers, which only form after photoexcitation, but are not present in the electronic ground state.^66−69^ Excimer formation is a known phenomenon for pyrene-based luminophors.^67,70,71^

In the light of the results on Pn(py)_3_, and as a further prelude to discussion of complexes **1** and **3**, we also investigated the photophysical properties of parent pyrene in the concentration range of 1 μM to 10 mM. At *c* = 1 mM, solutions in CH_2_Cl_2_ at room temperature clearly show the broad, featureless emission of pyrene excimers besides that from monomers. Excimer emission becomes the prominent feature as *c* is further increased to 10 mM (see Figures S56 and S57). Excitation spectra recorded at the wavelength of the excimer emission are considerably broader and are red-shifted with respect to those in dilute solution. This clearly evidences that static pyrene excimers exist in concentrated CH_2_Cl_2_ solution, in contrast to some reports in the literature.^66,72−76^ In a MeTHF glass at 77 K, excimer formation only leads to a decreased intensity of the sharp, most blue-shifted emission peak at 371 nm.

**Figure 4:**
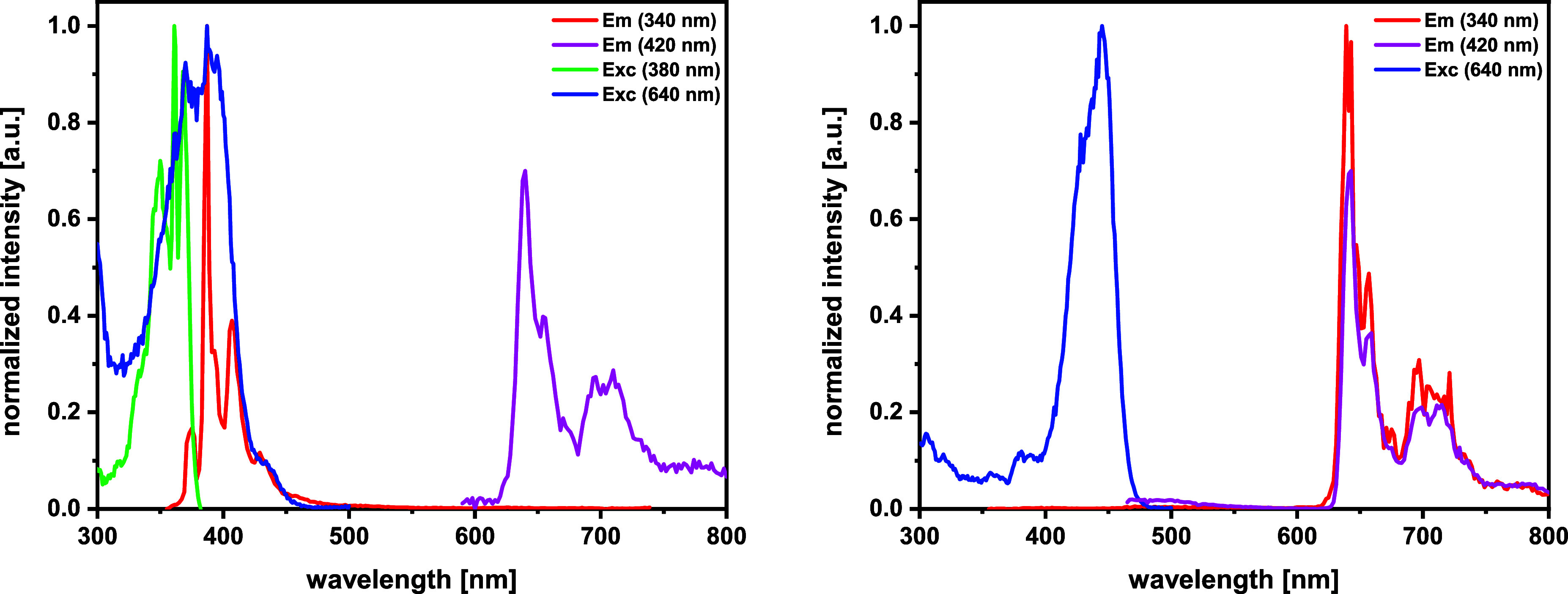
Left: emission (red and violet) and excitation spectra (green and blue) of **1** at a concentration of 0.1 μM in MeTHF at 77 K; right: emission (red and violet) and excitation spectra (blue) of **1** at a concentration of 1 mM in MeTHF at 77 K.

Set against this background, we monitored the emission profiles of complexes **1** and **3** at different sample concentrations *c*. Irrespective of *c*, excitation at 420 nm exclusively triggers red phosphorescence from the mercaptopyrene ligands with the vibrational structuring typical of pyrenyl chromophores and lifetimes of 152 to 485 μs (see Figures 4 and S58 and Table 1). Excitation into the S_1_ state of mixed π → π* and n(p(S)) → π*(pyrene) character is obviously followed by rapid ISC, promoted by the HAE of the Bi^3+^ ion, which quenches pyrene fluorescence and triggers red phosphorescence emission from the T_1_ state. Similar pyrene phosphorescence emissions were reported for complexes of Au, Pt, Hg, Re and Ir with pyrenyl ligands.^77−81^

**Figure 5:**
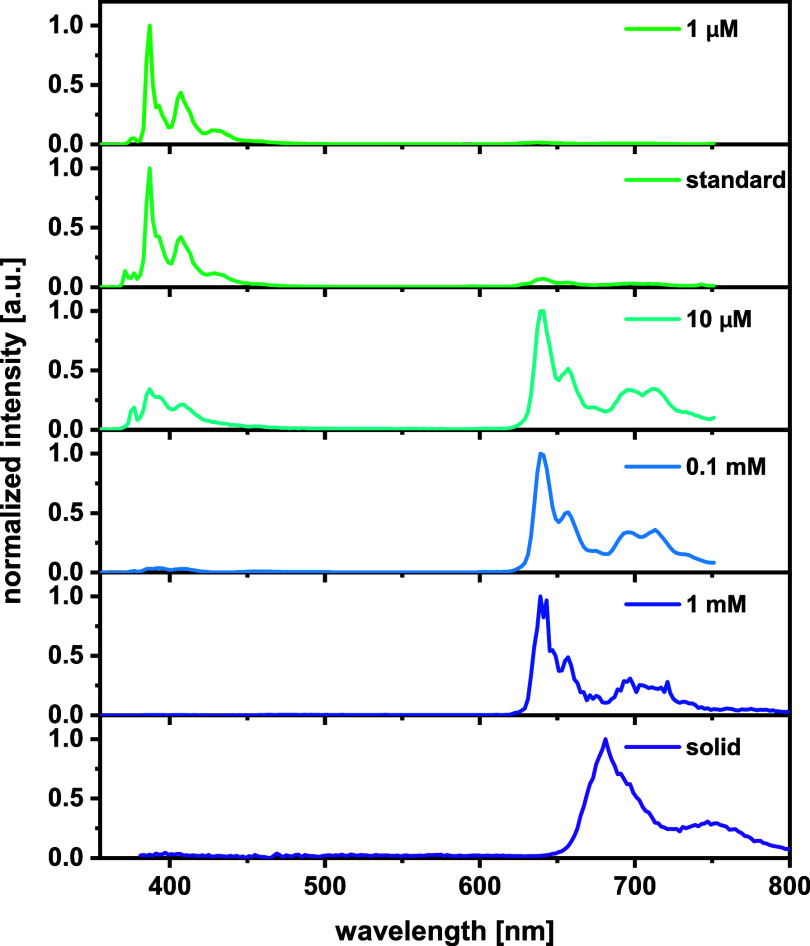
Concentration-dependent emission spectra of complex **1** excited at 340 nm in MeTHF at 77 K (standard = 3–5 μM).

The emission profile however changes upon excitation into higher excited electronic states, where a clear concentration dependence is noted. At *c* smaller than 1 μM, excitation at 340 nm yields solely blue pyrene fluorescence with a main peak at 387 nm and lifetimes in the range of 2 to 37 ns, which contrasts with the predications of Kasha’s rule.^82^ In the concentration range of 3–100 μM, the emission profile for excitation at 340 nm changes to dual fluorescence and phosphorescence. We note a steady decrease of the proportion of the fluorescence emission at increasing *c* until it completely vanishes at *c* = 1 mM (Figures 5 and S59). The *c*-dependent differences in emission spectra noted upon excitation into higher electronic states seem to relate to the presence of static excimers in higher concentrated solutions of complexes **1** and **3**. An onset of excimer formation at *c* = 1 μM is indicated by the appearance of a new peak at 432 nm in excitation spectra of complex **1** recorded at the wavelength of 640 nm, where the maximum phosphorescence intensity is found. This peak is clearly red-shifted from its position at 384 nm in 0.1 μM solutions (see Figure S60). On increasing *c* further to 100 μM or 1 mM, the excitation peak is displaced to even higher wavelengths of 450 or 463 nm. This suggests that, at higher concentrations, mercaptopyrenyl complexes **1** and **3** exist as static excimers or even higher oligomers in the MeTHF matrix at 77 K.

The latter results indicate that radiationless deactivation of higher excited singlet states S_*n*_ of monomeric complexes **1** and **3** to the first excited singlet state S_1_ and subsequent ISC to T_1_, are slow, so that fluorescence emission from a state S_*n*_ (*n* > 1) prevails. This however changes upon the association of individual molecules to dimers or higher oligomers.

The intriguing packing motif observed in crystalline **1** indicates strong π-stacking interactions between pyrenyl residues of proximal molecules as well as between pyrenyl residues and the cyclometalating phenyl ring of the *NCN* pincer ligand. Intermolecular interactions may delocalize excited states over more than just one individual molecule and give rise to a higher density and closer energetic proximity of excited singlet and triplet states as compared to the monomers. As a result, the energy gaps between states S(E)_*n*_ and T(E)_*n*_ will become smaller, which in turn increases the rate constant for ISC. This effect is known as aggregation-induced ISC (AI-ISC) and would explain why, at higher sample concentrations, the relative intensity of phosphorescence emission increases at the expense of monomer-based fluorescence.^83−85^

**Figure 6:**
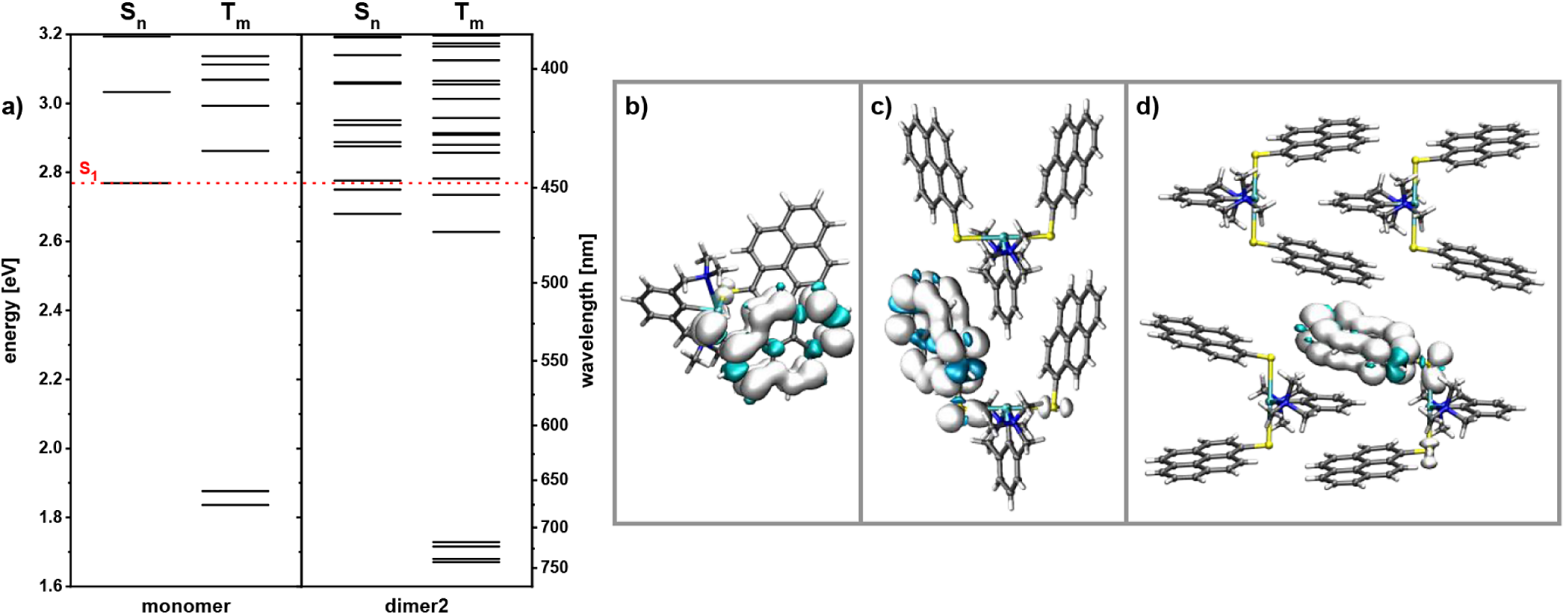
(a) Energies of the singlet and triplet states of monomeric **1** and **dimer2**. Triplet state spin density for triplet state T_1_ of (b) geometry-optimized monomeric **1**, and of (c) **dimer2** and (d) the **tetramer** with structure parameters taken from the X-ray data.

In order to probe this hypothesis, we conducted quantum chemical calculations on monomeric **1** as well as on simplified models of dimers and of a tetramer, the latter exemplifying a higher aggregate (see Figures S39–S45 and Tables S20–S35). The structural parameters and intermolecular association patterns of the models were directly taken from the experimental X-ray data and kept unchanged, irrespective of the electronic state. While, at first glance, this may seem a poor approximation, one should consider that the frozen THF matrix does likely prevent higher-amplitude structural changes. Our calculations considered the two kinds of dimers resulting from pyrene/pyrene π-stacking (**dimer1**) and pincer intercalation (**dimer2**), as well as an assembly of four molecules that show both types of interactions in a pairwise fashion (**tetramer**). We furthermore optimized the structure of the T_1_ state of monomeric **1** in order to compare the results with those for the three model excimers. We indeed observe a larger number of energetically distinct excited singlet and triplet states for both dimers as compared to monomeric **1** near the energies of the different S_*n*_ states (*n* > 1) of the monomer (see Figure 6). Even more revealingly, the spin densities of the triplet state T_1_ of **dimer2** and of **tetramer** are localized on just one pyrenyl unit of a single molecule, similar to what we found for the optimized triplet state of the monomer (see Figure 6). These findings together with the experimentally observed vibrational structuring of the triplet emission at any concentration, which is strongly reminiscent of the emission from pyrene monomers, support the notion of a localized pyrene emission. According to powder X-ray diffraction, microcrystalline **1** retains the high order of the crystalline material and therefore the π-stacking interactions. Powdered or crystalline solid samples of **1** likewise emit exclusively by phosphorescence at 77 K, albeit at a shifted wavelength of 681 nm (Figure 5). The different dielectric permittivities of the solid and of the MeTHF glass may contribute to the red-shift of 940 cm^–1^ in the solid state. None of the solid samples are emissive at room temperature, though.

## Conclusion

In conclusion, we have prepared four new mercaptocoumarin- and -pyrene-modified bismuth pincer complexes (*NCN*)Bi(SR)_2_*via* oxidative addition of the respective disulfide to *in situ* generated (*NCN*)Bi(I) species. Irrespective of the excitation wavelength, coumarin complexes **2** and **4** exhibit green phosphorescence at 77 K, whereas coumarin fluorescence is completely quenched. Mercaptopyrene complexes **1** and **3** are likewise stable and pure phosphorescence emitters when excited into their mixed π → π* and n(p(S)) → π*(pyrene) S_0_ → S_1_ HOMO → LUMO band at 77 K. This attests to the efficacy of the HAE of the Bi^3+^ ion to promote intersystem crossing of the attached dye ligands.

Irradiation into electronically higher excited states however results in pure blue pyrene-based fluorescence at sample concentrations *c* < 1 μM. In the concentration range 1–100 μM, the emission profile changes to dual fluorescence and phosphorescence emission. We observe a progressive decrease of the fluorescence intensity at increasing *c*, until the fluorescence completely fades at *c* ≥ 1 mM. Our concentration-dependent emission and excitation spectra support the idea that, at higher *c*, individual complex molecules associate to static excimers, whose higher excited singlet states ultimately populate the phosphorescent state T_1_, while this is not the case for monomeric complexes. We propose that the larger number of excited singlet and triplet states for the excimers and concomitantly reduced energy gaps between higher excited states of the singlet and triplet manifolds increase the efficacy of ISC to ultimately populate the phosphorescent state T_1_.

Appending diarylamine substituents to the cyclometalating *NCN* pincer ligand in complexes **3** and **4** adds additional diarylamine-to-chromophore excitations to their absorption envelope, but has no further impact on their emissive properties. In contrast to their stable emissions at 77 K, room temperature excitation of the four complexes into higher-lying excited electronic states leads to decomposition with the concomitant release of the corresponding disulfide.

## Experimental Section

### General Procedures

All syntheses were performed under an inert nitrogen atmosphere and protection from light, using standard Schlenk techniques. No uncommon hazards are involved, apart from those concomitant with work under cryogenic conditions (−70 °C, cooling baths with *iso*-propanol and dry ice; appropriate protective clothing should be worn). **(*****NCN*****)BiCl**_**2**_(62) was prepared according to literature procedures. ^1^H NMR and ^13^C NMR spectra and mass spectra of compounds **1**–**4** can be found in the Supporting Information. For compound **3**, decomposition was already observed during the acquisition time for recording the ^13^C{^1^H}-NMR spectrum, which explains the additional resonances.

### NMR Spectroscopy

^1^H NMR (400/500/800 MHz) and ^13^C{^1^H}-NMR (101/125/202 MHz) spectra were recorded in CD_2_Cl_2_ at 300 K using a Bruker Avance III 400, Bruker Avance III 500, or a Bruker Avance Neo 800 spectrometer. NMR spectra were referenced to residual protonated solvent (^1^H) or to the solvent signal itself (^13^C). The assignment of signals is based on 2D spectra.

### Mass Spectrometry

Mass spectra of the compounds were recorded in the positive mode on an ESI-calibrated LTQ Orbitrap Velos Spectrometer with the direct injection of their CH_2_Cl_2_ solutions. The use of CH_2_Cl_2_ as the solvent also explains the mass peaks of (*NCN*)BiCl^+^ ions that are observed in the mass spectra of complexes **1**–**4**.

### Powder X-Ray Diffraction

PXRD measurements were performed with a Bruker D8 powder diffractometer with an IμS-XR Source.

### X-Ray Crystallography

A STOE IPDS-II image plate diffractometer equipped with a Mo–Kα radiation source was used for complexes **2** and **(*****NCN*****)***^**DAA**^***BiCl**_**2**_. Data acquisition was conducted at 100 K. The program package X-Area was used for data processing. Depending on the structure, either semiempirical or spherical absorption corrections were performed. The structure of **2** was solved and refined with SHELXT^86,87^ and Olex2,^88^ using least-squares minimization, and refined with olex2.refine.^89^ All non-hydrogen atoms were refined anisotropically. Diffraction data for complex **1** were acquired on an XtaLAB Synergy-DW system with a HyPix-Arc 150° detector and processed in the CrysAlisPro software, using face-indexed absorption corrections in combination with multiscan scaling.^90^ Nonspherical atomic form factors calculated by NoSpherA2,^91^ based on wave functions from ORCA at the x2c-TZVP/R2SCAN level of theory were applied.^92^

### TD-DFT Calculations

The ground state electronic structures of the full models of complexes **1**–**4** were calculated by density functional theory (DFT) methods using the Gaussian 16 program packages.^93^ Open-shell systems were calculated by the unrestricted Kohn–Sham approach (UKS). Geometry optimization followed by vibrational analysis was performed in solvent media. Solvent effects were described by the SMD variation of IEFPCM implemented in the Gaussian program package with standard parameters for CH_2_Cl_2_.^94^ The fully relativistic small-core multiconfiguration-Dirac Hartree–Fock-adjusted pseudopotentials and the corresponding optimized set of basis functions for Bi (ECP60MDF)^95^ and 6-31G(d) polarized double-ζ basis sets^96^ for the remaining atoms were employed together with the Perdew, Burke, Ernzerhof exchange and correlation functional (PBE0).^97,98^ Calculations including spin–orbit coupling were carried out using the ORCA 5.0.4 software package.^99^ For ground states, optimization was performed according to the restricted Kohn–Sham (KS) DFT process. For triplet states, unrestricted KS ground state calculations were performed rather than computing the triplet excited states from TD-DFT with a restricted KS reference. LR-CPCM was used for calculations of dissolved monomeric species **1**, using CH_2_Cl_2_. For the excited states, TD-DFT without TDA was employed. Optimized structures were checked for negative frequencies. The spin–orbit integrals were calculated using the RI-SOMF(1X)^100^ approximation.^101,102^ Calculations were done using the PBE0-function (PBE0)^97,98^ with the SARC-ZORA-TZVP basis set for Bi^103−106^ and the ZORA-def2-TZVP basis^107^ and the SARC/J decontracted def2/J auxiliary basis^108^ for all other elements. The GaussSum program package was used to analyze the results,^109^ while the visualization of the results was performed with the Avogadro program package.^110^ Graphical representations of molecular orbitals were generated with the help of GNU Parallel,^111^ and plotted using the vmd program package^112^ in combination with POV-Ray.^113^

### UV/Vis Spectroscopy

UV/vis spectra of CH_2_Cl_2_ and MeTHF solutions of complexes **1**–**4**, **PyreneS**_**2**_, **CoumarinS**_**2**_ and of pyrene were recorded on a TIDAS fiber optic diode array spectrometer from J&M in HELLMA quartz cuvettes with 1.0 cm optical path lengths.

### Photoluminescence

Luminescence spectra and lifetimes in MeTHF and CH_2_Cl_2_ solutions were measured on a PicoQuant FluoTime 300 spectrometer.

### Synthesis

#### (*NCN*)Bi(SPyrene)_2_ (**1**)

**(*****NCN*****)BiCl**_**2**_(62) (120 mg, 0.25 mmol, 1.00 equiv) was dissolved in 10 mL of dry THF and cooled to −78 °C. A 1.0 M solution of *K-*Selectride (0.51 mL, 0.51 mmol, 2.00 equiv) in THF was added dropwise. The violet reaction mixture, containing the *in situ* formed bismuthinidene complex *(NCN)*Bi was stirred for 40 min at −70 °C. A suspension of pyrenedisulfide, **PyreneS**_**2**_, (118 mg, 0.25 mmol, 1.00 equiv) in 20 mL of dry THF was added. The reaction mixture turned brown. It was slowly warmed to room temperature and stirred for 2 h. Then, the solvent was removed *in vacuo*. The solid was washed with three 15 mL portions of dry *n*-hexane and the remaining solid was extracted with dry benzene (3 × 20 mL). After removal of benzene from the filtered solutions, complex **1** was obtained as an orange solid in a yield of 60% (133 mg, 0.15 mmol). ^1^H NMR (CD_2_Cl_2_, 400 MHz): δ = 8.76 (d, 2H, H^9^, ^3^*J*_HH_ = 9.3 Hz), 8.09–8.03 (m, 4H, H^7,8,10–13^), 7.97–7.90 (m, 4H, H^7,8,10–13^), 7.88 (s, 4H, H^5,6^), 7.81 (d, 2H, H^2^, ^3^*J*_HH_ = 7.5 Hz), 7.77–7.71 (m, 4H, H^7,8,10–13^), 7.65 (t, 1H, H^1^, ^3^*J*_HH_ = 7.5 Hz), 4.50 (s, 4H, H^3^), 2.82 (s, 12H, H^4^) (for atomic numbering, see the Supporting Information). ^13^C{^1^H}-NMR (CD_2_Cl_2_, 101 MHz): δ = 152.4, 133.2, 132.7, 132.0, 131.8, 129.6, 129.1, 128.6, 127.7, 127.3, 126.6, 126.2, 125.9, 125.7, 125.3, 124.8, 124.6, 124.5, 69.7, 48.2. HR ESI-MS (*m*/*z*) in CH_2_Cl_2_: 633.1778, calcd. for BiC_28_H_28_N_2_S^+^ = 633.1772; 435.1018, calcd. for BiC_12_H_19_N_2_Cl^+^ = 435.1035. Elemental analysis: calcd for C_44_H_37_BiN_2_S_2_·C_6_H_6_: C, 63.55; H, 4.59; N, 2.96. Found: C, 63.81; H, 4.87; N, 2.80.

#### (*NCN*)Bi(SCoumarin)_2_ (**2**)

**(*****NCN*****)BiCl**_**2**_(62) (221 mg, 0.47 mmol, 1.00 equiv) was dissolved in 10 mL of dry THF and cooled to −78 °C. A 1.0 M solution of *K-*Selectride (0.94 mL, 0.94 mmol, 2.00 equiv) in THF was added dropwise. The reaction mixture turned violet, indicating formation of bismuthinidene *(NCN)*Bi, and was stirred for 40 min at −70 °C. A suspension of **CoumarinS**_**2**_ (180 mg, 0.47 mmol, 1.00 equiv) in 20 mL of dry THF was added, upon which the reaction mixture turned brown. It was slowly warmed to room temperature and stirred for 2 h. The solvent was removed *in vacuo*. The solid was extracted with 25 mL of toluene, the mixture was filtered, and the solvent was removed from the filtrate *in vacuo*. The solid remaining after toluene evaporation was washed with 20 mL of dry hexane and then recrystallized from CH_2_Cl_2_ (150 mL). **2** was obtained as golden yellow crystals in a yield of 72% (263 mg, 0.34 mmol). ^1^H NMR (CDCl_3_, 400 MHz): δ = 7.69 (d, 2H, H^2^, ^3^*J*_HH_ = 7.4 Hz), 7.56 (t, 1H, H^1^, ^3^*J*_HH_ = 7.4 Hz), 7.36 (d, 2H, H^7^, ^3^*J*_HH_ = 8.3 Hz), 7.15 (dd, 2H, H^6^, ^3^*J*_HH_ = 8.3 Hz, ^4^*J*_HH_ = 1.3 Hz), 7.08 (s, 2H, H^5^), 6.10 (s, 2H, H^9^), 4.42 (s, 4H, H^3^), 2.95 (s, 12H, H^4^), 2.37 (s, 6H, H^8^) (for atomic numbering, see the Supporting Information). ^13^C{^1^H}-NMR (CDCl_3_, 101 MHz): δ = 196.1, 161.3, 153.5, 152.7, 151.8, 149.5, 130.4, 129.7, 129.1, 124.0, 120.8, 116.6, 112.7, 69.4, 48.6, 18.7. HR ESI-MS (*m*/*z*) in CH_2_Cl_2_: 591.1518, calcd. for BiC_22_H_26_N_2_O_2_S^+^ = 591.1513; 435.1050, calcd. for BiC_12_H_19_N_2_Cl^+^ = 435.1035. Elemental analysis: calcd for C_32_H_33_BiN_2_O_4_S_2_: C, 49.10; H, 4.35; N, 3.58. Found: C, 48.89; H, 4.54; N, 3.38.

#### (*NCN*)^*DAA*^BiCl_2_

The reaction and purification were carried out under inert gas conditions and light exclusion. **(*****NCHN*****)***^**DAA**^* was synthesized as detailed in the Supporting Information. **(*NCHN*)*^DAA^*** (1.12 g, 2.90 mmol, 1.00 equiv) was dissolved in *n-*hexane (10 mL) and 1.8 mL of a 1.6 M solution of *n-*BuLi in *n-*hexane (2.90 mmol, 1.00 equiv) were added. The reaction mixture was heated to reflux overnight. The solvent was removed *in vacuo*. The remaining solid was dissolved in 20 mL of Et_2_O and slowly cannulated into a solution of BiCl_3_ (0.92 g, 2.90 mmol, 1.00 equiv) in Et_2_O (15 mL), which was precooled to −78 °C. After stirring for 1 h, the reaction mixture was warmed to room temperature and stirred overnight. The solvent was removed *in vacuo*. The solid was extracted with 50 mL of CH_2_Cl_2_, cannula-filtered and the solvent was removed from the filtrate *in vacuo*. The solid remaining after solvent evaporation was washed with 30 mL of *n*-hexane and was then dried *in vacuo*. **(*NCN*)**^***DAA***^**BiCl**_**2**_ was obtained as a beige solid in a yield of 83% (1.60 g, 2.40 mmol). ^1^H NMR (CD_2_Cl_2_, 400 MHz): δ = 7.15 (s, 2H, H^4^), 7.12 (d, 4H, H^2^, ^3^*J*_HH_ = 8.4 Hz), 7.03 (d, 4H, H^3^), ^3^*J*_HH_ = 8.4 Hz), 4.27 (s, 4H, H^5^), 2.87 (s, 12H, H^6^), 2.32 (s, 6H, H^1^). ^13^C{^1^H}-NMR (CD_2_Cl_2_, 101 MHz): δ = 203.8 (C^e^), 155.3 (C^d^), 150.1 (C^c^), 144.9 (C^b^), 134.4 (C^a^), 130.6 (C^2^), 126.3 (C^3^), 120.0 (C^4^), 68.7 (C^5^), 47.7 (C^6^), 21.0 (C^1^). HR ESI-MS (*m*/*z*) in CH_2_Cl_2_: 1295.3788, calcd. for Bi_2_C_52_H_64_N_6_Cl_3_^+^ = 1295.2860.

#### (*NCN*)^*DAA*^Bi(SPyrene)_2_ (**3**)

Under inert gas conditions and light exclusion, *K-*Selectride (0.30 mL, 1.00 M, 0.30 mmol, 2.00 equiv) was added to a suspension of **(*****NCN*****)***^**DAA**^***BiCl**_**2**_ (100 mg, 0.15 mmol, 1.00 equiv) in dry THF (5 mL) at −78 °C. The suspension was stirred at this temperature for 1 h, during which the violet color of the corresponding bismuthinidene developed. Then, a suspension of **PyreneS**_**2**_ (70.2 mg, 0.15 mmol, 1.00 equiv) in dry THF (25 mL) was slowly added *via* cannula transfer at −70 °C, upon which the reaction mixture turned brown. The mixture was stirred for 30 min at −78 °C and then allowed to warm to room temperature. After stirring for another 2 h at this temperature, the solvent was removed *in vacuo*. The solid was extracted into CH_2_Cl_2_ (2 × 10 mL), the solution was filtered, and the solvent was removed under reduced pressure. The remaining solid was washed with *n*-hexane (3 × 10 mL) and with a mixture of benzene (10 mL) and *n*-hexane (40 mL) in order to remove remaining impurities. Drying of the residue *in vacuo* afforded complex **3** as an orange solid in a yield of 12% (19.7 mg, 0.02 mmol). ^1^H NMR (CD_2_Cl_2_, 400 MHz): δ = 8.71 (d, ^3^*J*_HH_ = 9.3 Hz, 2H, H^Pyr^), 8.08 (d, ^3^*J*_HH_ = 6.5 Hz, 2H, H^Pyr^), 8.02 (d, ^3^*J*_HH_ = 7.0 Hz, 2H, H^Pyr^), 7.96 (td, ^3^*J*_HH_ = 7.6 Hz, ^4^*J*_HH_ = 2.8 Hz, 2H, H^Pyr^), 7.90–7.84 (m, 6H, H^Pyr^), 7.79–7.69 (m, 4H, H^Pyr^), 7.22 (s, 2H, H^4^), 7.17 (d, ^3^*J*_HH_ = 8.4 Hz, 4H, H^2^), 7.10 (d, ^3^*J*_HH_ = 8.4 Hz, 4H, H^3^), 4.30 (s, 4H, H^5^), 2.80 (s, 12H, H^6^), 2.35 (s, 6H, H^1^) (for atomic numbering, see the Supporting Information). HR ESI-MS (*m*/*z*) in CH_2_Cl_2_: 828.2820, calcd. for BiC_42_H_41_N_3_S^+^ 828.2820; 630.2093, calcd. for BiC_26_H_32_N_3_Cl^+^ = 630.2083. No ^13^C{^1^H}-NMR and CHN combustion analysis data could be obtained due to sample decomposition during data acquisition.

#### (*NCN*)^*DAA*^Bi(SCoumarin)_2_ (**4**)

Under inert gas conditions and light exclusion, *K-*Selectride (0.45 mL, 1.00 M, 0.45 mmol, 2.00 equiv) was added to a suspension of **(*****NCN*****)***^**DAA**^***BiCl**_**2**_ (150 mg, 0.23 mmol, 1.00 equiv) in dry THF (8 mL) at −78 °C. After stirring for 1 h, a suspension of **CoumarinS**_**2**_ (86.1 mg, 0.23 mmol, 1.00 equiv) in dry THF (20 mL) was slowly added *via* cannula transfer at −70 °C, upon which the reaction mixture turned brown. The mixture was stirred for 30 min at −70 °C and then allowed to warm to room temperature, where stirring was continued for another 3.5 h. The solvent was removed *in vacuo*. The solid was extracted with CH_2_Cl_2_ (4 × 15 mL). The extracts were cannula-filtered and the solvent was removed from the filtrate under reduced pressure. The remaining solid was washed with *n*-hexane (3 × 15 mL) and toluene (6 × 20 mL) and then dried *in vacuo*. Complex **4** was obtained as a golden yellow solid in a yield of 17% (40.0 mg, 0.04 mmol). ^1^H NMR (CD_2_Cl_2_, 400 MHz): δ = 7.34 (d, ^3^*J*_HH_ = 8.2 Hz, 2H, H^9^), 7.15–7.11 (m, 8H, H^3,7,8^, 7.08 (s, 2H, H^4^) 7.05 (d, ^3^*J*_HH_ = 8.4 Hz, 4H, H^2^), 6.04 (s, 2H, H^11^), 4.21 (s, 4H, H^5^), 2.88 (s, 12H, H^6^), 2.35 (s, 6H, H^10^), 2.33 (s, 6H, H^1^) (for atomic numbering, see the Supporting Information). ^13^C{^1^H}-NMR (CD_2_Cl_2_, 150 MHz): δ = 188.8, 161.1, 154.0, 153.8, 152.9, 150.1, 144.9, 134.4, 130.6, 126.3, 124.1, 121.1, 120.6, 112.7, 69.2, 48.6, 21.0, 18.7. HR ESI-MS (*m*/*z*) in CH_2_Cl_2_: 1295.3793, calcd. for Bi_2_C_52_H_64_N_6_Cl_3_^+^ 1295.2860; 786.2566, calcd. for BiC_36_H_39_N_3_O_2_S^+^ 786.2561; 630.2093, calcd. for BiC_26_H_32_N_3_Cl^+^ = 630.2083. Elemental analysis: calcd for C_46_H_46_BiN_3_O_4_S_2_: C, 56.49; H, 4.74; N, 4.30. Found: C, 56.21; H, 5.09; N, 4.20.

## Supporting Information Available

Synthetic procedures for **PyreneS**_**2**_, **CoumarinS**_**2**_, and ligand ***NCHN*^DAA^**, ^1^H and ^13^C NMR spectra of all compounds; HR ESI mass spectra, relevant XRD data including tabulated bond lengths, interatomic angles, torsion angles, packing diagrams, and details to intermolecular interactions in the crystalline state, PXRD data, DFT-optimized geometries, and TD-DFT computed electronic transitions with associated electron density difference maps, photoluminescence data including emission and excitation spectra measured at different temperatures and concentrations, as well as lifetime measurements (Tables S1–S32 and Figures S1–S60) (PDF)
