# Supplementary material for: Dual Fluorescence and Phosphorescence Emissions from Dye-Modified (NCN)-Bismuth Pincer Thiolate Complexes
Source: Inorg Chem. 2024 Jul 30;63(32):14876–88. doi: 10.1021/acs.inorgchem.4c01023 (PMC11323247; doi:10.1021/acs.inorgchem.4c01023)
Supplement: Supplementary file 1 — ic4c01023_si_001.pdf [file ic4c01023_si_001.pdf]

Supporting Information

Dual Fluorescence and Phosphorescence Emissions from Dye-Modified (*NCN*)-Bismuth Pincer Thiolate Complexes

Marcel Geppert, Kai Jellinek, Michael Linseis, Michael Bodensteiner, Jessica Geppert, Miriam M. Unterlass, and Rainer F. Winter*

Corresponding author's e-mail address: Rainer.Winter@uni-konstanz.de

Table of Contents

Synthesis 6

NMR Spectra 8

ESI-MS Data 19

Single Crystal X-Ray Diffraction Data 24

TD-DFT Data 44

Photoluminescence Data 97

Literature 111

List of Figures

Figure S1. ^{1}H-NMR spectrum of **CoumarinS$_2$** in $CDCl_3$. 8
Figure S2. ^{13}C{^{1}H}-NMR spectrum of **CoumarinS$_2$** in $CDCl_3$. 8
Figure S3. ^{1}H-NMR spectrum of **PyreneS$_2$** in $CDCl_3$. 9
Figure S4. ^{13}C{^{1}H}-NMR spectrum of **PyreneS$_2$** in $CDCl_3$. 9
Figure S5. ^{1}H-NMR spectrum of **(NCHN)^{DAA}** in $CDCl_3$. 10
Figure S6. ^{13}C{^{1}H}-NMR spectrum of **(NCHN)^{DAA}** in $CDCl_3$. 10
Figure S7. ^{1}H-NMR spectrum of **(NCN)^{DAA}BiCl$_2$** in CD_2Cl_2. 11
Figure S8. ^{13}C{^{1}H}-NMR spectrum of **(NCN)^{DAA}BiCl$_2$** in CD_2Cl_2. 11
Figure S9. ^{1}H-NMR spectrum of **1** in CD_2Cl_2. 12
Figure S10. ^{13}C{^{1}H}-NMR spectrum of **1** in CD_2Cl_2. 12
Figure S11. ^{1}H-NMR spectrum of **2** in $CDCl_3$. 13
Figure S12. ^{13}C{^{1}H}-NMR spectrum of **2** in $CDCl_3$. 13
Figure S13. ^{1}H-NMR spectrum of **3** in CD_2Cl_2. 14
Figure S14. ^{13}C{^{1}H}-NMR spectrum of **3** and of decomposition products formed during data acquisition. 14
Figure S15. Photodecomposition of **3** monitored by ^{1}H-NMR spectroscopy in CD_2Cl_2 (~20 mM under t ambient conditions). 15
Figure S16. Photodecomposition of **3** monitored by ^{13}C{^{1}H}-NMR spectroscopy in CD_2Cl_2 (~20 mM under ambient conditions). 15
Figure S17. ^{1}H-NMR spectrum of **4** in CD_2Cl_2. 16
Figure S18. ^{13}C{^{1}H}-NMR spectrum of **4** in CD_2Cl_2. 16
Figure S19. Phtoinduced decomposition of **2**. a) ^{1}H-NMR spectrum of **2** in $CDCl_3$; b) ^{1}H-NMR spectrum of **2** (~10 mM) after irradiation at λ = 365 nm (2.4 W) for 5 minutes at room temperature in $CDCl_3$; c) ^{1}H-NMR spectrum of pure **CoumarinS$_2$** in $CDCl_3$. 17

Figure S20. Photoinduced decomposition of **1.** a) ^{1}H-NMR spectrum of **PyreneS$_2$** in $CDCl_3$; b) ^{1}H-NMR spectrum of **1** (~10 mM) after irradiation atλ = 365 nm (2.4 W) for 5 minutes at room temperature in $CDCl_3$; c) ^{1}H-NMR spectrum of **1** in $CDCl_3$. 18
Figure S21. ESI-MS of complex **(NCN)^{DAA}BiCl$_2$** in CH_2Cl_2 (experimental data in black, calculated in blue). 19
Figure S22. ESI-MS of complex **1** in CH_2Cl_2 (experimental data in black, calculated in blue). 20
Figure S23. ESI-MS of complex **2** in CH_2Cl_2 (experimental data in black, calculated in blue). 21
Figure S24. ESI-MS of complex **3** in CH_2Cl_2 (experimental data in black, calculated in blue). 22
Figure S25. ESI-MS of complex **4** in CH_2Cl_2 (experimental data in black, calculated in blue). 23
Figure S26. Packing diagrams of complex **(NCN)^{DAA}BiCl$_2$** viewed along a) the a-axis, and b) the b-axis of the unit cell. 28
Figure S27. Molecular packing and relevant interatomic contacts of **(NCN)^{DAA}BiCl$_2$**. 28
Figure S28. Packing of molecules of complex **2** in the crystal viewed along a) the c-axis of the unit cell, and b) along the a-axis of the unit cell. 35
Figure S29. The pattern of hydrogen bonds and π-stacking interactions in crystalline **2** viewed along the a-axis of the unit cell. 36
Figure S30. The pattern of hydrogen bonding interactions in crystalline **2**. 36
Figure S31. Packing of molecules of complex **1** in the crystal viewed along a) the b-axis of the unit cell, and b) along the c-axis of the unit cell. 41
Figure S32. The pattern of π-stacking interactions in crystalline **1**. 42
Figure S33. PXRD of **1** along with comparison between the experimental (black line) and the calculated (blue line) pattern based on the X-ray structure determination on a single crystal. 42
Figure S34. Left: Geometry-optimized structure of the transoid conformer of complex **1** along with comparison between the experimental (black line) and the TD-DFT-computed (blue line) electronic absorption spectra. Individual electronic transitions are indicated as colored bars. Right: MO diagrams of relevant molecular orbitals involved in the individual TD-DFT-computed electronic transitions along with the corresponding electron density difference maps. A loss of electron density is indicated in blue, a gain in red color. 44
Figure S35. Left: Geometry-optimized structure of complex **2** along with comparison between the experimental (black line) and the TD-DFT-computed (blue line) electronic absorption spectra. Individual electronic transitions are indicated as colored bars. Right: MO diagrams of relevant molecular orbitals involved in the individual TD-DFT-computed electronic transitions along with the corresponding electron density difference maps. A loss of electron density is indicated in blue, a gain in red color. 45
Figure S36. Left: Geometry-optimized structure of transoid conformer of complex **3** along with comparison between the experimental (black line) and the TD-DFT-computed (blue line) electronic absorption spectra. Individual electronic transitions are indicated as colored bars. Right: MO diagrams of relevant molecular orbitals involved in the individual TD-DFT-computed electronic transitions along with the corresponding electron density difference maps. A loss of electron density is indicated in blue, a gain in red color. 46
Figure S37. Left: Geometry-optimized structure of cisoid conformer of complex **3** along with comparison between the experimental (black line) and the TD-DFT-computed (blue line) electronic absorption spectra. Individual electronic transitions are indicated as colored bars. Right: MO diagrams of relevant molecular orbitals involved in the individual TD-DFT-computed electronic transitions along with the corresponding electron density difference maps. A loss of electron density is indicated in blue, a gain in red color. 47
Figure S38. Left: Geometry-optimized structure of complex **4** along with comparison between the experimental (black line) and the TD-DFT-computed (blue line) electronic absorption spectra. Individual electronic transitions are indicated as colored bars. Right: MO diagrams of relevant molecular orbitals involved in the individual TD-DFT-computed electronic transitions along with the

corresponding electron density difference maps. A loss of electron density is indicated in blue, a gain in red color........ 48
Figure S39. pbe0/SARC-ZORA-TZVP-calculated molecular orbitals of the ground state S_0 of monomeric **1** at the structure parameters taken from the SXRD-measurement. 61
Figure S40. pbe0/SARC-ZORA-TZVP-optimized molecular orbitals of the geometry-optimized triplet state T_1 of monomeric **1** with CPCM(CH_2Cl_2) correction. 62
Figure S41. pbe0/SARC-ZORA-TZVP-calculated molecular orbitals of the triplet state T_1 of monomeric **1** at the structure taken from the SXRD-measurement........ 63
Figure S42. pbe0/SARC-ZORA-TZVP-calculated molecular orbitals of the triplet state T_1 of the **dimer1**. Structure parameters were taken from the SXRD-measurement. 64
Figure S43. pbe0/SARC-ZORA-TZVP-calculated molecular orbitals of the triplet state T_1 of the **dimer2**. Structure parameters were taken from the SXRD-measurement. 65
Figure S44. pbe0/SARC-ZORA-TZVP-calculated molecular orbitals of the triplet state T_1 of the **tetramer** model. Structure parameters were taken from the SXRD-measurement........ 66
Figure S45. Triplet state spin densities of **dimer1**, **dimer2**, **tetramer**, and monomeric **1** with structure parameters taken from the X-ray experiment (SXRD), and of the geometry-optimized monomer **1**. .. 67
Figure S46. PL Data of **2** at 77 K in MeTHF. a) Emission (red) and excitation spectra (blue) of **2**; b) Lifetime of the 509 nm emission of **2**. 97
Figure S47. PL Data of **4** at 77 K in MeTHF. a) Emission (red) and excitation spectra (blue) of **4**; b) Lifetime of the 509 nm emission of **4**. 98
Figure S48. PL Data of **CoumarinS$_2$** at 77 K in MeTHF. a) Emission (red) and excitation spectra (blue) of **CoumarinS$_2$**; b) Lifetime of the 509 nm emission of **2**; c) Emission spectra of **CoumarinS$_2$** (blue) and **2** (orange) at room temperature........ 99
Figure S49. PL Data of **2** and **4** at room temperature in CH_2Cl_2 a) Absorption (black) and emission (red and orange) of **2**; b) Absorption (black) and emission spectra of **4** (red and orange)........ 100
Figure S50. Lifetime measurements for the products formed after photodecomposition of **2** and **4** upon irradiation in degassed CH_2Cl_2 (λ = 352 nm, output power 4 W, 5 min). a) Lifetime of the 393 nm emission of decomposed **2**; b) Lifetime of the 405 nm emission of decomposed **4**; c) Lifetime of the 392 nm emission of **CoumarinS$_2$**. 101
Figure S51. Partial decomposition of **2** and **4** after photoirradiation in degassed CH_2Cl_2 (λ = 352 nm, output power 4 W, 3 min). Top: Absorption (black), emission (red) and excitation spectra (blue) of decomposed **2**; bottom: Absorption (black), emission (red) and excitation spectra (blue) of **CoumarinS$_2$**; b) Top: Absorption (black), emission (red) and excitation spectra (blue) of decomposed **4**; bottom: Absorption (black), emission (red) and excitation spectra (blue) of **CoumarinS$_2$**........ 102
Figure S52. Decomposition of **1** and **3** in degassed CH_2Cl_2 (λ = 340 nm, output power 300 W, 3 min). at room temperature (PL). Top: Absorption (black), emission (red) and excitation spectra (blue) of decomposed **3**; middle: Absorption (black), emission (red) and excitation spectra (blue) of decomposed **1**; bottom: Absorption (black), emission (red) and excitation spectra (blue) of **PyreneS$_2$**.
.... 103
Figure S53. Lifetime measurements of the products formed after decomposition of **1** and **3** after irradiation in degassed CH_2Cl_2 (λ = 352 nm, output power 4 W, 5 min). a) Lifetime of 388 nm emission of decomposed **1**; b) Lifetime of 387 nm emission of decomposed **3**; c) Lifetime of 387 nm emission of **PyreneS$_2$**........ 104
Figure S54. Photodecomposition experiments of **3**. Spectra recorded before and after irradiation in MeTHF (PL) (λ = 340 nm, output power 300 W, 10 min). Top panel (black): Emission spectrum ($\lambda_{exc.}$ = 340 nm) of **3** at 77 K in MeTHF before irradiation; second from top (gray): Emission spectrum ($\lambda_{exc.}$ = 340 nm) of **3** at 77 K in MeTHF after irradiation (λ = 340 nm, output power 300 W, 30 min) at 77 K for 10 min. The complex has remained intact; middle (green): Emission spectrum ($\lambda_{exc.}$ = 340 nm) of **3** at 77 K in MeTHF after irradiation at room temperature in solution for 5 minutes at 340 nm, output power 300 W, showing additional peaks due to partial photodecomposition; second

from bottom (turquoise): Emission spectrum ($\lambda_{exc.}$ = 340 nm) of **3** at 77 K in MeTHF after irradiation at room temperature in solution for 10 minutes at λ = 340 nm, output power 300 W, indicating major decomposition; bottom panel (blue): Emission spectrum ($\lambda_{exc.}$ = 340 nm) of **PyreneS$_2$** at 77 K in MeTHF. 105
Figure S55. PL Data of **PyreneS$_2$** at 77 K in MeTHF: a) Emission (red) and excitation spectrum (blue) of **PyreneS$_2$**; b) Lifetime of the 400 nm emission of **PyreneS$_2$** c) Emission spectra of **PyreneS$_2$** (yellow) and of complex **1** (orange). 106
Figure S56. a) Concentration-dependent emission spectra of pyrene excited at 335 nm in MeTHF at 77 K; b) Concentration-dependent emission of pyrene excited at 330 nm in CH_2Cl_2 at room temperature. 107
Figure S57. PL Data of pyrene at room temperature in CH_2Cl_2 at different concentrations: Absorption (black), emission (red) and excitation spectra (blue) of pyrene at a concentration of a) 3-5 μM, b) 1 mM, and c) 10 mM. 108
Figure S58. PL Data of complexes **1** and **3** at 77 K in MeTHF: Emission (red) and excitation spectra (green and blue) of a) complex **1** and b) complex **3** under "standard" conditions (3 to 5 μM solutions). c) Lifetimes of the 395 nm emission of **1**; d) Lifetime of 393 nm emission of **3**; e) Lifetime of 640 nm emission of **1**; f) Lifetime of 640 nm emission of **3**. 109
Figure S59. Concentration-dependent emission spectra of complex **3** excited at 340 nm in MeTHF at 77 K. 110
Figure S60. Concentration-dependent excitation spectra of **1** detected at 640 nm in MeTHF at 77 K. 110

List of Tables

Table S1. Crystal data and structure refinement for **(NCN)^{DAA}BiCl$_2$**. 24
Table S2. Bond Lengths [Å] for **(NCN)^{DAA}BiCl$_2$**. 25
Table S3. Bond Angles [°] for **(NCN)^{DAA}BiCl$_2$**. 26
Table S4. Torsion Angles [°] for **(NCN)^{DAA}BiCl$_2$**. 27
Table S5. Crystal data and structure refinement for **2**. 29
Table S6. Bond Lengths [Å] for **2**. 30
Table S7. Bond Angles [°] for **2**. 31
Table S8. Torsion Angles [°] for **2**. 33
Table S9. Crystal data and structure refinement for **1**. 37
Table S10. Bond Lengths [Å] for **1**. 38
Table S11. Bond Angles [°] for **1**. 39
Table S12. Torsion Angles [°] for **1**. 40
Table S13. Selected interatomic distances (Å) and angles (deg) for **1**, **2** and **(NCN)^{DAA}BiCl$_2$**. 43
Table S14. XYZ coordinates of the DFT-optimized structure of the cisoid conformer of complex **1**. 49
Table S15. XYZ coordinates of the DFT-optimized structure of the transoid conformer of complex **1**. 51
Table S16. XYZ coordinates of the DFT-optimized structure of the transoid conformer of complex **2**. 53
Table S17. XYZ coordinates of the DFT-optimized structure of the cisoid conformer of complex **3**. 55
Table S18. XYZ coordinates of the DFT-optimized structure of the transoid conformer of complex **3**. 57
Table S19. XYZ coordinates of the DFT-optimized structure of complex **4**. 59
Table S20. Energies of singlet and triplet states of geometry-optimized monomer **1**. 68
Table S21. Energies of singlet and triplet states of **1** with structure parameters taken from the SXRD-measurement 69

Table S22. Energies of singlet and triplet states of **dimer1**, structure parameters taken from the SXRD-measurement. 70
Table S23. Energies of singlet and triplet states of **dimer2**, structure parameters taken from the SXRD-measurement. 71
Table S24. pbe0/SARC-ZORA-TZVP-calculated transition states of geometry-optimized monomeric **1**. 72
Table S25. pbe0/SARC-ZORA-TZVP-calculated transition states of **1** with structure parameters taken from the SXRD-measurement. 74
Table S26. pbe0/SARC-ZORA-TZVP-calculated transition states of **dimer1**, with structure parameters taken from the SXRD-measurement. 76
Table S27. pbe0/SARC-ZORA-TZVP-calculated transition states of **dimer2**, with structure parameters taken from the SXRD-measurement.. 78
Table S28. XYZ coordinates of pbe0/SARC-ZORA-TZVP geometry-optimized **1**. 81
Table S29. XYZ coordinates of pbe0/SARC-ZORA-TZVP-calculated **1**, with structure parameters taken from the SXRD-measurement. 83
Table S30. XYZ coordinates of pbe0/SARC-ZORA-TZVP-calculated **dimer1**, with structure parameters taken from the SXRD-measurement. 85
Table S31. XYZ coordinates of pbe0/SARC-ZORA-TZVP-calculated **dimer2**, with structure parameters taken from the SXRD-measurement. 88
Table S32. XYZ coordinates of pbe0/SARC-ZORA-TZVP-calculated **tetramer**, with structure parameters taken from the SXRD-measurement. 91

Synthesis

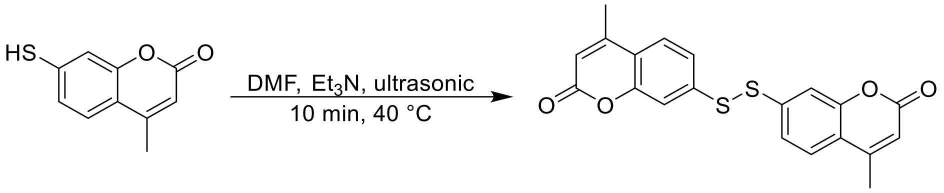

The synthesis of coumarin disulfide was performed according to a literature-known procedure for the oxidation of thiols to disulfides.[1] 7-Mercapto-4-methyl-2H-chromen-2-one[2] (192 mg, 1.00 mmol, 1.00 eq) was dissolved in 20 mL of DMF. 1.0 mL of Et_3N was added and the mixture was placed for 10 min in an ultrasonic bath at 40 °C. The solvent was removed under reduced pressure and coumarin disulfide was obtained as a light yellow solid (180 mg, 0.470 mmol) in a yield of 94%.

^{1}H-NMR ($CDCl_3$, 400 MHz) δ [ppm]: 7.54 (d, $^3J_{HH}$ = 8.4 Hz, 1H, H^3), 7.43 (d, $^4J_{HH}$ = 1.9 Hz, 1H, H^1), 7.38 (dd, $^3J_{HH}$ = 8.4 Hz, $^4J_{HH}$ = 1.9 Hz, 1H, H^2), 6.25 (q, $^4J_{HH}$ = 1.3 Hz, 1H, H^4), 2.41 (d, $^4J_{HH}$ = 1.3 Hz, 3H, H^5).

^{13}C{^{1}H}-NMR ($CDCl_3$, 101 MHz) δ [ppm]: 160.3 (C^e), 154.1 (C^d), 152.0 (C^a), 141.0 (C^c), 125.4 (C^3), 122.2 (C^2), 119.2 (C^b), 115.1 (C^4), 114.5 (C^1), 18.8 (C^5).

Elemental analysis: Calcd. for $C_{20}H_{14}O_4S_2$: C, 62.81; H, 3.69. Found: C, 62.44; H, 3.99.

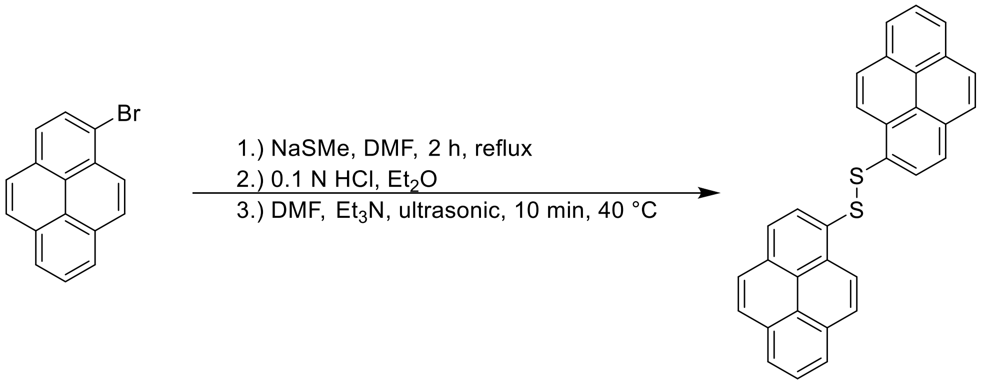

The synthesis of pyrene disulfide was performed according to a literature-known procedure for the oxidation of thiols to disulfides.[1] 1-Pyrenethiol was synthesized according to a literature-known procedure as follows:[3] 1-Bromopyrene (1.41 g, 5.00 mmol, 1.00 eq) and sodium methanethiolate (1.75 g, 25.0 mmol, 5.00 eq) were dissolved in 200 mL of dry DMF under nitrogen atmosphere and heated to reflux for 2 h. After allowing to cool to room temperature, the solvent was removed *in vacuo*. 50 ml of 0.1 M HCl were added and the mixture was extracted with diethyl ether (3 x 150 ml). After washing the organic phase with brine (2 x 50 ml), drying over $MgSO_4$ and removing all volatiles, the crude intermediate product was dissolved in 30 mL of DMF. 1.5 mL of Et_3N were added and the mixture was placed for 30 min in an ultrasonic bath at 40 °C. The solvent was removed under reduced pressure. Pyrenedisulfide was obtained as a yellow solid after recrystallization from CH_2Cl_2 (664 mg, 1.42 mmol) in 56% yield.

^{1}H-NMR ($CDCl_3$, 500 MHz) δ [ppm]: 8.48 (d, $^3J_{HH}$ = 9.2 Hz, 1H), 8.91 (d, $^3J_{HH}$ = 7.6 Hz, 1H), 8.15-8.07 (m, 3H), 8.00 (d, $^3J_{HH}$ = 7.6 Hz, 2H), 7.79 (d, $^3J_{HH}$ = 7.9 Hz, 1H), 7.90 (d, $^3J_{HH}$ = 9.2 Hz, 1H).

^{13}C{^{1}H}-NMR ($CDCl_3$, 125 MHz) δ [ppm]: 132.1, 132.1, 131.4, 131.3, 131.2, 130.9, 128.5, 128.3, 127.3, 126.4, 125.8, 125.7, 125.3, 125.0, 124.6, 124.5.

Elemental analysis: Calcd. for $C_{32}H_{18}S_2$: C, 82.37; H, 4.17. Found: C, 81.92; H, 4.17.

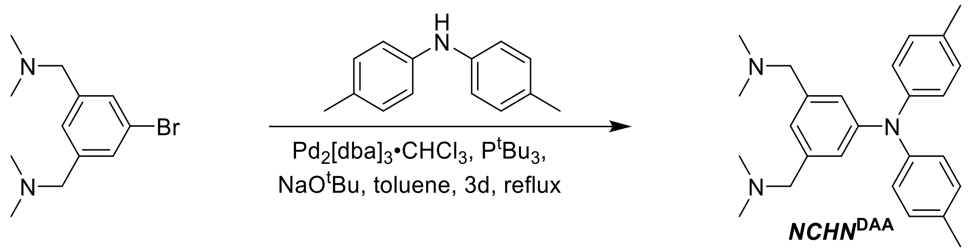

The reaction was carried out under inert gas atmosphere. 1-Bromo-3,5-bis(dimethylaminomethyl)benzene[4] (1.71 g, 6.31 mmol, 1.00 eq), di(*p*-tolyl)amine (1.25 g, 6.31 mmol, 1.00 eq), NaO^{t}Bu (1.84 g, 19.15 mmol, 3.00 eq), [$Pd_2(dba)_3$]×$CHCl_3$ and a 10% solution of P^{t}Bu$_3$ in *n*-hexane (2.82 mL, 0.95 mmol, 0.15 eq) were dissolved in toluene (10 mL). The reaction mixture was heated to reflux for 3 d. The mixture was then filtered through a pad of Celite and the solvent was removed *in vacuo*. The crude product was dissolved in *n*-pentane and the insoluble materials were filtered off. After removing the solvent *in vacuo*, the product was purified by column chromatography (*n*-pentane/CH_2Cl_2 3/1-0/1 with 3% Et_3N). **(*NCHN*)*^{DAA}*** was obtained as a brownish oil (2.20 g, 5.68 mmol, 90% yield).

^{1}H-NMR ($CDCl_3$, 400 MHz) δ [ppm]: 7.02 (d, $^3J_{HH}$ = 8.3 Hz, 4H, H^2), 6.94 (d, $^3J_{HH}$ = 8.6 Hz, 4H, H^3), 6.88 (s, 3H, $H^{4,7}$), 3.31 (s, 4H, H^5), 2.30 (s, 6H, H^1), 2.20 (s, 12H, H^6).

^{13}C{1H}-NMR ($CDCl_3$, 101 MHz) δ [ppm]: 148.3 (C^c), 145.7 (C^b), 139.6 (C^f), 132.1 (C^a), 129.9 (C^2), 124.3 (C^3), 124.1 (C^d), 123.2 (C^4), 64.2 (C^5), 45.3 (C^6), 20.9(C^1).

NMR Spectra

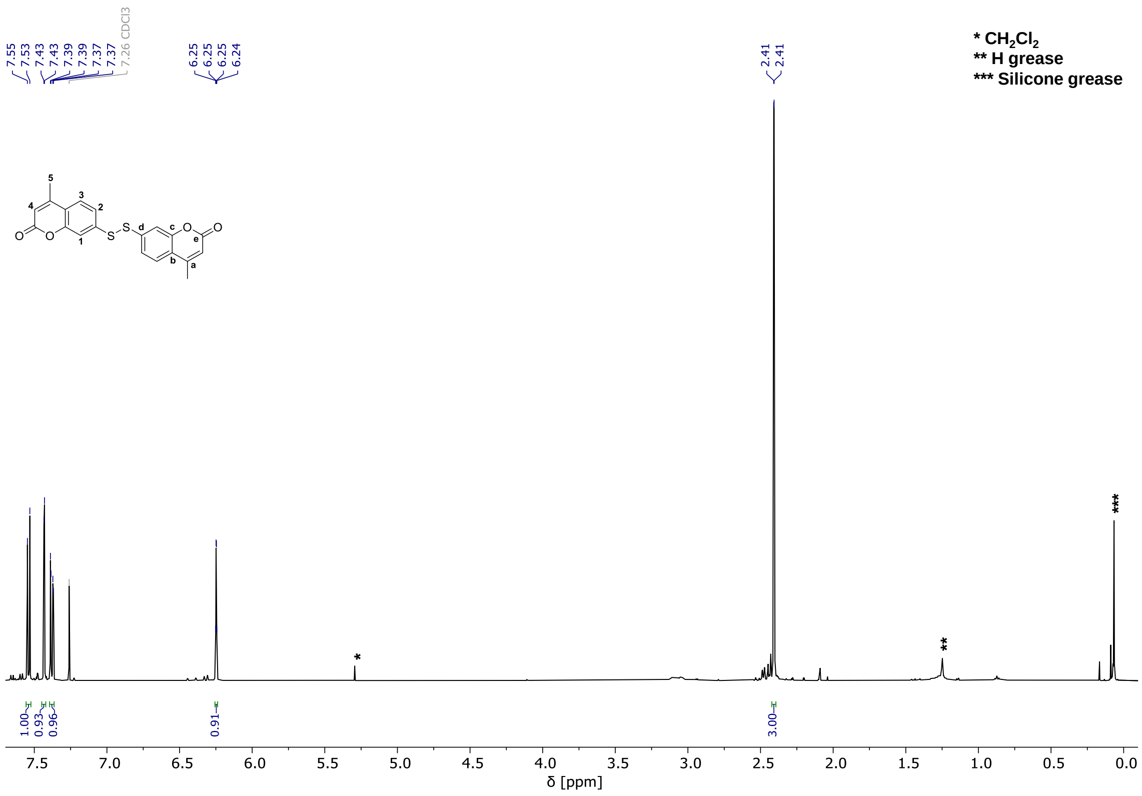

Figure S1. ^{1}H-NMR spectrum of **CoumarinS$_2$** in $CDCl_3$.

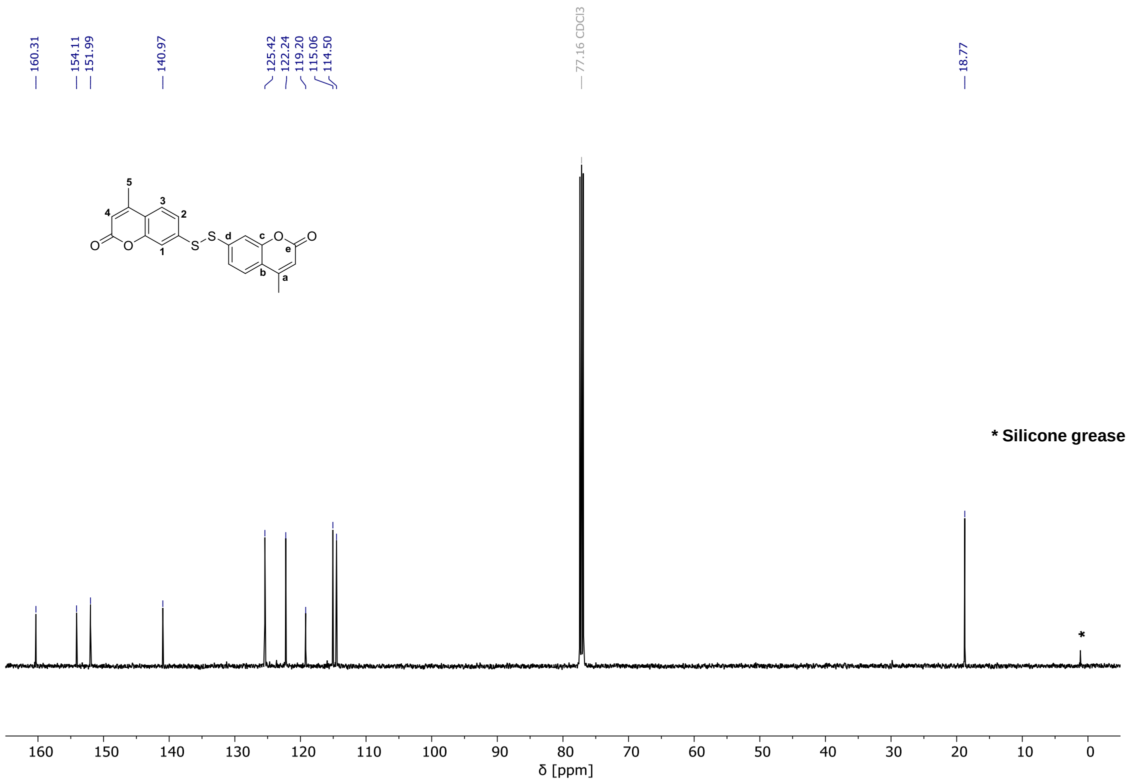

Figure S2. ^{13}C{^{1}H}-NMR spectrum of **CoumarinS$_2$** in $CDCl_3$.

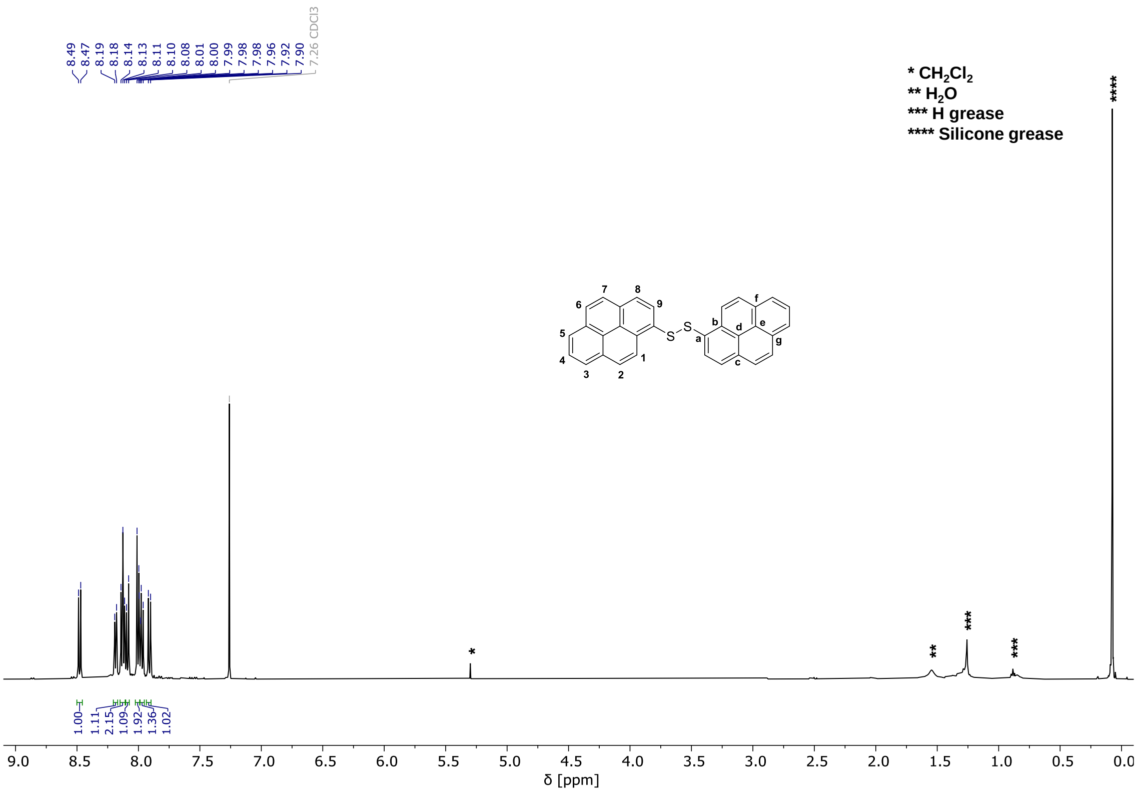

Figure S3. ^{1}H-NMR spectrum of **PyreneS$_2$** in $CDCl_3$.

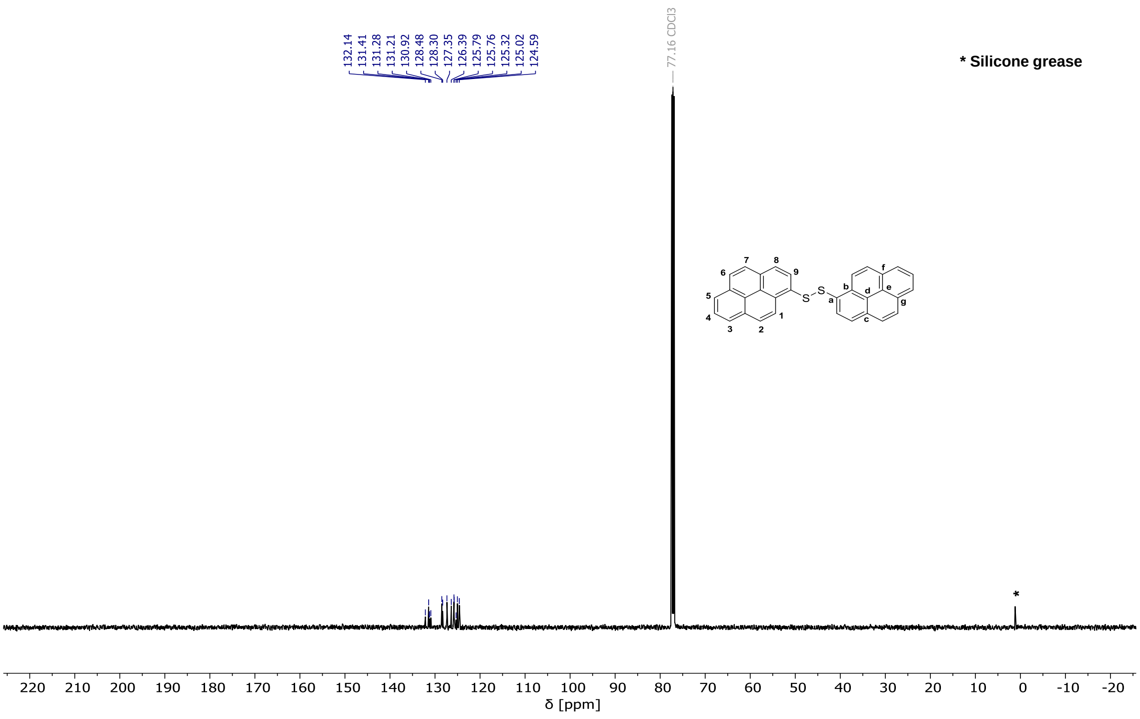

Figure S4. $^{13}C\{^{1}H\}$-NMR spectrum of **PyreneS$_2$** in $CDCl_3$.

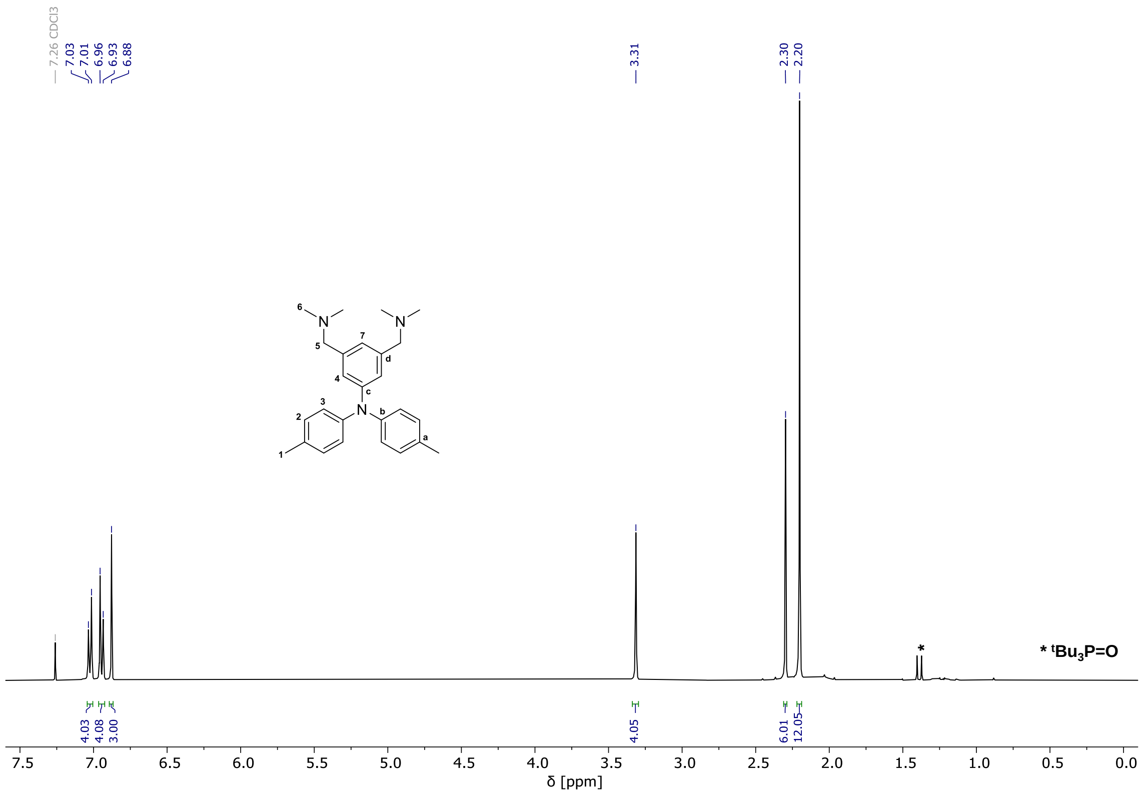

Figure S5. ^{1}H-NMR spectrum of **(*NCHN*)*^{DAA}*** in $CDCl_3$.

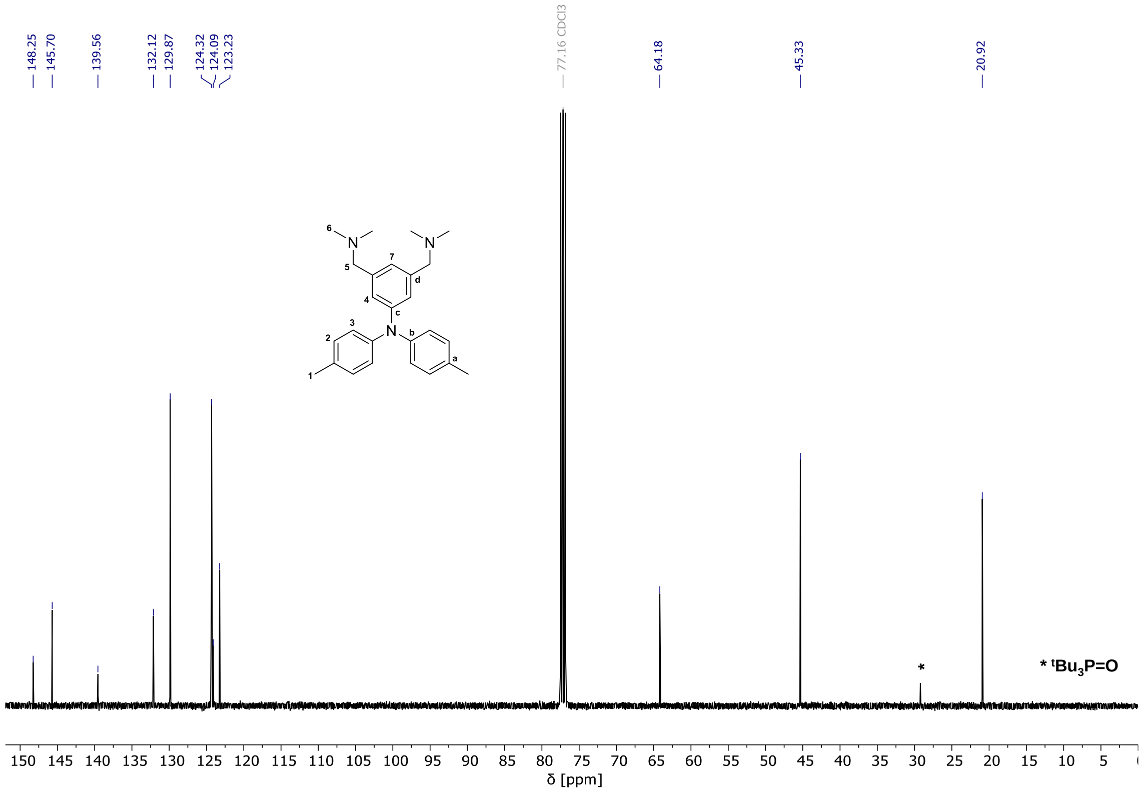

Figure S6. ^{13}C{^{1}H}-NMR spectrum of **(*NCHN*)*^{DAA}*** in $CDCl_3$.

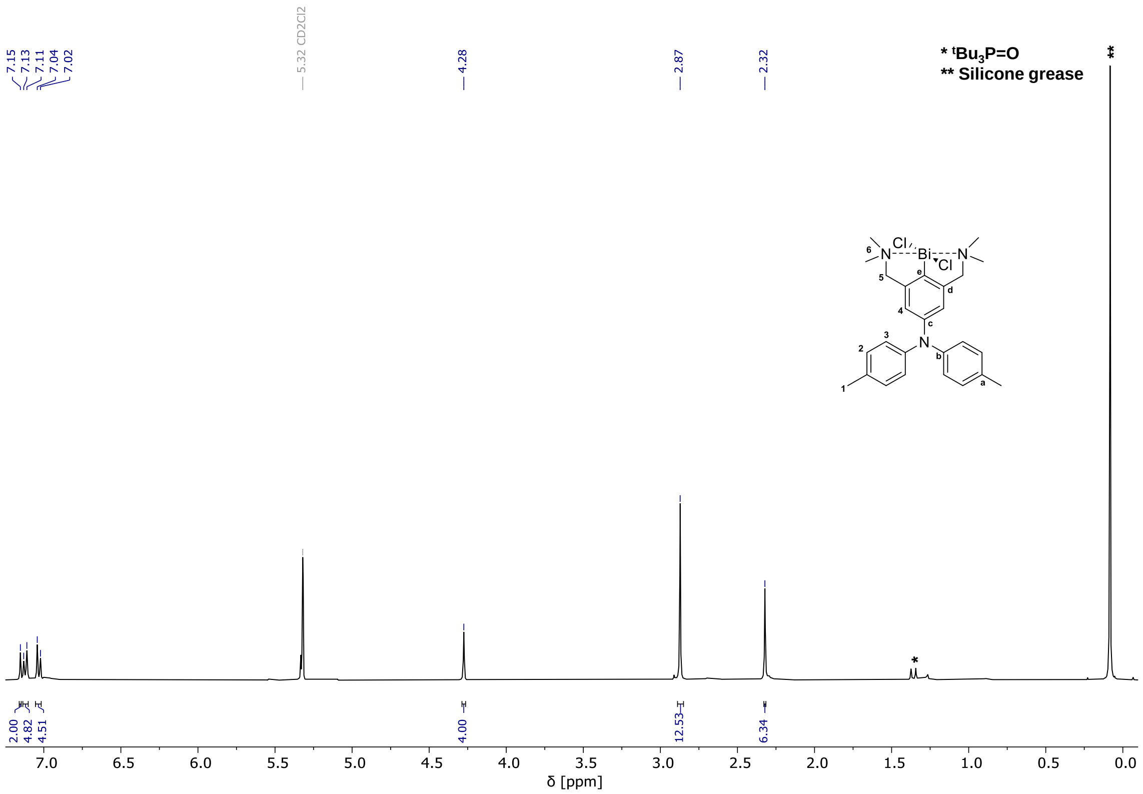

Figure S7. ^{1}H-NMR spectrum of **(*NCN*)^{DAA}BiCl$_2$** in CD_2Cl_2.

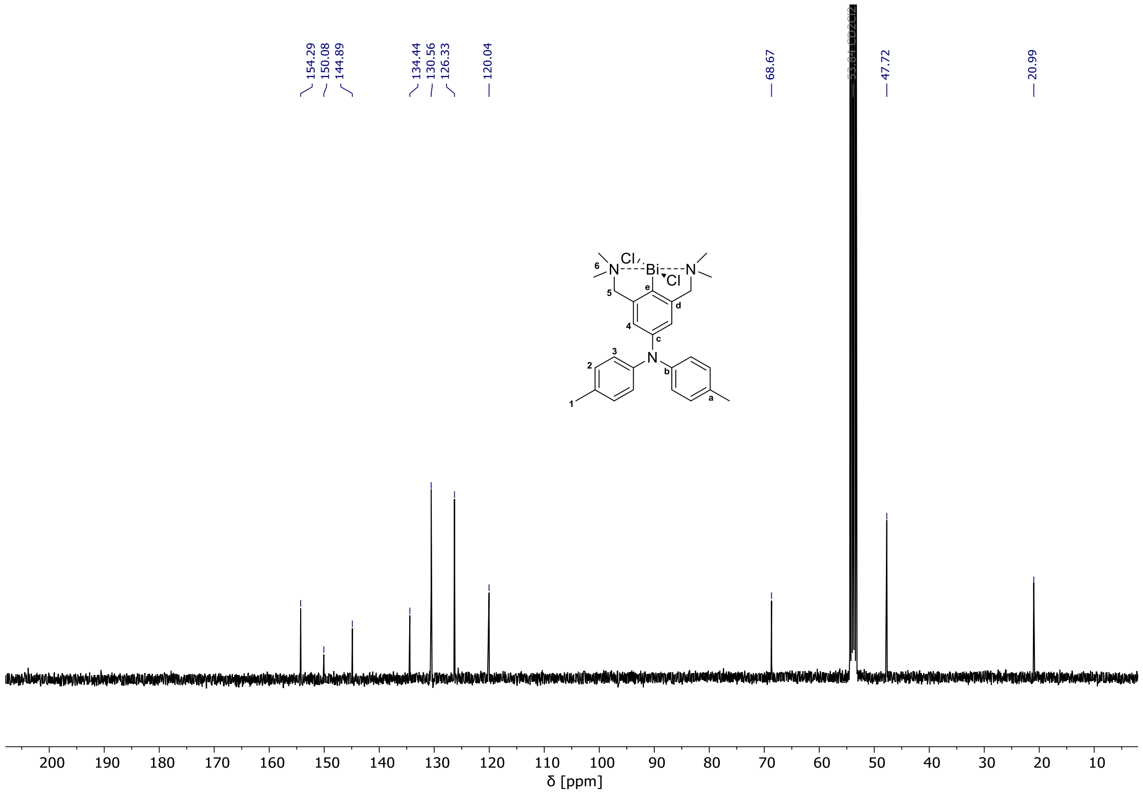

Figure S8. ^{13}C{^{1}H}-NMR spectrum of **(*NCN*)^{DAA}BiCl$_2$** in CD_2Cl_2.

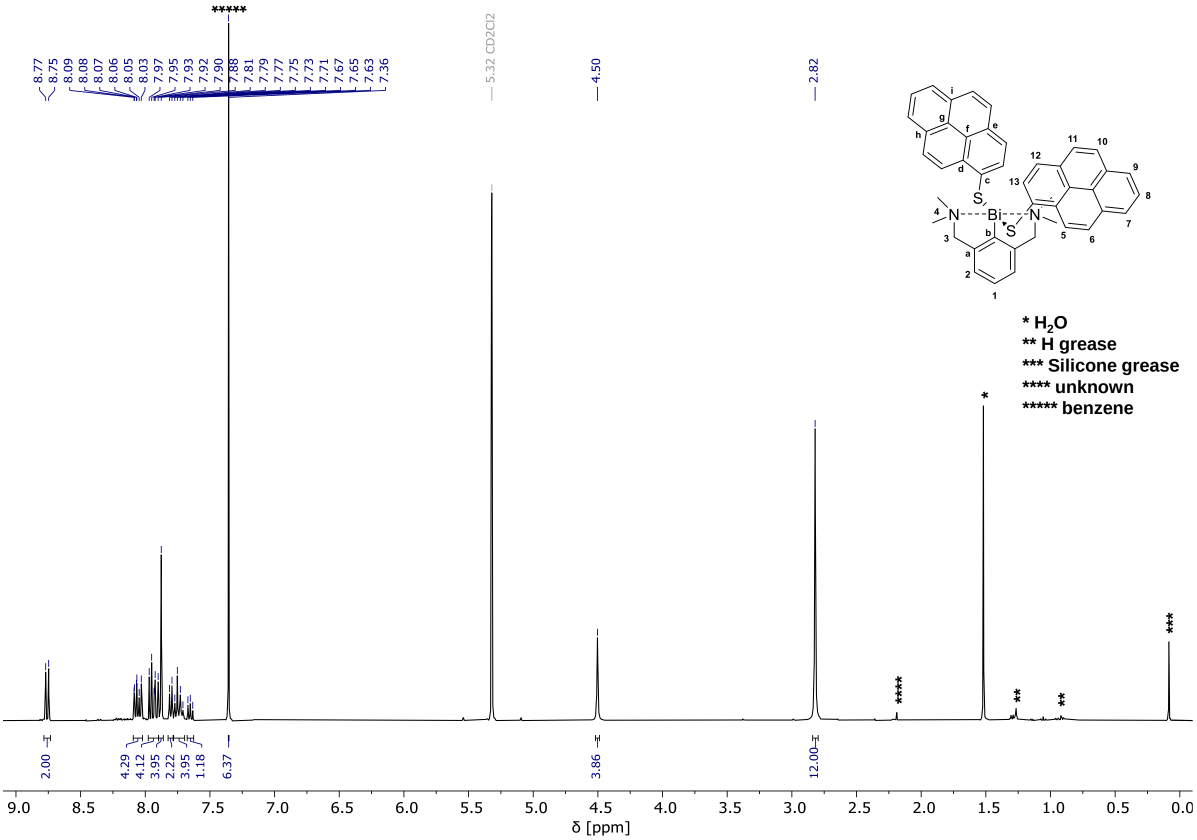

Figure S9. ^{1}H-NMR spectrum of complex **1** in CD_2Cl_2.

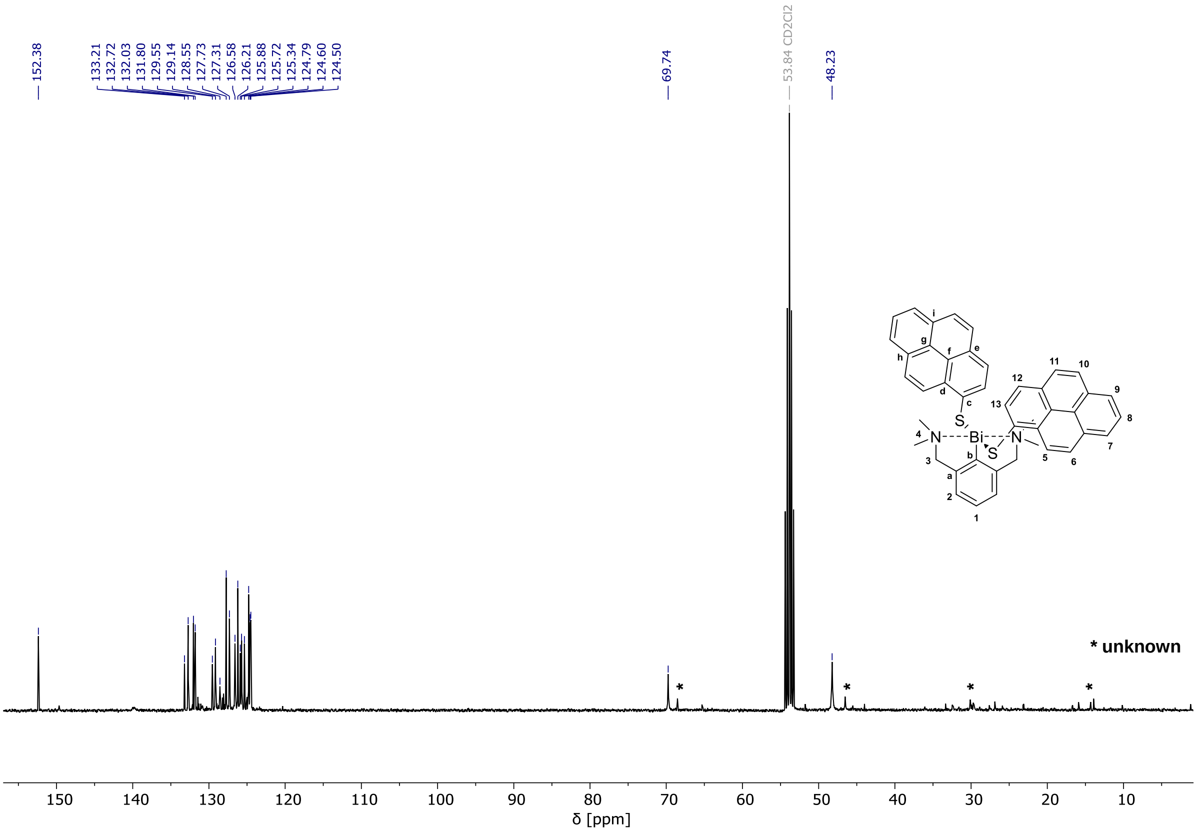

Figure S10. $^{13}C\{^{1}H\}$-NMR spectrum of complex **1** in CD_2Cl_2.

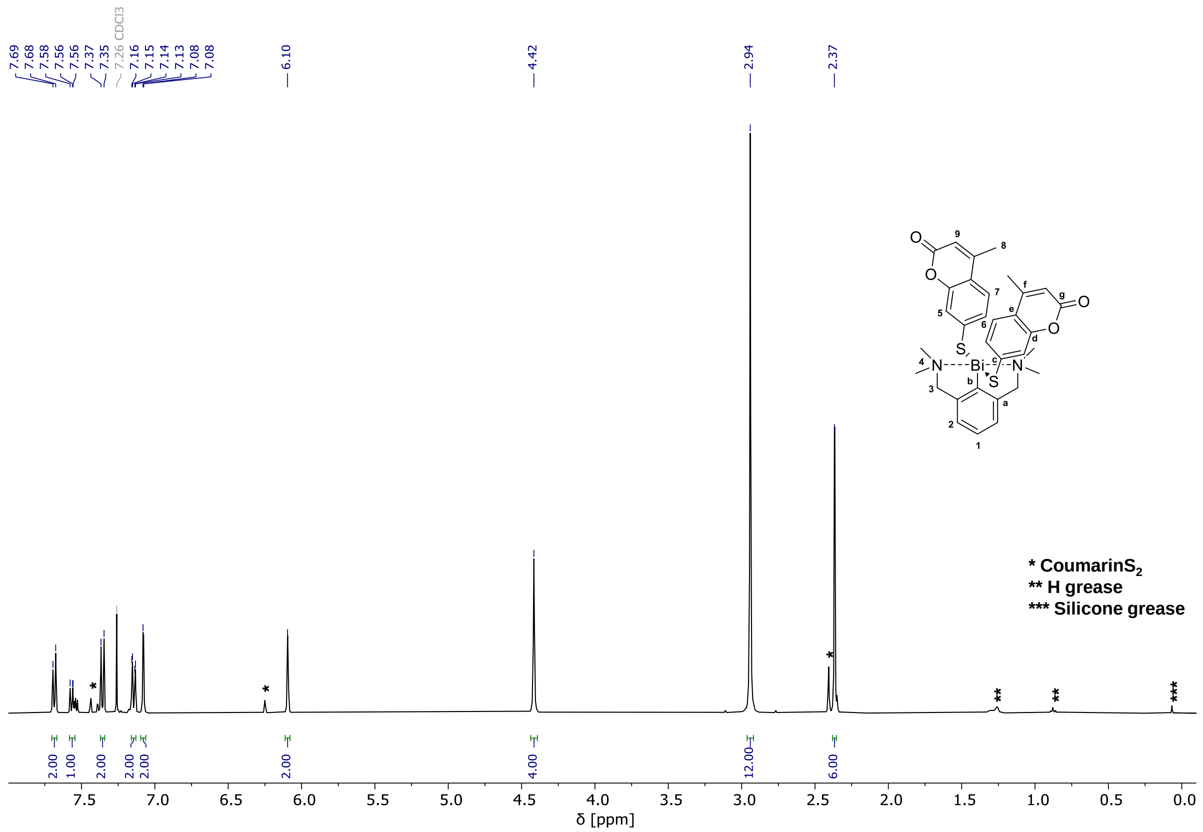

Figure S11. ^{1}H-NMR spectrum of complex **2** in $CDCl_3$.

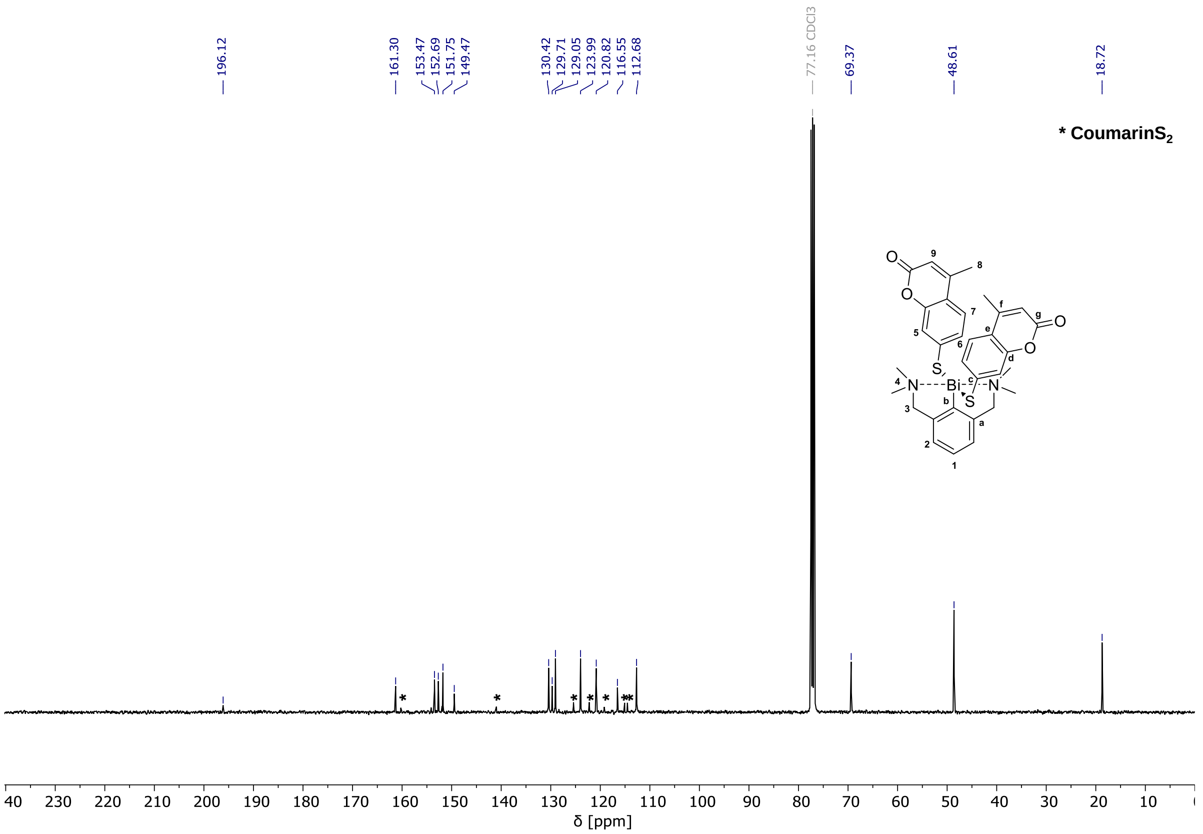

Figure S12. $^{13}C\{^{1}H\}$-NMR spectrum of complex **2** in $CDCl_3$.

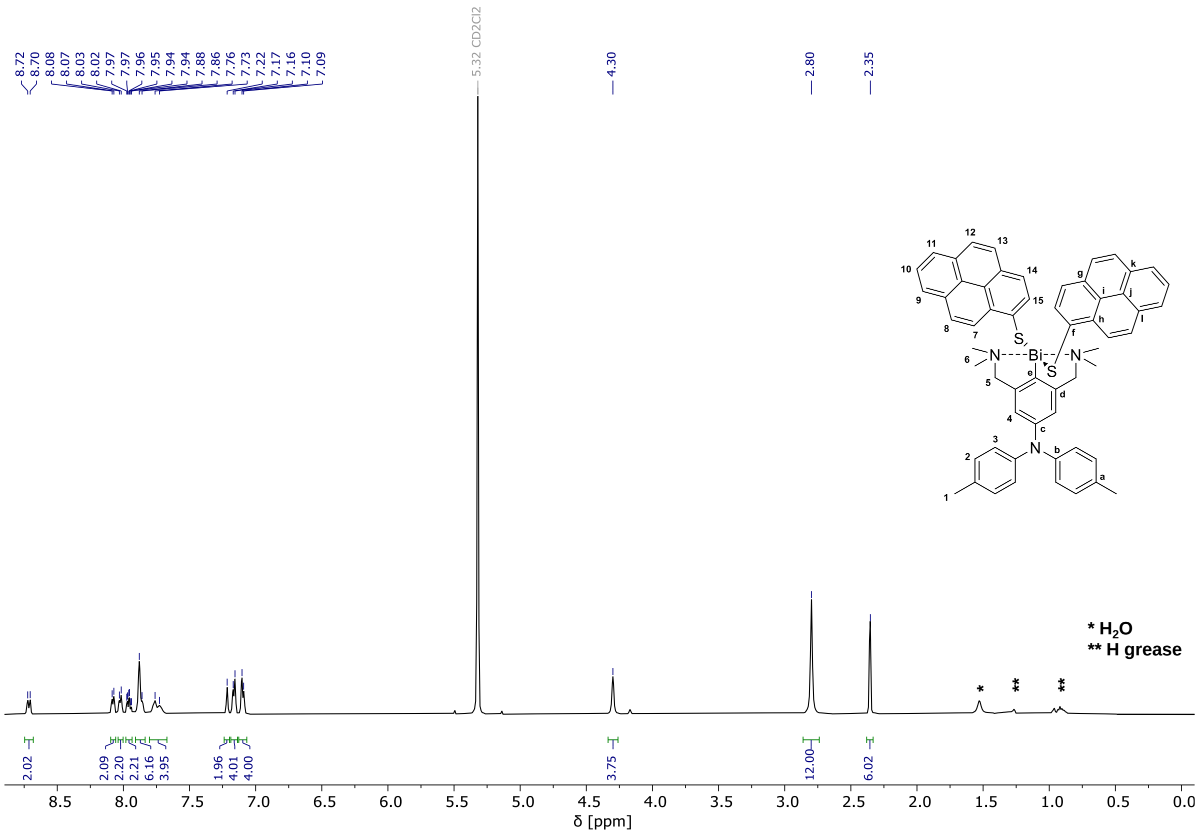

Figure S13. ^{1}H-NMR spectrum of complex **3** in CD_2Cl_2.

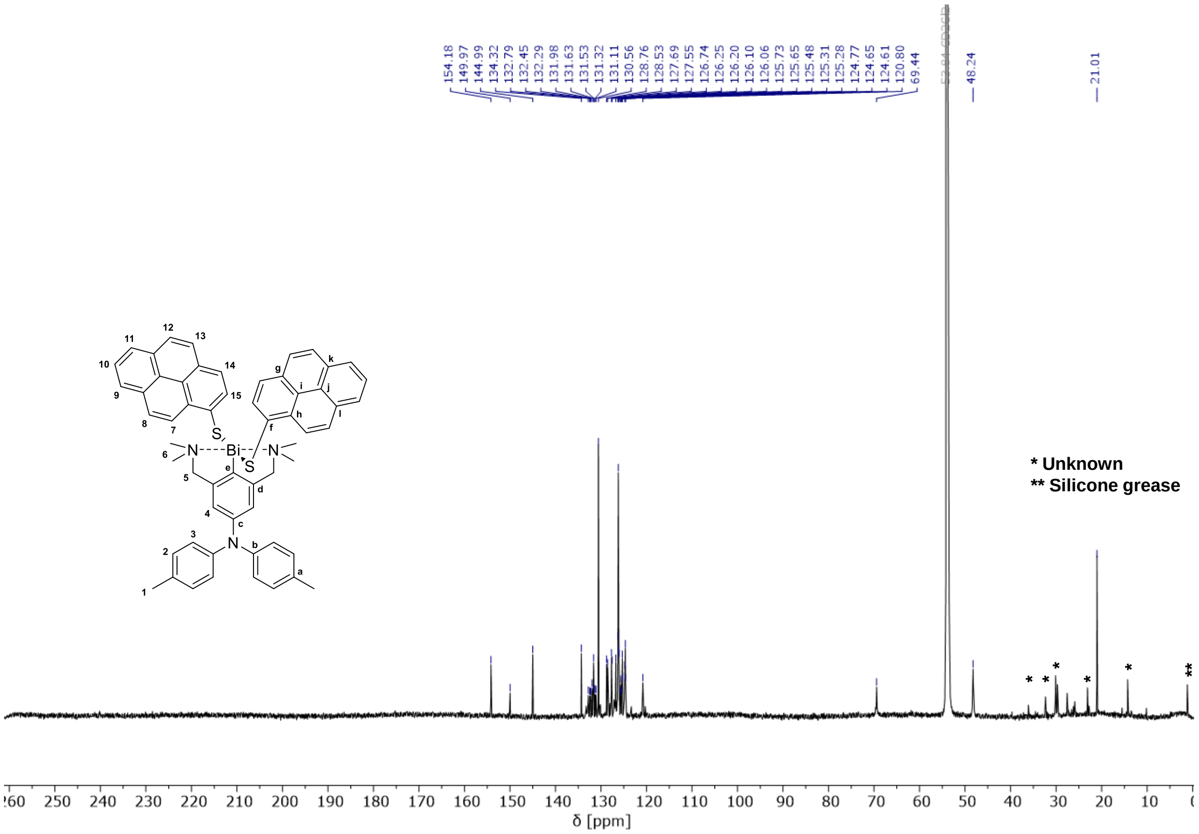

Figure S14. $^{13}C\{^{1}H\}$-NMR spectrum of complex **3** and of decomposition products formed during data acquisition.

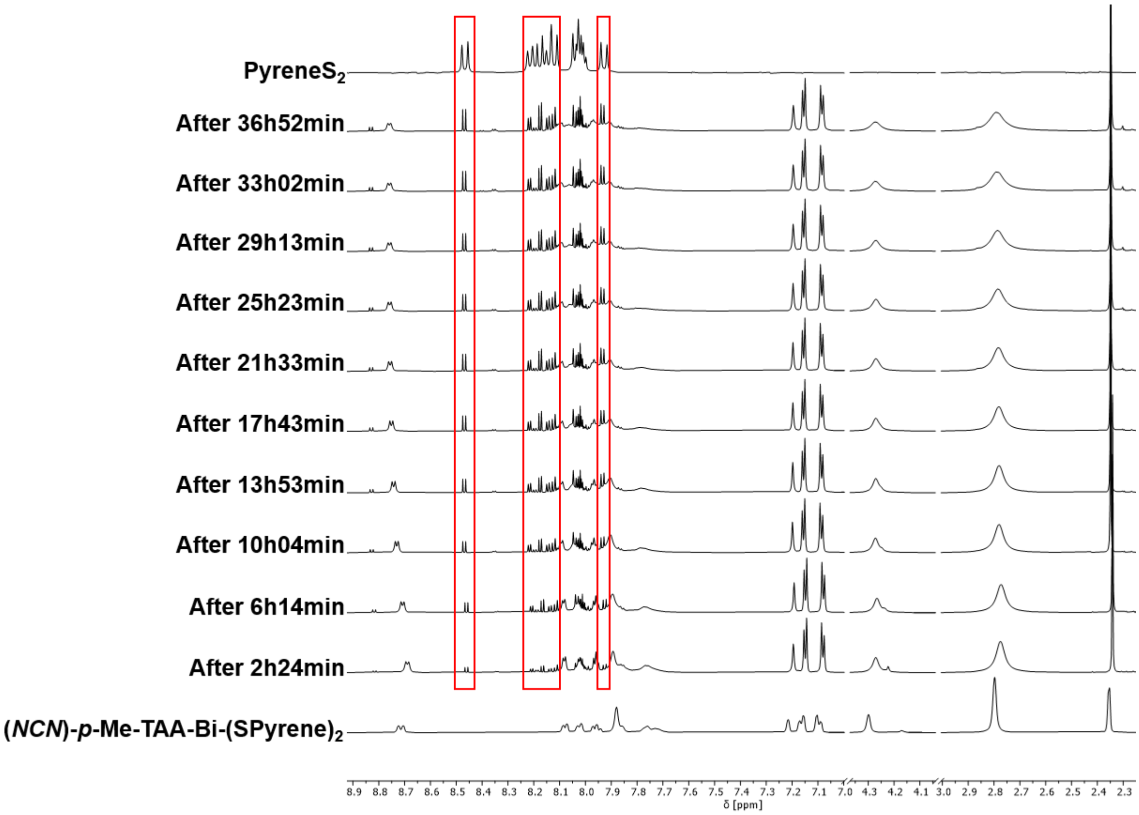

Figure S15. Photodecomposition of complex **3** monitored by ^{1}H-NMR spectroscopy in CD_2Cl_2 (~20 mM under ambient conditions).

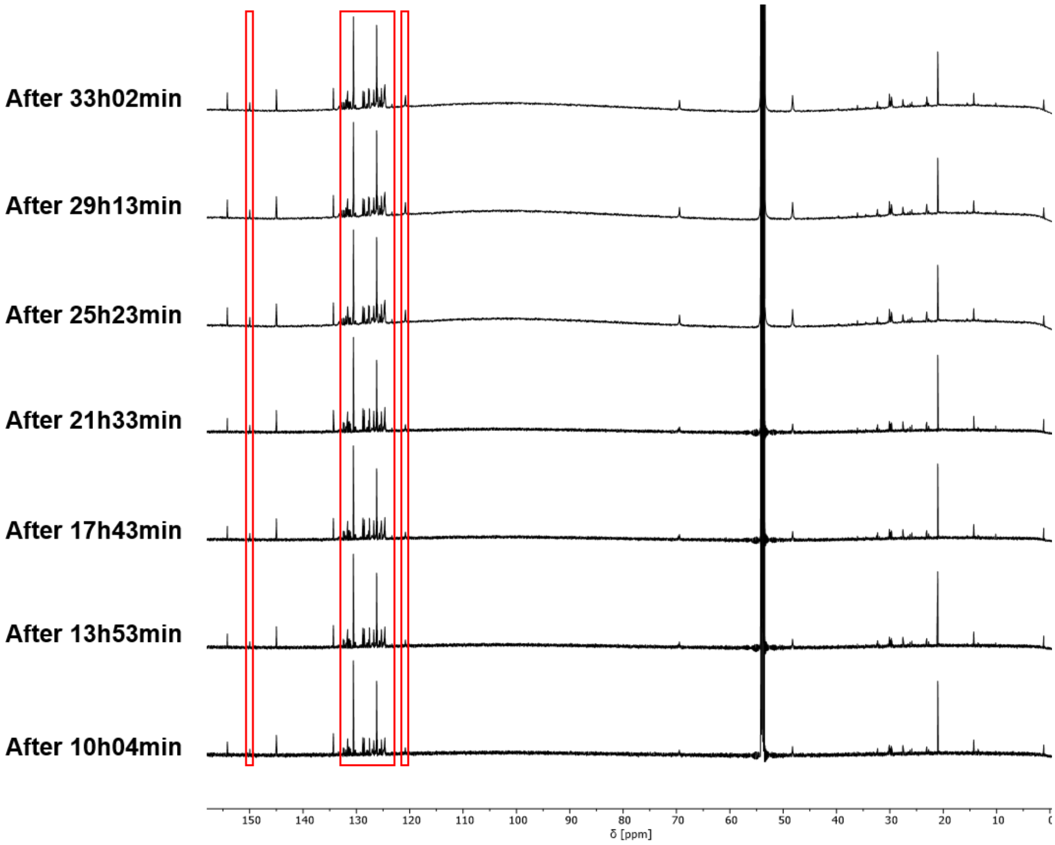

Figure S16. Photodecomposition of complex **3** monitored by ^{13}C{^{1}H}-NMR spectroscopy in CD_2Cl_2 (concentration *c* ~20 mM) under ambient conditions.

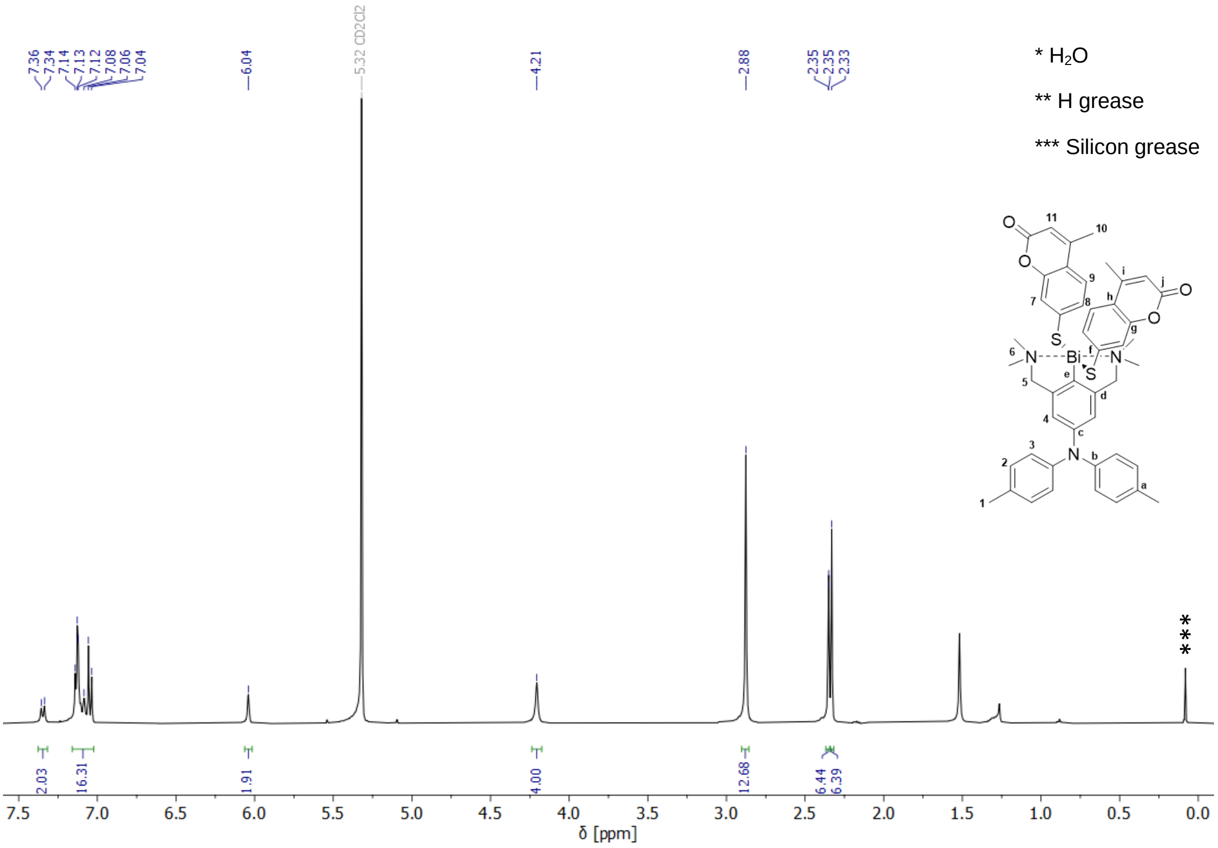

Figure S17. ^{1}H-NMR spectrum of complex **4** in CD_2Cl_2.

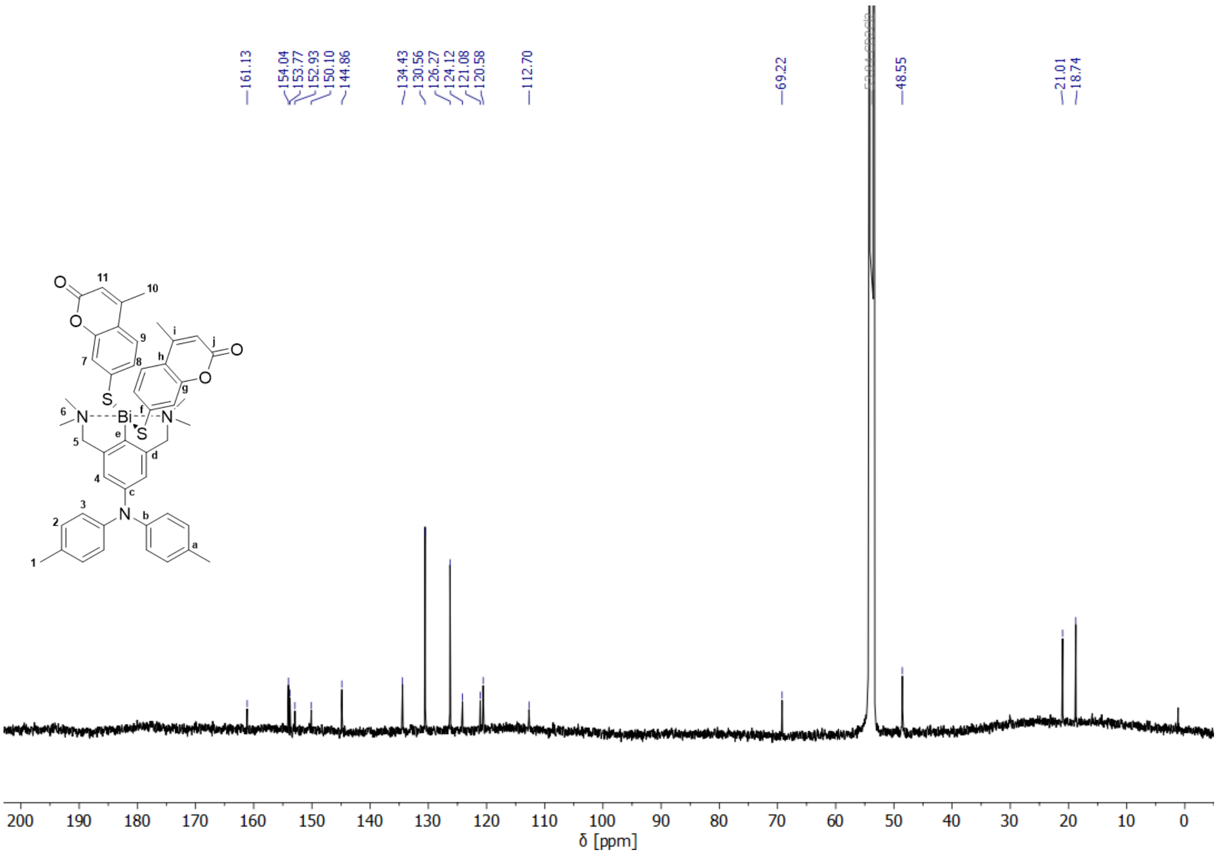

Figure S18. $^{13}C\{^{1}H\}$-NMR spectrum of complex **4** in CD_2Cl_2.

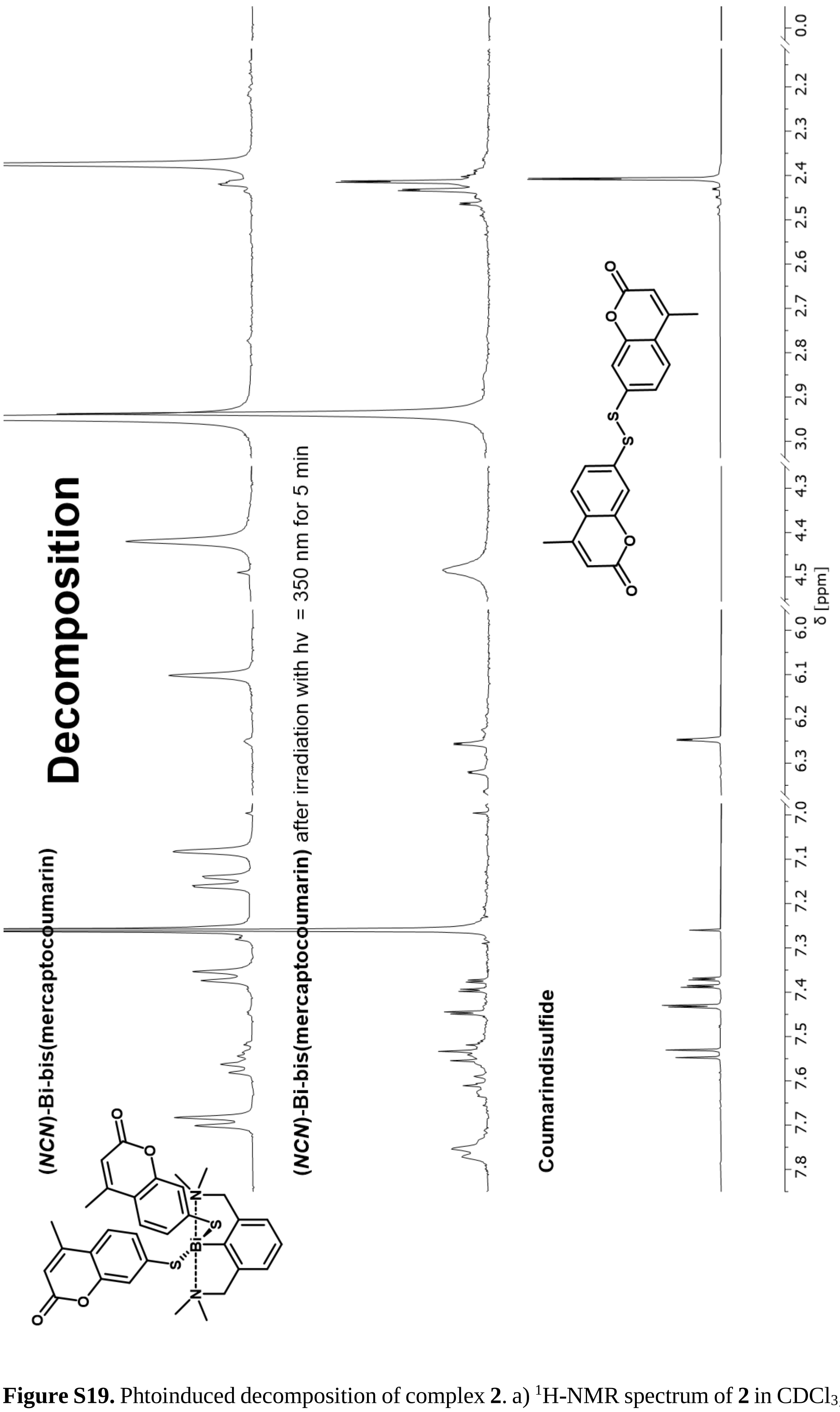

Figure S19. Phtoinduced decomposition of complex **2**. a) ^{1}H-NMR spectrum of **2** in $CDCl_3$; b) ^{1}H-NMR spectrum of **2** (concentration *c* ~10 mM) after irradiation at λ = 365 nm (2.4 W) for 5 minutes at room temperature in $CDCl_3$; c) ^{1}H-NMR spectrum of pure **CoumarinS$_2$** in $CDCl_3$.

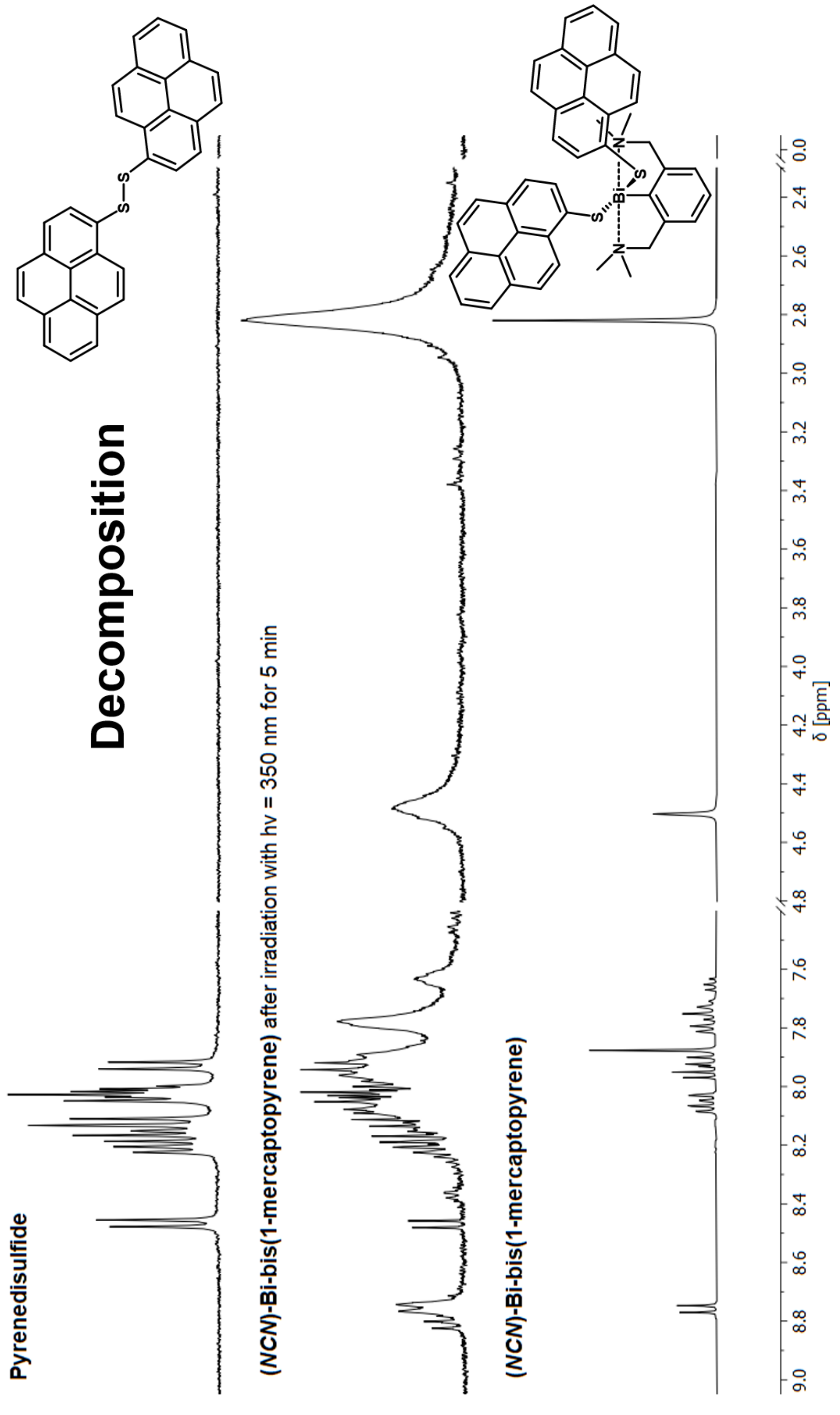

Figure S20. Photoinduced decomposition of complex **1**. a) ^{1}H-NMR spectrum of **PyreneS$_2$** in $CDCl_3$; b) ^{1}H-NMR spectrum of **1** (concentration *c* ~10 mM) after irradiation at λ = 365 nm (2.4 W) for 5 minutes at room temperature in $CDCl_3$; c) ^{1}H-NMR spectrum of **1** in $CDCl_3$.

ESI-MS Data

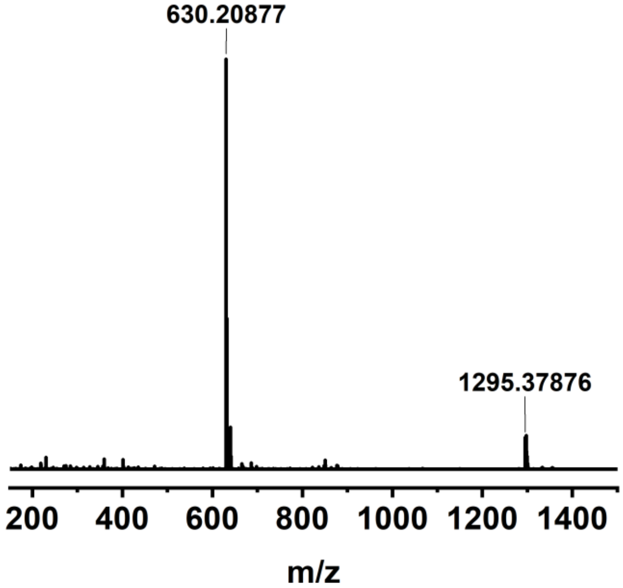

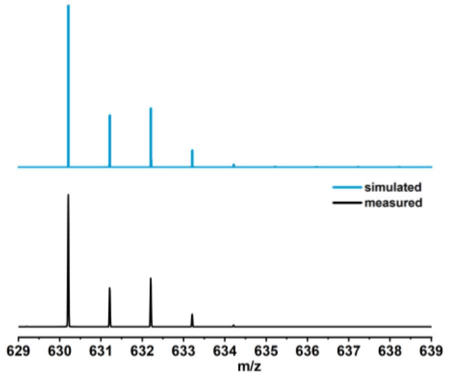

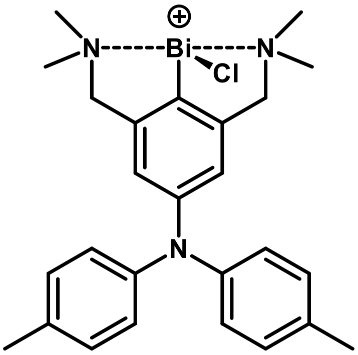

Chemical Formula: $C_{26}H_{32}BiClN_3^+$
Exact Mass: 630,2083

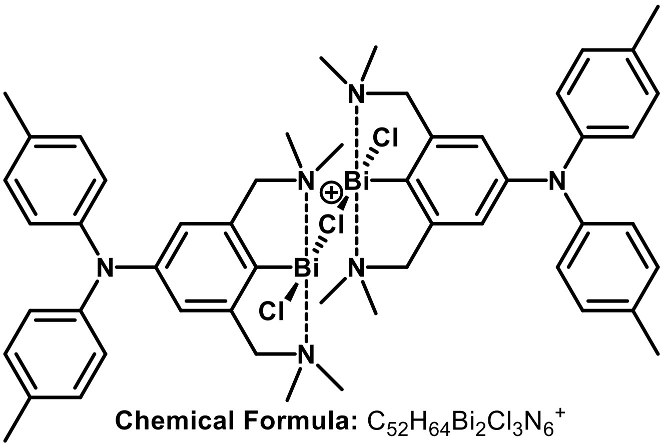

Chemical Formula: $C_{52}H_{64}Bi_2Cl_3N_6^+$
Exact Mass: 1295,3860

Figure S21. ESI-MS of complex **(*NCN*)*^DAA^*BiCl₂** in CH_2Cl_2 (experimental data in black, calculated data in blue).

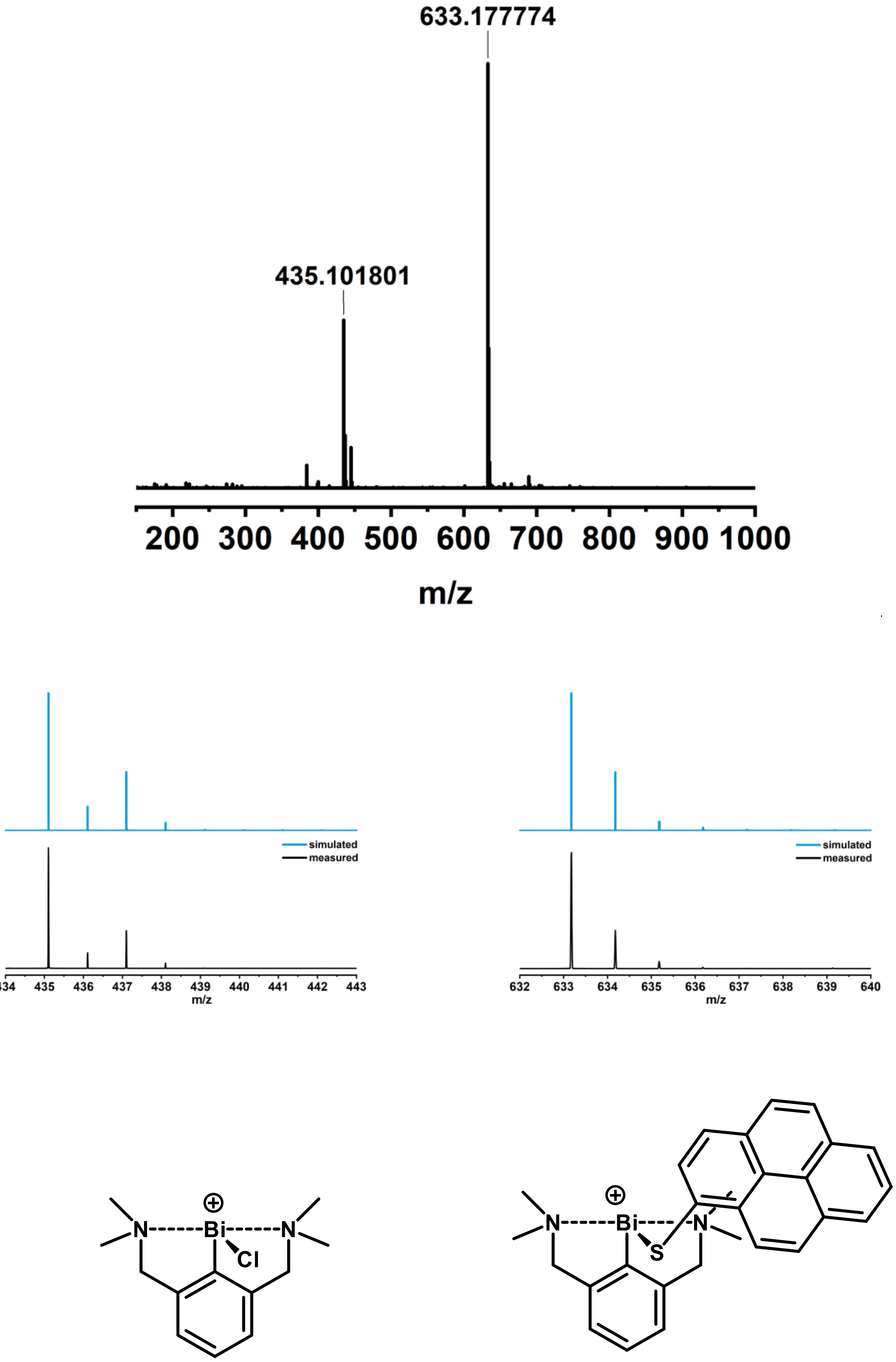

Chemical Formula: $C_{12}H_{19}BiClN_2^+$
Exact Mass: 435,1035

Chemical Formula: $C_{28}H_{28}BiN_2S^+$
Exact Mass: 633,1772

Figure S22. ESI-MS of complex **1** in CH_2Cl_2 (experimental data in black, calculated data in blue).

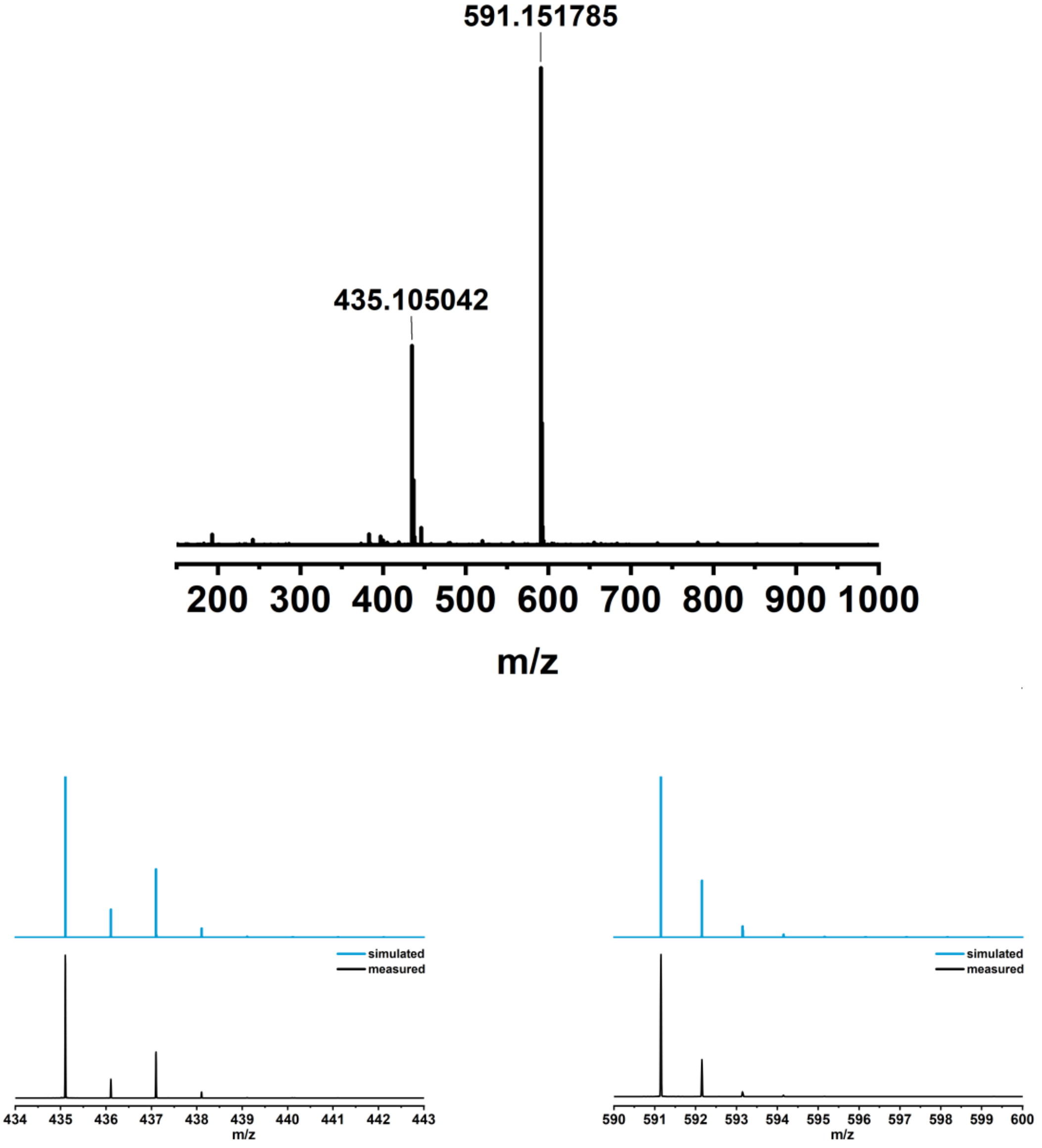

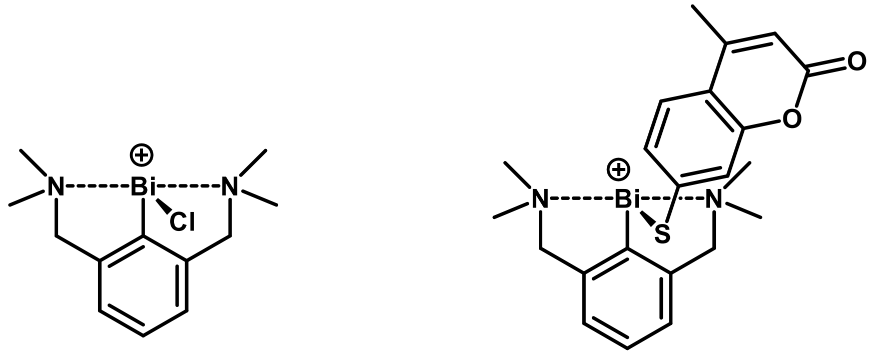

Chemical Formula: $C_{12}H_{19}BiClN_2^+$
Exact Mass: 435,1035

Chemical Formula: $C_{22}H_{26}BiN_2O_2S^+$
Exact Mass: 591,1513

Figure S23. ESI-MS of complex **2** in CH_2Cl_2 (experimental data in black, calculated data in blue).

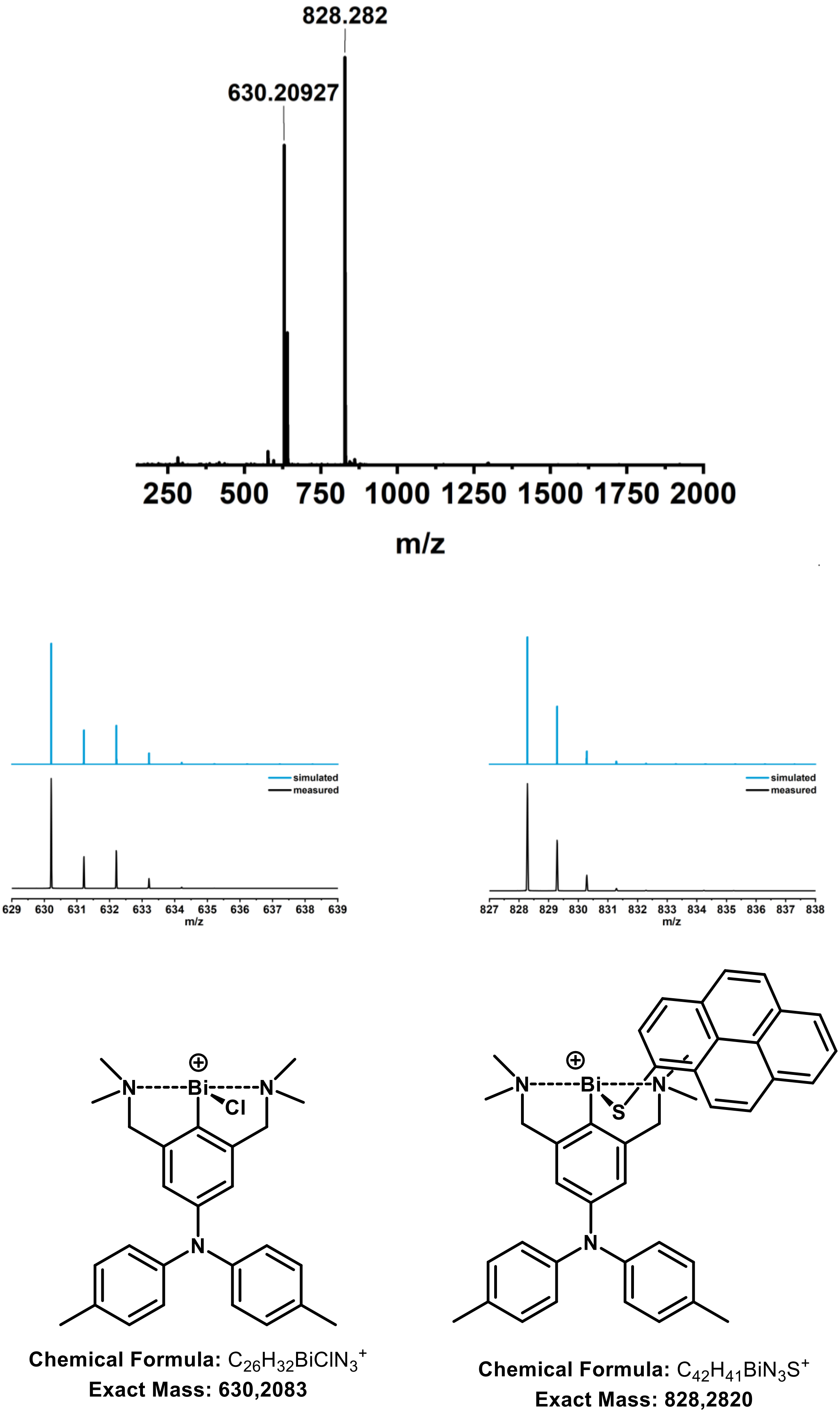

Figure S24. ESI-MS of complex **3** in CH_2Cl_2 (experimental data in black, calculated data in blue).

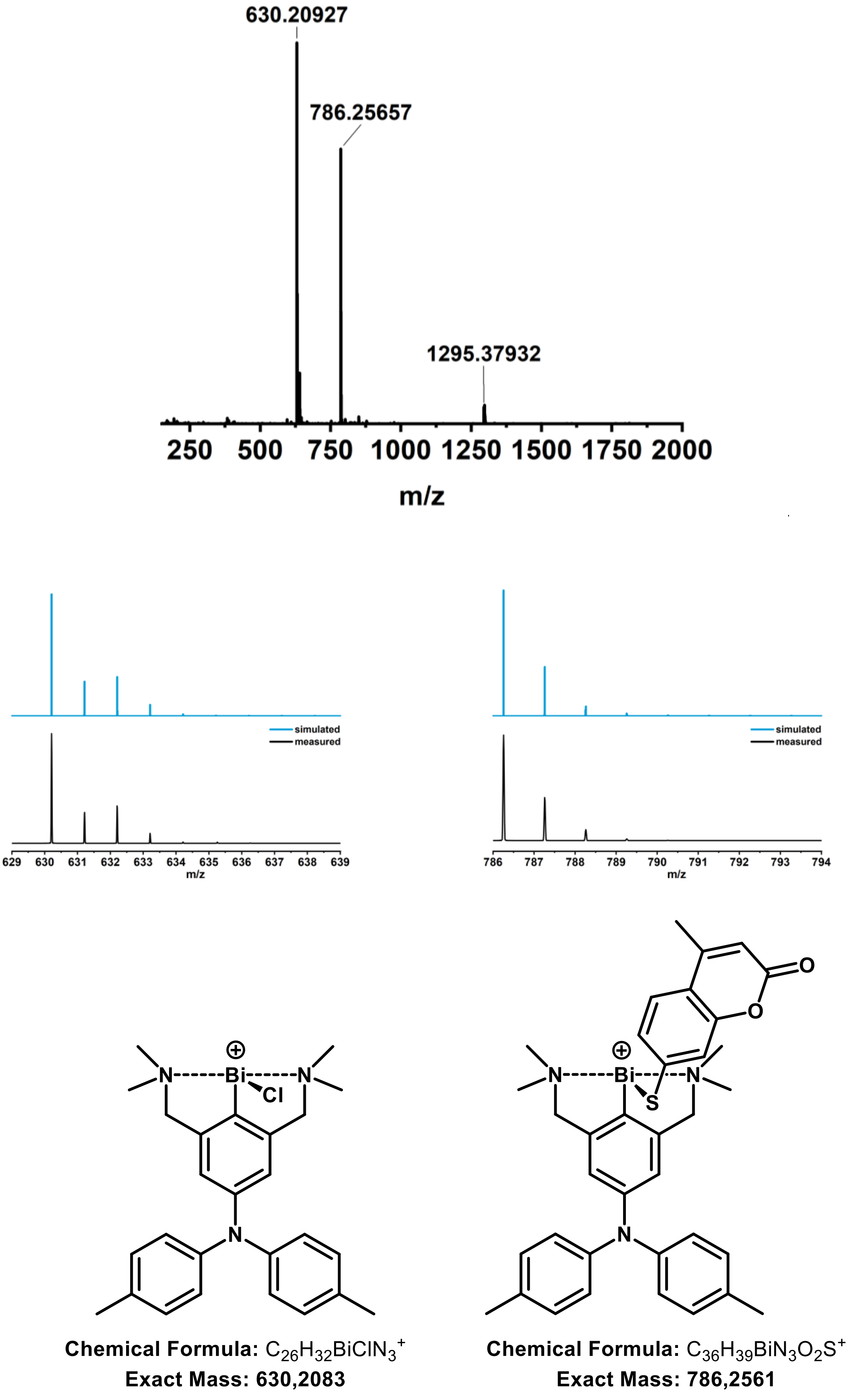

Figure S25. ESI-MS of complex **4** in CH_2Cl_2 (experimental data in black, calculated data in blue).

Single Crystal X-Ray Diffraction Data

Table S1. Crystal data and structure refinement for **(*NCN*)^{DAA}BiCl$_2$**.

Identification code	**(*NCN*)^{DAA}BiCl$_2$**
Empirical formula	$C_{26}H_{32}BiCl_2N_3$
Formula weight	666.450
Temperature/K	100.00
Crystal system	triclinic
Space group	$P\bar{1}$
a/Å	8.4745(3)
b/Å	10.9100(5)
c/Å	15.3256(6)
α/°	71.473(3)
β/°	85.314(3)
γ/°	87.171(3)
Volume/Å^3	1338.62(10)
Z	2
ρ_{calc}g/cm^3	1.653
μ/mm^{-1}	6.802
F(000)	646.3
Crystal size/mm^3	0.2 × 0.09 × 0.02
Radiation	MoKα (λ = 0.71073)
2Θ range for data collection/°	4.06 to 50.24
Index ranges	-10 ≤ h ≤ 11, -14 ≤ k ≤ 14, -19 ≤ l ≤ 19
Reflections collected	11397
Independent reflections	4753 [R_{int} = 0.0410, R_{sigma} = 0.0537]
Data/restraints/parameters	4753/0/296
Goodness-of-fit on F^2	1.031
Final R indexes [I>=2σ (I)]	R_1 = 0.0375, wR_2 = 0.0887
Final R indexes [all data]	R_1 = 0.0484, wR_2 = 0.0940
Largest diff. peak/hole / e Å^{-3}	2.16/-1.69

Table S2. Bond Lengths [Å] for ***(NCN)^{DAA}*BiCl$_2$**.

Atom	Atom	Length/Å	Atom	Atom	Length/Å
Bi1	Cl2	2.7037(17)	C5	C4	1.425(9)
Bi1	Cl1	2.7085(17)	C3	C4	1.400(9)
Bi1	N1	2.519(5)	C3	C2	1.385(9)
Bi1	N2	2.549(6)	C2	C7	1.502(9)
Bi1	C1	2.207(6)	C19	C18	1.391(9)
N1	C7	1.482(8)	C19	C13	1.411(9)
N1	C9	1.474(9)	C18	C16	1.395(10)
N1	C8	1.469(9)	C23	C22	1.399(10)
N2	C10	1.492(8)	C23	C25	1.395(10)
N2	C11	1.463(8)	C23	C24	1.500(9)
N2	C12	1.475(9)	C22	C21	1.399(9)
N3	C4	1.406(8)	C25	C26	1.391(9)
N3	C13	1.419(8)	C21	C20	1.392(9)
N3	C20	1.416(8)	C26	C20	1.396(9)
C6	C1	1.384(9)	C16	C15	1.390(10)
C6	C5	1.385(9)	C16	C17	1.494(9)
C6	C10	1.505(8)	C13	C14	1.407(9)
C1	C2	1.398(8)	C15	C14	1.392(9)
Bi1	Cl2	2.7037(17)	C5	C4	1.425(9)

Table S3. Bond Angles [°] for ***(NCN)**^{DAA}***BiCl$_2$**.

**Atom**	**Atom**	**Atom**	**Angle/°**	**Atom**	**Atom**	**Atom**	**Angle/°**
Cl1	Bi1	Cl2	176.25(5)	C4	C5	C6	119.5(6)
N1	Bi1	Cl2	82.41(14)	C6	C10	N2	111.1(5)
N1	Bi1	Cl1	96.44(14)	C2	C3	C4	121.6(6)
N2	Bi1	Cl2	96.05(14)	C5	C4	N3	120.1(6)
N2	Bi1	Cl1	82.83(14)	C3	C4	N3	121.2(6)
N2	Bi1	N1	144.97(18)	C3	C4	C5	118.7(6)
C1	Bi1	Cl2	87.71(17)	C3	C2	C1	118.5(6)
C1	Bi1	Cl1	88.55(17)	C7	C2	C1	118.4(5)
C1	Bi1	N1	72.6(2)	C7	C2	C3	123.0(5)
C1	Bi1	N2	72.3(2)	C13	C19	C18	120.0(6)
C7	N1	Bi1	105.2(4)	C2	C7	N1	112.6(5)
C9	N1	Bi1	107.5(4)	C16	C18	C19	121.8(6)
C9	N1	C7	109.9(5)	C25	C23	C22	118.0(6)
C8	N1	Bi1	113.3(4)	C24	C23	C22	119.9(6)
C8	N1	C7	110.6(5)	C24	C23	C25	122.1(6)
C8	N1	C9	110.2(6)	C21	C22	C23	120.4(6)
C10	N2	Bi1	105.0(4)	C26	C25	C23	121.6(6)
C11	N2	Bi1	113.2(4)	C20	C21	C22	121.2(6)
C11	N2	C10	110.6(6)	C20	C26	C25	120.3(6)
C12	N2	Bi1	108.3(4)	C15	C16	C18	117.7(6)
C12	N2	C10	109.9(5)	C17	C16	C18	121.3(7)
C12	N2	C11	109.7(6)	C17	C16	C15	121.0(7)
C13	N3	C4	120.5(5)	C19	C13	N3	119.2(6)
C20	N3	C4	121.3(5)	C14	C13	N3	122.1(6)
C20	N3	C13	117.5(5)	C14	C13	C19	118.6(6)
C5	C6	C1	120.3(6)	C14	C15	C16	122.1(6)
C10	C6	C1	119.3(5)	C15	C14	C13	119.9(6)
C10	C6	C5	120.4(6)	C21	C20	N3	120.3(6)
C6	C1	Bi1	119.6(4)	C26	C20	N3	121.2(6)
C2	C1	Bi1	119.0(5)	C26	C20	C21	118.5(6)
Cl1	Bi1	Cl2	176.25(5)	C4	C5	C6	119.5(6)
C2	C1	C6	121.4(6)				

Table S4. Torsion Angles [°] for ***(NCN)***^{DAA}**BiCl₂**.

A	B	C	D	Angle/°	A	B	C	D	Angle/°
Bi1	N1	C7	C2	36.5(4)	N3	C20	C26	C25	179.1(6)
Bi1	N2	C10	C6	37.5(4)	C6	C1	C2	C3	1.2(8)
Bi1	C1	C6	C5	-176.7(5)	C6	C1	C2	C7	-176.5(6)
Bi1	C1	C6	C10	5.7(6)	C6	C5	C4	C3	1.3(7)
Bi1	C1	C2	C3	177.4(5)	C1	C2	C3	C4	-0.6(7)
Bi1	C1	C2	C7	-0.3(6)	C5	C4	C3	C2	-0.6(7)
N1	C7	C2	C1	-27.7(6)	C19	C18	C16	C15	-0.8(8)
N1	C7	C2	C3	154.6(5)	C19	C18	C16	C17	179.1(7)
N2	C10	C6	C1	-32.5(7)	C19	C13	C14	C15	0.9(7)
N2	C10	C6	C5	150.0(5)	C18	C16	C15	C14	1.5(8)
N3	C4	C5	C6	-178.8(6)	C23	C22	C21	C20	-0.6(8)
N3	C4	C3	C2	179.5(6)	C23	C25	C26	C20	-0.2(8)
N3	C13	C19	C18	-176.9(6)	C22	C21	C20	C26	0.1(7)
N3	C13	C14	C15	177.5(6)	C25	C26	C20	C21	0.3(7)
N3	C20	C21	C22	-178.7(6)	C16	C15	C14	C13	-1.6(8)

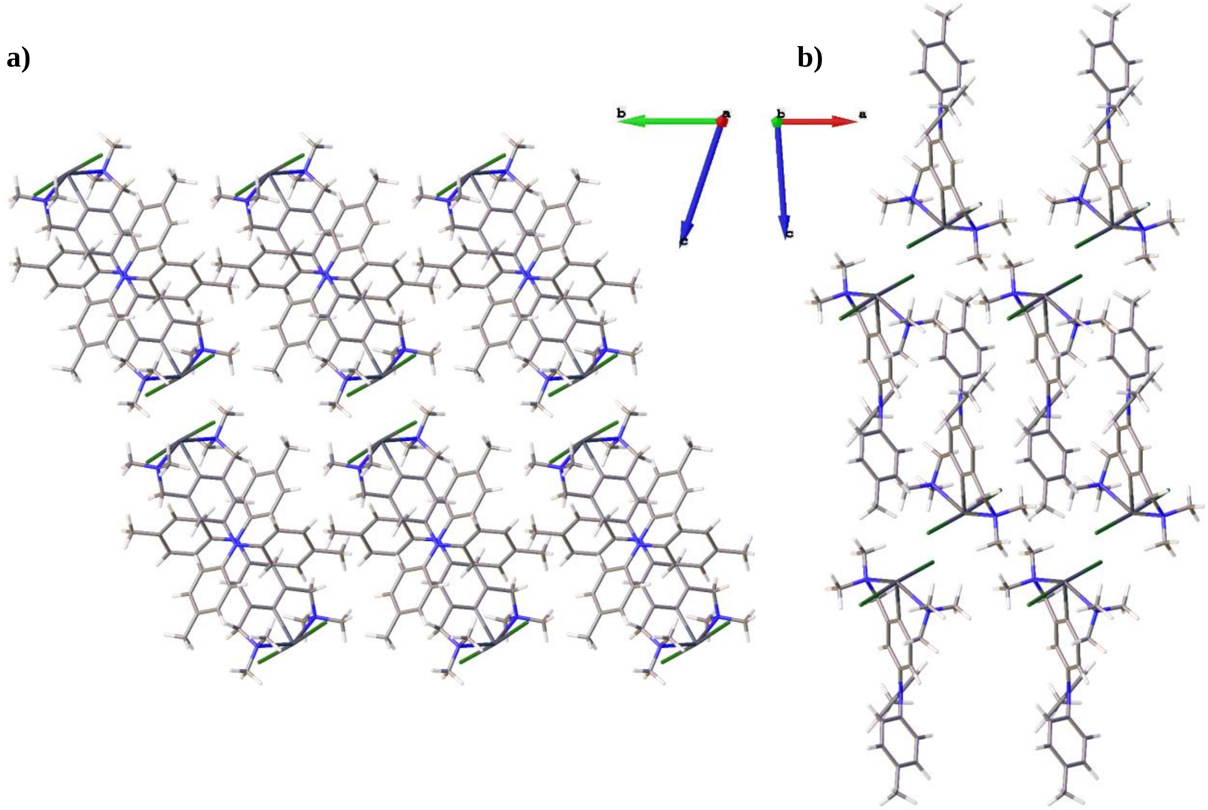

Figure S26. Packing diagrams of complex **(*NCN*)^{DAA}BiCl$_2$** viewed along a) the *a*-axis, and b) the *b*-axis of the unit cell.

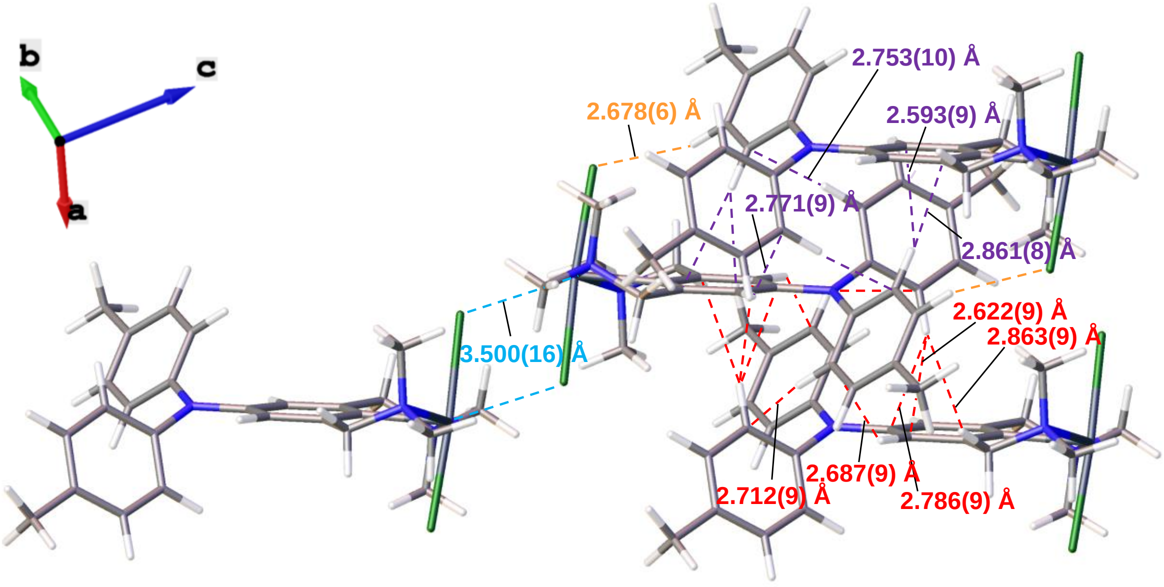

Figure S27. Molecular packing and relevant interatomic contacts of **(*NCN*)^{DAA}BiCl$_2$**.

Table S5. Crystal data and structure refinement for complex **2**.

Identification code	**2**
Empirical formula	$C_{32}H_{33}BiN_2O_4S_2$
Formula weight	782.70
Temperature/K	100
Crystal system	monoclinic
Space group	$P2_1$
a/Å	10.4962(2)
b/Å	29.1629(4)
c/Å	10.0437(2)
α/°	90
β/°	90.058(2)
γ/°	90
Volume/Å^3	3074.37(9)
Z	4
ρ_{calc}g/cm^3	1.691
μ/mm^{-1}	5.910
F(000)	1544.0
Crystal size/mm^3	0.3 × 0.233 × 0.2
Radiation	Mo Kα (λ = 0.71073)
2Θ range for data collection/°	3.88 to 55.26
Index ranges	$-13 \leq h \leq 13$, $-37 \leq k \leq 36$, $-13 \leq l \leq 13$
Reflections collected	35649
Independent reflections	34072 [R_{int} = 0.0210, R_{sigma} = 0.0233]
Data/restraints/parameters	34072/1/752
Goodness-of-fit on F^2	1.220
Final *R* indexes [$I \geq 2\sigma(I)$]	R_1 = 0.0315, wR_2 = 0.0614
Final *R* indexes [all data]	R_1 = 0.0422, wR_2 = 0.0792
Largest diff. peak/hole / e Å^{-3}	0.99/-1.56
Flack parameter	-0.031(3)

Table S6. Bond Lengths [Å] for complex **2**.

Atom	Atom	Length/Å	Atom	Atom	Length/Å
Bi1A	S2A	2.734(5)	C14	C13	1.369(19)
Bi1A	S1A	2.771(4)	C14	C15	1.38(2)
Bi1A	C1A	2.192(12)	C25A	C24A	1.37(2)
Bi1A	N2A	2.527(12)	C21	C22	1.393(19)
Bi1A	N1A	2.526(14)	C22	C13	1.40(2)
Bi1	S2	2.745(4)	C23	C24	1.40(2)
Bi1	S1	2.762(4)	C23	C32	1.40(2)
Bi1	N1	2.527(13)	C16A	O2A	1.229(18)
Bi1	N2	2.535(13)	C16A	C17A	1.43(2)
Bi1	C1	2.190(11)	O1	C16	1.390(16)
S2	C23	1.748(14)	O1	C15	1.378(16)
S2A	C23A	1.776(15)	C27	C26	1.46(3)
S1	C13	1.770(14)	C21A	C22A	1.38(2)
S1A	C13A	1.746(14)	C16	C17	1.43(2)
C12	N2	1.45(2)	C16	O2	1.223(17)
N1	C8	1.47(2)	C17	C18	1.35(2)
N1	C7	1.47(2)	C23A	C24A	1.35(2)
N1	C9	1.47(2)	C23A	C32A	1.42(2)
C28	C30	1.45(2)	O4A	C26A	1.23(2)
C28	C27	1.30(3)	C18	C19	1.50(2)
C28	C29	1.52(3)	C2	C3	1.386(19)
C20A	C21A	1.41(2)	C2	C1	1.392(19)
C20A	C15A	1.38(2)	C2	C7	1.50(2)
C20A	C18A	1.449(19)	C14A	C15A	1.384(19)
C30	C25	1.39(2)	C4	C3	1.37(2)
C30	C31	1.40(2)	C4	C5	1.38(2)
C30A	C28A	1.43(2)	C18A	C17A	1.34(2)
C30A	C31A	1.38(2)	C18A	C19A	1.50(2)
C30A	C25A	1.40(2)	C26	O4	1.24(2)
C25	O3	1.38(2)	C1	C6	1.362(19)
C25	C24	1.39(2)	C1A	C6A	1.358(19)
N2	C11	1.475(18)	C1A	C2A	1.374(19)
N2	C10	1.491(17)	C10	C6	1.529(19)
C27A	C28A	1.37(3)	C32	C31	1.37(2)
C27A	C26A	1.41(3)	C11A	N2A	1.463(17)
O3A	C25A	1.40(2)	C5	C6	1.402(18)
O3A	C26A	1.37(2)	N2A	C12A	1.499(19)
C13A	C22A	1.40(2)	N2A	C10A	1.473(18)
C13A	C14A	1.390(19)	C5A	C6A	1.424(18)
O1A	C16A	1.394(17)	C5A	C4A	1.35(2)
O1A	C15A	1.384(17)	C7A	N1A	1.48(2)
C20	C21	1.386(19)	C7A	C2A	1.51(2)

Atom	Atom	Length/Å	Atom	Atom	Length/Å
C20	C18	1.43(2)	N1A	C8A	1.46(2)
C20	C15	1.39(2)	N1A	C9A	1.46(2)
C28A	C29A	1.49(3)	C6A	C10A	1.527(19)
O3	C26	1.37(2)	C4A	C3A	1.38(2)
C31A	C32A	1.40(2)	C2A	C3A	1.40(2)

Table S7. Bond Angles [°] for complex **2**.

Atom	Atom	Atom	Angle/°	Atom	Atom	Atom	Angle/°
S2A	Bi1A	S1A	169.85(13)	C14	C13	C22	118.6(13)
C1A	Bi1A	S2A	82.2(4)	C22	C13	S1	121.7(11)
C1A	Bi1A	S1A	87.7(4)	C15	O1	C16	120.2(12)
C1A	Bi1A	N2A	72.4(5)	C28	C27	C26	123.7(18)
C1A	Bi1A	N1A	72.7(5)	C22A	C21A	C20A	120.2(14)
N2A	Bi1A	S2A	92.0(3)	O1	C16	C17	117.1(12)
N2A	Bi1A	S1A	84.0(3)	O2	C16	O1	115.9(13)
N1A	Bi1A	S2A	84.3(4)	O2	C16	C17	127.0(13)
N1A	Bi1A	S1A	93.7(4)	C21A	C22A	C13A	122.4(14)
N1A	Bi1A	N2A	145.1(4)	C18	C17	C16	124.0(14)
S2	Bi1	S1	170.85(13)	C24A	C23A	S2A	120.1(12)
N1	Bi1	S2	84.4(3)	C24A	C23A	C32A	120.3(15)
N1	Bi1	S1	92.7(3)	C32A	C23A	S2A	119.5(13)
N1	Bi1	N2	144.7(4)	C20	C18	C19	121.0(13)
N2	Bi1	S2	93.1(3)	C17	C18	C20	117.5(14)
N2	Bi1	S1	84.2(3)	C17	C18	C19	121.5(14)
C1	Bi1	S2	82.6(4)	C3	C2	C1	118.9(14)
C1	Bi1	S1	88.3(4)	C3	C2	C7	123.1(13)
C1	Bi1	N1	72.0(5)	C1	C2	C7	118.0(13)
C1	Bi1	N2	72.7(5)	C15A	C14A	C13A	120.7(14)
C23	S2	Bi1	100.3(5)	C20A	C15A	O1A	121.6(13)
C23A	S2A	Bi1A	100.9(5)	C20A	C15A	C14A	122.9(14)
C13	S1	Bi1	96.8(5)	O1A	C15A	C14A	115.5(13)
C13A	S1A	Bi1A	97.0(5)	C25	C24	C23	119.1(15)
C8	N1	Bi1	112.3(10)	C3	C4	C5	120.9(14)
C8	N1	C9	110.7(14)	C14	C15	C20	122.6(14)
C7	N1	Bi1	103.8(9)	O1	C15	C20	121.9(13)
C7	N1	C8	110.5(13)	O1	C15	C14	115.6(13)
C7	N1	C9	111.2(13)	C20A	C18A	C19A	120.6(13)
C9	N1	Bi1	108.2(10)	C17A	C18A	C20A	118.4(14)
C30	C28	C29	118.8(18)	C17A	C18A	C19A	120.9(13)
C27	C28	C30	118.8(19)	O3A	C26A	C27A	118.4(19)
C27	C28	C29	122.4(18)	O4A	C26A	C27A	126.7(19)
C21A	C20A	C18A	123.9(14)	O4A	C26A	O3A	115(2)
C15A	C20A	C21A	117.0(13)	C23A	C24A	C25A	120.3(16)

Atom	Atom	Atom	Angle/°	Atom	Atom	Atom	Angle/°
C15A	C20A	C18A	119.0(13)	C4	C3	C2	120.1(15)
C25	C30	C28	117.5(16)	O3	C26	C27	117.4(17)
C25	C30	C31	117.5(15)	O4	C26	O3	115(2)
C31	C30	C28	125.0(16)	O4	C26	C27	127.5(18)
C31A	C30A	C28A	126.0(18)	C18A	C17A	C16A	123.0(14)
C31A	C30A	C25A	115.4(16)	C2	C1	Bi1	119.0(10)
C25A	C30A	C28A	118.5(17)	C6	C1	Bi1	119.8(10)
O3	C25	C30	122.8(14)	C6	C1	C2	121.2(12)
O3	C25	C24	114.5(15)	C6A	C1A	Bi1A	119.2(9)
C24	C25	C30	122.7(17)	C6A	C1A	C2A	121.7(13)
C12	N2	Bi1	110.0(8)	C2A	C1A	Bi1A	119.1(10)
C12	N2	C11	110.5(12)	N2	C10	C6	110.9(11)
C12	N2	C10	111.9(12)	C31	C32	C23	122.6(16)
C11	N2	Bi1	111.1(9)	C4	C5	C6	119.2(14)
C11	N2	C10	109.1(11)	C11A	N2A	Bi1A	111.7(9)
C10	N2	Bi1	104.1(8)	C11A	N2A	C12A	108.9(12)
C28A	C27A	C26A	123.5(18)	C11A	N2A	C10A	110.8(12)
C26A	O3A	C25A	119.9(15)	C12A	N2A	Bi1A	110.4(9)
C22A	C13A	S1A	123.0(11)	C10A	N2A	Bi1A	104.4(8)
C14A	C13A	S1A	120.2(11)	C10A	N2A	C12A	110.6(12)
C14A	C13A	C22A	116.8(13)	C4A	C5A	C6A	119.4(15)
C15A	O1A	C16A	120.1(12)	N1A	C7A	C2A	111.4(12)
C21	C20	C18	124.4(13)	C1	C6	C10	118.9(12)
C21	C20	C15	116.3(13)	C1	C6	C5	119.7(13)
C15	C20	C18	119.2(13)	C5	C6	C10	121.4(13)
C30A	C28A	C29A	120(2)	C31A	C32A	C23A	117.4(17)
C27A	C28A	C30A	118.1(19)	C7A	N1A	Bi1A	104.1(9)
C27A	C28A	C29A	122.1(19)	C8A	N1A	Bi1A	111.4(10)
C26	O3	C25	119.6(15)	C8A	N1A	C7A	108.5(13)
C30A	C31A	C32A	123.3(17)	C9A	N1A	Bi1A	111.6(11)
C13	C14	C15	120.5(13)	C9A	N1A	C7A	109.8(14)
O3A	C25A	C30A	121.6(15)	C9A	N1A	C8A	111.2(15)
C24A	C25A	C30A	123.1(16)	C1A	C6A	C5A	118.7(14)
C24A	C25A	O3A	115.2(16)	C1A	C6A	C10A	119.2(11)
C20	C21	C22	121.9(13)	C5A	C6A	C10A	122.0(13)
C21	C22	C13	120.1(13)	C32	C31	C30	120.3(16)
C24	C23	S2	119.1(12)	N1	C7	C2	111.4(12)
C24	C23	C32	117.8(13)	C5A	C4A	C3A	121.7(15)
C32	C23	S2	123.0(12)	C1A	C2A	C7A	118.9(13)
O1A	C16A	C17A	117.9(13)	C1A	C2A	C3A	119.6(15)
O2A	C16A	O1A	114.0(14)	C3A	C2A	C7A	121.4(14)
O2A	C16A	C17A	128.1(14)	C4A	C3A	C2A	118.8(15)
C14	C13	S1	119.7(11)	N2A	C10A	C6A	110.4(11)

Table S8. Torsion Angles [°] for complex **2**.

**A**	**B**	**C**	**D**	**Angle/°**	**A**	**B**	**C**	**D**	**Angle/°**
Bi1A	S2A	C23A	C24A	-127.1(11)	C21A	C20A	C18A	C17A	-178.6(14)
Bi1A	S2A	C23A	C32A	55.9(12)	C21A	C20A	C18A	C19A	6(2)
Bi1A	S1A	C13A	C22A	48.3(12)	C16	O1	C15	C20	-0.7(19)
Bi1A	S1A	C13A	C14A	-131.3(10)	C16	O1	C15	C14	179.4(12)
Bi1A	C1A	C6A	C5A	179.6(10)	C16	C17	C18	C20	-3(2)
Bi1A	C1A	C6A	C10A	-2.0(17)	C16	C17	C18	C19	177.4(14)
Bi1A	C1A	C2A	C7A	-2.7(19)	C22A	C13A	C14A	C15A	-2.3(19)
Bi1A	C1A	C2A	C3A	-178.5(11)	C18	C20	C21	C22	-179.3(14)
Bi1A	N2A	C10A	C6A	-39.1(12)	C18	C20	C15	C14	179.0(13)
Bi1	S2	C23	C24	-124.2(11)	C18	C20	C15	O1	-1(2)
Bi1	S2	C23	C32	56.1(13)	C2	C1	C6	C10	177.3(13)
Bi1	S1	C13	C14	-130.5(10)	C2	C1	C6	C5	-1(2)
Bi1	S1	C13	C22	48.5(12)	C14A	C13A	C22A	C21A	2(2)
Bi1	N1	C7	C2	-41.3(14)	C15A	C20A	C21A	C22A	1(2)
Bi1	N2	C10	C6	-38.4(12)	C15A	C20A	C18A	C17A	-1(2)
Bi1	C1	C6	C10	-3.1(17)	C15A	C20A	C18A	C19A	-177.1(13)
Bi1	C1	C6	C5	178.9(10)	C15A	O1A	C16A	O2A	178.7(13)
S2	C23	C24	C25	-176.9(11)	C15A	O1A	C16A	C17A	0(2)
S2	C23	C32	C31	178.2(12)	O2	C16	C17	C18	-178.3(16)
S2A	C23A	C24A	C25A	-176.9(11)	C24	C25	O3	C26	-177.2(15)
S2A	C23A	C32A	C31A	178.5(12)	C24	C23	C32	C31	-2(2)
S1A	C13A	C22A	C21A	-177.6(11)	C4	C5	C6	C1	1(2)
S1A	C13A	C14A	C15A	177.4(11)	C4	C5	C6	C10	-177.2(13)
C12	N2	C10	C6	-157.1(12)	C15	C20	C21	C22	-0.5(19)
C28	C30	C25	O3	1(2)	C15	C20	C18	C17	3(2)
C28	C30	C25	C24	-178.8(14)	C15	C20	C18	C19	-177.8(13)
C28	C30	C31	C32	-179.9(15)	C15	C14	C13	S1	177.4(11)
C28	C27	C26	O3	2(3)	C15	C14	C13	C22	-1.6(19)
C28	C27	C26	O4	-178.4(19)	C15	O1	C16	C17	0.4(19)
C20A	C21A	C22A	C13A	-1(2)	C15	O1	C16	O2	-179.6(13)
C20A	C18A	C17A	C16A	0(2)	C18A	C20A	C21A	C22A	178.1(14)
C30	C28	C27	C26	2(3)	C18A	C20A	C15A	O1A	3(2)
C30	C25	O3	C26	3(2)	C18A	C20A	C15A	C14A	-178.5(13)
C30	C25	C24	C23	-2(2)	O2A	C16A	C17A	C18A	-177.4(16)
C30A	C31A	C32A	C23A	-1(2)	C26A	C27A	C28A	C30A	1(2)
C30A	C25A	C24A	C23A	-3(2)	C26A	C27A	C28A	C29A	-177.9(18)
C25	C30	C31	C32	1(2)	C26A	O3A	C25A	C30A	3(2)
C25	O3	C26	C27	-4(2)	C26A	O3A	C25A	C24A	-178.9(14)
C25	O3	C26	O4	176.4(15)	C24A	C23A	C32A	C31A	2(2)
N2	C10	C6	C1	31.8(17)	C3	C2	C1	Bi1	-179.9(10)
N2	C10	C6	C5	-150.3(12)	C3	C2	C1	C6	0(2)
O3A	C25A	C24A	C23A	179.5(13)	C3	C2	C7	N1	-148.6(14)

A	B	C	D	Angle/°	A	B	C	D	Angle/°
C13A	C14A	C15A	C20A	2(2)	C3	C4	C5	C6	0(2)
C13A	C14A	C15A	O1A	-179.1(12)	C1	C2	C3	C4	1(2)
O1A	C16A	C17A	C18A	1(2)	C1	C2	C7	N1	32(2)
C20	C21	C22	C13	0(2)	C1A	C6A	C10A	N2A	31.6(17)
C28A	C30A	C31A	C32A	-178.8(15)	C1A	C2A	C3A	C4A	-3(2)
C28A	C30A	C25A	O3A	-2(2)	C32	C23	C24	C25	3(2)
C28A	C30A	C25A	C24A	-179.3(15)	C11A	N2A	C10A	C6A	81.3(14)
C28A	C27A	C26A	O3A	0(3)	C5	C4	C3	C2	-1(2)
C28A	C27A	C26A	O4A	-178.9(19)	C5A	C6A	C10A	N2A	-150.1(12)
O3	C25	C24	C23	177.3(13)	C5A	C4A	C3A	C2A	2(2)
C31A	C30A	C28A	C27A	176.1(16)	C8	N1	C7	C2	79.2(16)
C31A	C30A	C28A	C29A	-5(3)	C7A	C2A	C3A	C4A	-178.8(15)
C31A	C30A	C25A	O3A	-178.8(14)	C32A	C23A	C24A	C25A	0(2)
C31A	C30A	C25A	C24A	4(2)	N1A	C7A	C2A	C1A	32(2)
C11	N2	C10	C6	80.3(14)	N1A	C7A	C2A	C3A	-152.5(14)
C25A	C30A	C28A	C27A	-1(2)	C6A	C1A	C2A	C7A	179.0(13)
C25A	C30A	C28A	C29A	178.7(15)	C6A	C1A	C2A	C3A	3(2)
C25A	C30A	C31A	C32A	-2(2)	C6A	C5A	C4A	C3A	-1(2)
C25A	O3A	C26A	C27A	-2(2)	C19A	C18A	C17A	C16A	175.4(14)
C25A	O3A	C26A	O4A	176.6(15)	C31	C30	C25	O3	-179.0(14)
C21	C20	C18	C17	-178.5(14)	C31	C30	C25	C24	1(2)
C21	C20	C18	C19	1(2)	C7	C2	C3	C4	-177.8(14)
C21	C20	C15	C14	0(2)	C7	C2	C1	Bi1	-0.8(18)
C21	C20	C15	O1	-179.7(12)	C7	C2	C1	C6	178.8(13)
C21	C22	C13	S1	-177.8(11)	C12A	N2A	C10A	C6A	-157.9(11)
C21	C22	C13	C14	1(2)	C4A	C5A	C6A	C1A	1(2)
C23	C32	C31	C30	0(2)	C4A	C5A	C6A	C10A	-177.4(13)
C16A	O1A	C15A	C20A	-2(2)	C2A	C1A	C6A	C5A	-2(2)
C16A	O1A	C15A	C14A	179.0(13)	C2A	C1A	C6A	C10A	176.3(13)
C13	C14	C15	C20	1(2)	C2A	C7A	N1A	Bi1A	-38.8(15)
C13	C14	C15	O1	-179.2(12)	C2A	C7A	N1A	C8A	79.9(16)
O1	C16	C17	C18	2(2)	C2A	C7A	N1A	C9A	-158.3(15)
C27	C28	C30	C25	-3(2)	C29	C28	C30	C25	177.9(15)
C27	C28	C30	C31	177.0(17)	C29	C28	C30	C31	-2(3)
C21A	C20A	C15A	O1A	179.9(12)	C29	C28	C27	C26	-179.8(18)
C21A	C20A	C15A	C14A	-1(2)	C9	N1	C7	C2	-157.4(13)

a)

b)

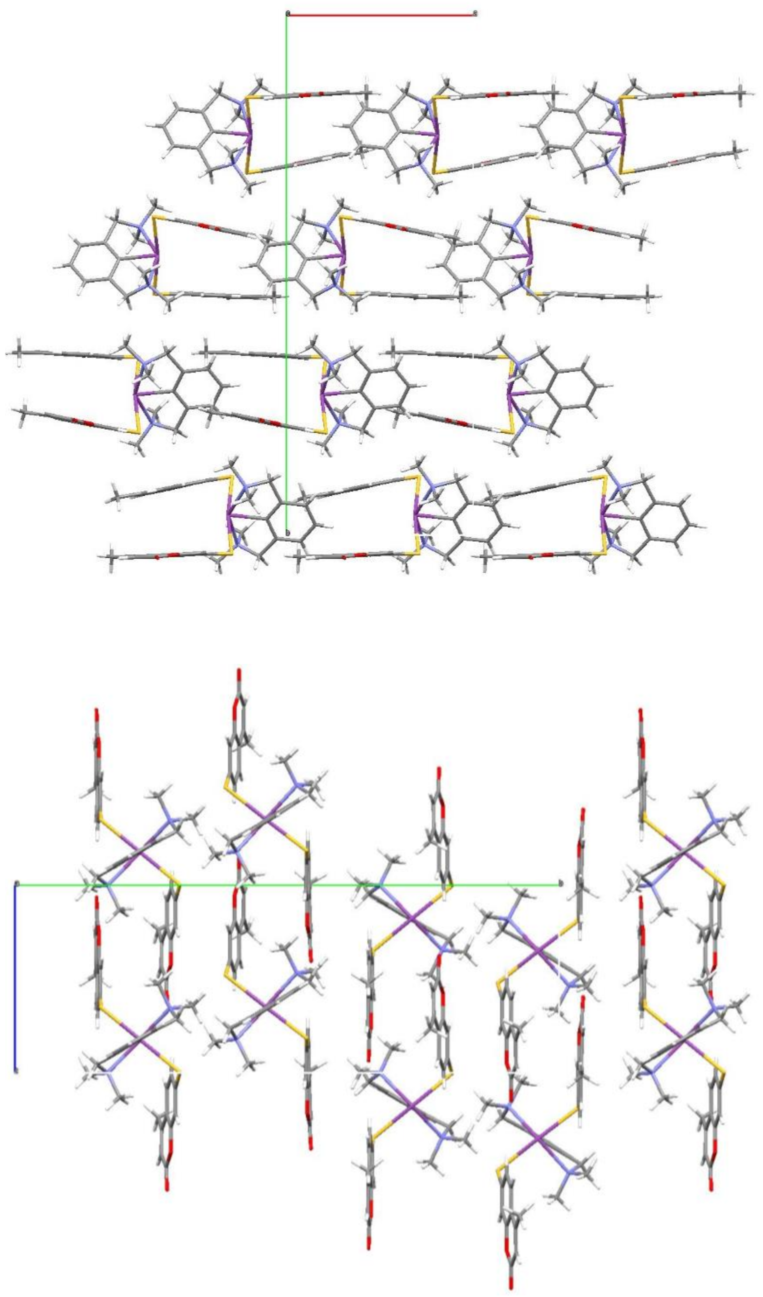

Figure S28. Packing of molecules of complex **2** in the crystal viewed along a) the *c*-axis of the unit cell, and b) along the *a*-axis of the unit cell.

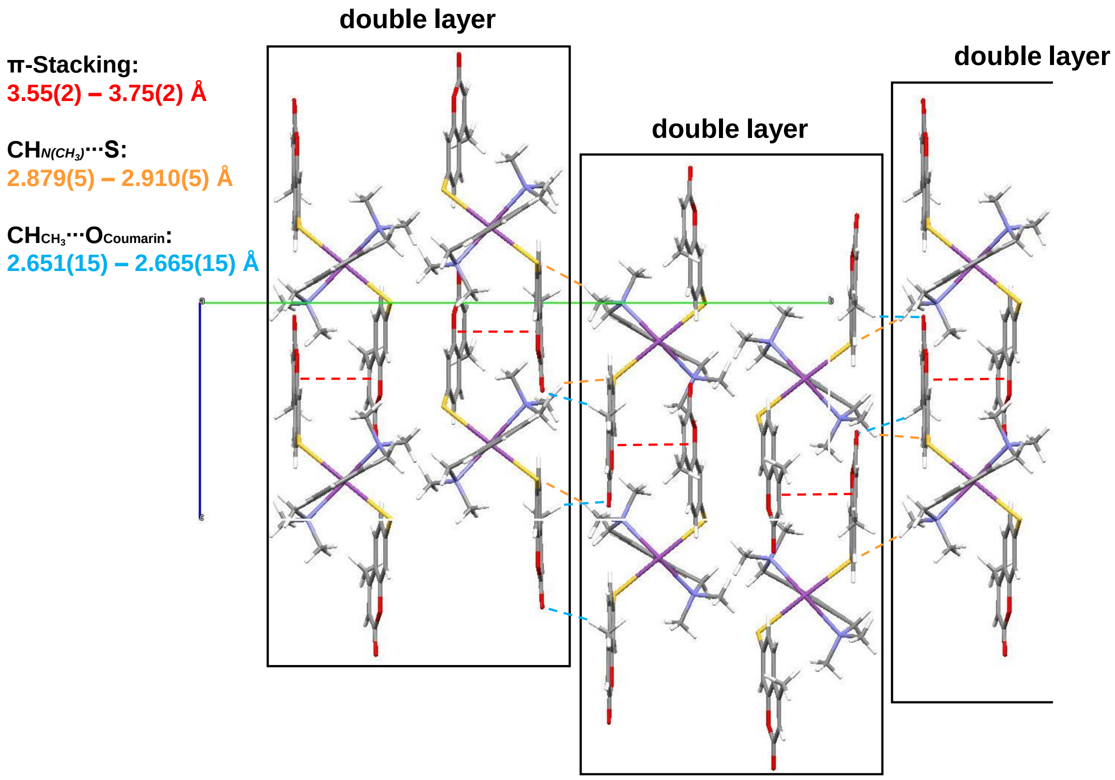

Figure S29. The pattern of hydrogen bonds and π-stacking interactions in crystalline **2** viewed along the *a*-axis of the unit cell.

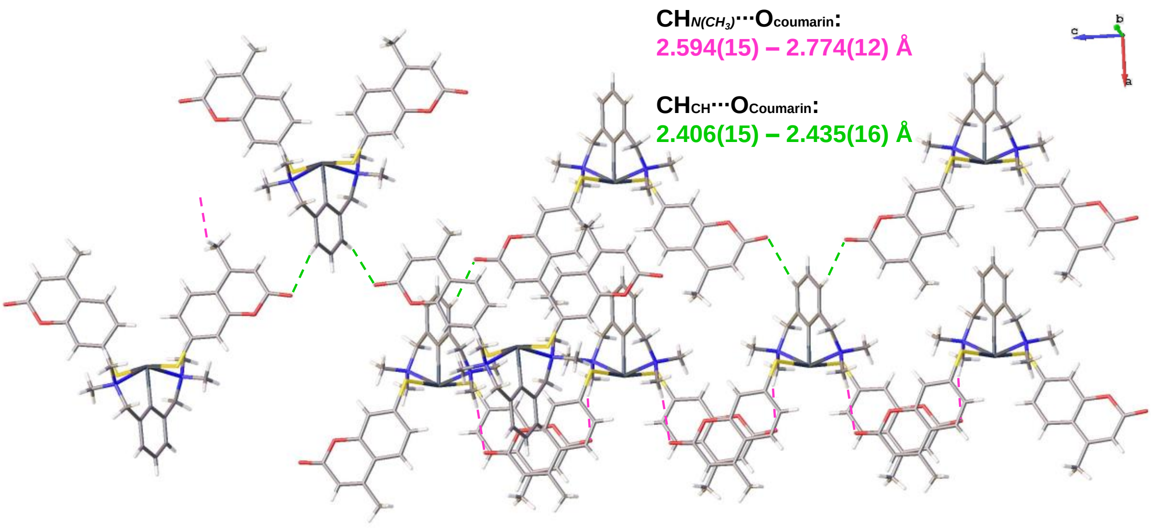

Figure S30. The pattern of hydrogen bonding interactions in crystalline **2**.

Table S9. Crystal data and structure refinement for **1**.

Identification code	**1**
Empirical formula	$C_{56}H_{49}BiN_2S_2$
Formula weight	1023.07
Temperature/K	123.15
Crystal system	monoclinic
Space group	*C*2/*c*
a/Å	18.0655(4)
b/Å	10.1411(2)
c/Å	24.6004(5)
α/°	90
β/°	95.567(2)
γ/°	90
Volume/Å^3	4485.64(16)
Z	4
ρ_{calc}g/cm^3	1.515
μ/mm^{-1}	8.886
F(000)	2056.0
Crystal size/mm^3	0.089 × 0.071 × 0.052
Radiation	CuKα (λ = 1.54184)
2Θ range for data collection/°	7.22 to 150.736
Index ranges	$-22 \leq h \leq 22$, $-11 \leq k \leq 12$, $-30 \leq l \leq 27$
Reflections collected	20630
Independent reflections	4543 [R_{int} = 0.0292, R_{sigma} = 0.0175]
Data/restraints/parameters	4543/257/498
Goodness-of-fit on F^2	1.060
Final R indexes [I>=2σ (I)]	R_1 = 0.0326, wR_2 = 0.0746
Final R indexes [all data]	R_1 = 0.0330, wR_2 = 0.0749
Largest diff. peak/hole / e Å^{-3}	2.74/-2.54

Table S10. Bond Lengths [Å] for **1**.

Atom	Atom	Length/Å	Atom	Atom	Length/Å
Bi1	S1	2.7621(10)	C11	C12	1.429(7)
Bi1	S1a	2.7621(10)	C20	C19	1.352(6)
Bi1	C1	2.195(6)	C19	C18	1.428(7)
Bi1	N1	2.585(4)	C18	C17	1.404(6)
Bi1	N1a	2.585(4)	C9	C10	1.375(7)
S1	C8	1.772(5)	C2	C3	1.392(7)
C1	C2	1.387(5)	C2	C5	1.504(7)
C1	C2a	1.387(5)	C12	C13	1.345(8)
N1	C6	1.475(6)	C14	C13	1.433(8)
N1	C7	1.474(7)	C14	C15	1.411(7)
N1	C5	1.471(6)	C26	C25	1.384(8)
C22	C21	1.420(6)	C26	C27	1.383(8)
C22	C23	1.428(6)	C25	C24	1.379(8)
C22	C11	1.426(6)	C28	C27	1.391(7)
C21	C8	1.424(6)	C28	C29	1.385(8)
C21	C20	1.440(6)	C17	C16	1.382(8)
C8	C9	1.398(6)	C3	C4	1.383(8)
C23	C18	1.421(6)	C16	C15	1.381(8)
C23	C14	1.421(6)	C29	C24	1.378(8)
C11	C10	1.390(7)			

Table S11. Bond Angles [°] for **1**.

Atom	Atom	Atom	Angle/°	Atom	Atom	Atom	Angle/°
S1	Bi1	S1a	179.26(5)	C14	C23	C18	120.2(4)
C1	Bi1	S1	90.37(2)	C22	C11	C12	118.9(4)
C1	Bi1	S1a	90.37(2)	C10	C11	C22	118.0(4)
C1	Bi1	N1	71.41(9)	C10	C11	C12	123.0(5)
C1	Bi1	N1a	71.41(9)	C19	C20	C21	121.4(4)
N1a	Bi1	S1a	86.79(8)	C20	C19	C18	121.8(4)
N1	Bi1	S1	86.79(8)	C23	C18	C19	118.5(4)
N1a	Bi1	S1	93.44(8)	C17	C18	C23	118.8(5)
N1	Bi1	S1a	93.44(8)	C17	C18	C19	122.7(5)
N1a	Bi1	N1	142.81(18)	C10	C9	C8	122.4(5)
C8	S1	Bi1	96.58(14)	C9	C10	C11	121.5(4)
C2	C1	Bi1	119.4(3)	C1	C2	C3	119.4(5)
C2a	C1	Bi1	119.4(3)	C1	C2	C5	118.1(5)
C2a	C1	C2	121.2(6)	C3	C2	C5	122.5(5)
C6	N1	Bi1	112.5(3)	C13	C12	C11	122.1(5)
C7	N1	Bi1	112.3(3)	C23	C14	C13	119.4(5)
C7	N1	C6	109.8(4)	C15	C14	C23	118.4(5)
C5	N1	Bi1	101.9(3)	C15	C14	C13	122.2(5)
C5	N1	C6	110.0(4)	C27	C26	C25	119.9(5)
C5	N1	C7	110.1(4)	C24	C25	C26	120.4(5)
C21	C22	C23	120.4(4)	C12	C13	C14	120.5(5)
C21	C22	C11	120.4(4)	C29	C28	C27	119.8(5)
C11	C22	C23	119.2(4)	C16	C17	C18	120.8(5)
C22	C21	C8	119.8(4)	C4	C3	C2	119.1(6)
C22	C21	C20	118.0(4)	C26	C27	C28	119.8(5)
C8	C21	C20	122.2(4)	N1	C5	C2	110.8(4)
C21	C8	S1	122.2(3)	C15	C16	C17	120.9(5)
C9	C8	S1	120.1(4)	C16	C15	C14	120.8(5)
C9	C8	C21	117.7(4)	C24	C29	C28	120.3(5)
C18	C23	C22	119.8(4)	C29	C24	C25	119.9(5)
C14	C23	C22	119.9(4)	C3a	C4	C3	121.7(7)

Table S12. Torsion Angles [°] for **1**.

A	B	C	D	Angle/°	A	B	C	D	Angle/°
Bi1	S1	C8	C21	-114.9(3)	C11	C22	C21	C20	-177.9(4)
Bi1	S1	C8	C9	67.3(3)	C11	C22	C23	C18	179.3(4)
Bi1	C1	C2	C3	-179.8(3)	C11	C22	C23	C14	-0.4(6)
Bi1	C1	C2	C5	3.1(4)	C11	C12	C13	C14	0.7(8)
Bi1	N1	C5	C2	44.3(4)	C20	C21	C8	S1	0.1(5)
S1	C8	C9	C10	178.8(4)	C20	C21	C8	C9	178.0(4)
C1	C2	C3	C4	-0.4(7)	C20	C19	C18	C23	2.6(6)
C1	C2	C5	N1	-37.0(6)	C20	C19	C18	C17	-178.4(4)
C22	C21	C8	S1	-180.0(3)	C19	C18	C17	C16	179.8(4)
C22	C21	C8	C9	-2.1(6)	C18	C23	C14	C13	-178.1(4)
C22	C21	C20	C19	-1.4(6)	C18	C23	C14	C15	1.9(6)
C22	C23	C18	C19	-1.4(6)	C18	C17	C16	C15	2.0(8)
C22	C23	C18	C17	179.4(4)	C10	C11	C12	C13	-178.8(5)
C22	C23	C14	C13	1.6(6)	C2a	C1	C2	C3	0.2(3)
C22	C23	C14	C15	-178.4(4)	C2a	C1	C2	C5	-176.9(4)
C22	C11	C10	C9	-0.4(7)	C2	C3	C4	C3a	0.2(3)
C22	C11	C12	C13	0.6(7)	C12	C11	C10	C9	179.0(4)
C21	C22	C23	C18	-1.1(6)	C14	C23	C18	C19	178.3(4)
C21	C22	C23	C14	179.2(4)	C14	C23	C18	C17	-0.8(6)
C21	C22	C11	C10	-0.9(6)	C26	C25	C24	C29	-0.6(8)
C21	C22	C11	C12	179.7(4)	C25	C26	C27	C28	-0.5(8)
C21	C8	C9	C10	0.9(6)	C13	C14	C15	C16	178.9(5)
C21	C20	C19	C18	-1.1(7)	C28	C29	C24	C25	-0.1(8)
C8	C21	C20	C19	178.5(4)	C17	C16	C15	C14	-0.9(8)
C8	C9	C10	C11	0.4(7)	C6	N1	C5	C2	-75.2(5)
C23	C22	C21	C8	-177.4(3)	C3	C2	C5	N1	146.0(5)
C23	C22	C21	C20	2.5(5)	C27	C26	C25	C24	0.9(8)
C23	C22	C11	C10	178.7(4)	C27	C28	C29	C24	0.5(9)
C23	C22	C11	C12	-0.7(6)	C7	N1	C5	C2	163.7(4)
C23	C18	C17	C16	-1.1(7)	C5	C2	C3	C4	176.6(4)
C23	C14	C13	C12	-1.8(7)	C15	C14	C13	C12	178.2(5)
C23	C14	C15	C16	-1.0(7)	C29	C28	C27	C26	-0.2(8)
C11	C22	C21	C8	2.2(6)					

a)

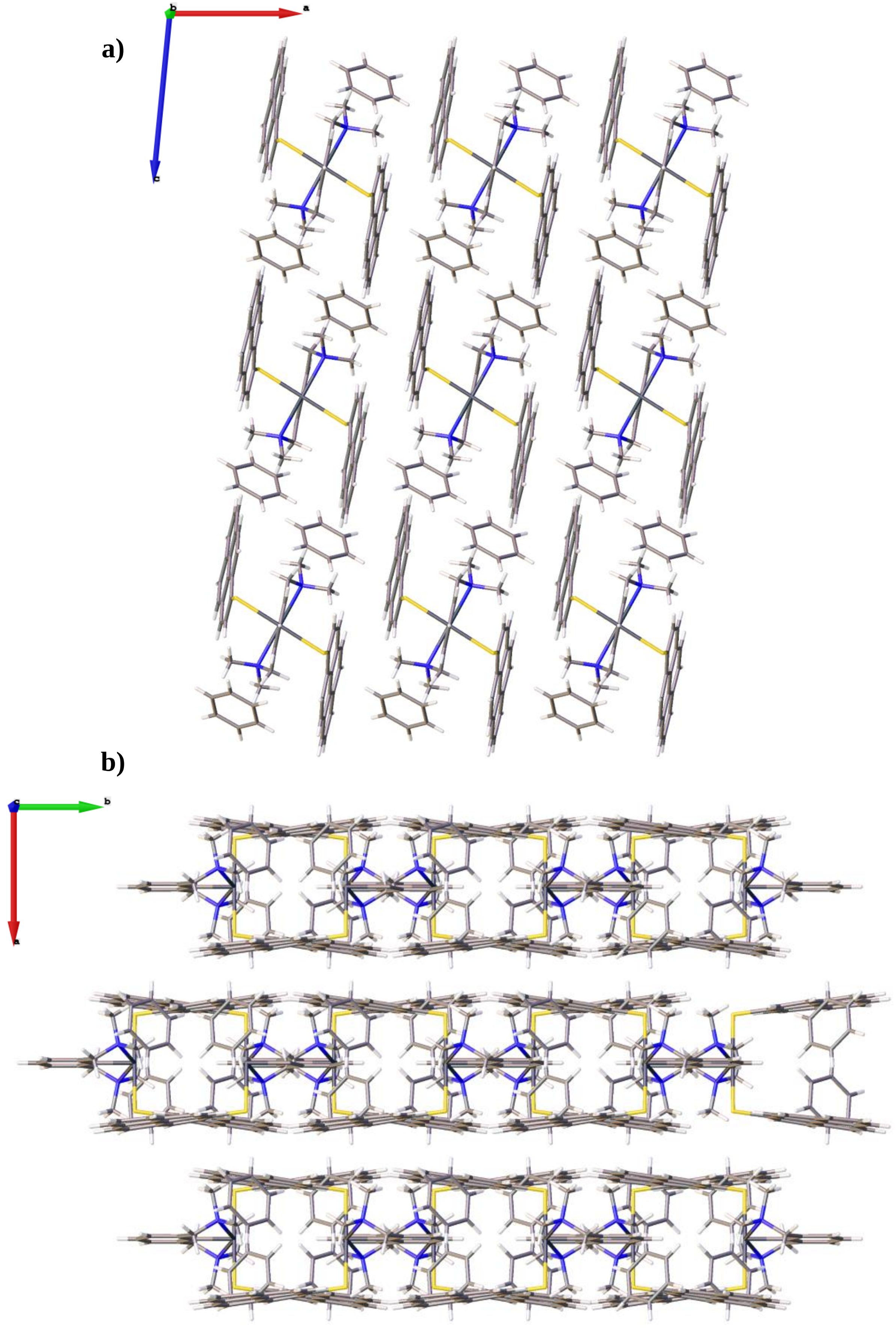

b)

Figure S31. Packing of molecules of complex **1** in the crystal viewed along a) the *b*-axis of the unit cell, and b) along the *c*-axis of the unit cell.

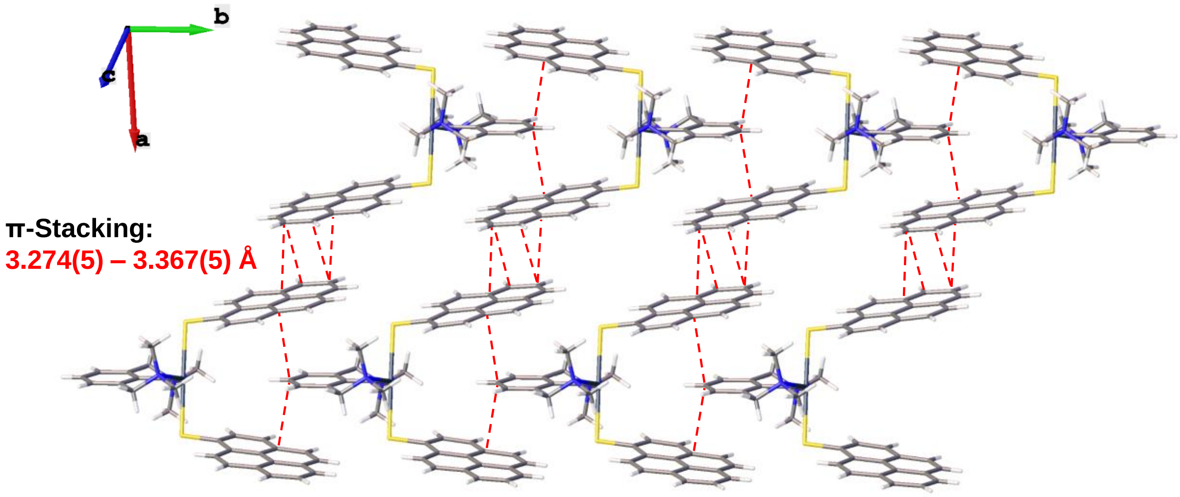

Figure S32. The pattern of π-stacking interactions in crystalline **1**.

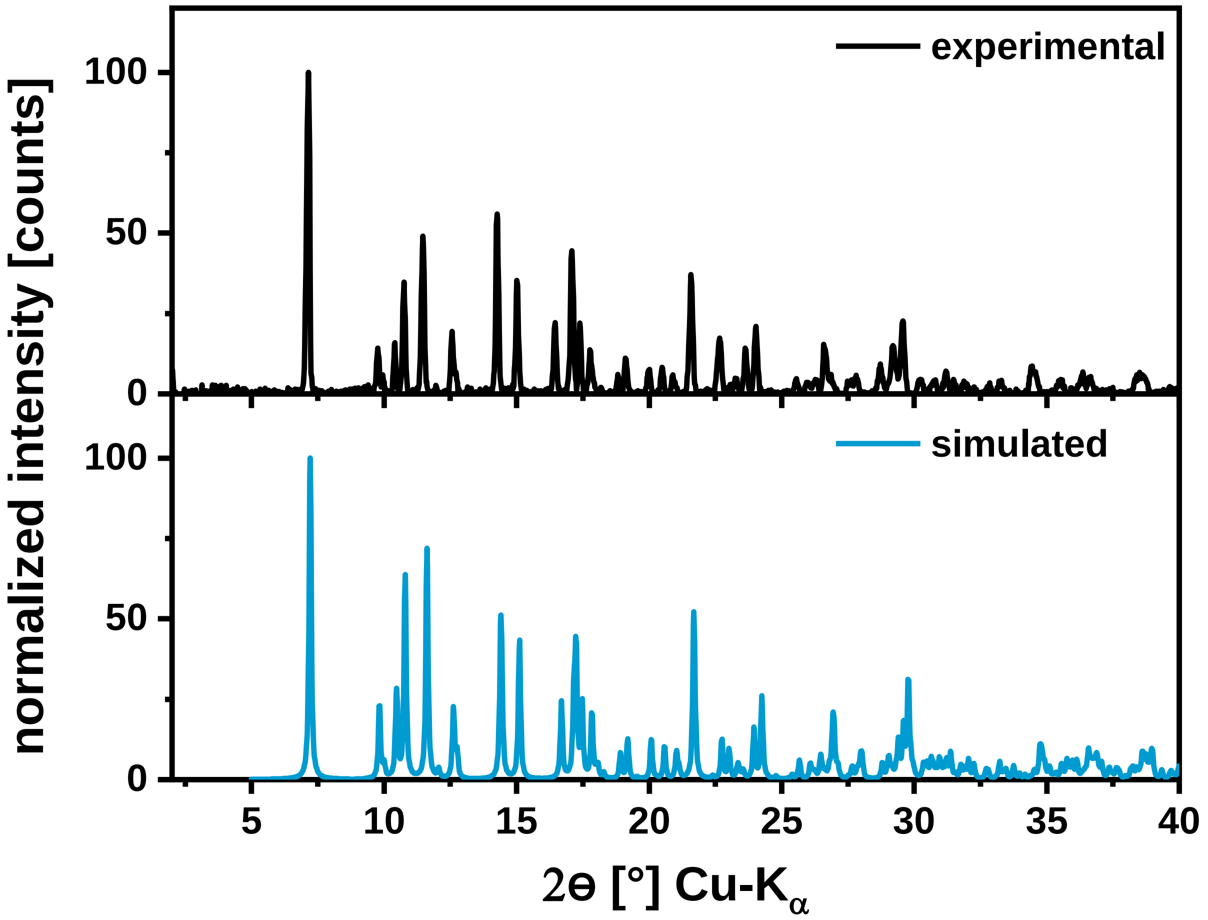

Figure S33. PXRD of **1** along with comparison of the experimental (black line) and the calculated (blue line) pattern based on the X-ray structure determination on a single crystal.

Table S13. Selected interatomic distances (Å) and angles (deg) for **1**, **2** and **(*NCN*)^{DAA}BiCl$_2$**.

**Parameter**	**1**	**2**	**Parameter**	**(*NCN*)^{DAA}BiCl$_2$**
Bi(1)-C(1)	2.195(6)	2.190(11)	Bi(1)-C(1)	2.187(6)
Bi(1)-S(1)	2.762(1)	2.762(4)	Bi(1)-Cl(1)	2.709(2)
Bi(1)-S(2)	2.762(1)	2.744(4)	Bi(1)-Cl(2)	2.705(2)
Bi(1)-N(1)	2.585(3)	2.527(13)	Bi(1)-N(1)	2.513(7)
Bi(1)-N(2)	2.585(3)	2.535(13)	Bi(1)-N(2)	2.543(7)
S(1)-C(13)	1772(5)	1.770(14)	N(3)-C(4)	1.403(9)
S(2)-C(23)	1772(5)	1.748(14)	N(3)-C(13)	1.431(9)
			N(3)-C(20)	1.427(9)
N(1)-Bi(1)-N(2)	142.82(18)	144.7(4)	Cl(1)-Bi(1)-Cl(2)	176.33(6)
S(1)-Bi(1)-S(2)	179.26(5)	170.85(12)	Cl(1)-Bi(1)-C(1)	88.5(2)
N(1)-Bi(1)-C(1)	71.41(9)	72.0(5)	Cl(2)-Bi(1)-C(1)	87.8(2)
N(2)-Bi(1)-C(1)	71.41(9)	72.7(5)	N(1)-Bi(1)-N(2)	145.0(2)
S(1)-Bi(1)-C(1)	90.37(2)	88.3(4)	N(1)-Bi(1)-C(1)	72.8(3)
S(2)-Bi(1)-C(1)	90.37(2)	82.6(4)	N(2)-Bi(1)-C(1)	72.3(3)
S(2)-Bi(1)-N(1)	93.44(8)	84.4(3)	Cl(1)-Bi(1)-N(1)	96.47(16)
S(2)-Bi(1)-N(2)	86.79(8)	93.1(3)	Cl(1)-Bi(1)-N(2)	82.87(16)
S(1)-Bi(1)-N(2)	93.44(8)	84.2(3)	Cl(2)-Bi(1)-N(1)	82.38(16)
S(1)-Bi(1)-N(1)	86.79(8)	92.7(3)	Cl(2)-Bi(1)-N(2)	96.06(16)
Bi(1)-S(1)-C(13)	96.58(13)	96.8(5)	C(4)-N(3)-C(13)	120.6(6)
Bi(1)-S(2)-C(23)	96.58(13)	100.3(5)	C(13)-N(3)-C(20)	117.5(6)
◊PhS(1)–◊PhS(2)	17.30(19)	8.5(7)	C(20)-N(3)-C(4)	121.4(6)
◊PhS(1)–◊PhBi(1)	9.09(19)	58.7(7)		
◊PhS(2)–◊PhBi(1)	9.09(19)	57.2(7)		

TD-DFT Data

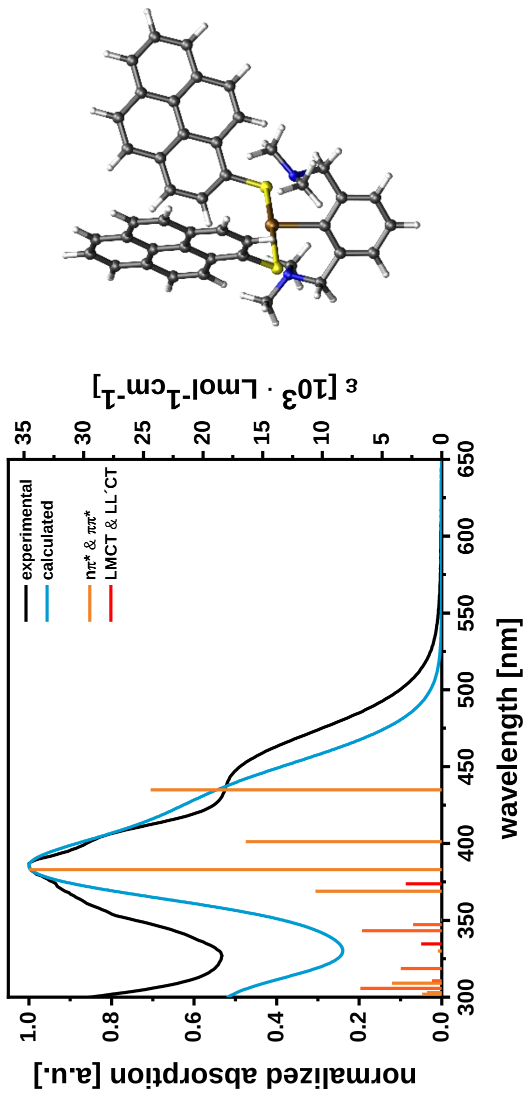

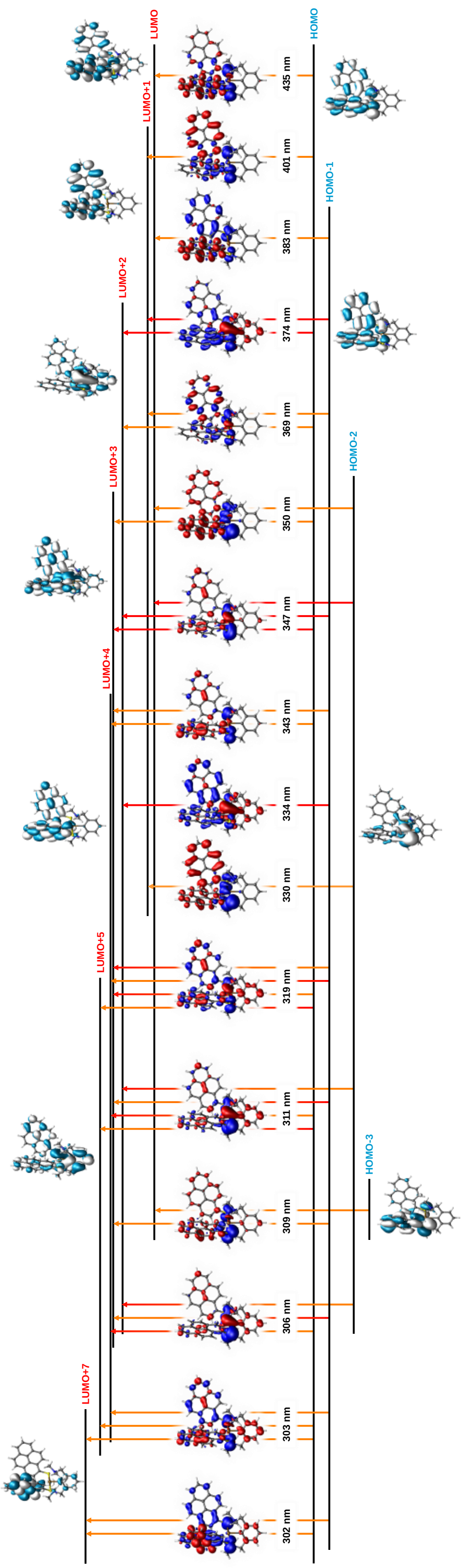

Figure S34. Left: Geometry-optimized structure of the *transoid* conformer of complex **1** along with comparison between the experimental (black line) and the TD-DFT-computed (blue line) electronic absorption spectra. Individual electronic transitions are indicated as colored bars. Right: MO diagrams of relevant molecular orbitals involved in the individual TD-DFT-computed electronic transitions along with the corresponding electron density difference maps. A loss of electron density is indicated in blue, a gain in red color.

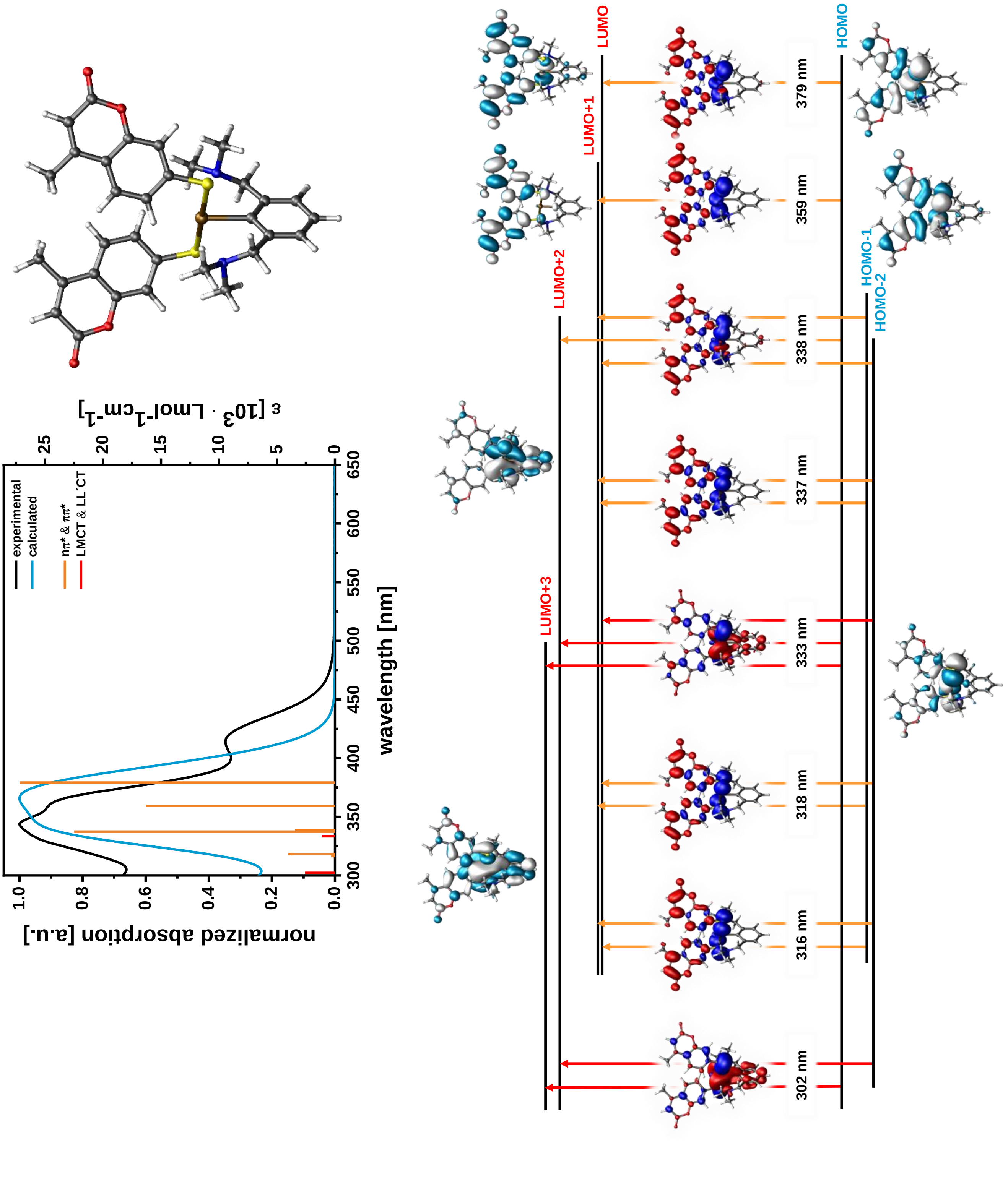

Figure S35. Left: Geometry-optimized structure of complex **2** along with comparison between the experimental (black line) and the TD-DFT-computed (blue line) electronic absorption spectra. Individual electronic transitions are indicated as colored bars. Right: MO diagrams of relevant molecular orbitals involved in the individual TD-DFT-computed electronic transitions along with the corresponding electron density difference maps. A loss of electron density is indicated in blue, a gain in red color.

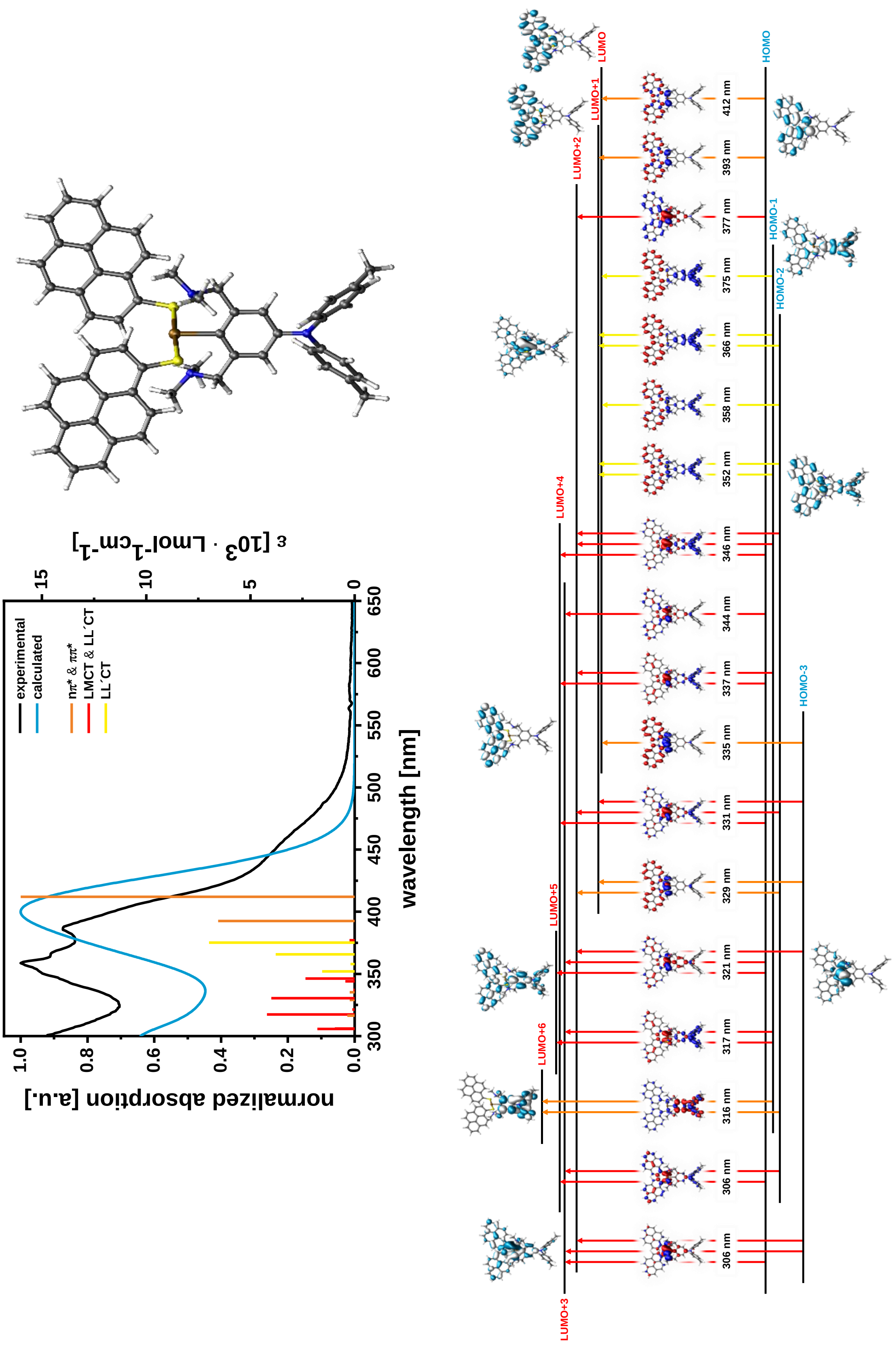

Figure S36. Left: Geometry-optimized structure of *transoid* conformer of complex **3** along with comparison between the experimental (black line) and the TD-DFT-computed (blue line) electronic absorption spectra. Individual electronic transitions are indicated as colored bars. Right: MO diagrams of relevant molecular orbitals involved in the individual TD-DFT-computed electronic transitions along with the corresponding electron density difference maps. A loss of electron density is indicated in blue, a gain in red color.

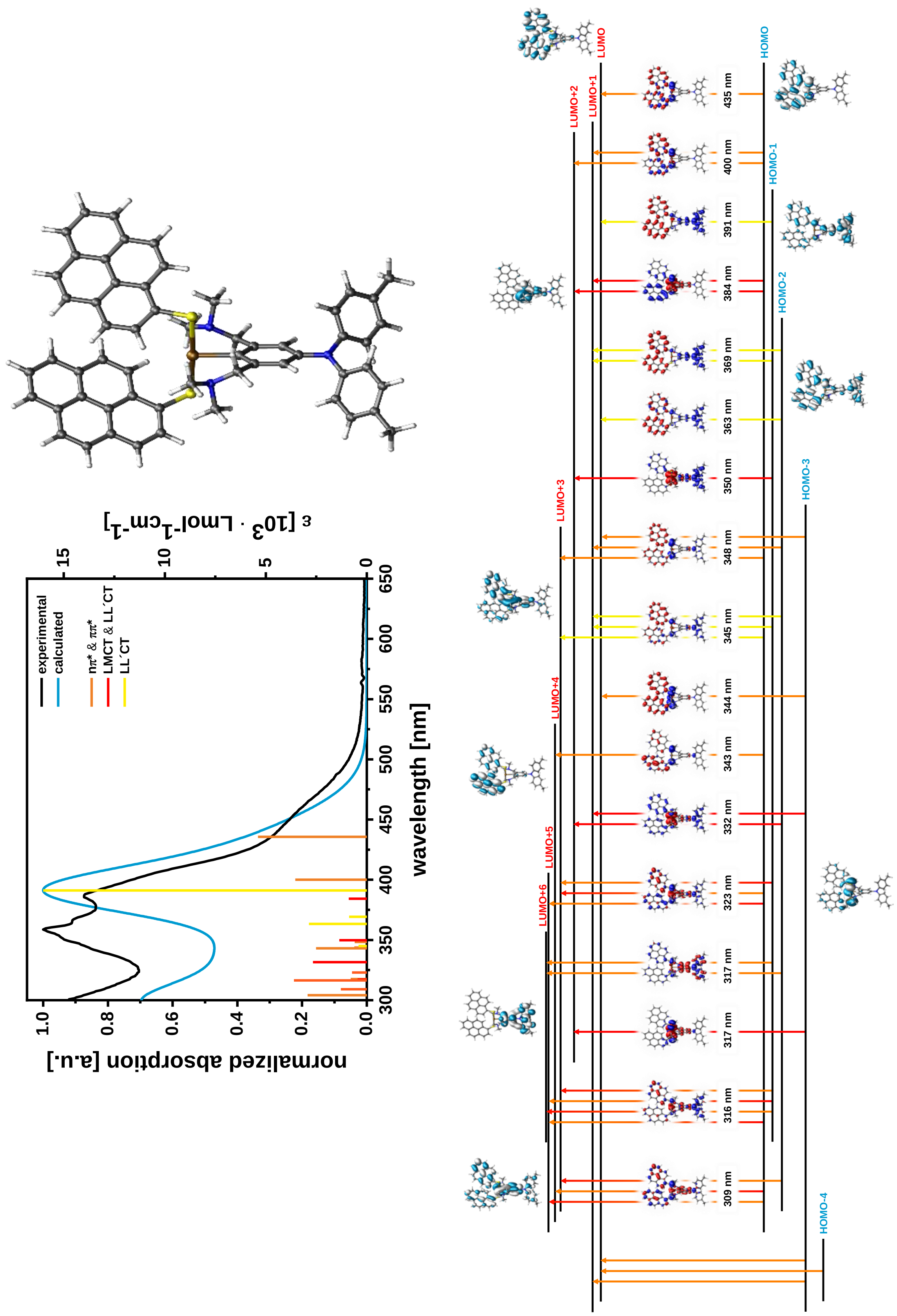

Figure S37. Left: Geometry-optimized structure of cisoid conformer of complex **3** along with comparison between the experimental (black line) and the TD-DFT-computed (blue line) electronic absorption spectra. Individual electronic transitions are indicated as colored bars. Right: MO diagrams of relevant molecular orbitals involved in the individual TD-DFT-computed electronic transitions along with the corresponding electron density difference maps. A loss of electron density is indicated in blue, a gain in red color.

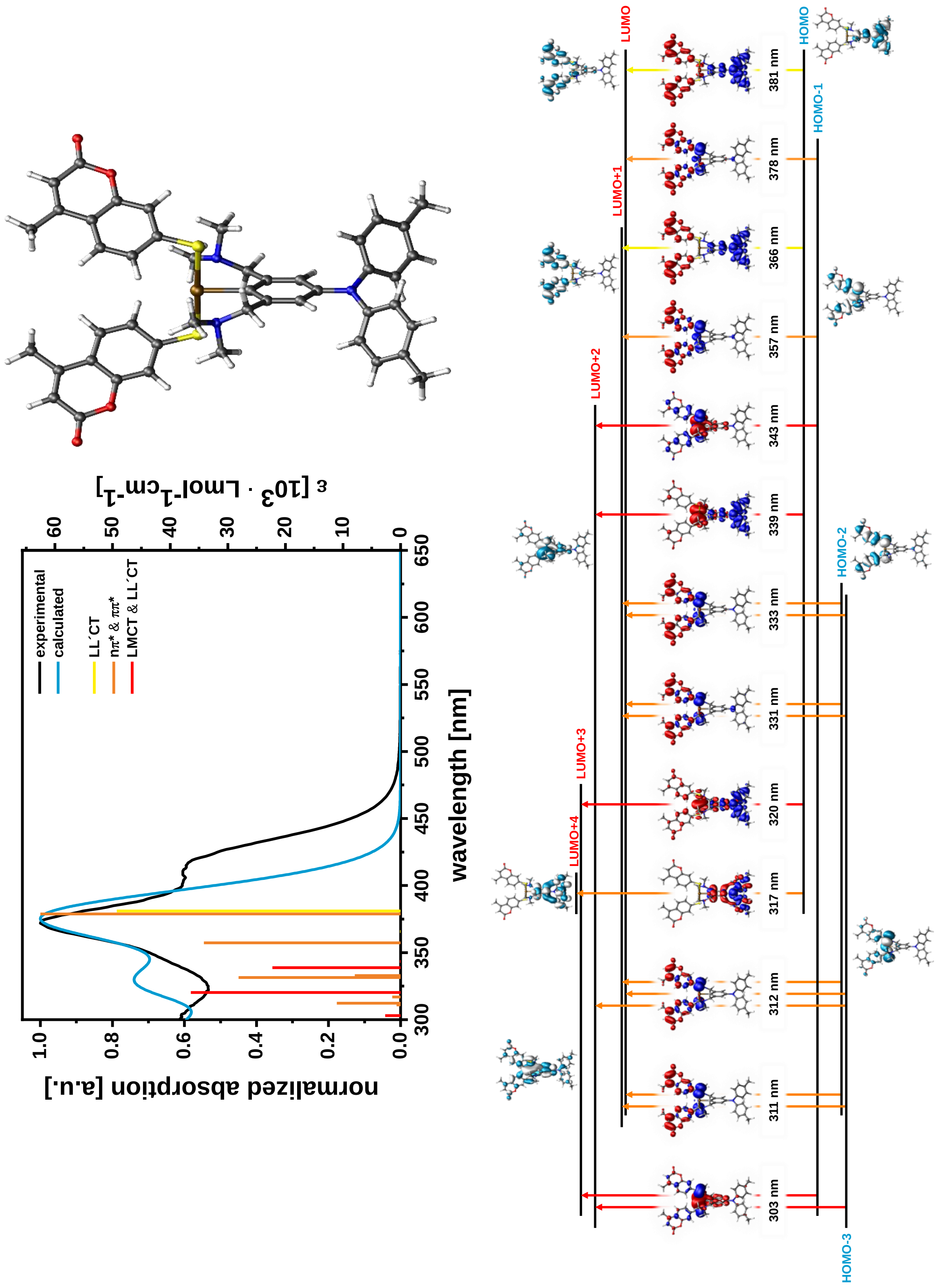

Figure S38. Left: Geometry-optimized structure of complex **4** along with comparison between the experimental (black line) and the TD-DFT-computed (blue line) electronic absorption spectra. Individual electronic transitions are indicated as colored bars. Right: MO diagrams of relevant molecular orbitals involved in the individual TD-DFT-computed electronic transitions along with the corresponding electron density difference maps. A loss of electron density is indicated in blue, a gain in red color.

Table S14. XYZ coordinates of the DFT-optimized structure of the *cisoid* conformer of complex **1**.

Atom	X	Y	Z
Bi	1.59491	0.57638	0.01501
S	0.26603	2.41467	1.61731
C	3.39030	1.86889	-0.05215
N	3.04405	0.05045	2.08044
C	2.24579	-0.38669	3.22881
H	1.48264	0.36398	3.45032
H	2.88130	-0.52995	4.11533
H	1.75940	-1.33717	2.99087
N	1.46649	2.05523	-2.11907
C	5.30242	2.82537	1.05605
H	5.92143	2.94328	1.94312
C	4.03640	-0.97652	1.74592
H	3.52727	-1.91039	1.49664
H	4.70728	-1.14997	2.60077
H	4.62798	-0.66040	0.88392
C	4.85307	3.34515	-1.26800
H	5.12283	3.86768	-2.18352
C	5.63725	3.48893	-0.12381
H	6.51865	4.12454	-0.15195
C	3.71318	1.33236	2.36558
H	4.55579	1.17237	3.05478
H	2.98602	1.97194	2.88097
C	0.82420	3.33655	-1.80720
H	-0.21312	3.16642	-1.51047
H	0.84293	3.99403	-2.68970
H	1.34539	3.82813	-0.98305
C	4.16588	2.01506	1.09862
C	2.88308	2.27118	-2.46494
H	3.23987	1.36410	-2.96872
H	2.97391	3.09871	-3.18479
C	3.72490	2.52268	-1.23836
S	2.82409	-1.31516	-1.68362
C	0.75644	1.40864	-3.22585
H	1.21757	0.44179	-3.44315
H	0.78766	2.03504	-4.12991
H	-0.28928	1.25540	-2.94414
C	2.05543	-2.77380	-1.04393
C	0.65463	-3.01199	-1.08802
C	2.87956	-3.75407	-0.46197
C	0.12156	-4.19842	-0.49445
C	-0.26533	-2.10916	-1.71697
C	2.36601	-4.91729	0.08815
H	3.95234	-3.58248	-0.44599
C	-1.28730	-4.42976	-0.49086
C	0.98574	-5.16034	0.10165
C	-1.60800	-2.33112	-1.71923
H	0.14196	-1.23826	-2.22051
H	3.03863	-5.65011	0.52885
C	-2.17154	-3.48851	-1.09220
C	-1.82019	-5.60544	0.11713
C	0.42510	-6.33669	0.69487
H	-2.27970	-1.62926	-2.20851
C	-3.55329	-3.72676	-1.06534
C	-3.20856	-5.80357	0.12033
C	-0.91787	-6.55059	0.70708
H	1.10365	-7.05979	1.14221
C	-4.06274	-4.87235	-0.46223
H	-4.22327	-3.00226	-1.52253
H	-3.61195	-6.69975	0.58621
H	-1.33278	-7.44586	1.16438
H	-5.13614	-5.04367	-0.44929
C	-1.32122	1.64441	1.46869
C	-2.44250	2.33562	0.93537
C	-1.49024	0.31012	1.87600
C	-3.69304	1.65710	0.80975

C	-2.37498	3.70465	0.51324
C	-2.70238	-0.35153	1.74648
H	-0.64404	-0.21505	2.31114
C	-4.82664	2.33538	0.26938
C	-3.82227	0.29646	1.21150
C	-3.45676	4.35313	0.00325
H	-1.42516	4.22022	0.61722
H	-2.78911	-1.38805	2.06492
C	-4.72091	3.69551	-0.13997
C	-6.07306	1.65454	0.13363
C	-5.08466	-0.36378	1.06214
H	-3.37743	5.39295	-0.30631
C	-5.84650	4.34424	-0.66822
C	-7.17190	2.34203	-0.40062
C	-6.16308	0.28379	0.54593
H	-5.15953	-1.40403	1.37137
C	-7.05725	3.67165	-0.79554
H	-5.76061	5.38315	-0.97834
H	-8.12081	1.82076	-0.50391
H	-7.11677	-0.22731	0.43643
H	-7.92078	4.18788	-1.20718

Table S15. XYZ coordinates of the DFT-optimized structure of the *transoid* conformer of complex **1**.

Atom	X	Y	Z
Bi	-1.73635	-0.36640	-0.21666
S	-3.12062	1.99624	0.47065
C	-3.53102	-1.57560	0.24555
N	-3.30287	-0.38668	-2.31019
C	-3.19821	0.82657	-3.12462
H	-3.38748	1.70420	-2.50118
H	-3.92570	0.80894	-3.95004
H	-2.19214	0.89318	-3.54899
N	-1.48807	-1.26588	2.17367
C	-5.79209	-2.23492	-0.25422
H	-6.68080	-2.17003	-0.87871
C	-3.03808	-1.56083	-3.14889
H	-2.02343	-1.51021	-3.54898
H	-3.75569	-1.59933	-3.98259
H	-3.13158	-2.47464	-2.55797
C	-4.64650	-3.16777	1.66652
H	-4.64654	-3.82579	2.53314
C	-5.77927	-3.07494	0.85891
H	-6.66135	-3.66287	1.09958
C	-4.64490	-0.49284	-1.70915
H	-5.38209	-0.76906	-2.47809
H	-4.91854	0.50347	-1.34009
C	-2.10312	-0.33377	3.12439
H	-1.57266	0.62088	3.09598
H	-2.05186	-0.74250	4.14479
H	-3.14860	-0.16207	2.85862
C	-4.66398	-1.47171	-0.56141
C	-2.23017	-2.53884	2.14882
H	-1.58137	-3.28249	1.67002
H	-2.42686	-2.88119	3.17557
C	-3.50823	-2.42005	1.35664
S	-0.41822	-2.64791	-1.09239
C	-0.08277	-1.48366	2.52847
H	0.38106	-2.15488	1.80134
H	0.00402	-1.92443	3.53248
H	0.44628	-0.52641	2.51823
C	1.22663	-1.99629	-1.13031
C	2.31273	-2.67967	-0.51921
C	1.48842	-0.78275	-1.78898
C	3.62379	-2.11630	-0.58003
C	2.15068	-3.93235	0.16024
C	2.75802	-0.22655	-1.83312
H	0.67059	-0.26927	-2.28842
C	4.72507	-2.79389	0.02421
C	3.84788	-0.87229	-1.23656
C	3.20050	-4.57969	0.73404
H	1.15439	-4.36198	0.20280
H	2.91520	0.72146	-2.34314
C	4.52534	-4.03800	0.68822
C	6.03393	-2.22898	-0.03218
C	5.17143	-0.32657	-1.27604
H	3.04798	-5.52973	1.24150
C	5.62057	-4.68902	1.27413
C	7.09969	-2.91417	0.56805
C	6.21957	-0.97399	-0.70085
H	5.31931	0.62574	-1.78057
C	6.89276	-4.13013	1.21256
H	5.46228	-5.63918	1.77913
H	8.09640	-2.48114	0.52384
H	7.22074	-0.55081	-0.73732
H	7.73194	-4.64632	1.67183
C	-1.73768	2.93948	1.04187
C	-0.66659	3.35236	0.20372
C	-1.69950	3.30584	2.39938
C	0.43254	4.07354	0.76483

C	-0.64101	3.08689	-1.20497
C	-0.64552	4.02519	2.93924
H	-2.52736	3.00901	3.03741
C	1.53163	4.46346	-0.05847
C	0.44517	4.41082	2.14793
C	0.40216	3.46498	-1.99298
H	-1.50067	2.58896	-1.64110
H	-0.65405	4.28718	3.99513
C	1.53440	4.15231	-1.44871
C	2.63534	5.16994	0.50543
C	1.56182	5.12723	2.68611
H	0.38665	3.25885	-3.06119
C	2.62683	4.53805	-2.23882
C	3.70769	5.53434	-0.32172
C	2.61340	5.48853	1.90305
H	1.55096	5.37525	3.74523
C	3.70169	5.21959	-1.67694
H	2.62160	4.30067	-3.30023
H	4.54865	6.07069	0.11177
H	3.45808	6.02910	2.32382
H	4.54190	5.51150	-2.30183

Table S16. XYZ coordinates of the DFT-optimized structure of the *transoid* conformer of complex **2**.

Atom	X	Y	Z
Bi	-1.37556	0.00040	0.00007
S	-1.38046	-2.49452	1.24166
O	2.47230	-5.41243	-0.31672
C	-3.58309	0.00096	-0.00015
O	3.97407	-6.84273	-1.08642
N	-2.17898	0.94061	2.27125
C	-1.24143	0.63965	3.35647
H	-1.05743	-0.43730	3.39315
H	-1.64320	0.97134	4.32531
H	-0.29766	1.16224	3.17420
C	2.13696	-4.24992	0.30510
C	4.50826	-3.71437	0.59258
N	-2.17899	-0.93944	-2.27126
C	2.67169	-2.18747	1.40867
H	3.40049	-1.48140	1.79674
C	5.58787	-2.80277	1.08087
H	5.50718	-2.64701	2.16357
H	5.50945	-1.81600	0.60816
H	6.57670	-3.21414	0.86274
C	-5.66151	0.16594	1.20052
H	-6.20998	0.29398	2.13142
C	4.80011	-4.88431	-0.03376
H	5.82549	-5.19778	-0.20041
C	1.32264	-1.91950	1.55571
H	1.00874	-1.01569	2.07061
C	3.11821	-3.36218	0.77907
C	-2.38725	2.39009	2.18595
H	-1.43926	2.88748	1.96795
H	-2.78434	2.77238	3.13833
H	-3.09256	2.62197	1.38530
C	0.34449	-2.81540	1.06631
C	3.78248	-5.78799	-0.51879
C	-5.66134	-0.16309	-1.20124
H	-6.20969	-0.29088	-2.13225
C	-6.35095	0.00158	-0.00043
H	-7.43798	0.00183	-0.00054
C	-3.46632	0.25393	2.48388
H	-4.03918	0.76227	3.27351
H	-3.24033	-0.75734	2.84366
C	0.77802	-3.98815	0.43654
H	0.06436	-4.70733	0.04643
C	-2.38800	-2.38881	-2.18600
H	-1.44031	-2.88667	-1.96777
H	-2.78505	-2.77091	-3.13846
H	-3.09361	-2.62032	-1.38551
C	-4.26507	0.15798	1.20676
C	-3.46593	-0.25210	-2.48416
H	-3.23936	0.75906	-2.84389
H	-4.03888	-0.76014	-3.27391
C	-4.26490	-0.15576	-1.20721
S	-1.37892	2.49532	-1.24153
O	2.47548	5.41106	0.31687
C	2.67306	2.18607	-1.40868
H	3.40147	1.47963	-1.79681
O	3.97805	6.84051	1.08657
C	0.34621	2.81524	-1.06620
C	2.13949	4.24876	-0.30500
C	4.51049	3.71194	-0.59260
C	3.12024	3.36051	-0.77904
C	0.78040	3.98773	-0.43639
H	0.06714	4.70727	-0.04623
C	1.32386	1.91884	-1.55569
H	1.00945	1.01523	-2.07062
C	3.78587	5.78590	0.51890
C	4.80299	4.88169	0.03379

H	5.82854	5.19460	0.20041
C	5.58958	2.79979	-1.08097
H	5.51065	1.81303	-0.60832
H	6.57865	3.21060	-0.86286
H	5.50877	2.64412	-2.16368
C	-1.24105	-0.63896	-3.35629
H	-1.05649	0.43790	-3.39293
H	-1.64278	-0.97045	-4.32521
H	-0.29759	-1.16202	-3.17381

Table S17. XYZ coordinates of the DFT-optimized structure of the *cisoid* conformer of complex **3**.

Atom	X	Y	Z
Bi	0.21793	-0.42623	0.00081
S	0.48388	1.49808	-1.97588
C	-1.96883	-0.17904	0.01252
N	-0.78267	-1.94559	-1.83649
C	0.12695	-2.14267	-2.96897
H	0.43218	-1.16916	-3.36180
H	-0.35928	-2.72117	-3.76796
H	1.01215	-2.68944	-2.63179
N	-0.41153	1.27772	1.86446
C	-4.10376	-0.47696	-1.07046
H	-4.69268	-0.81902	-1.91705
C	-1.15466	-3.24153	-1.25708
H	-0.25857	-3.75476	-0.90074
H	-1.65477	-3.86787	-2.01077
H	-1.82710	-3.08916	-0.41021
C	-3.97910	0.58752	1.10295
H	-4.47373	1.05090	1.95213
C	-4.74827	0.13528	0.01757
C	-1.98820	-1.20860	-2.25730
H	-2.64231	-1.86305	-2.85306
H	-1.65693	-0.39220	-2.91080
C	-0.49022	2.62935	1.29808
H	0.49478	2.93869	0.94151
H	-0.83965	3.34152	2.06063
H	-1.18110	2.63769	0.45266
C	-2.72143	-0.62994	-1.07195
C	-1.75084	0.82483	2.28374
H	-1.61079	-0.04647	2.93562
H	-2.24293	1.60546	2.88378
C	-2.59759	0.42775	1.09946
S	0.08562	-2.33716	2.06802
C	0.51476	1.26859	3.00075
H	0.59310	0.25302	3.39746
H	0.16661	1.94291	3.79680
H	1.50056	1.60422	2.66683
C	1.48183	-3.30261	1.56763
C	2.80929	-2.79886	1.52213
C	1.26126	-4.63781	1.18682
C	3.86308	-3.63185	1.03411
C	3.15123	-1.47643	1.95809
C	2.28779	-5.45544	0.74279
H	0.24951	-5.02934	1.24458
C	5.19306	-3.12336	0.93517
C	3.59936	-4.97354	0.63824
C	4.41944	-0.99218	1.86919
H	2.36482	-0.86764	2.39171
H	2.07457	-6.48301	0.45672
C	5.48366	-1.78839	1.33738
C	6.24109	-3.94880	0.43037
C	4.67236	-5.78382	0.14670
H	4.64680	0.01443	2.21222
C	6.79340	-1.30265	1.21708
C	7.53641	-3.42206	0.32825
C	5.93670	-5.29478	0.04265
H	4.45148	-6.80725	-0.14779
C	7.80625	-2.11318	0.71485
H	7.00854	-0.28110	1.52107
H	8.33335	-4.05255	-0.05876
H	6.74203	-5.91921	-0.33661
H	8.81673	-1.72287	0.62782
C	2.23830	1.65959	-1.79109
C	2.84147	2.88889	-1.41463
C	3.06560	0.54405	-1.99958
C	4.25645	2.95125	-1.23355
C	2.08327	4.08853	-1.20914

C	4.43894	0.60169	-1.81489
H	2.61288	-0.39006	-2.32073
C	4.87707	4.17690	-0.84819
C	5.06237	1.79264	-1.42530
C	2.67583	5.25856	-0.84864
H	1.00928	4.03942	-1.35932
H	5.04296	-0.28849	-1.97416
C	4.09128	5.34810	-0.65207
C	6.28898	4.23685	-0.65535
C	6.47736	1.87974	-1.22092
H	2.07613	6.15433	-0.70410
C	4.72046	6.54305	-0.27527
C	6.87524	5.45244	-0.27733
C	7.06638	3.04805	-0.85204
H	7.07370	0.98186	-1.36575
C	6.09790	6.59139	-0.09086
H	4.11639	7.43517	-0.12813
H	7.95176	5.49600	-0.13025
H	8.14174	3.10190	-0.70002
H	6.57100	7.52499	0.20141
C	-6.73900	1.41363	0.64924
C	-6.22780	2.70154	0.45280
C	-7.86528	1.25425	1.46320
C	-6.82364	3.79473	1.07079
H	-5.36377	2.84267	-0.19046
C	-8.46242	2.35905	2.05979
H	-8.27466	0.26060	1.62117
C	-7.95182	3.64916	1.88505
H	-6.41151	4.78720	0.90099
H	-9.34032	2.21167	2.68513
C	-6.97688	-0.66882	-0.60157
C	-8.04791	-0.26451	-1.40506
C	-6.76047	-2.03804	-0.40811
C	-8.87976	-1.21016	-1.99427
H	-8.22969	0.79504	-1.56069
C	-7.58855	-2.97242	-1.01867
H	-5.94290	-2.36669	0.22731
C	-8.66481	-2.58111	-1.82239
H	-9.70944	-0.87237	-2.61150
H	-7.40384	-4.03139	-0.85100
C	-8.57818	4.83437	2.56564
H	-9.64960	4.68142	2.73026
H	-8.12100	5.01550	3.54722
H	-8.45048	5.74794	1.97575
C	-9.54372	-3.59913	-2.49432
H	-10.54764	-3.20278	-2.67696
H	-9.13074	-3.90039	-3.46592
H	-9.64001	-4.50648	-1.88889
N	-6.14261	0.29265	0.02047

Table S18. XYZ coordinates of the DFT-optimized structure of the *transoid* conformer of complex **3**.

Atom	X	Y	Z
Bi	-0.44080	-0.00008	-0.00008
N	0.37803	1.43121	2.00181
S	-0.38837	-2.34479	1.48932
C	-0.57207	2.50042	2.32205
H	-1.50268	2.05808	2.68874
H	-0.78471	3.08402	1.42272
H	-0.17046	3.16619	3.09991
C	1.65031	1.98510	1.50252
H	2.22747	2.41888	2.33292
H	1.39847	2.79785	0.81055
C	-1.72209	-5.00820	0.50605
H	-0.65359	-4.81718	0.53116
C	0.62067	0.60209	3.18734
H	1.34174	-0.18253	2.94884
H	-0.30963	0.12734	3.50540
H	1.01477	1.21865	4.00908
C	-4.10984	-7.70399	-0.43235
H	-3.41821	-8.46968	-0.77554
C	-4.41527	-2.01327	1.86415
H	-5.10452	-1.25125	2.22093
C	-2.59782	-3.97002	0.96594
C	-4.92218	-3.23163	1.39684
C	3.84041	0.94104	0.76391
H	4.38571	1.67798	1.34707
C	-6.32634	-3.51121	1.36514
H	-7.01226	-2.74188	1.71227
C	-5.48021	-7.93950	-0.44914
H	-5.85984	-8.89268	-0.80722
C	-2.11635	-2.72048	1.44008
C	-3.60048	-6.48081	0.02604
C	-2.19658	-6.20170	0.05869
H	-1.50813	-6.97151	-0.28240
C	-5.90470	-5.72634	0.45686
C	-4.00287	-4.22296	0.94878
C	-4.50100	-5.47444	0.47780
C	-3.04980	-1.77205	1.89044
H	-2.68614	-0.82741	2.28705
C	-6.36942	-6.96340	-0.01025
H	-7.43999	-7.15339	-0.02570
C	2.44950	0.94055	0.76178
C	-6.79899	-4.70370	0.91547
H	-7.86763	-4.90367	0.89655
C	1.75669	-0.00007	-0.00005
C	-0.57194	-2.50057	-2.32226
H	-1.50253	-2.05824	-2.68901
H	-0.78464	-3.08416	-1.42294
H	-0.17027	-3.16634	-3.10009
N	0.37812	-1.43134	-2.00196
C	0.62080	-0.60220	-3.18747
H	1.34185	0.18242	-2.94893
H	-0.30951	-0.12745	-3.50554
H	1.01494	-1.21873	-4.00920
C	1.65039	-1.98523	-1.50263
H	2.22759	-2.41898	-2.33301
H	1.39853	-2.79799	-0.81068
C	2.44954	-0.94067	-0.76185
C	3.84045	-0.94116	-0.76391
H	4.38578	-1.67810	-1.34705
S	-0.38835	2.34462	-1.48950
C	-1.72171	5.00827	-0.50642
H	-0.65323	4.81716	-0.53164
C	-4.10911	7.70442	0.43188
H	-3.41736	8.47011	0.77483
C	-4.41534	2.01332	-1.86359
H	-5.10470	1.25128	-2.22012

C	-2.59759	3.97009	-0.96601
C	-4.92208	3.23180	-1.39642
C	-6.32621	3.51150	-1.36454
H	-7.01225	2.74216	-1.71143
C	-5.47945	7.94004	0.44884
H	-5.85896	8.89331	0.80681
C	-2.11630	2.72044	-1.44003
C	-3.59991	6.48112	-0.02638
C	-2.19605	6.20189	-0.05919
H	-1.50748	6.97170	0.28168
C	-5.90426	5.72676	-0.45671
C	-4.00262	4.22314	-0.94867
C	-4.50058	5.47474	-0.47783
C	-3.04989	1.77199	-1.89006
H	-2.68636	0.82726	-2.28655
C	-6.36881	6.96393	0.01025
H	-7.43937	7.15401	0.02583
C	-6.79870	4.70411	-0.91501
H	-7.86732	4.90416	-0.89597
C	4.55140	-0.00006	0.00002
N	5.95472	-0.00006	0.00005
C	6.67508	-0.38678	-1.15721
C	6.67502	0.38664	1.15736
C	6.31239	0.09285	-2.42117
C	7.77752	-1.24102	-1.05337
C	7.77749	1.24084	1.05360
C	6.31224	-0.09300	2.42130
H	5.46863	0.77069	-2.51506
C	7.02861	-0.28992	-3.54895
C	8.49652	-1.60065	-2.18803
H	8.07247	-1.61739	-0.07804
H	8.07250	1.61721	0.07829
C	8.49644	1.60044	2.18830
C	7.02842	0.28974	3.54912
H	5.46847	-0.77082	2.51514
H	6.73077	0.09797	-4.52081
C	8.13442	-1.14238	-3.45841
H	9.35323	-2.26280	-2.08266
H	9.35317	2.26257	2.08298
C	8.13425	1.14216	3.45865
H	6.73051	-0.09816	4.52096
C	8.89083	-1.56671	-4.68645
C	8.89061	1.56646	4.68674
H	8.90861	-0.77223	-5.43990
H	9.92521	-1.83310	-4.44715
H	8.42759	-2.44550	-5.15380
H	8.42737	2.44527	5.15407
H	9.92501	1.83282	4.44750
H	8.90831	0.77198	5.44019

Table S19. XYZ coordinates of the DFT-optimized structure of complex **4**.

Atom	X	Y	Z
Bi	-0.67793	-0.00030	0.00087
S	-0.63881	2.12251	1.79885
O	-4.26053	5.46808	0.58221
C	1.51633	-0.00064	0.00027
O	-5.65123	7.08237	-0.01448
N	0.12670	-1.44645	1.99058
C	-0.80488	-1.40860	3.12220
H	-0.98870	-0.36884	3.40538
H	-0.39839	-1.95487	3.98559
H	-1.74942	-1.87521	2.82826
C	-4.01391	4.21049	1.03980
C	-6.41944	3.78937	1.19391
N	0.12609	1.44563	-1.98937
C	-4.70270	2.04965	1.82328
H	-5.48262	1.34176	2.08785
C	-7.56728	2.88875	1.52574
H	-7.53095	2.58111	2.57745
H	-7.53466	1.97448	0.92144
H	-8.52230	3.38745	1.34536
C	3.60013	-0.44376	1.12789
H	4.14502	-0.77642	2.00699
C	-6.62257	5.05112	0.73380
H	-7.62091	5.45001	0.59016
C	-3.37740	1.67995	1.96201
H	-3.13155	0.69471	2.34716
C	-5.05955	3.32742	1.35900
C	0.33496	-2.83337	1.55794
H	-0.61297	-3.26259	1.22507
H	0.73355	-3.43543	2.38782
H	1.03686	-2.85965	0.72229
C	-2.33491	2.57447	1.63181
C	-5.53906	5.94900	0.40379
C	3.59966	0.44250	-1.12820
H	4.14420	0.77519	-2.00750
C	1.41546	-0.82690	2.35230
H	1.98925	-1.50213	3.00462
H	1.18938	0.07570	2.93286
C	-2.67775	3.84877	1.16648
H	-1.91032	4.56951	0.90306
C	0.33494	2.83251	-1.55688
H	-0.61270	3.26199	-1.22353
H	0.73326	3.43443	-2.38699
H	1.03727	2.85862	-0.72159
C	2.20942	-0.44092	1.12754
C	1.41449	0.82571	-2.35168
H	1.18787	-0.07689	-2.93204
H	1.98813	1.50073	-3.00436
C	2.20896	0.43964	-1.12728
S	-0.64056	-2.12280	-1.79740
O	-4.26403	-5.46809	-0.58552
C	-4.70445	-2.04726	-1.82055
H	-5.48401	-1.33844	-2.08370
O	-5.65555	-7.08259	0.00871
C	-2.33693	-2.57386	-1.63065
C	-4.01677	-4.20987	-1.04099
C	-6.42209	-3.78700	-1.19368
C	-5.06196	-3.32560	-1.35836
C	-2.68043	-3.84874	-1.16739
H	-1.91338	-4.57039	-0.90538
C	-3.37897	-1.67814	-1.95905
H	-3.13266	-0.69239	-2.34260
C	-5.54281	-5.94856	-0.40761
C	-6.62586	-5.04943	-0.73574
H	-7.62441	-5.44797	-0.59253
C	-7.56945	-2.88506	-1.52354

H	-7.53600	-1.97185	-0.91768
H	-8.52474	-3.38344	-1.34365
H	-7.53331	-2.57563	-2.57474
C	-0.80600	1.40802	-3.12058
H	-0.99037	0.36830	-3.40356
H	-0.39965	1.95402	-3.98420
H	-1.75021	1.87507	-2.82631
C	4.31136	-0.00060	-0.00028
N	5.71358	-0.00050	-0.00056
C	6.43534	-0.97735	0.73001
C	6.43487	0.97656	-0.73134
C	6.07749	-2.32889	0.66754
C	7.53524	-0.60805	1.51078
C	7.53447	0.60753	-1.51264
C	6.07682	2.32803	-0.66855
H	5.23600	-2.63250	0.05115
C	6.79613	-3.27855	1.38405
C	8.25680	-1.57130	2.20715
H	7.82617	0.43713	1.56442
H	7.82556	-0.43759	-1.56652
C	8.25556	1.57098	-2.20923
C	6.79497	3.27789	-1.38528
H	5.23555	2.63143	-0.05175
H	6.50243	-4.32401	1.31814
C	7.89952	-2.92260	2.16716
H	9.11164	-1.26178	2.80456
H	9.11017	1.26167	-2.80706
C	7.89805	2.92221	-2.16894
H	6.50111	4.32329	-1.31913
C	8.65868	-3.95398	2.95464
C	8.65667	3.95381	-2.95665
H	8.66156	-4.92352	2.44572
H	9.69784	-3.64972	3.11539
H	8.20788	-4.10860	3.94365
H	8.20530	4.10856	-3.94539
H	9.69577	3.64969	-3.11803
H	8.65973	4.92326	-2.44756

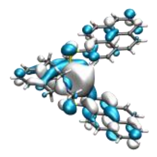

LUMO+3 – 218a

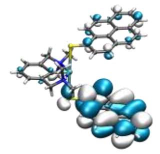

LUMO+2 – 217a

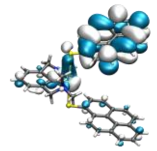

LUMO+1 – 216a

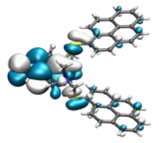

LUMO – 215a

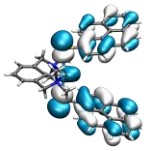

HOMO – 214a

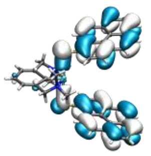

HOMO-1 – 213a

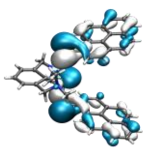

HOMO-2 – 212a

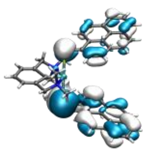

HOMO-2 – 211a

Figure S39. pbe0/SARC-ZORA-TZVP-calculated molecular orbitals of the ground state S_0 of monomeric **1** at the structure parameters taken from the SXRD-measurement.

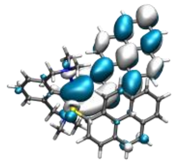		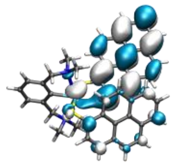
α-LUSO+2 – 218a		β-LUSO+4
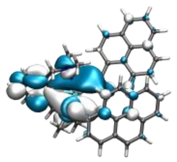		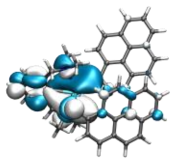
α-LUSO+1 – 217a		β-LUSO+3
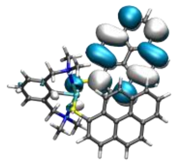		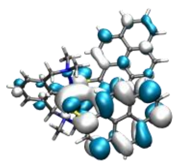
α-LUSO – 216a		β-LUSO+2
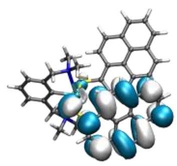		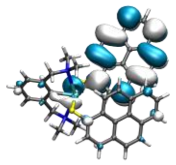
**α-HOSO – 215a**		β-LUSO+1
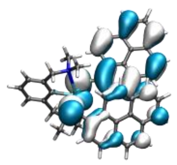		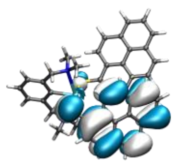
**α-HOSO-1 – 214a**		β-LUSO
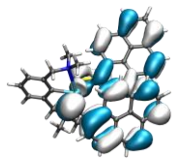		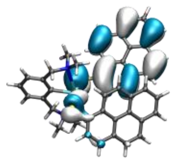
**α-HOSO-2 – 213a**		**β-HOSO**
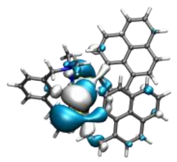		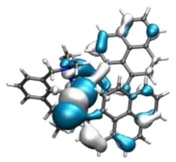
**α-HOSO-3 – 212a**		**β-HOSO-1**
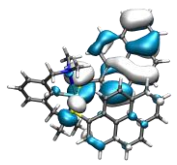		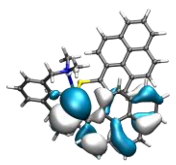
**α-HOSO-3 – 211a**		**β-HOSO-1**

Figure S40. pbe0/SARC-ZORA-TZVP-optimized molecular orbitals of the geometry-optimized triplet state T_1 of monomeric **1** with CPCM(CH_2Cl_2) correction.

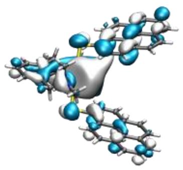		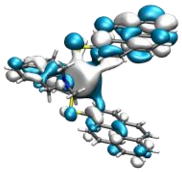
α-LUSO+2 – 218a		β-LUSO+4
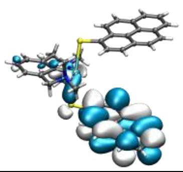		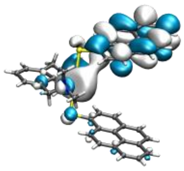
α-LUSO+1 – 217a		β-LUSO+3
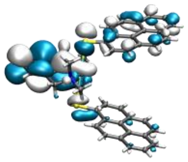		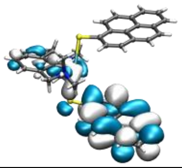
α-LUSO – 216a		β-LUSO+2
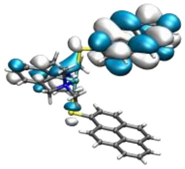		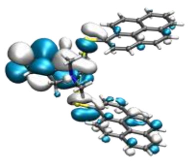
**α-HOSO – 215a**		β-LUSO+1
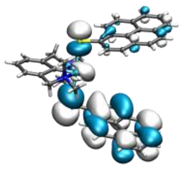		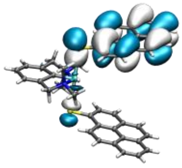
**α-HOSO-1 – 214a**		β-LUSO
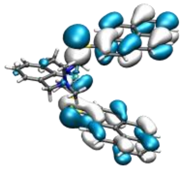		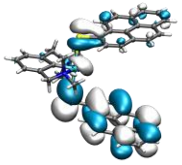
**α-HOSO-2 – 213a**		**β-HOSO**
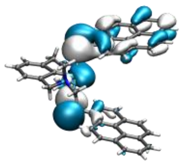		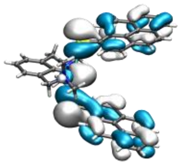
**α-HOSO-3 – 212a**		**β-HOSO-1**
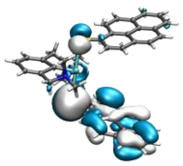		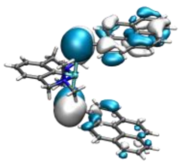
**α-HOSO-4 – 211a**		**β-HOSO-2**

Figure S41. pbe0/SARC-ZORA-TZVP-calculated molecular orbitals of the triplet state T_1 of monomeric **1** at the structure taken from the SXRD-measurement.

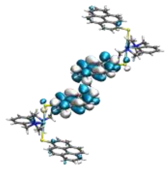		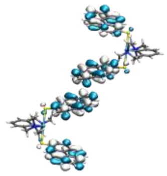
α-LUSO+2 – 433a		β-LUSO+4
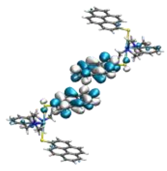		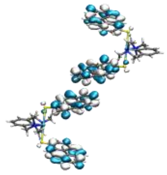
α-LUSO+1 – 432a		β-LUSO+3
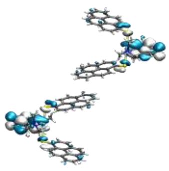		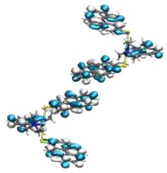
α-LUSO – 431a		β-LUSO+2
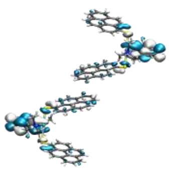		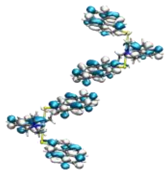
**α-HOSO – 430a**		β-LUSO+1
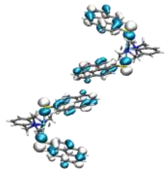		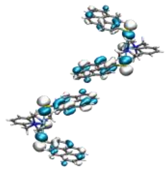
**α-HOSO-1 – 429a**		β-LUSO
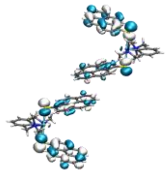		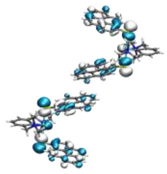
**α-HOSO-2 – 428a**		**β-HOSO**
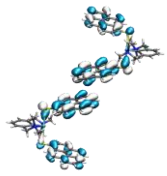		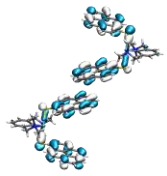
**α-HOSO-3 – 427a**		**β-HOSO-1**
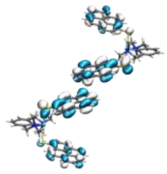		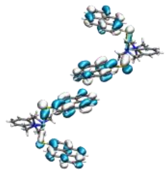
**α-HOSO-3 – 426a**		**β-HOSO-2**

Figure S42. pbe0/SARC-ZORA-TZVP-calculated molecular orbitals of the triplet state T_1 of **dimer1**. Structure parameters were taken from the SXRD-measurement.

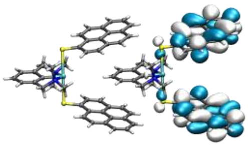		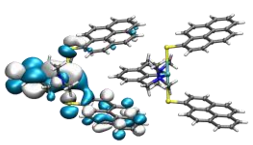
α-LUSO+2 – 433a		β-LUSO+4
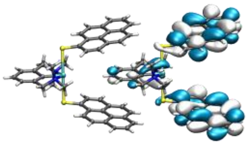		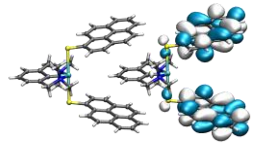
α-LUSO+1 – 432a		β-LUSO+3
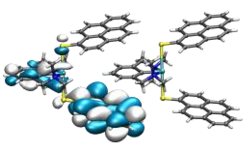		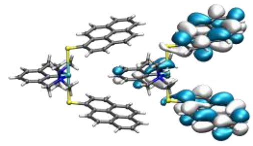
α-LUSO – 431a		β-LUSO+2
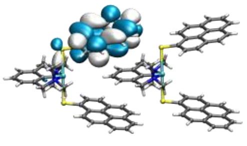		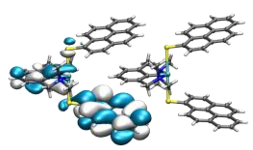
**α-HOSO – 430a**		β-LUSO+1
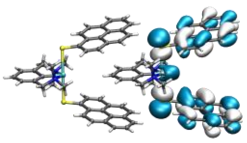		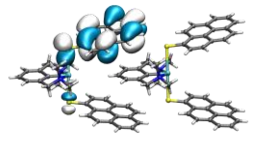
**α-HOSO-1 – 429a**		β-LUSO
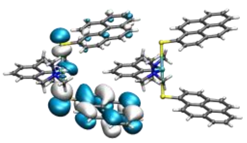		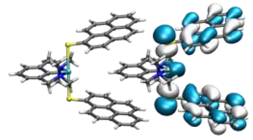
**α-HOSO-2 – 428a**		**β-HOSO**
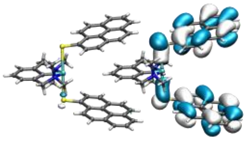		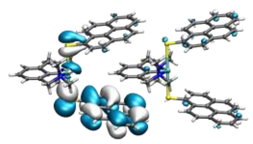
**α-HOSO-3 – 427a**		**β-HOSO-1**
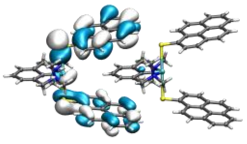		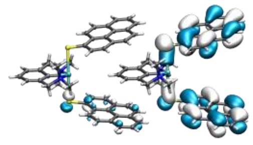
**α-HOSO-3 – 426a**		**β-HOSO-2**

Figure S43. pbe0/SARC-ZORA-TZVP-calculated molecular orbitals of the triplet state T_1 of **dimer2**. Structure parameters were taken from the SXRD-measurement.

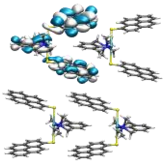		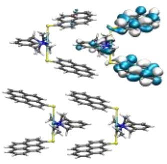
α-LUSO+2 – 863a		β-LUSO+4
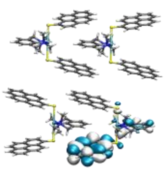		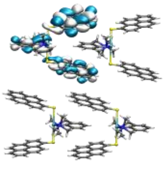
α-LUSO+1 – 862a		β-LUSO+3
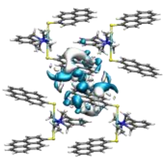		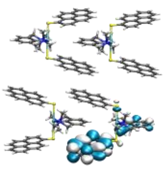
α-LUSO – 861a		β-LUSO+2
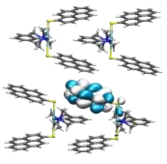		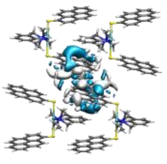
**α-HOSO – 860a**		β-LUSO+1
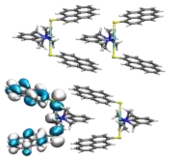		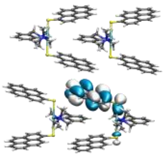
**α-HOSO-1 – 859a**		β-LUSO
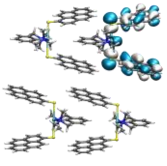		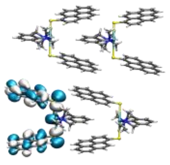
**α-HOSO-2 – 858a**		**β-HOSO**
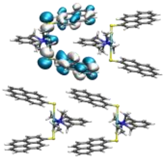		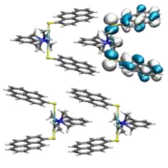
**α-HOSO-3 – 857a**		**β-HOSO-1**
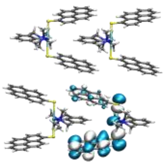		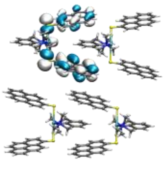
**α-HOSO-3 – 856a**		**β-HOSO-2**

Figure S44. pbe0/SARC-ZORA-TZVP-calculated molecular orbitals of the triplet state T_1 of the **tetramer** model. Structure parameters were taken from the SXRD-measurement.

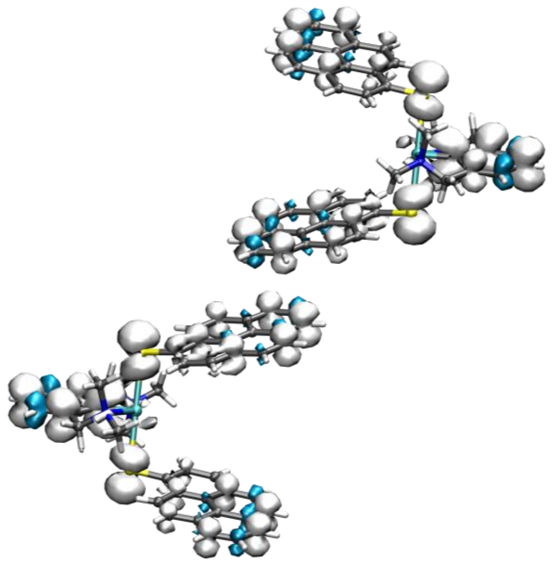

SXRD-dimer1 T_1 spin density

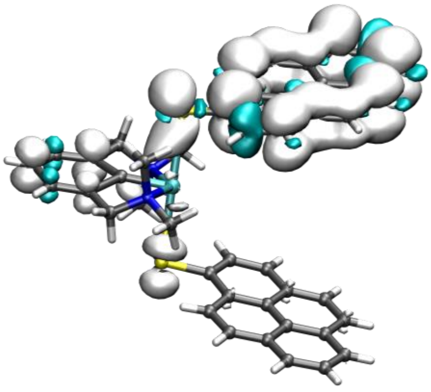

SXRD-monomer T_1 spin density

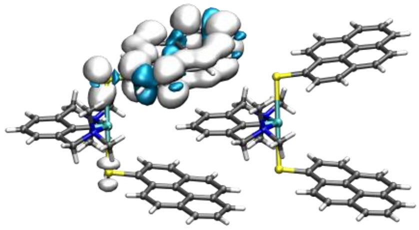

SXRD-dimer2 T_1 spin density

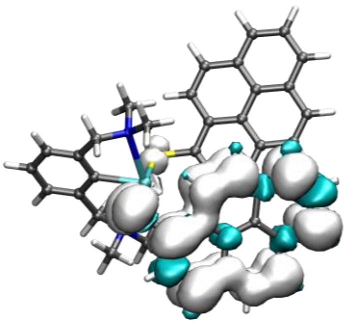

geometry-optimized monomer T_1 spin density

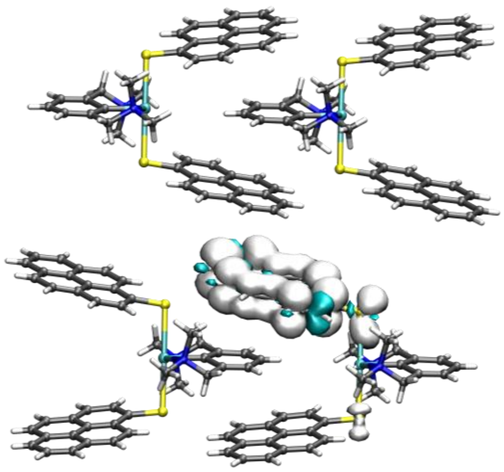

SXRD-tetramer T_1 spin density

Figure S45. Triplet state spin densities of **dimer1**, **dimer2**, **tetramer**, and monomeric **1** with structure parameters taken from the X-ray experiment (SXRD), and of the geometry-optimized monomer **1**.

Table S20. Energies of singlet and triplet states of geometry-optimized monomer **1**.

State	au	eV	cm^{-1}	nm	State	au	eV	cm^{-1}	nm
S1	0.101762	2.769	22334.2	448	T1	0.067475	1.836	14809.00	675
S2	0.111445	3.033	24459.3	409	T2	0.068959	1.876	15134.80	661
S3	0.117373	3.194	25760.4	388	T3	0.105183	2.862	23085.00	433
S4	0.118534	3.225	26015.3	384	T4	0.109977	2.993	24137.20	414
S5	0.122841	3.343	26960.4	371	T5	0.112762	3.068	24748.30	404
S6	0.127793	3.477	28047.4	357	T6	0.114402	3.113	25108.40	398
S7	0.128891	3.507	28288.2	354	T7	0.115294	3.137	25304.20	395
S8	0.129257	3.517	28368.7	353	T8	0.121108	3.296	26580.10	376
S9	0.133762	3.64	29357.4	341	T9	0.122652	3.338	26918.90	371
S10	0.135557	3.689	29751.3	336	T10	0.124376	3.384	27297.40	366
S11	0.1388	3.777	30463.2	328	T11	0.124767	3.395	27383.20	365
S12	0.142587	3.88	31294.3	320	T12	0.12571	3.421	27590.10	362
S13	0.143774	3.912	31554.9	317	T13	0.126539	3.443	27772.20	360
S14	0.145144	3.95	31855.4	314	T14	0.129311	3.519	28380.40	352
S15	0.146289	3.981	32106.7	311	T15	0.129963	3.536	28523.60	351
S16	0.147066	4.002	32277.4	310	T16	0.133079	3.621	29207.40	342
S17	0.148676	4.046	32630.6	306	T17	0.134223	3.652	29458.60	339
S18	0.150085	4.084	32939.8	304	T18	0.135691	3.692	29780.80	336
S19	0.151514	4.123	33253.4	301	T19	0.137133	3.732	30097.30	332
S20	0.153503	4.177	33689.9	297	T20	0.138721	3.775	30445.70	328
S21	0.154425	4.202	33892.4	295	T21	0.139159	3.787	30542.00	327
S22	0.156894	4.269	34434.2	290	T22	0.1426	3.88	31297.00	320
S23	0.159433	4.338	34991.6	286	T23	0.144332	3.927	31677.30	316
S24	0.159473	4.339	35000.3	286	T24	0.146773	3.994	32212.90	310
S25	0.161275	4.389	35395.7	283	T25	0.147623	4.017	32399.50	309

Table S21. Energies of singlet and triplet states of **1** with structure parameters taken from the SXRD-measurement.

State	au	eV	cm^{-1}	nm	State	au	eV	cm^{-1}	nm
S1	0.07878	2.14	17291	578	T1	0.06042	1.64	13261	754
S2	0.09412	2.56	20658	484	T2	0.06311	1.72	13852	722
S3	0.10381	2.83	22784	439	T3	0.0732	1.99	16067	622
S4	0.10831	2.95	23772	421	T4	0.07981	2.17	17517	571
S5	0.10886	2.96	23892	419	T5	0.09467	2.58	20777	481
S6	0.11719	3.19	25721	389	T6	0.10214	2.78	22417	446
S7	0.11932	3.25	26188	382	T7	0.1054	2.87	23133	432
S8	0.12063	3.28	26476	378	T8	0.10924	2.97	23975	417
S9	0.12169	3.31	26708	374	T9	0.11055	3.01	24262	412
S10	0.12443	3.39	27308	366	T10	0.11536	3.14	25318	395
S11	0.12911	3.51	28336	353	T11	0.1182	3.22	25941	385
S12	0.13271	3.61	29127	343	T12	0.11959	3.25	26248	381
S13	0.13401	3.65	29411	340	T13	0.1201	3.27	26358	379
S14	0.13498	3.67	29625	338	T14	0.12069	3.28	26489	378
S15	0.13698	3.73	30064	333	T15	0.12313	3.35	27024	370
S16	0.13832	3.76	30358	329	T16	0.12699	3.46	27871	359
S17	0.14079	3.83	30899	324	T17	0.12826	3.49	28150	355
S18	0.14425	3.93	31660	316	T18	0.13131	3.57	28819	347
S19	0.14633	3.98	32115	311	T19	0.1318	3.59	28926	346
S20	0.14705	4	32273	310	T20	0.13257	3.61	29097	344
S21	0.14731	4.01	32330	309	T21	0.13301	3.62	29191	343
S22	0.14835	4.04	32559	307	T22	0.13461	3.66	29544	338
S23	0.1492	4.06	32745	305	T23	0.13508	3.68	29646	337
S24	0.15031	4.09	32990	303	T24	0.1378	3.75	30243	331
S25	0.15132	4.12	33211	301	T25	0.13809	3.76	30307	330

Table S22. Energies of singlet and triplet states of **dimer1**; structure parameters are taken from the SXRD-measurement.

State	au	eV	cm^{-1}	nm	State	au	eV	cm^{-1}	nm
S1	0.077883	2.119	17093.3	585	T1	0.060372	1.643	13250.2	755
S2	0.078489	2.136	17226.4	581	T2	0.060373	1.643	13250.3	755
S3	0.09192	2.501	20174.2	496	T3	0.062939	1.713	13813.5	724
S4	0.091938	2.502	20178	496	T4	0.062947	1.713	13815.3	724
S5	0.093549	2.546	20531.5	487	T5	0.07286	1.983	15990.9	625
S6	0.093594	2.547	20541.4	487	T6	0.072868	1.983	15992.6	625
S7	0.103374	2.813	22687.9	441	T7	0.079566	2.165	17462.7	573
S8	0.104476	2.843	22929.9	436	T8	0.079568	2.165	17463.1	573
S9	0.105095	2.86	23065.7	434	T9	0.092074	2.505	20208	495
S10	0.105097	2.86	23066.1	434	T10	0.092089	2.506	20211.3	495
S11	0.108232	2.945	23754.1	421	T11	0.094267	2.565	20689.2	483
S12	0.108265	2.946	23761.5	421	T12	0.094283	2.566	20692.8	483
S13	0.108452	2.951	23802.4	420	T13	0.10168	2.767	22316.1	448
S14	0.108644	2.956	23844.7	419	T14	0.10168	2.767	22316.2	448
S15	0.111228	3.027	24411.8	410	T15	0.105082	2.859	23062.9	434
S16	0.111444	3.033	24459.2	409	T16	0.105094	2.86	23065.4	434
S17	0.116809	3.179	25636.6	390	T17	0.105635	2.874	23184.2	431
S18	0.116817	3.179	25638.3	390	T18	0.10566	2.875	23189.7	431
S19	0.117019	3.184	25682.8	389	T19	0.108632	2.956	23842.1	419
S20	0.11711	3.187	25702.7	389	T20	0.108642	2.956	23844.1	419
S21	0.119003	3.238	26118.1	383	T21	0.110749	3.014	24306.6	411
S22	0.119053	3.24	26129.2	383	T22	0.11083	3.016	24324.3	411
S23	0.120506	3.279	26447.9	378	T23	0.111322	3.029	24432.3	409
S24	0.120617	3.282	26472.3	378	T24	0.111333	3.03	24434.7	409
S25	0.120898	3.29	26534.1	377	T25	0.115148	3.133	25272	396

Table S23. Energies of singlet and triplet states of **dimer2**; structure parameters are taken from the SXRD-measurement.

State	au	eV	cm^{-1}	nm	State	au	eV	cm^{-1}	nm
S1	0.098497	2.68	21617.6	463	T1	0.061394	1.671	13474.5	742
S2	0.101044	2.75	22176.5	451	T2	0.061721	1.68	13546.2	738
S3	0.102024	2.776	22391.7	447	T3	0.063044	1.716	13836.6	723
S4	0.105696	2.876	23197.5	431	T4	0.063544	1.729	13946.2	717
S5	0.106115	2.888	23289.5	429	T5	0.096558	2.627	21192	472
S6	0.10794	2.937	23690	422	T6	0.100504	2.735	22058.2	453
S7	0.108444	2.951	23800.7	420	T7	0.102221	2.782	22434.8	446
S8	0.112335	3.057	24654.7	406	T8	0.105011	2.857	23047.2	434
S9	0.112474	3.061	24685.3	405	T9	0.105847	2.88	23230.7	430
S10	0.115388	3.14	25324.7	395	T10	0.106908	2.909	23463.6	426
S11	0.11725	3.191	25733.5	389	T11	0.107071	2.914	23499.4	426
S12	0.117382	3.194	25762.5	388	T12	0.108713	2.958	23859.7	419
S13	0.118097	3.214	25919.4	386	T13	0.11074	3.013	24304.7	411
S14	0.119215	3.244	26164.6	382	T14	0.112283	3.055	24643.4	406
S15	0.119442	3.25	26214.4	381	T15	0.112628	3.065	24719	405
S16	0.120873	3.289	26528.5	377	T16	0.114832	3.125	25202.8	397
S17	0.121084	3.295	26575	376	T17	0.116301	3.165	25525.1	392
S18	0.121309	3.301	26624.4	376	T18	0.116646	3.174	25600.7	391
S19	0.122919	3.345	26977.7	371	T19	0.117467	3.196	25781.1	388
S20	0.124533	3.389	27331.8	366	T20	0.117932	3.209	25883	386
S21	0.125203	3.407	27478.9	364	T21	0.118573	3.227	26023.8	384
S22	0.126654	3.446	27797.4	360	T22	0.118608	3.227	26031.5	384
S23	0.128167	3.488	28129.4	355	T23	0.119432	3.25	26212.4	381
S24	0.129255	3.517	28368.2	353	T24	0.119893	3.262	26313.4	380
S25	0.129518	3.524	28425.9	352	T25	0.12007	3.267	26352.4	379

Table S24. pbe0/SARC-ZORA-TZVP-calculated transition states of geometry-optimized monomeric **1**.

State	E (cm^{-1})	λ (nm)	fosc	Singlet percentage (%)
1	15044.2	664.7	0.000013138	0
2	15045.1	664.7	0.000259677	0
3	15045.9	664.6	0.000059060	0
4	15405.3	649.1	0.000131105	0
5	15410.0	648.9	0.000049017	0
6	15410.2	648.9	0.000129839	0
7	21251.1	470.6	0.000373197	0
8	21339.2	468.6	0.016725027	8
9	21440.8	466.4	0.086009662	37
10	21968.4	455.2	0.143717380	58
11	24208.4	413.1	0.008495023	11
12	24288.2	411.7	0.000146691	0
13	24291.4	411.7	0.000127969	0
14	24741.5	404.2	0.086637896	74
15	25072.3	398.8	0.022015114	17
16	25194.1	396.9	0.013669523	1
17	25239.0	396.2	0.001534055	7
18	25337.3	394.7	0.543902034	54
19	25369.5	394.2	0.001677980	13
20	25412.1	393.5	0.000712017	0
21	25414.5	393.5	0.023770856	3
22	25536.9	391.6	0.026376541	6
23	25553.0	391.3	0.000067896	0
24	25588.1	390.8	0.098466959	13
25	25743.6	388.4	0.007447009	23
26	26553.4	376.6	0.009239418	3
27	26565.6	376.4	0.001630265	0
28	26646.7	375.3	0.062219384	24
29	26856.8	372.3	0.001398767	0
30	26886.9	371.9	0.009752853	2
31	27049.0	369.7	0.026608334	28
32	27263.4	366.8	0.001608478	6
33	27281.2	366.6	0.002407154	3
34	27284.8	366.5	0.000090695	0
35	27364.0	365.4	0.068825452	54
36	27500.7	363.6	0.003701538	42
37	27628.8	361.9	0.001739575	0
38	27633.3	361.9	0.000995782	0
39	27655.2	361.6	0.003223192	2
40	27797.4	359.7	0.010513180	13
41	27846.2	359.1	0.000315774	0
42	27849.5	359.1	0.000344685	0
43	28056.2	356.4	0.002277993	3
44	28074.4	356.2	0.000140270	0
45	28104.5	355.8	0.000651831	3

46	28472.2	351.2	0.009987164	72
47	28500.3	350.9	0.023465162	61
48	28697.5	348.5	0.014767732	8
49	28711.3	348.3	0.001039873	0
50	28748.3	347.8	0.067569001	57
51	29171.0	342.8	0.013536051	47
52	29482.7	339.2	0.000122299	0
53	29494.7	339.0	0.001212320	0
54	29580.6	338.1	0.006059130	7
55	29664.3	337.1	0.001778010	10
56	29701.5	336.7	0.000355915	0
57	29753.3	336.1	0.001014835	3
58	29849.7	335.0	0.000167835	1
59	29856.2	334.9	0.000232659	0
60	29893.4	334.5	0.000490918	23
61	30059.9	332.7	0.005725606	73
62	30411.0	328.8	0.011496710	28
63	30520.4	327.6	0.001306359	0
64	30530.4	327.5	0.000161205	0
65	30618.2	326.6	0.004683733	8
66	30666.4	326.1	0.001114709	0
67	30676.2	326.0	0.001349206	0
68	30730.9	325.4	0.000748003	0
69	30844.9	324.2	0.001547853	0
70	30856.1	324.1	0.002122911	1
71	30942.1	323.2	0.015601346	17
72	31020.3	322.4	0.000408030	0
73	31044.2	322.1	0.005825002	6
74	31239.6	320.1	0.015357981	73
75	31496.3	317.5	0.000530996	22
76	31940.9	313.1	0.000378578	2
77	31946.3	313.0	0.004119699	2
78	32074.7	311.8	0.044188281	64
79	32095.7	311.6	0.035835153	48
80	32248.3	310.1	0.011432277	72
81	32337.6	309.2	0.000224064	0
82	32361.8	309.0	0.001440646	5
83	32387.2	308.8	0.009813717	74
84	32493.4	307.8	0.047502379	31
85	32601.4	306.7	0.000126313	18
86	32616.3	306.6	0.001230143	0
87	32637.1	306.4	0.002870984	32
88	32665.1	306.1	0.066121455	62
89	32979.8	303.2	0.003409143	8
90	32993.4	303.1	0.004039403	3
91	33041.2	302.7	0.030444280	81
92	33240.8	300.8	0.038538986	42
93	33276.9	300.5	0.035356799	92
94	33558.2	298.0	0.000941220	99

95	34293.4	291.6	0.004216241	93
96	34690.7	288.3	0.091531217	90
97	34807.9	287.3	0.153875539	96
98	35440.0	282.2	0.121390229	95
99	35814.4	279.2	0.052296422	91
100	36016.2	277.7	0.024693723	91

Table S25. pbe0/SARC-ZORA-TZVP-calculated transition states of **1** with structure parameters taken from the SXRD-measurement.

State	E (cm^{-1})	λ (nm)	fosc	Singlet percentage (%)
1	13407.4	745.9	0.000002897	0
2	13409.7	745.7	0.000041927	0
3	13412.5	745.6	0.000581383	0
4	14050.0	711.7	0.000007950	0
5	14052.5	711.6	0.000034599	0
6	14053.7	711.6	0.000055522	0
7	15965.8	626.3	0.000060352	0
8	15992.5	625.3	0.001039394	1
9	16001.7	624.9	0.000285147	0
10	17276.9	578.8	0.145892858	91
11	17721.3	564.3	0.002433197	0
12	17723.0	564.2	0.000821442	0
13	17737.7	563.8	0.005304883	4
14	20602.3	485.4	0.095371678	91
15	20780.7	481.2	0.000139957	0
16	20784.5	481.1	0.000253112	0
17	20804.5	480.7	0.016931972	4
18	22284.1	448.8	0.000650162	2
19	22431.7	445.8	0.000728909	0
20	22466.0	445.1	0.000112084	0
21	22923.1	436.2	0.152728715	71
22	23382.9	427.7	0.041367655	26
23	23457.5	426.3	0.000556874	0
24	23480.9	425.9	0.003894329	0
25	23936.9	417.8	0.071915950	92
26	24071.5	415.4	0.180647498	89
27	24200.2	413.2	0.000160564	2
28	24202.4	413.2	0.000202834	0
29	24206.2	413.1	0.000136929	0
30	24565.4	407.1	0.000889738	2
31	24569.0	407.0	0.003198605	6
32	24573.1	406.9	0.001147215	3
33	25488.0	392.3	0.001193224	12
34	25537.5	391.6	0.000127213	0
35	25552.2	391.4	0.000066343	0
36	25758.8	388.2	0.000142664	41

37	26115.9	382.9	0.000559476	0
38	26120.8	382.8	0.000982651	2
39	26152.6	382.4	0.001118806	5
40	26338.1	379.7	0.013263673	41
41	26381.1	379.1	0.000064440	0
42	26422.9	378.5	0.019309536	49
43	26537.0	376.8	0.015374032	17
44	26618.6	375.7	0.022839420	34
45	26683.4	374.8	0.002165544	0
46	26694.0	374.6	0.004183313	1
47	26742.1	373.9	0.026347538	20
48	26847.6	372.5	0.119128602	46
49	26896.4	371.8	0.001600055	0
50	26917.1	371.5	0.037205186	26
51	27025.0	370.0	0.111267865	69
52	27338.6	365.8	0.002276437	1
53	27345.6	365.7	0.000241852	0
54	27349.5	365.6	0.005925611	8
55	27577.4	362.6	0.072299800	94
56	28118.2	355.6	0.000923036	0
57	28135.6	355.4	0.000184221	0
58	28137.4	355.4	0.000126009	0
59	28373.8	352.4	0.005790241	23
60	28442.4	351.6	0.000081021	0
61	28448.4	351.5	0.000002128	0
62	28610.5	349.5	0.019437361	71
63	29067.3	344.0	0.000161323	0
64	29072.9	344.0	0.000238631	0
65	29088.2	343.8	0.000405723	4
66	29212.6	342.3	0.004569801	3
67	29250.6	341.9	0.000820901	0
68	29258.7	341.8	0.000622954	0
69	29329.3	341.0	0.007397510	41
70	29368.0	340.5	0.000067097	0
71	29374.6	340.4	0.000366553	0
72	29399.3	340.1	0.001334905	12
73	29442.8	339.6	0.000325562	1
74	29447.4	339.6	0.000877671	0
75	29504.9	338.9	0.006139027	31
76	29711.6	336.6	0.004754209	86
77	29861.8	334.9	0.001911398	50
78	29873.9	334.7	0.000509734	3
79	29878.0	334.7	0.000234713	21
80	29880.0	334.7	0.000268870	13
81	29945.3	333.9	0.001543273	22
82	29978.1	333.6	0.001058703	0
83	29993.4	333.4	0.001237492	4
84	30488.2	328.0	0.000645080	2
85	30495.4	327.9	0.000028964	2

86	30499.1	327.9	0.000357639	2
87	30533.7	327.5	0.002645585	69
88	30593.0	326.9	0.000952465	84
89	30604.8	326.7	0.000140034	7
90	30607.6	326.7	0.000278288	4
91	30649.0	326.3	0.000057370	17
92	31170.5	320.8	0.043902786	97
93	31942.9	313.1	0.029876976	98
94	32440.9	308.3	0.048095154	98
95	32587.1	306.9	0.028211582	98
96	32656.0	306.2	0.055845303	95
97	32831.9	304.6	0.009474209	98
98	33023.0	302.8	0.003517112	99
99	33344.9	299.9	0.010839391	97
100	33663.6	297.1	0.029118687	95

Table S26. pbe0/SARC-ZORA-TZVP-calculated transition states of **dimer1**, with structure parameters taken from the SXRD-measurement.

State	E (cm^{-1})	λ (nm)	fosc	Singlet percentage (%)
1	13389.3	746.9	0.000064605	0
2	13389.8	746.8	0.000000003	0
3	13392.4	746.7	0.000147787	0
4	13393.0	746.7	0.000116503	0
5	13399.3	746.3	0.000000020	0
6	13407.0	745.9	0.000000005	0
7	14004.9	714.0	0.000038587	0
8	14005.3	714.0	0.000000057	0
9	14006.5	714.0	0.000027275	0
10	14006.8	713.9	0.000096781	0
11	14008.1	713.9	0.000000030	0
12	14009.6	713.8	0.000000025	0
13	15956.2	626.7	0.000648796	0
14	15959.3	626.6	0.000715434	0
15	15972.6	626.1	0.000000014	1
16	15973.6	626.0	0.002670297	1
17	15982.7	625.7	0.000000007	0
18	16032.1	623.7	0.000000018	0
19	17121.6	584.1	0.353591509	94
20	17281.2	578.7	0.000000616	91
21	17669.0	566.0	0.001745990	0
22	17675.1	565.8	0.000029658	0
23	17676.1	565.7	0.000000005	0
24	17678.6	565.7	0.000000001	0
25	17684.8	565.5	0.005642804	3
26	17692.7	565.2	0.000000085	5
27	20357.0	491.2	0.031662357	96

28	20363.8	491.1	0.000000381	97
29	20375.6	490.8	0.002666434	3
30	20380.7	490.7	0.000000022	2
31	20393.4	490.4	0.000002622	0
32	20393.6	490.4	0.000048357	0
33	20397.1	490.3	0.000000002	0
34	20397.2	490.3	0.000000003	0
35	20704.7	483.0	0.111116193	93
36	20720.7	482.6	0.000003446	94
37	20843.5	479.8	0.034862239	6
38	20875.3	479.0	0.000000078	0
39	20876.8	479.0	0.000079462	0
40	20878.3	479.0	0.000000677	0
41	20880.1	478.9	0.001244186	0
42	20898.3	478.5	0.000000005	5
43	22444.2	445.5	0.000845935	0
44	22449.6	445.4	0.001732934	0
45	22451.1	445.4	0.000001254	0
46	22500.5	444.4	0.000000031	0
47	22506.7	444.3	0.015177354	5
48	22513.5	444.2	0.000000069	2
49	22836.0	437.9	0.400311248	74
50	23040.8	434.0	0.000000094	64
51	23272.3	429.7	0.000000006	74
52	23276.1	429.6	0.000610936	72
53	23279.5	429.6	0.000000175	3
54	23280.3	429.5	0.001382274	2
55	23280.9	429.5	0.000000002	3
56	23281.9	429.5	0.000089673	6
57	23284.2	429.5	0.000000318	20
58	23284.8	429.5	0.001135242	21
59	23479.2	425.9	0.126413378	20
60	23518.1	425.2	0.000000466	31
61	23557.0	424.5	0.012069057	0
62	23563.6	424.4	0.002789328	0
63	23579.4	424.1	0.000000089	0
64	23629.8	423.2	0.000000026	4
65	23939.9	417.7	0.000203079	80
66	23940.3	417.7	0.085990425	79
67	23998.1	416.7	0.411718232	86
68	24056.5	415.7	0.000000056	82
69	24108.8	414.8	0.004983561	4
70	24114.4	414.7	0.002157632	0
71	24116.5	414.7	0.000000215	5
72	24128.3	414.5	0.000027194	1
73	24128.8	414.4	0.015406225	19
74	24145.3	414.2	0.000000607	23
75	24548.7	407.4	0.000479044	0
76	24548.8	407.4	0.000310934	0

77	24556.0	407.2	0.002790716	0
78	24574.1	406.9	0.000000003	2
79	24584.9	406.8	0.000000007	0
80	24587.0	406.7	0.000000012	0
81	24643.4	405.8	0.003027907	93
82	24667.6	405.4	0.000000090	54
83	24692.1	405.0	0.018333164	12
84	24737.9	404.2	0.000000424	46
85	24740.1	404.2	0.001015362	0
86	24741.3	404.2	0.000117223	0
87	24754.7	404.0	0.000000282	0
88	24769.0	403.7	0.000000136	3
89	25489.8	392.3	0.002874115	4
90	25572.4	391.0	0.000517255	0
91	25578.1	391.0	0.000143276	0
92	25868.4	386.6	0.014188142	99
93	25868.6	386.6	0.000116691	99
94	26232.0	381.2	0.000005867	92
95	26258.3	380.8	0.029040670	89
96	26346.4	379.6	0.121043664	98
97	26360.5	379.4	0.000001070	99
98	26698.3	374.6	0.205846038	98
99	26717.6	374.3	0.000005839	98
100	26823.9	372.8	0.429730955	98

Table S27. pbe0/SARC-ZORA-TZVP-calculated transition states of **dimer2**, with structure parameters taken from the SXRD-measurement..

State	E (cm^{-1})	λ (nm)	fosc	Singlet percentage (%)
1	13717.0	729.0	0.000092638	0
2	13718.7	728.9	0.000007224	0
3	13718.8	728.9	0.001024080	0
4	13769.7	726.2	0.000111299	0
5	13770.7	726.2	0.000008308	0
6	13770.8	726.2	0.001067919	0
7	14102.2	709.1	0.000023295	0
8	14104.5	709.0	0.000445292	0
9	14104.7	709.0	0.000719302	0
10	14183.1	705.1	0.000052401	0
11	14184.2	705.0	0.000477995	0
12	14184.4	705.0	0.000672870	0
13	21689.1	461.1	0.145215365	76
14	21892.0	456.8	0.493840764	81
15	22293.9	448.6	0.000547538	0
16	22321.6	448.0	0.000297848	0
17	22511.1	444.2	0.072164598	25
18	22569.4	443.1	0.000480003	0

19	22603.0	442.4	0.000432924	0
20	22780.8	439.0	0.052945146	18
21	23605.1	423.6	0.722322858	95
22	23687.6	422.2	0.001038393	6
23	23717.3	421.6	0.003519113	29
24	23718.9	421.6	0.000654680	61
25	23720.0	421.6	0.000307482	12
26	23746.6	421.1	0.079310759	88
27	23847.3	419.3	0.003412015	1
28	23873.1	418.9	0.000173432	0
29	23875.6	418.8	0.001019204	0
30	23947.7	417.6	0.001512766	1
31	23961.6	417.3	0.000279012	0
32	23963.3	417.3	0.001674320	0
33	24176.4	413.6	0.001653013	0
34	24248.3	412.4	0.000088740	0
35	24248.8	412.4	0.000375289	0
36	24314.4	411.3	0.012670613	96
37	24404.2	409.8	0.004015487	8
38	24406.8	409.7	0.000632989	5
39	24410.8	409.7	0.000321859	26
40	24412.7	409.6	0.000544000	60
41	24465.2	408.7	0.017314463	5
42	24567.3	407.0	0.012222528	3
43	24579.1	406.9	0.000197824	0
44	24582.1	406.8	0.002259243	0
45	24635.4	405.9	0.000015524	0
46	24637.0	405.9	0.001417992	0
47	25001.3	400.0	0.040479020	9
48	25115.6	398.2	0.000825396	0
49	25121.5	398.1	0.000673846	0
50	25147.3	397.7	0.006880059	20
51	25158.2	397.5	0.003292792	57
52	25161.5	397.4	0.000017935	2
53	25164.7	397.4	0.000463407	3
54	25178.3	397.2	0.008440257	22
55	25278.1	395.6	0.001159847	0
56	25280.7	395.6	0.000399186	0
57	25609.1	390.5	0.614119281	86
58	25748.6	388.4	0.021479551	23
59	25847.3	386.9	0.049335925	19
60	25853.6	386.8	0.001501476	0
61	25868.3	386.6	0.113844831	43
62	25932.5	385.6	0.062635952	46
63	26207.3	381.6	0.140366209	93
64	26266.4	380.7	0.025448939	43
65	26275.5	380.6	0.000659515	0
66	26280.1	380.5	0.001512362	2
67	26356.9	379.4	0.002818271	3

68	26445.6	378.1	0.000350844	0
69	26460.0	377.9	0.000615897	0
70	26493.5	377.5	0.122678262	75
71	26518.1	377.1	0.002237688	0
72	26524.3	377.0	0.004777465	1
73	26539.5	376.8	0.005345049	4
74	26555.8	376.6	0.034009236	16
75	26661.6	375.1	0.000455872	0
76	26669.0	375.0	0.000024284	0
77	26762.7	373.7	0.000149391	0
78	26772.4	373.5	0.000355460	2
79	26775.2	373.5	0.011348815	52
80	26800.8	373.1	0.006431828	43
81	26877.7	372.1	0.007465388	9
82	26912.2	371.6	0.001135249	0
83	26913.4	371.6	0.000426225	1
84	26929.5	371.3	0.007849338	6
85	26952.0	371.0	0.014320933	85
86	27021.9	370.1	0.013324263	17
87	27043.5	369.8	0.001930334	0
88	27075.5	369.3	0.009946838	21
89	27218.1	367.4	0.000956787	0
90	27228.2	367.3	0.000188433	0
91	27433.1	364.5	0.021827500	43
92	27597.9	362.3	0.006772760	97
93	27618.1	362.1	0.007671361	99
94	28161.5	355.1	0.043569572	95
95	28435.9	351.7	0.040205575	97
96	28531.2	350.5	0.023205679	98
97	28865.0	346.4	0.001657804	99
98	28952.1	345.4	0.003323211	97
99	29239.8	342.0	0.013635658	97
100	29290.2	341.4	0.000333556	96

Table S28. XYZ coordinates of pbe0/SARC-ZORA-TZVP geometry-optimized **1**.

Atom	X	Y	Z
Bi	1.68977155649344	-0.37595751591895	0.13916348567870
S	0.27084956756921	-1.98838768358724	-1.46168327959031
C	3.36673671757174	-1.81484598559568	0.06573734571360
N	3.11722244473839	0.13430278986772	-1.92817533512445
C	2.33493320240031	0.74205212534612	-3.00183609382016
H	1.49399387689355	0.09344737157869	-3.24921076271032
H	2.95220762008509	0.89004636080165	-3.89636717665174
H	1.95945305320852	1.71132009184946	-2.66988573164514
N	1.52735402267809	-1.94150015715787	2.19305948044983
C	5.11031362327099	-2.88863299164935	-1.17601693500859
H	5.67000561826605	-3.02158753085887	-2.09645869826134
C	4.23372656459765	1.00617181050505	-1.56372531252296
H	3.85139542593556	1.97326077217486	-1.24279433768412
H	4.89583025376770	1.14763230419027	-2.42703752298923
H	4.80096112697477	0.56305054549595	-0.74614544818299
C	4.72879498028919	-3.46700502027973	1.13644750224390
H	4.99062024342110	-4.05213036633737	2.01229592287251
C	5.43551083841930	-3.63109466029082	-0.04801647852393
H	6.24972670946161	-4.34568598260845	-0.09230262164363
C	3.62193671460306	-1.18657090988796	-2.32224961371811
H	4.43908670569348	-1.07723648604635	-3.04666767398214
H	2.80577323138341	-1.71467243632567	-2.82456366643969
C	0.78074764188488	-3.14799380767034	1.83761211285915
H	-0.24822829219092	-2.88405083284962	1.59607329095631
H	0.78550366109623	-3.85327041878524	2.67843900679607
H	1.22992516188922	-3.62333505627036	0.96657648077888
C	4.06263954708286	-1.97724210739791	-1.12312939129493
C	2.93198288027523	-2.27231449734817	2.46538889489333
H	3.37841922875266	-1.41014348314048	2.97093024006295
H	2.99226001962907	-3.12451944479712	3.15460538058156
C	3.68974011068063	-2.54622780047963	1.19846085992058
S	3.12196321694795	1.47382852552878	1.83579847341708
C	0.91625584701263	-1.30733315063549	3.35815653826895
H	1.47882009849438	-0.41167854492507	3.62217125029291
H	0.91353047065856	-1.99255493637992	4.21507023357080
H	-0.11372320018593	-1.03504933192435	3.12523186460037
C	2.24486068696310	2.77325553940053	1.11856169715791
C	0.79020639117881	2.84611086612897	1.21091302816177
C	2.91377278985142	3.75045618893462	0.35249339499548
C	0.09100726692796	3.79319113379009	0.40751597665862
C	0.05445572941622	2.00819531056993	2.03600813259145
C	2.24561775919191	4.68863248268156	-0.37936538762820
H	3.99801096045147	3.72537317555217	0.33278157177898
C	-1.32395422964972	3.82825883570994	0.42650450399285
C	0.80556678372292	4.71427522808911	-0.40774718751338
C	-1.34323710984177	2.04651882732293	2.06215625462182
H	0.58678912855262	1.32535027577153	2.68347619521173
H	2.78890214644315	5.41785719534934	-0.96992166235439
C	-2.05129787976351	2.93709056613841	1.26609985993021
C	-2.02444968913951	4.75686278953063	-0.39108183383589
C	0.09915696492112	5.61497289186705	-1.19347895033664
H	-1.88613607893828	1.37095293653033	2.71501126927913
C	-3.47992319949784	2.99726504292507	1.26807928060022
C	-3.45305508013552	4.77104349919599	-0.35846727753760
C	-1.29368572075617	5.63232575711337	-1.19337548409288
H	0.64702556462434	6.31662554496886	-1.81364455783559
C	-4.14786060162063	3.90275529661720	0.46557626186993
H	-4.02850118103435	2.31295757205250	1.90510497292980
H	-3.98098875845873	5.48129506154783	-0.98482021547235
H	-1.82521697296672	6.34231968957071	-1.81843043061417
H	-5.23208492953641	3.93010168356287	0.47841817923760
C	-1.28485582750867	-1.19172069196603	-1.27501535647882
C	-2.41605413383284	-1.91102668260700	-0.83088955786992
C	-1.42520966624393	0.16403322033658	-1.58397221990317
C	-3.66820048607288	-1.24813981560312	-0.73474159303851

C	-2.35702307378941	-3.29488418171271	-0.48300017080597
C	-2.63866663099596	0.81275335313589	-1.47185908725551
H	-0.56240589880400	0.71746591128051	-1.93645245854814
C	-4.82157937112020	-1.96195079019611	-0.31297885311543
C	-3.78152841306694	0.12619362472856	-1.06274217476566
C	-3.45500728658329	-3.97363449142073	-0.07498860505375
H	-1.39989933256493	-3.79611120627113	-0.55644603354440
H	-2.71120845077501	1.86838818806540	-1.71089848525154
C	-4.72752603830682	-3.33593524208989	0.02239033828791
C	-6.07685213217617	-1.30462038518408	-0.22975555350753
C	-5.05663779659693	0.76042510160753	-0.97709143735595
H	-3.38370198929887	-5.02479206139129	0.18518324467700
C	-5.87288222956303	-4.02122316900192	0.43342491995975
C	-7.19684438827757	-2.02743490957692	0.18559707487843
C	-6.15632475600155	0.07833476355964	-0.58023754167611
H	-5.12272024625532	1.81020734491184	-1.23932183700239
C	-7.09308696671754	-3.37038223293864	0.51382715275976
H	-5.79440461078688	-5.07283527741682	0.68835641732946
H	-8.15500283581200	-1.52237804509103	0.24751323814608
H	-7.12111731235090	0.57108963209024	-0.52128045880755
H	-7.97393135715384	-3.91512690636088	0.83480916198188

Table S29. XYZ coordinates of pbe0/SARC-ZORA-TZVP-calculated **1**, with structure parameters taken from the SXRD-measurement.

Atom	X	Y	Z
Bi	4.790733	6.261425	18.358981
S	4.823664	3.937144	19.847891
S	4.782717	8.602082	16.901849
N	3.998862	5.114782	16.216428
N	3.991550	7.442141	20.511312
C	8.716015	9.007568	15.788650
C	9.396711	3.180662	19.555535
C	8.791587	3.523295	20.836423
C	6.717067	8.542918	14.446652
H	5.777134	8.429315	14.367574
C	8.906408	8.620511	13.388208
C	1.912330	6.491193	19.553091
C	10.754827	2.955924	19.474869
H	11.140064	2.726051	18.637012
C	7.177981	9.124904	18.118204
H	6.678558	9.135381	18.925994
C	8.530944	9.362134	18.169537
H	8.922248	9.591196	19.003458
C	7.380707	3.718789	20.921978
C	6.539617	8.875665	16.947316
C	7.518614	8.468426	13.351542
H	7.109289	8.303132	12.508918
C	7.187182	3.364223	18.541092
H	6.644166	3.334618	17.760753
C	11.021124	3.406167	21.858200
C	11.516932	9.411200	15.969539
H	12.453657	9.565552	16.037055
C	10.910168	9.080703	14.683763
C	7.315109	8.794376	15.725095
C	0.529428	6.491352	19.535980
H	0.091082	6.684545	20.355015
C	1.895582	5.984815	17.147760
C	8.518077	3.126175	18.453092
H	8.883610	2.911549	17.602035
C	9.501875	8.892722	14.620207
C	9.630033	3.601465	21.951089
C	-0.200699	6.254192	18.519092
H	-1.149301	6.243433	18.575388
C	6.869866	3.990247	22.249310
H	5.940582	4.114738	22.367915
C	11.257587	3.858015	24.248864
H	11.809999	3.962053	25.015856
C	3.690902	8.864245	20.318201
H	4.524772	9.356914	20.170801
H	3.240569	9.213796	21.114943
H	3.106016	8.971144	19.538840
C	7.692862	4.074605	23.334643
H	7.320859	4.259036	24.188462
C	4.862620	7.278027	21.606422
H	5.034655	6.320507	21.746953
H	4.456011	7.656475	22.411353
H	5.711293	7.733822	21.421745
C	3.687470	3.664804	16.419316
H	4.499469	3.192102	16.696418
H	3.356421	3.281748	15.579968
H	3.001823	3.572073	17.113269
C	6.589151	3.662502	19.819535
C	2.726798	5.813251	15.923095
H	2.917950	6.702088	15.534135
H	2.210747	5.292920	15.254589
C	10.723534	9.480055	17.062205
H	11.126559	9.687380	17.897984
C	2.708582	6.696814	20.794868
H	2.927466	5.817566	21.194803

H	2.174242	7.207708	21.449489
C	11.703789	8.950316	13.512875
H	12.645429	9.073073	13.548393
C	0.526778	5.991580	17.208871
H	0.027759	5.828087	16.418412
C	4.918632	5.254471	15.091984
H	5.203106	6.188923	15.017307
H	4.466403	4.983548	14.265372
H	5.701626	4.684294	15.237869
C	9.911106	4.014437	24.358864
H	9.531147	4.207275	25.206474
C	11.066381	8.644669	12.307765
H	11.586044	8.537530	11.519530
C	11.840433	3.541449	23.014421
H	12.779764	3.422661	22.947370
C	9.064329	3.891995	23.205087
C	9.349493	9.279583	17.027982
C	9.712292	8.496541	12.246654
H	9.305918	8.303839	11.409946
C	11.556989	3.055196	20.574868
H	12.495553	2.897519	20.489043
C	2.941994	6.242880	18.504425

Table S30. XYZ coordinates of pbe0/SARC-ZORA-TZVP-calculated **dimer1**, with structure parameters taken from the SXRD-measurement.

Atom	X	Y	Z
Bi	4.790733	6.261425	18.358981
S	4.823664	3.937144	19.847891
S	4.782717	8.602082	16.901849
N	3.998862	5.114782	16.216428
N	3.991550	7.442141	20.511312
C	8.716015	9.007568	15.788650
C	9.396711	3.180662	19.555535
C	8.791587	3.523295	20.836423
C	6.717067	8.542918	14.446652
H	5.777134	8.429315	14.367574
C	8.906408	8.620511	13.388208
C	1.912330	6.491193	19.553091
C	10.754827	2.955924	19.474869
H	11.140064	2.726051	18.637012
C	7.177981	9.124904	18.118204
H	6.678558	9.135381	18.925994
C	8.530944	9.362134	18.169537
H	8.922248	9.591196	19.003458
C	7.380707	3.718789	20.921978
C	6.539617	8.875665	16.947316
C	7.518614	8.468426	13.351542
H	7.109289	8.303132	12.508918
C	7.187182	3.364223	18.541092
H	6.644166	3.334618	17.760753
C	11.021124	3.406167	21.858200
C	11.516932	9.411200	15.969539
H	12.453657	9.565552	16.037055
C	10.910168	9.080703	14.683763
C	7.315109	8.794376	15.725095
C	0.529428	6.491352	19.535980
H	0.091082	6.684545	20.355015
C	1.895582	5.984815	17.147760
C	8.518077	3.126175	18.453092
H	8.883610	2.911549	17.602035
C	9.501875	8.892722	14.620207
C	9.630033	3.601465	21.951089
C	-0.200699	6.254192	18.519092
H	-1.149301	6.243433	18.575388
C	6.869866	3.990247	22.249310
H	5.940582	4.114738	22.367915
C	11.257587	3.858015	24.248864
H	11.809999	3.962053	25.015856
C	3.690902	8.864245	20.318201
H	4.524772	9.356914	20.170801
H	3.240569	9.213796	21.114943
H	3.106016	8.971144	19.538840
C	7.692862	4.074605	23.334643
H	7.320859	4.259036	24.188462
C	4.862620	7.278027	21.606422
H	5.034655	6.320507	21.746953
H	4.456011	7.656475	22.411353
H	5.711293	7.733822	21.421745
C	3.687470	3.664804	16.419316
H	4.499469	3.192102	16.696418
H	3.356421	3.281748	15.579968
H	3.001823	3.572073	17.113269
C	6.589151	3.662502	19.819535
C	2.726798	5.813251	15.923095
H	2.917950	6.702088	15.534135
H	2.210747	5.292920	15.254589
C	10.723534	9.480055	17.062205
H	11.126559	9.687380	17.897984
C	2.708582	6.696814	20.794868
H	2.927466	5.817566	21.194803

H	2.174242	7.207708	21.449489
C	11.703789	8.950316	13.512875
H	12.645429	9.073073	13.548393
C	0.526778	5.991580	17.208871
H	0.027759	5.828087	16.418412
C	4.918632	5.254471	15.091984
H	5.203106	6.188923	15.017307
H	4.466403	4.983548	14.265372
H	5.701626	4.684294	15.237869
C	9.911106	4.014437	24.358864
H	9.531147	4.207275	25.206474
C	11.066381	8.644669	12.307765
H	11.586044	8.537530	11.519530
C	11.840433	3.541449	23.014421
H	12.779764	3.422661	22.947370
C	9.064329	3.891995	23.205087
C	9.349493	9.279583	17.027982
C	9.712292	8.496541	12.246654
H	9.305918	8.303839	11.409946
C	11.556989	3.055196	20.574868
H	12.495553	2.897519	20.489043
C	2.941994	6.242880	18.504425
Bi	15.371902	14.092655	6.085438
S	15.338971	16.416936	4.596529
S	15.379919	11.751998	7.542570
N	16.163774	15.239298	8.227992
N	16.171086	12.911939	3.933107
C	11.446620	11.346513	8.655769
C	10.765925	17.173418	4.888884
C	11.371048	16.830785	3.607996
C	13.445569	11.811163	9.997767
H	14.385502	11.924765	10.076845
C	11.256227	11.733570	11.056211
C	18.250305	13.862887	4.891328
C	9.407809	17.398157	4.969550
H	9.022571	17.628029	5.807407
C	12.984654	11.229176	6.326216
H	13.484077	11.218699	5.518425
C	11.631692	10.991946	6.274882
H	11.240387	10.762884	5.440961
C	12.781929	16.635291	3.522441
C	13.623018	11.478415	7.497103
C	12.644021	11.885655	11.092877
H	13.053347	12.050949	11.935501
C	12.975454	16.989857	5.903327
H	13.518469	17.019462	6.683666
C	9.141512	16.947913	2.586220
C	8.645703	10.942881	8.474880
H	7.708979	10.788528	8.407365
C	9.252467	11.273377	9.760657
C	12.847527	11.559704	8.719324
C	19.633207	13.862728	4.908439
H	20.071554	13.669535	4.089405
C	18.267053	14.369266	7.296659
C	11.644559	17.227905	5.991327
H	11.279025	17.442531	6.842384
C	10.660761	11.461358	9.824212
C	10.532602	16.752616	2.493331
C	20.363335	14.099888	5.925327
H	21.311937	14.110647	5.869032
C	13.292770	16.363834	2.195109
H	14.222054	16.239342	2.076505
C	8.905048	16.496066	0.195555
H	8.352637	16.392028	-0.571437
C	16.471734	11.489835	4.126218
H	15.637864	10.997166	4.273618
H	16.922066	11.140285	3.329477
H	17.056619	11.382936	4.905579

C	12.469773	16.279475	1.109777
H	12.841777	16.095045	0.255958
C	15.300015	13.076053	2.837997
H	15.127981	14.033573	2.697466
H	15.706625	12.697606	2.033067
H	14.451342	12.620258	3.022675
C	16.475165	16.689276	8.025103
H	15.663167	17.161979	7.748001
H	16.806214	17.072332	8.864451
H	17.160812	16.782007	7.331150
C	13.573484	16.691578	4.624884
C	17.435837	14.540830	8.521325
H	17.244685	13.651993	8.910284
H	17.951889	15.061160	9.189831
C	9.439101	10.874026	7.382215
H	9.036076	10.666701	6.546435
C	17.454053	13.657267	3.649552
H	17.235169	14.536514	3.249617
H	17.988393	13.146372	2.994930
C	8.458846	11.403764	10.931544
H	7.517206	11.281007	10.896027
C	19.635857	14.362500	7.235548
H	20.134877	14.525993	8.026007
C	15.244003	15.099609	9.352435
H	14.959529	14.165158	9.427113
H	15.696232	15.370532	10.179047
H	14.461010	15.669786	9.206551
C	10.251529	16.339644	0.085555
H	10.631488	16.146805	-0.762055
C	9.096255	11.709411	12.136654
H	8.576591	11.816551	12.924889
C	8.322203	16.812632	1.429999
H	7.382871	16.931419	1.497050
C	11.098307	16.462085	1.239332
C	10.813142	11.074498	7.416437
C	10.450344	11.857539	12.197765
H	10.856718	12.050241	13.034473
C	8.605646	17.298885	3.869552
H	7.667082	17.456561	3.955376
C	17.220641	14.111200	5.939994

Table S31. XYZ coordinates of pbe0/SARC-ZORA-TZVP-calculated **dimer2**, with structure parameters taken from the SXRD-measurement.

Atom	X	Y	Z
Bi	4.79000	6.26100	18.35900
S	4.82400	3.93800	19.84800
S	4.78300	8.60200	16.90200
N	3.99700	5.11400	16.21600
N	3.99000	7.44000	20.50900
C	8.71500	9.00700	15.78600
C	9.39600	3.17900	19.55300
C	8.79100	3.52200	20.83400
C	6.71700	8.54100	14.44700
H	5.77800	8.42800	14.36500
C	8.90800	8.62100	13.38800
C	1.90500	6.49300	19.56300
C	10.75500	2.95600	19.47200
H	11.14400	2.72600	18.63600
C	7.17800	9.12600	18.12100
H	6.67800	9.13600	18.92800
C	8.53200	9.36400	18.16700
H	8.92300	9.59300	19.00100
C	7.38100	3.71900	20.92400
C	6.53300	8.87600	16.94500
C	7.51800	8.46800	13.35200
H	7.11100	8.30200	12.51000
C	7.18700	3.36400	18.54100
H	6.64300	3.33300	17.76000
C	11.02100	3.40600	21.85800
C	11.52700	9.41100	15.97000
H	12.45600	9.56500	16.03700
C	10.91000	9.08100	14.68600
C	7.31500	8.79400	15.72500
C	0.52900	6.49100	19.53800
H	0.08500	6.68300	20.35500
C	1.89400	5.98800	17.14800
C	8.51900	3.12700	18.45300
H	8.88400	2.91400	17.60200
C	9.50000	8.89300	14.62300
C	9.62900	3.60100	21.94900
C	-0.19100	6.25300	18.51700
H	-1.14300	6.24100	18.57200
C	6.87000	3.99100	22.24900
H	5.94000	4.11600	22.36900
C	11.25700	3.85700	24.24900
H	11.81000	3.96000	25.01500
C	3.69000	8.86400	20.32100
H	4.52500	9.35400	20.16000
H	3.25600	9.21500	21.12500
H	3.09500	8.97400	19.55100
C	7.69300	4.07300	23.33500
H	7.32400	4.25500	24.19000
C	4.87300	7.27800	21.60400
H	5.05300	6.32100	21.73400
H	4.45400	7.64100	22.41300
H	5.70800	7.74800	21.42400
C	3.68700	3.66400	16.41900
H	4.50100	3.19100	16.69200
H	3.35100	3.28100	15.58300
H	3.00600	3.57100	17.11900
C	6.59000	3.66500	19.82200
C	2.71700	5.81400	15.92300
H	2.20600	5.29300	15.25600
H	2.91400	6.70300	15.53300
C	10.72400	9.48000	17.06000
H	11.12800	9.68700	17.89600
C	2.70900	6.69800	20.79500
H	2.17600	7.20900	21.45300

H	2.92600	5.81800	21.19400
C	11.70500	8.95300	13.51000
H	12.64300	9.07600	13.54400
C	0.52700	5.99200	17.21600
H	0.02600	5.83000	16.42800
C	4.91900	5.25400	15.09200
H	5.20800	6.18800	15.02000
H	4.46700	4.98900	14.26400
H	5.70100	4.68100	15.23600
C	9.91100	4.01400	24.35900
H	9.53000	4.20600	25.20600
C	11.06600	8.64400	12.31000
H	11.58500	8.53600	11.52000
C	11.84100	3.54300	23.01200
H	12.78000	3.42200	22.94500
C	9.06900	3.89300	23.20000
C	9.34900	9.28000	17.02800
C	9.71100	8.49500	12.24900
H	9.30500	8.30000	11.41400
C	11.56900	3.05900	20.57200
H	12.49800	2.89800	20.48700
C	2.69400	6.23300	18.48700
Bi	14.91300	6.26100	18.35900
S	14.94700	3.93800	19.84800
S	14.90600	8.60200	16.90200
N	14.12000	5.11400	16.21600
N	14.11200	7.44000	20.50900
C	18.83800	9.00700	15.78600
C	19.51900	3.17900	19.55300
C	18.91400	3.52200	20.83400
C	16.84000	8.54100	14.44700
H	15.90100	8.42800	14.36500
C	19.03100	8.62100	13.38800
C	12.02800	6.49300	19.56300
C	20.87800	2.95600	19.47200
H	21.26700	2.72600	18.63600
C	17.30100	9.12600	18.12100
H	16.80000	9.13600	18.92800
C	18.65500	9.36400	18.16700
H	19.04600	9.59300	19.00100
C	17.50400	3.71900	20.92400
C	16.65600	8.87600	16.94500
C	17.64100	8.46800	13.35200
H	17.23400	8.30200	12.51000
C	17.31000	3.36400	18.54100
H	16.76500	3.33300	17.76000
C	21.14400	3.40600	21.85800
C	21.65000	9.41100	15.97000
H	22.57900	9.56500	16.03700
C	21.03300	9.08100	14.68600
C	17.43800	8.79400	15.72500
C	10.65200	6.49100	19.53800
H	10.20800	6.68300	20.35500
C	12.01600	5.98800	17.14800
C	18.64100	3.12700	18.45300
H	19.00700	2.91400	17.60200
C	19.62300	8.89300	14.62300
C	19.75200	3.60100	21.94900
C	9.93200	6.25300	18.51700
H	8.98000	6.24100	18.57200
C	16.99300	3.99100	22.24900
H	16.06300	4.11600	22.36900
C	21.38000	3.85700	24.24900
H	21.93300	3.96000	25.01500
C	13.81300	8.86400	20.32100
H	14.64800	9.35400	20.16000
H	13.37900	9.21500	21.12500
H	13.21800	8.97400	19.55100

C	17.81600	4.07300	23.33500
H	17.44700	4.25500	24.19000
C	14.99600	7.27800	21.60400
H	15.17600	6.32100	21.73400
H	14.57700	7.64100	22.41300
H	15.83100	7.74800	21.42400
C	13.81000	3.66400	16.41900
H	14.62400	3.19100	16.69200
H	13.47400	3.28100	15.58300
H	13.12900	3.57100	17.11900
C	16.71300	3.66500	19.82200
C	12.84000	5.81400	15.92300
H	12.32900	5.29300	15.25600
H	13.03700	6.70300	15.53300
C	20.84600	9.48000	17.06000
H	21.25100	9.68700	17.89600
C	12.83200	6.69800	20.79500
H	12.29900	7.20900	21.45300
H	13.04900	5.81800	21.19400
C	21.82800	8.95300	13.51000
H	22.76600	9.07600	13.54400
C	10.65000	5.99200	17.21600
H	10.14900	5.83000	16.42800
C	15.04200	5.25400	15.09200
H	15.33100	6.18800	15.02000
H	14.59000	4.98900	14.26400
H	15.82400	4.68100	15.23600
C	20.03400	4.01400	24.35900
H	19.65300	4.20600	25.20600
C	21.18900	8.64400	12.31000
H	21.70800	8.53600	11.52000
C	21.96400	3.54300	23.01200
H	22.90300	3.42200	22.94500
C	19.19200	3.89300	23.20000
C	19.47200	9.28000	17.02800
C	19.83400	8.49500	12.24900
H	19.42800	8.30000	11.41400
C	21.69200	3.05900	20.57200
H	22.62100	2.89800	20.48700
C	12.81700	6.23300	18.48700

Table S32. XYZ coordinates of pbe0/SARC-ZORA-TZVP-calculated **tetramer**, with structure parameters taken from the SXRD-measurement.

Atom	X	Y	z
Bi	4.79000	6.26100	18.35900
S	4.82400	3.93800	19.84800
S	4.78300	8.60200	16.90200
N	3.99700	5.11400	16.21600
N	3.99000	7.44000	20.50900
C	8.71500	9.00700	15.78600
C	9.39600	3.17900	19.55300
C	8.79100	3.52200	20.83400
C	6.71700	8.54100	14.44700
H	5.77800	8.42800	14.36500
C	8.90800	8.62100	13.38800
C	1.90500	6.49300	19.56300
C	10.75500	2.95600	19.47200
H	11.14400	2.72600	18.63600
C	7.17800	9.12600	18.12100
H	6.67800	9.13600	18.92800
C	8.53200	9.36400	18.16700
H	8.92300	9.59300	19.00100
C	7.38100	3.71900	20.92400
C	6.53300	8.87600	16.94500
C	7.51800	8.46800	13.35200
H	7.11100	8.30200	12.51000
C	7.18700	3.36400	18.54100
H	6.64300	3.33300	17.76000
C	11.02100	3.40600	21.85800
C	11.52700	9.41100	15.97000
H	12.45600	9.56500	16.03700
C	10.91000	9.08100	14.68600
C	7.31500	8.79400	15.72500
C	0.52900	6.49100	19.53800
H	0.08500	6.68300	20.35500
C	1.89400	5.98800	17.14800
C	8.51900	3.12700	18.45300
H	8.88400	2.91400	17.60200
C	9.50000	8.89300	14.62300
C	9.62900	3.60100	21.94900
C	-0.19100	6.25300	18.51700
H	-1.14300	6.24100	18.57200
C	6.87000	3.99100	22.24900
H	5.94000	4.11600	22.36900
C	11.25700	3.85700	24.24900
H	11.81000	3.96000	25.01500
C	3.69000	8.86400	20.32100
H	4.52500	9.35400	20.16000
H	3.25600	9.21500	21.12500
H	3.09500	8.97400	19.55100
C	7.69300	4.07300	23.33500
H	7.32400	4.25500	24.19000
C	4.87300	7.27800	21.60400
H	5.05300	6.32100	21.73400
H	4.45400	7.64100	22.41300
H	5.70800	7.74800	21.42400
C	3.68700	3.66400	16.41900
H	4.50100	3.19100	16.69200
H	3.35100	3.28100	15.58300
H	3.00600	3.57100	17.11900
C	6.59000	3.66500	19.82200
C	2.71700	5.81400	15.92300
H	2.20600	5.29300	15.25600
H	2.91400	6.70300	15.53300
C	10.72400	9.48000	17.06000
H	11.12800	9.68700	17.89600
C	2.70900	6.69800	20.79500
H	2.17600	7.20900	21.45300

H	2.92600	5.81800	21.19400
C	11.70500	8.95300	13.51000
H	12.64300	9.07600	13.54400
C	0.52700	5.99200	17.21600
H	0.02600	5.83000	16.42800
C	4.91900	5.25400	15.09200
H	5.20800	6.18800	15.02000
H	4.46700	4.98900	14.26400
H	5.70100	4.68100	15.23600
C	9.91100	4.01400	24.35900
H	9.53000	4.20600	25.20600
C	11.06600	8.64400	12.31000
H	11.58500	8.53600	11.52000
C	11.84100	3.54300	23.01200
H	12.78000	3.42200	22.94500
C	9.06900	3.89300	23.20000
C	9.34900	9.28000	17.02800
C	9.71100	8.49500	12.24900
H	9.30500	8.30000	11.41400
C	11.56900	3.05900	20.57200
H	12.49800	2.89800	20.48700
C	2.69400	6.23300	18.48700
Bi	14.91300	6.26100	18.35900
S	14.94700	3.93800	19.84800
S	14.90600	8.60200	16.90200
N	14.12000	5.11400	16.21600
N	14.11200	7.44000	20.50900
C	18.83800	9.00700	15.78600
C	19.51900	3.17900	19.55300
C	18.91400	3.52200	20.83400
C	16.84000	8.54100	14.44700
H	15.90100	8.42800	14.36500
C	19.03100	8.62100	13.38800
C	12.02800	6.49300	19.56300
C	20.87800	2.95600	19.47200
H	21.26700	2.72600	18.63600
C	17.30100	9.12600	18.12100
H	16.80000	9.13600	18.92800
C	18.65500	9.36400	18.16700
H	19.04600	9.59300	19.00100
C	17.50400	3.71900	20.92400
C	16.65600	8.87600	16.94500
C	17.64100	8.46800	13.35200
H	17.23400	8.30200	12.51000
C	17.31000	3.36400	18.54100
H	16.76500	3.33300	17.76000
C	21.14400	3.40600	21.85800
C	21.65000	9.41100	15.97000
H	22.57900	9.56500	16.03700
C	21.03300	9.08100	14.68600
C	17.43800	8.79400	15.72500
C	10.65200	6.49100	19.53800
H	10.20800	6.68300	20.35500
C	12.01600	5.98800	17.14800
C	18.64100	3.12700	18.45300
H	19.00700	2.91400	17.60200
C	19.62300	8.89300	14.62300
C	19.75200	3.60100	21.94900
C	9.93200	6.25300	18.51700
H	8.98000	6.24100	18.57200
C	16.99300	3.99100	22.24900
H	16.06300	4.11600	22.36900
C	21.38000	3.85700	24.24900
H	21.93300	3.96000	25.01500
C	13.81300	8.86400	20.32100
H	14.64800	9.35400	20.16000
H	13.37900	9.21500	21.12500
H	13.21800	8.97400	19.55100

C	17.81600	4.07300	23.33500
H	17.44700	4.25500	24.19000
C	14.99600	7.27800	21.60400
H	15.17600	6.32100	21.73400
H	14.57700	7.64100	22.41300
H	15.83100	7.74800	21.42400
C	13.81000	3.66400	16.41900
H	14.62400	3.19100	16.69200
H	13.47400	3.28100	15.58300
H	13.12900	3.57100	17.11900
C	16.71300	3.66500	19.82200
C	12.84000	5.81400	15.92300
H	12.32900	5.29300	15.25600
H	13.03700	6.70300	15.53300
C	20.84600	9.48000	17.06000
H	21.25100	9.68700	17.89600
C	12.83200	6.69800	20.79500
H	12.29900	7.20900	21.45300
H	13.04900	5.81800	21.19400
C	21.82800	8.95300	13.51000
H	22.76600	9.07600	13.54400
C	10.65000	5.99200	17.21600
H	10.14900	5.83000	16.42800
C	15.04200	5.25400	15.09200
H	15.33100	6.18800	15.02000
H	14.59000	4.98900	14.26400
H	15.82400	4.68100	15.23600
C	20.03400	4.01400	24.35900
H	19.65300	4.20600	25.20600
C	21.18900	8.64400	12.31000
H	21.70800	8.53600	11.52000
C	21.96400	3.54300	23.01200
H	22.90300	3.42200	22.94500
C	19.19200	3.89300	23.20000
C	19.47200	9.28000	17.02800
C	19.83400	8.49500	12.24900
H	19.42800	8.30000	11.41400
C	21.69200	3.05900	20.57200
H	22.62100	2.89800	20.48700
C	12.81700	6.23300	18.48700
Bi	15.71900	-1.56900	30.52900
S	15.68500	0.75400	29.04100
S	15.72600	-3.91000	31.98700
N	16.51200	-0.42200	32.67200
N	16.51900	-2.74800	28.38000
C	11.79400	-4.31500	33.10300
C	11.11300	1.51400	29.33600
C	11.71800	1.17000	28.05500
C	13.79200	-3.84900	34.44200
H	14.73100	-3.73500	34.52300
C	11.60100	-3.92800	35.50100
C	18.60400	-1.80100	29.32600
C	9.75400	1.73700	29.41600
H	9.36500	1.96600	30.25300
C	13.33000	-4.43400	30.76800
H	13.83100	-4.44300	29.96100
C	11.97700	-4.67100	30.72200
H	11.58600	-4.90100	29.88800
C	13.12800	0.97300	27.96400
C	13.97600	-4.18400	31.94400
C	12.99100	-3.77500	35.53700
H	13.39800	-3.60900	36.37900
C	13.32200	1.32800	30.34800
H	13.86600	1.35900	31.12900
C	9.48800	1.28600	27.03100
C	8.98200	-4.71900	32.91900
H	8.05300	-4.87300	32.85200
C	9.59900	-4.38900	34.20300

C	13.19400	-4.10200	33.16400
C	19.98000	-1.79800	29.35000
H	20.42400	-1.99100	28.53400
C	18.61500	-1.29600	31.74100
C	11.99000	1.56500	30.43600
H	11.62500	1.77900	31.28700
C	11.00900	-4.20100	34.26600
C	10.88000	1.09100	26.94000
C	20.70000	-1.56100	30.37200
H	21.65100	-1.54800	30.31700
C	13.63900	0.70100	26.64000
H	14.56900	0.57600	26.52000
C	9.25200	0.83500	24.64000
H	8.69900	0.73200	23.87400
C	16.81900	-4.17100	28.56800
H	15.98300	-4.66100	28.72900
H	17.25300	-4.52300	27.76400
H	17.41400	-4.28200	29.33800
C	12.81600	0.61900	25.55400
H	13.18500	0.43700	24.69900
C	15.63600	-2.58600	27.28500
H	15.45600	-1.62900	27.15500
H	16.05400	-2.94900	26.47600
H	14.80100	-3.05600	27.46500
C	16.82200	1.02800	32.47000
H	16.00800	1.50100	32.19700
H	17.15800	1.41100	33.30600
H	17.50200	1.12100	31.77000
C	13.91900	1.02800	29.06700
C	17.79200	-1.12200	32.96600
H	18.30300	-0.60100	33.63300
H	17.59500	-2.01100	33.35600
C	9.78500	-4.78800	31.82900
H	9.38100	-4.99500	30.99300
C	17.80000	-2.00600	28.09400
H	18.33300	-2.51600	27.43600
H	17.58300	-1.12600	27.69500
C	8.80400	-4.26100	35.37800
H	7.86600	-4.38300	35.34500
C	19.98200	-1.30000	31.67300
H	20.48300	-1.13800	32.46100
C	15.59000	-0.56200	33.79700
H	15.30100	-1.49600	33.86800
H	16.04200	-0.29600	34.62500
H	14.80800	0.01100	33.65300
C	10.59800	0.67800	24.53000
H	10.97900	0.48600	23.68300
C	9.44300	-3.95200	36.57900
H	8.92300	-3.84400	37.36800
C	8.66800	1.14900	25.87700
H	7.72900	1.27000	25.94300
C	11.44000	0.79900	25.68900
C	11.15900	-4.58700	31.86100
C	10.79800	-3.80300	36.64000
H	11.20400	-3.60800	37.47500
C	8.94000	1.63400	28.31600
H	8.01100	1.79400	28.40200
C	17.81400	-1.54100	30.40200
Bi	5.59600	-1.56900	30.52900
S	5.56200	0.75400	29.04100
S	5.60300	-3.91000	31.98700
N	6.38900	-0.42200	32.67200
N	6.39700	-2.74800	28.38000
C	1.67100	-4.31500	33.10300
C	0.99000	1.51400	29.33600
C	1.59500	1.17000	28.05500
C	3.66900	-3.84900	34.44200
H	4.60800	-3.73500	34.52300

C	1.47800	-3.92800	35.50100
C	8.48100	-1.80100	29.32600
C	-0.36900	1.73700	29.41600
H	-0.75800	1.96600	30.25300
C	3.20800	-4.43400	30.76800
H	3.70900	-4.44300	29.96100
C	1.85400	-4.67100	30.72200
H	1.46300	-4.90100	29.88800
C	3.00500	0.97300	27.96400
C	3.85300	-4.18400	31.94400
C	2.86800	-3.77500	35.53700
H	3.27500	-3.60900	36.37900
C	3.19900	1.32800	30.34800
H	3.74400	1.35900	31.12900
C	-0.63500	1.28600	27.03100
C	-1.14100	-4.71900	32.91900
H	-2.07000	-4.87300	32.85200
C	-0.52400	-4.38900	34.20300
C	3.07100	-4.10200	33.16400
C	9.85700	-1.79800	29.35000
H	10.30100	-1.99100	28.53400
C	8.49300	-1.29600	31.74100
C	1.86700	1.56500	30.43600
H	1.50200	1.77900	31.28700
C	0.88600	-4.20100	34.26600
C	0.75700	1.09100	26.94000
C	10.57700	-1.56100	30.37200
H	11.52900	-1.54800	30.31700
C	3.51600	0.70100	26.64000
H	4.44600	0.57600	26.52000
C	-0.87100	0.83500	24.64000
H	-1.42400	0.73200	23.87400
C	6.69600	-4.17100	28.56800
H	5.86100	-4.66100	28.72900
H	7.13000	-4.52300	27.76400
H	7.29100	-4.28200	29.33800
C	2.69300	0.61900	25.55400
H	3.06200	0.43700	24.69900
C	5.51300	-2.58600	27.28500
H	5.33300	-1.62900	27.15500
H	5.93200	-2.94900	26.47600
H	4.67800	-3.05600	27.46500
C	6.69900	1.02800	32.47000
H	5.88500	1.50100	32.19700
H	7.03500	1.41100	33.30600
H	7.38000	1.12100	31.77000
C	3.79600	1.02800	29.06700
C	7.66900	-1.12200	32.96600
H	8.18000	-0.60100	33.63300
H	7.47200	-2.01100	33.35600
C	-0.33700	-4.78800	31.82900
H	-0.74200	-4.99500	30.99300
C	7.67700	-2.00600	28.09400
H	8.21000	-2.51600	27.43600
H	7.46000	-1.12600	27.69500
C	-1.31900	-4.26100	35.37800
H	-2.25700	-4.38300	35.34500
C	9.85900	-1.30000	31.67300
H	10.36000	-1.13800	32.46100
C	5.46700	-0.56200	33.79700
H	5.17800	-1.49600	33.86800
H	5.91900	-0.29600	34.62500
H	4.68500	0.01100	33.65300
C	0.47500	0.67800	24.53000
H	0.85600	0.48600	23.68300
C	-0.68000	-3.95200	36.57900
H	-1.19900	-3.84400	37.36800
C	-1.45500	1.14900	25.87700

H	-2.39400	1.27000	25.94300
C	1.31700	0.79900	25.68900
C	1.03700	-4.58700	31.86100
C	0.67500	-3.80300	36.64000
H	1.08100	-3.60800	37.47500
C	-1.18300	1.63400	28.31600
H	-2.11200	1.79400	28.40200
C	7.69200	-1.54100	30.40200

Photoluminescence Data

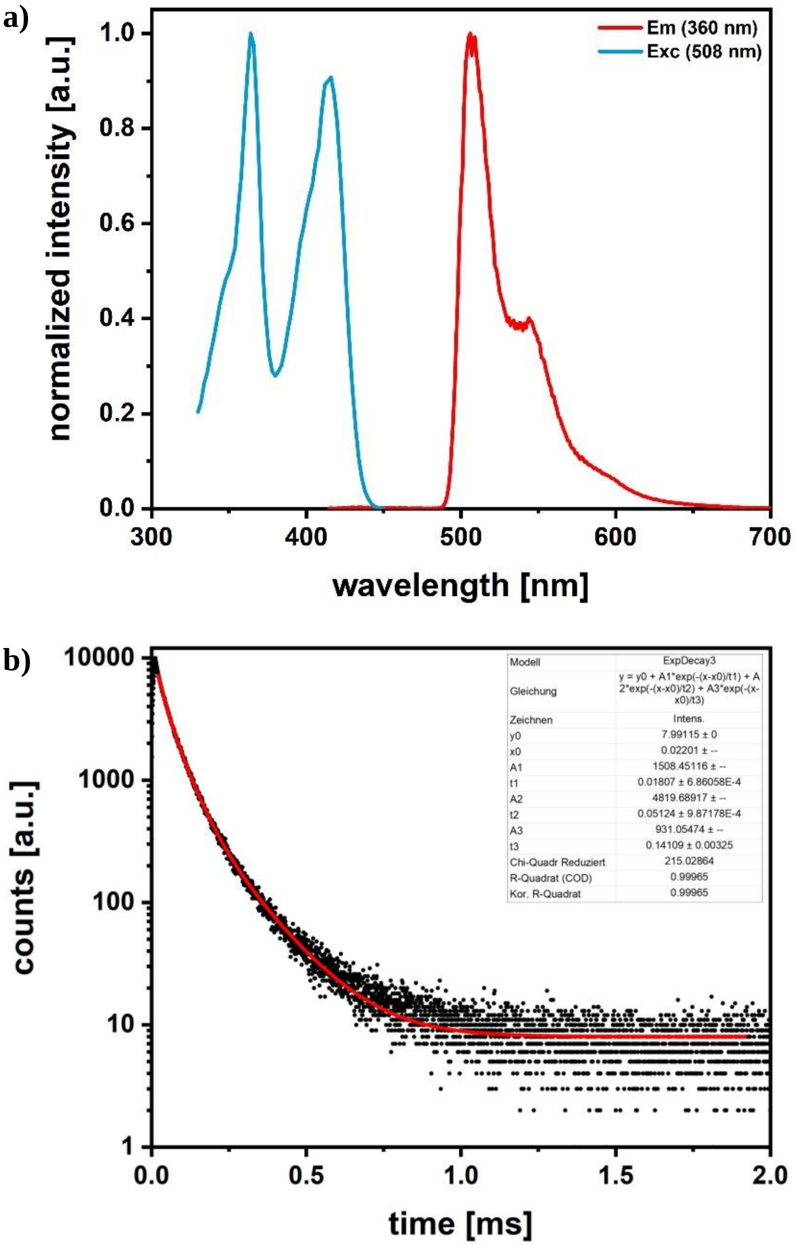

Figure S46. PL data of complex **2** at 77 K in MeTHF. a) Emission (red) and excitation spectra (blue) of **2**; b) Lifetime of the 509 nm emission of **2**.

a)

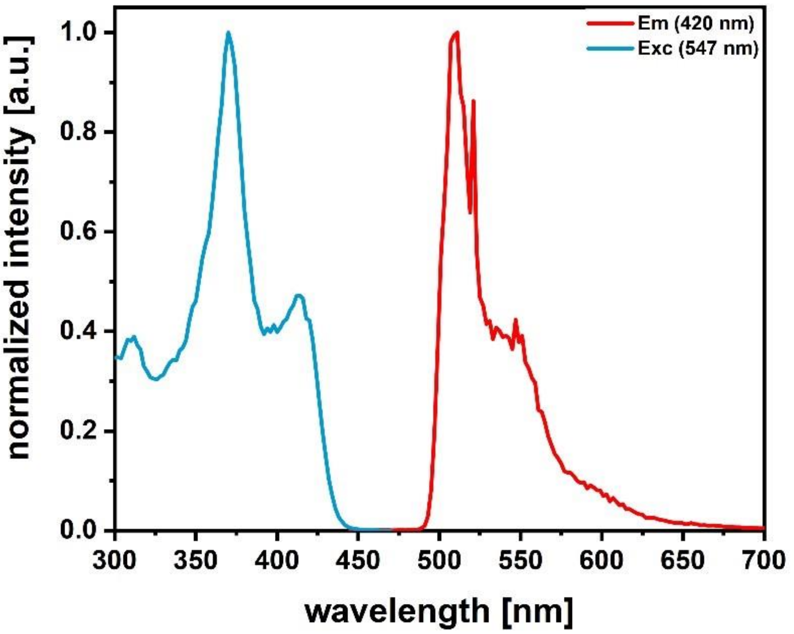

b)

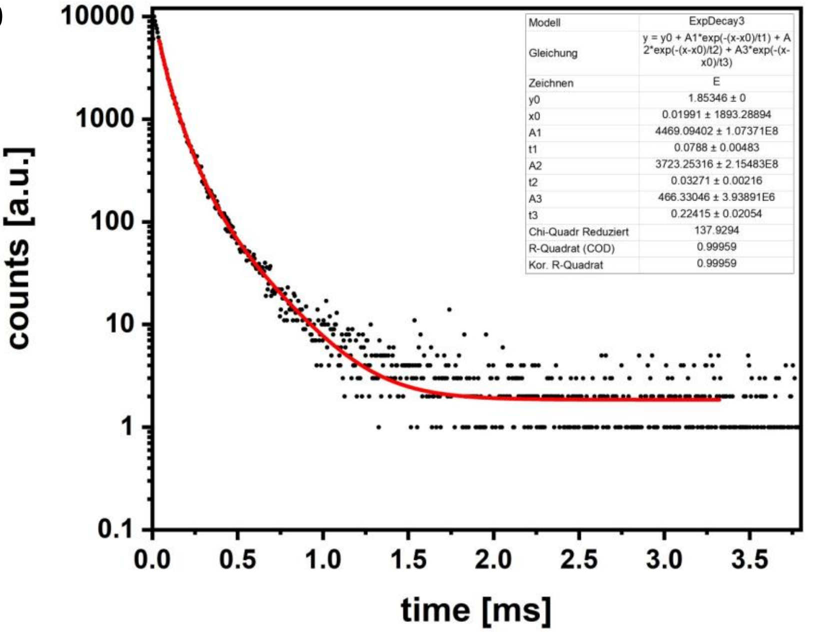

Figure S47. PL Data of complex **4** at 77 K in MeTHF. a) Emission (red) and excitation spectra (blue) of **4**; b) Lifetime of the 509 nm emission of **4**.

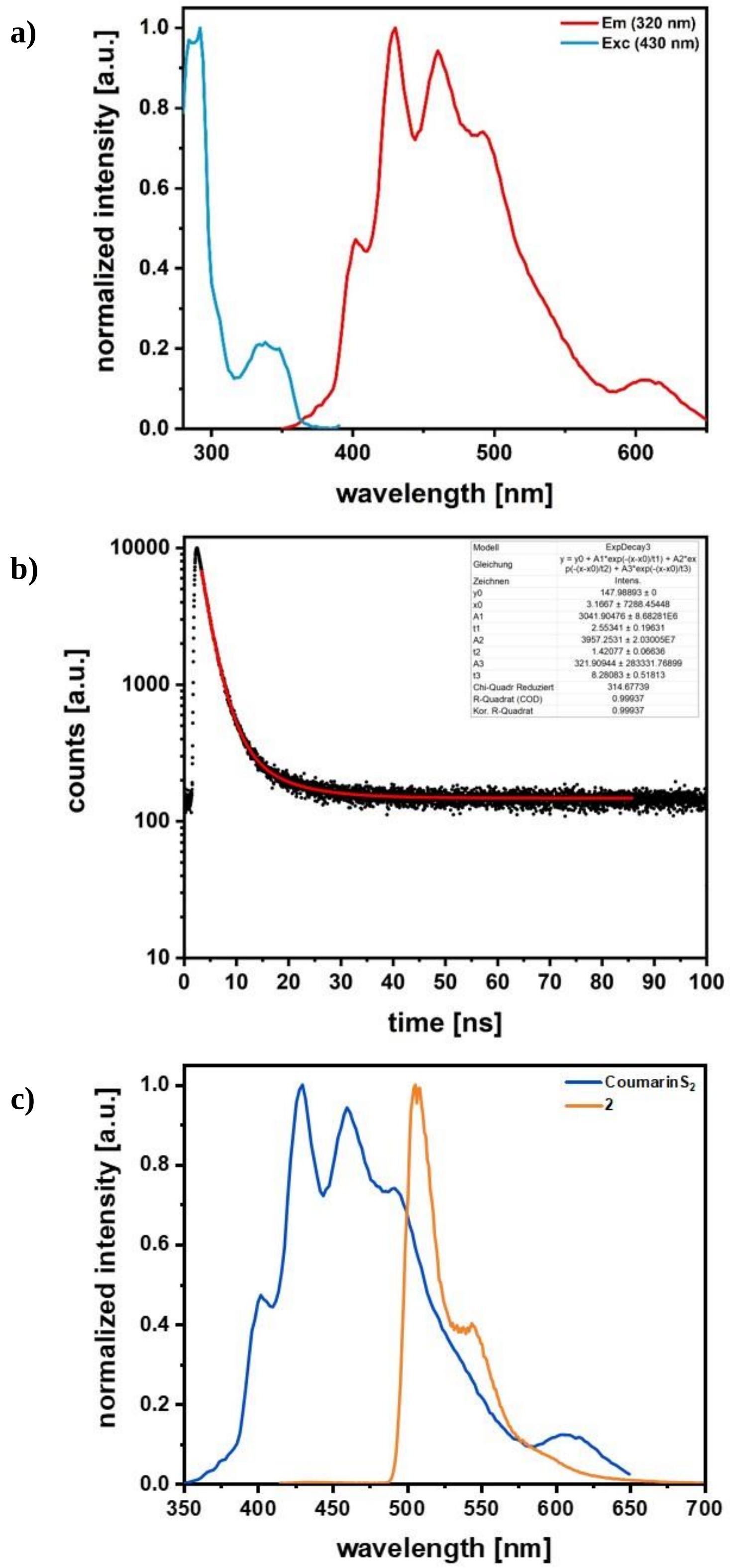

Figure S48. PL Data of **CoumarinS$_2$** at 77 K in MeTHF. a) Emission (red) and excitation spectra (blue) of **CoumarinS$_2$**; b) Lifetime of the 509 nm emission of **2**; c) Emission spectra of **CoumarinS$_2$** (blue) and **2** (orange) at room temperature

a)

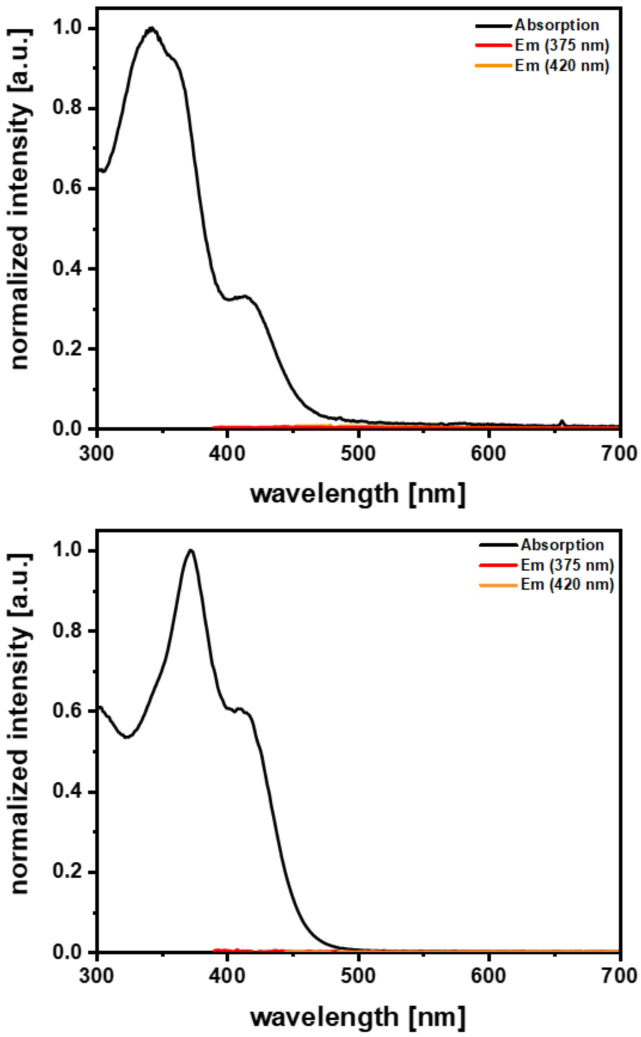

b)

Figure S49. PL Data of complexes **2** and **4** at room temperature in CH_2Cl_2. a) Absorption (black) and emission (red and orange) of **2**; b) Absorption (black) and emission spectra of **4** (red and orange).

a)

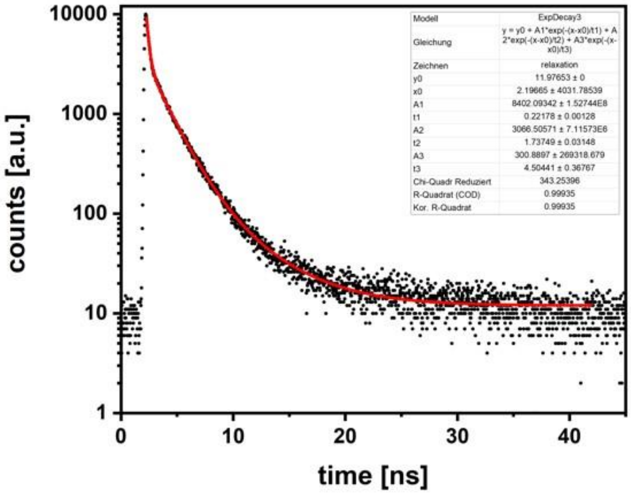

b)

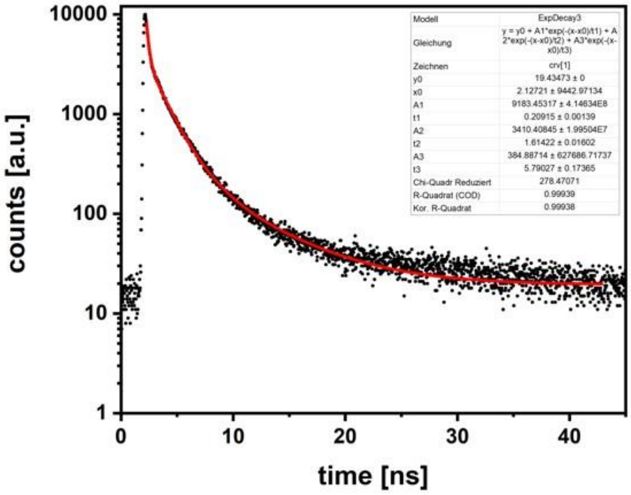

c)

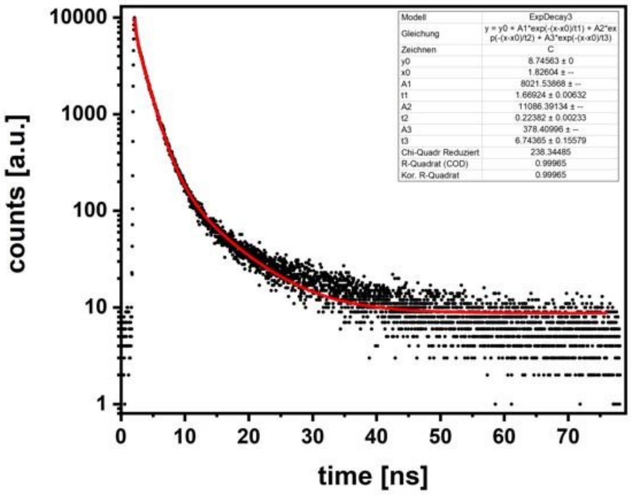

Figure S50. Lifetime measurements for the products formed after photodecomposition of complexes **2** and **4** upon irradiation in degassed CH_2Cl_2 (λ = 352 nm, output power 4 W, 5 min). a) Lifetime of the 393 nm emission of decomposed **2**; b) Lifetime of the 405 nm emission of decomposed **4**; c) Lifetime of the 392 nm emission of **CoumarinS$_2$**.

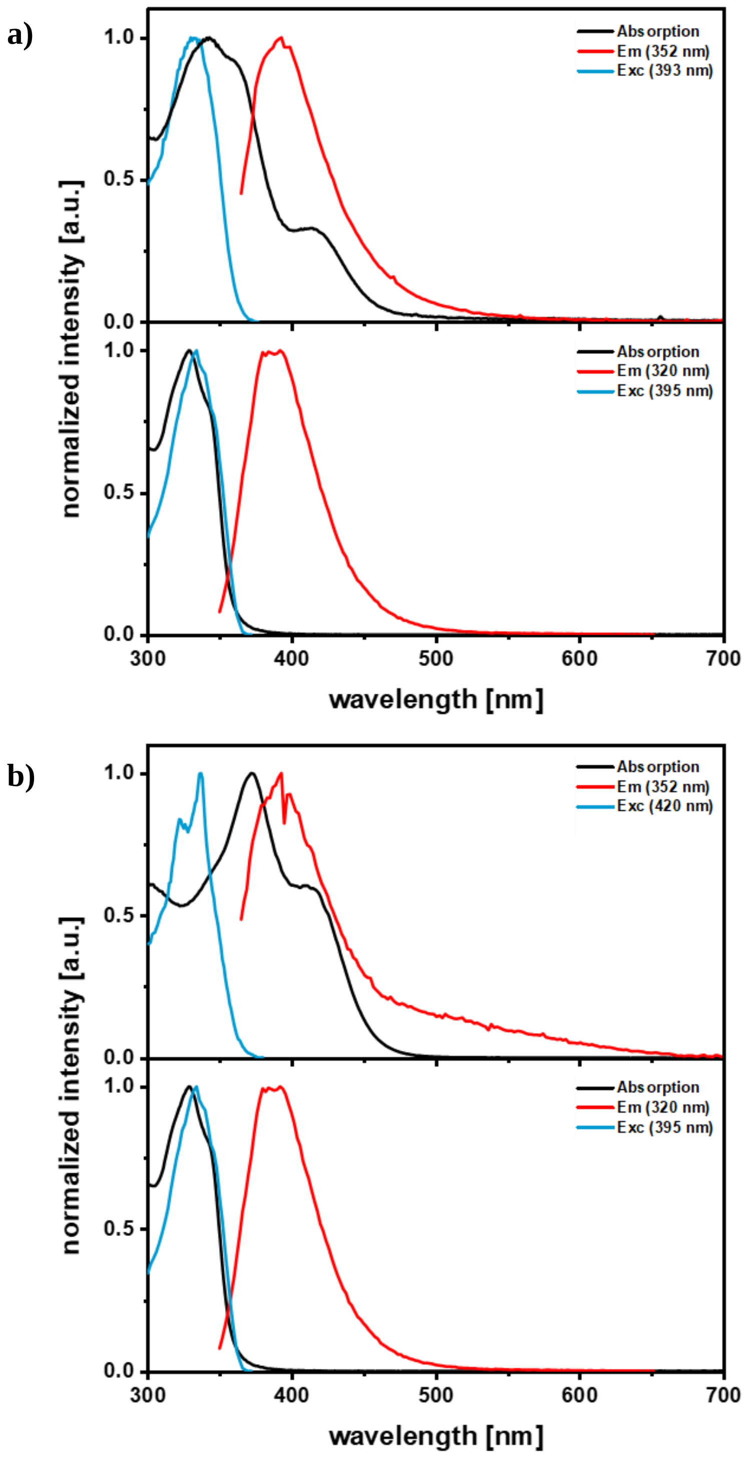

Figure S51. Partial decomposition of complexes **2** and **4** after photoirradiation in degassed CH_2Cl_2 (λ = 352 nm, output power 4 W, 3 min). Top: Absorption (black), emission (red) and excitation spectra (blue) of decomposed **2**; bottom: Absorption (black), emission (red) and excitation spectra (blue) of **CoumarinS**$_2$; b) Top: Absorption (black), emission (red) and excitation spectra (blue) of decomposed **4**; bottom: Absorption (black), emission (red) and excitation spectra (blue) of **CoumarinS**$_2$.

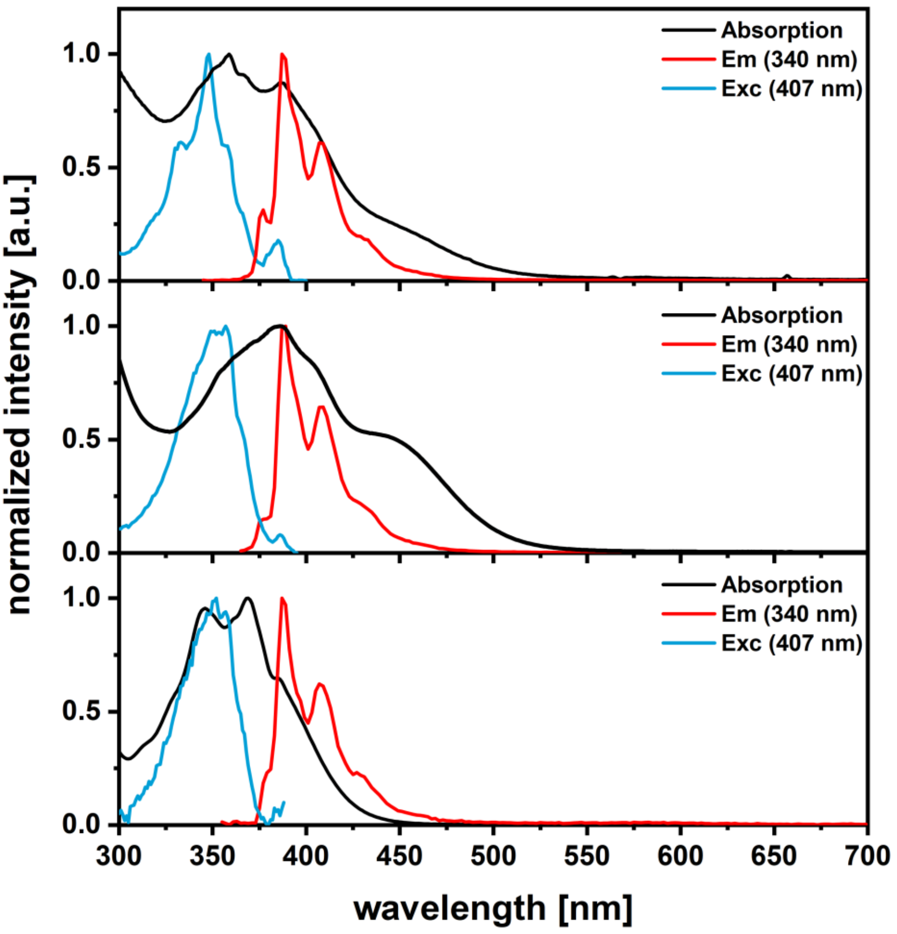

Figure S52. Decomposition of complexes **1** and **3** in degassed CH_2Cl_2 (λ = 340 nm, output power 300 W, 3 min). at room temperature (PL). Top: Absorption (black), emission (red) and excitation spectra (blue) of decomposed **3**; middle: Absorption (black), emission (red) and excitation spectra (blue) of decomposed **1**; bottom: Absorption (black), emission (red) and excitation spectra (blue) of **PyreneS$_2$**.

a)

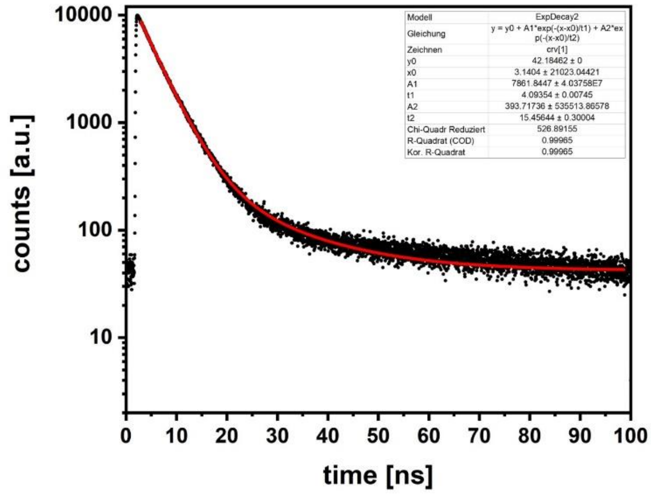

b)

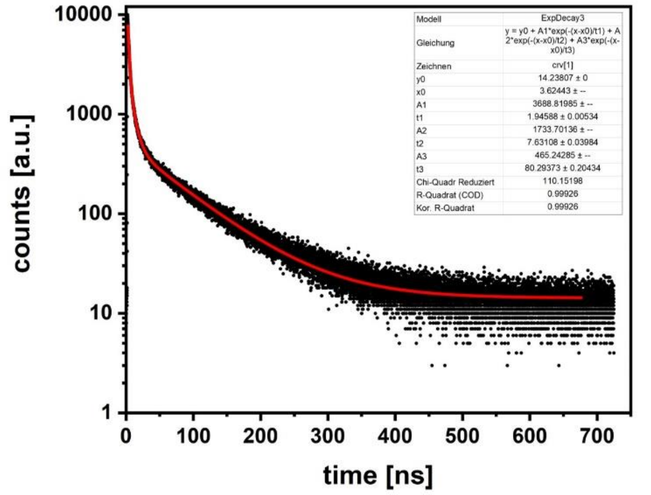

c)

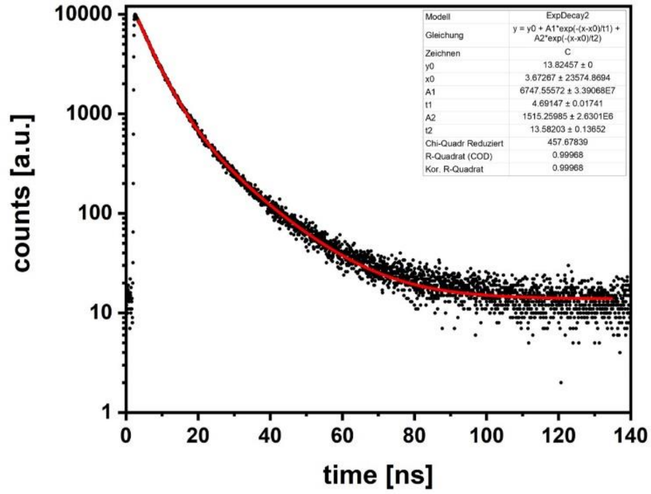

Figure S53. Lifetime measurements of the products formed after decomposition of complexes **1** and **3** after irradiation in degassed CH_2Cl_2 (λ = 352 nm, output power 4 W, 5 min). a) Lifetime of the 388 nm emission of decomposed **1**; b) Lifetime of the 387 nm emission of decomposed **3**; c) Lifetime of the 387 nm emission of **PyreneS₂**.

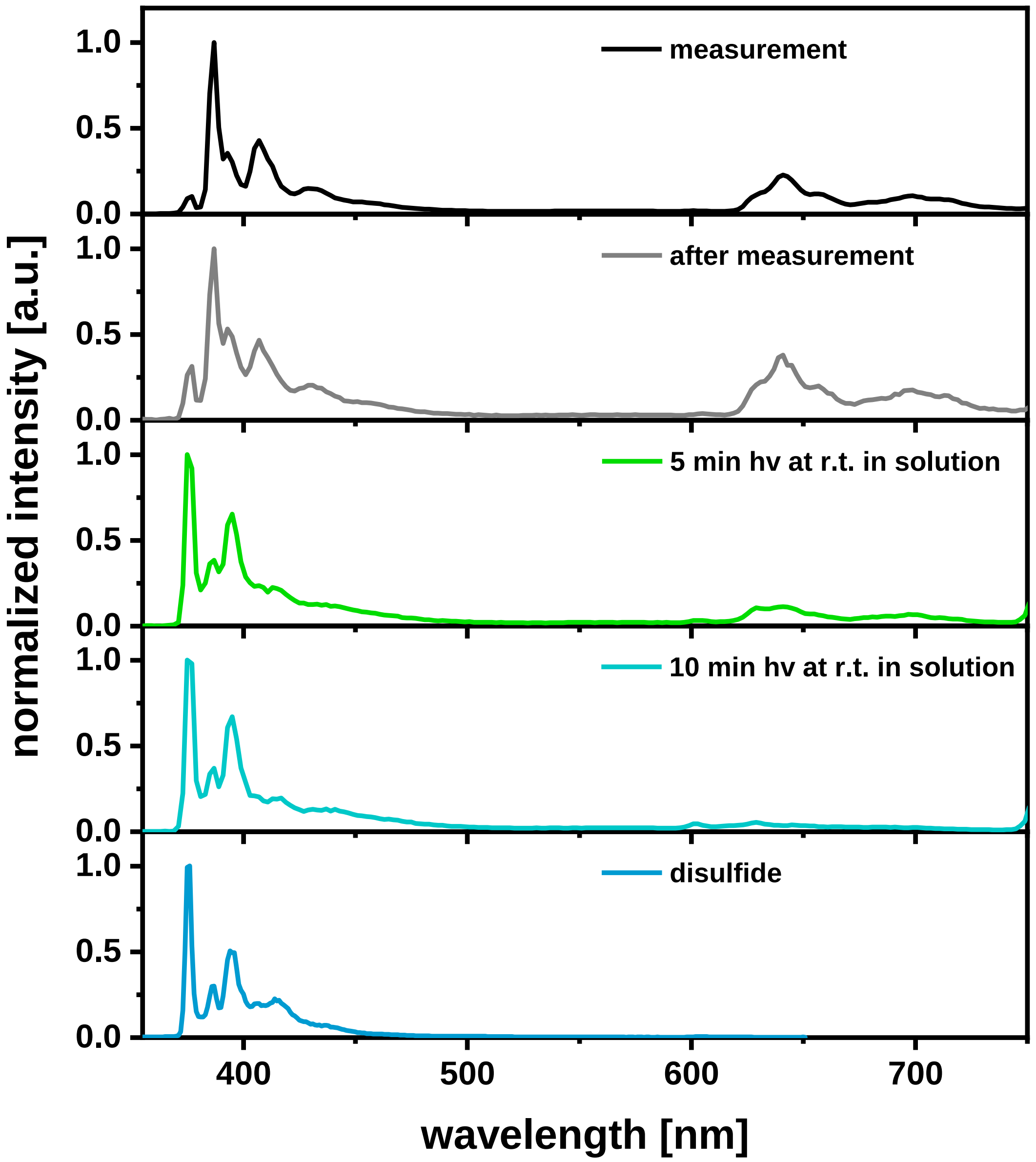

Figure S54. Photodecomposition experiments of complex **3**. Spectra recorded before and after irradiation in MeTHF (PL) (λ = 340 nm, output power 300 W, 10 min). Top panel (black): Emission spectrum ($\lambda_{exc.}$ = 340 nm) of **3** at 77 K in MeTHF before irradiation; second from top (gray): Emission spectrum ($\lambda_{exc.}$ = 340 nm) of **3** at 77 K in MeTHF after irradiation (λ = 340 nm, output power 300 W, 30 min) at 77 K for 10 min. The complex has remained intact. Panel 2 (green): Emission spectrum ($\lambda_{exc.}$ = 340 nm) of complex **3** at 77 K in MeTHF after irradiation at room temperature in solution for 5 minutes at 340 nm, output power 300 W, showing additional peaks due to partial photodecomposition. Panel 3 (turquoise): Emission spectrum ($\lambda_{exc.}$ = 340 nm) of complex **3** at 77 K in MeTHF after irradiation at room temperature in solution for 10 minutes at λ = 340 nm, output power 300 W, indicating major decomposition. Bottom panel (blue): Emission spectrum ($\lambda_{exc.}$ = 340 nm) of **PyreneS**$_2$ at 77 K in MeTHF.

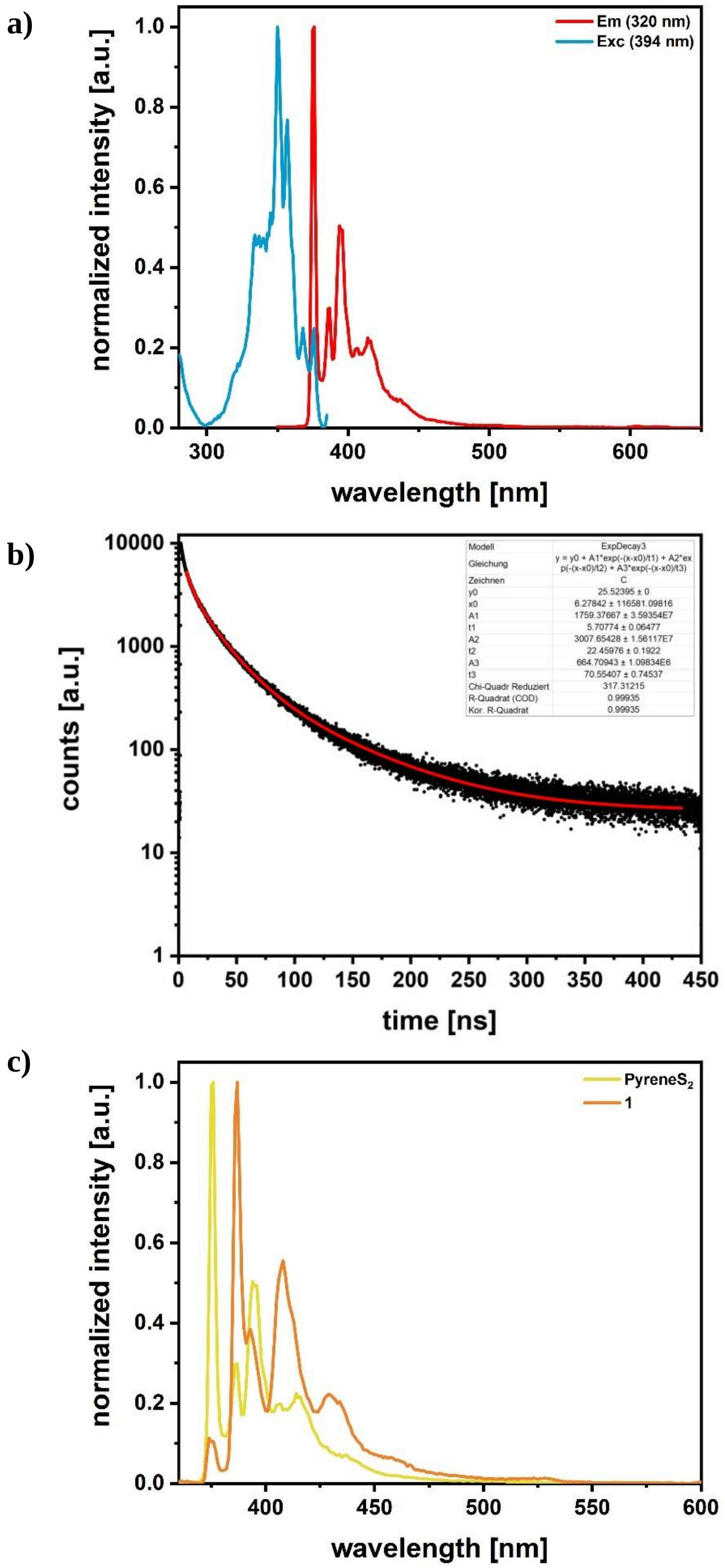

Figure S55. PL data of **PyreneS$_2$** at 77 K in MeTHF: a) Emission (red) and excitation spectrum (blue) of **PyreneS$_2$**; b) Lifetime of the 400 nm emission of **PyreneS$_2$**; c) Emission spectra of **PyreneS$_2$** (yellow) and of complex **1** (orange).

a)

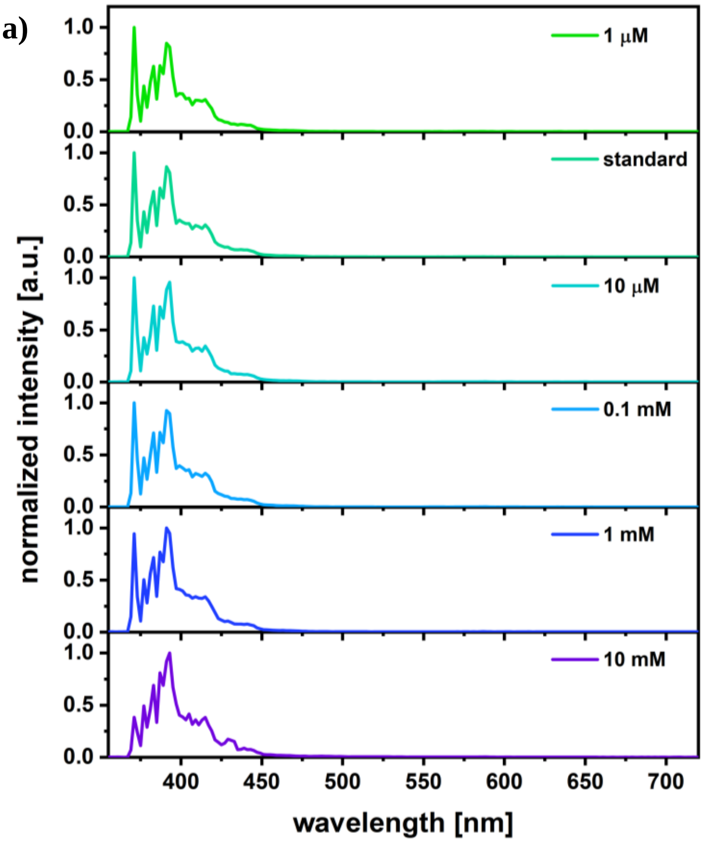

b)

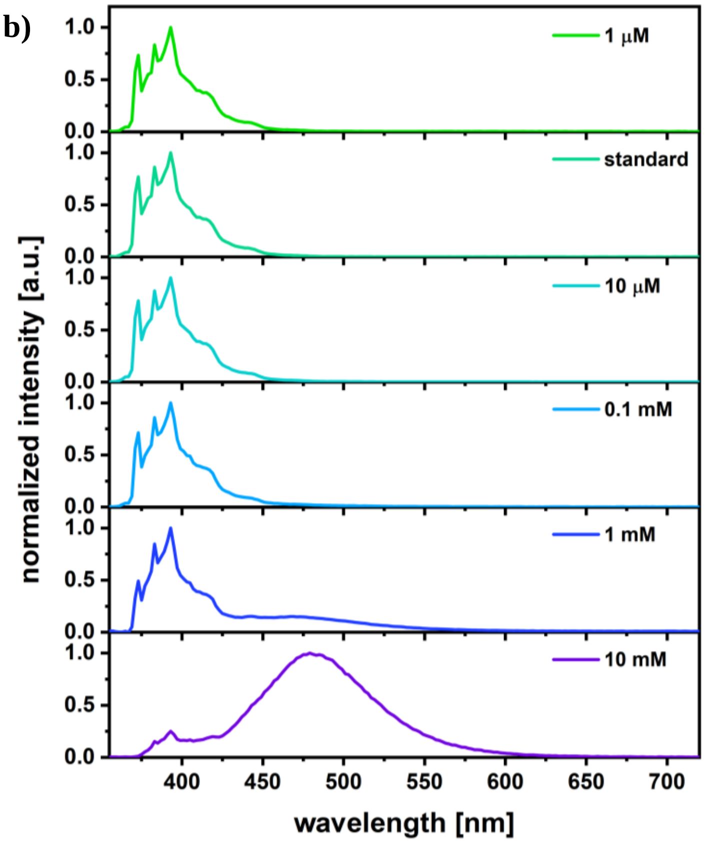

Figure S56. a) Concentration-dependent emission spectra of pyrene excited at 335 nm in MeTHF at 77 K; b) Concentration-dependent emission of pyrene excited at 330 nm in CH_2Cl_2 at room temperature.

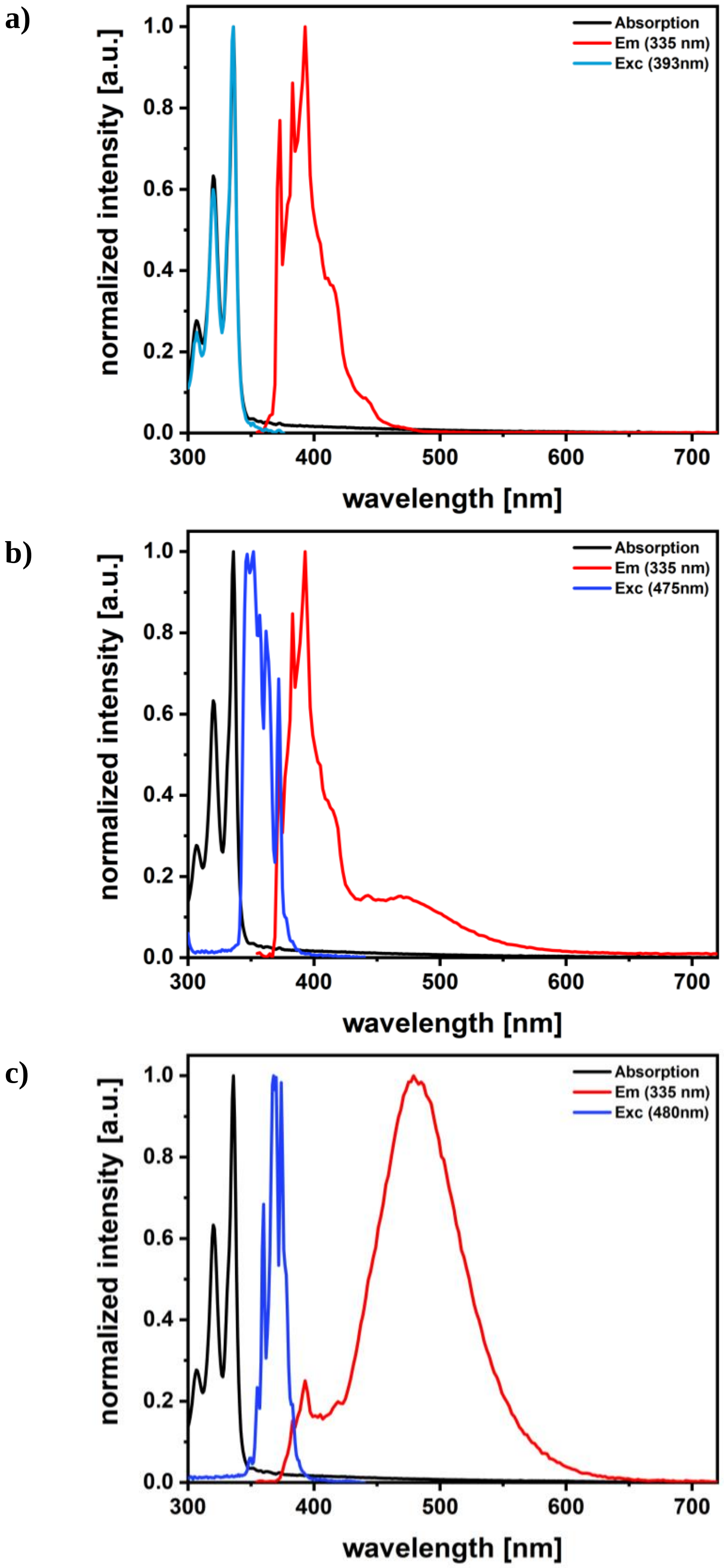

Figure S57. PL data of pyrene at room temperature in CH_2Cl_2 at different concentrations: Absorption (black), emission (red) and excitation spectra (blue) of pyrene at a concentration of a) 3-5 µM, b) 1 mM, and c) 10 mM.

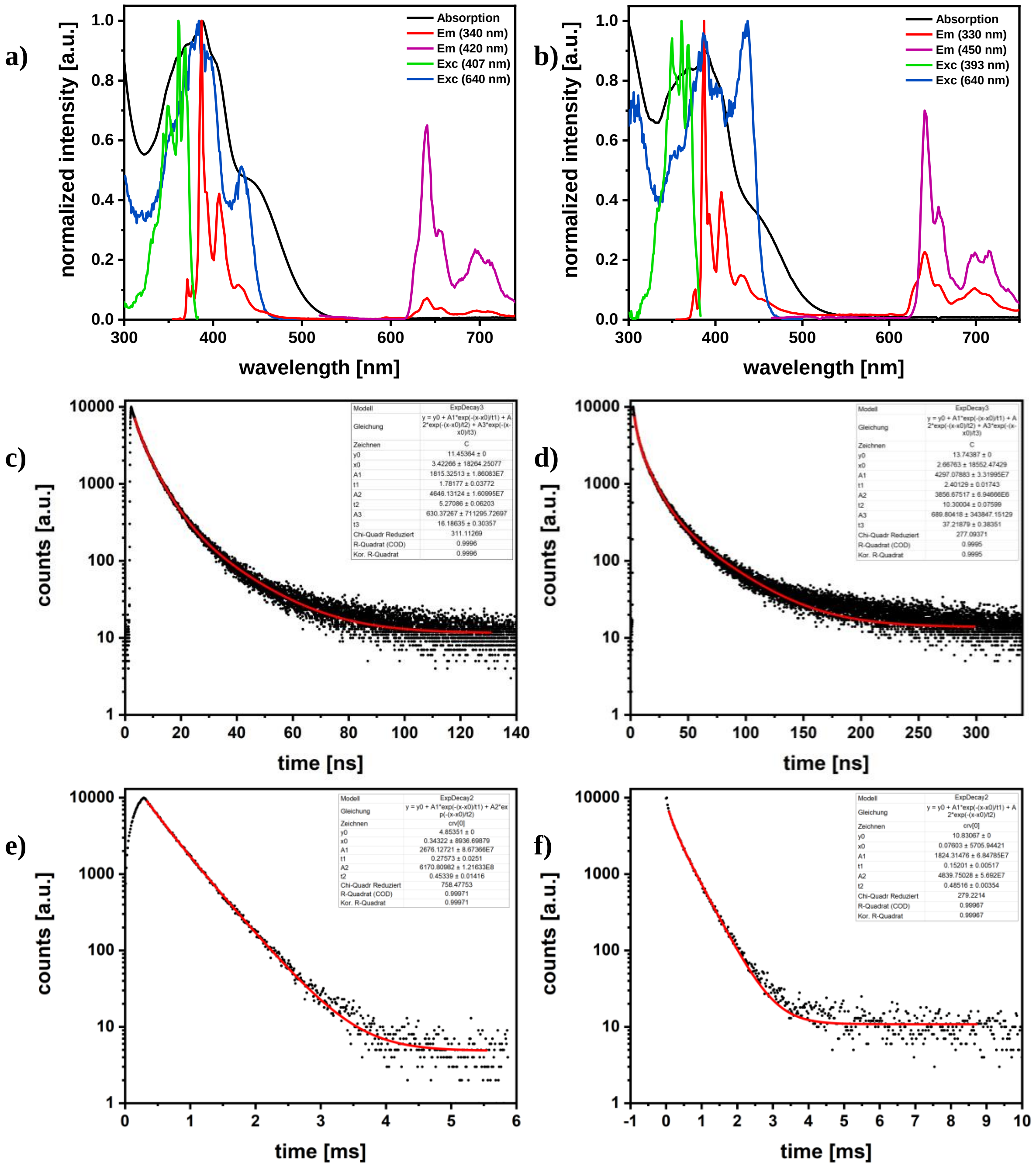

Figure S58. PL data of complexes **1** and **3** at 77 K in MeTHF: Emission (red) and excitation spectra (green and blue) of a) complex **1** and b) complex **3** under “standard” conditions (3 to 5 µM solutions). c) Lifetimes of the 395 nm emission of **1**; d) Lifetime of the 393 nm emission of **3**; e) Lifetime of the 640 nm emission of **1**; f) Lifetime of the 640 nm emission of **3**.

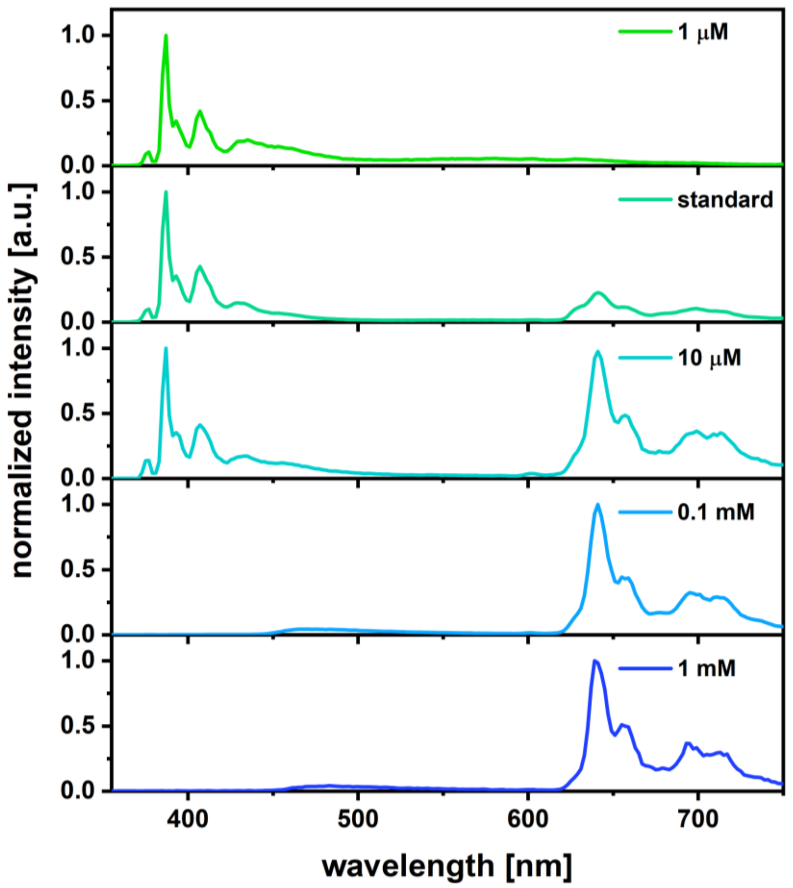

Figure S59. Concentration-dependent emission spectra of complex **3** excited at 340 nm in MeTHF at 77 K.

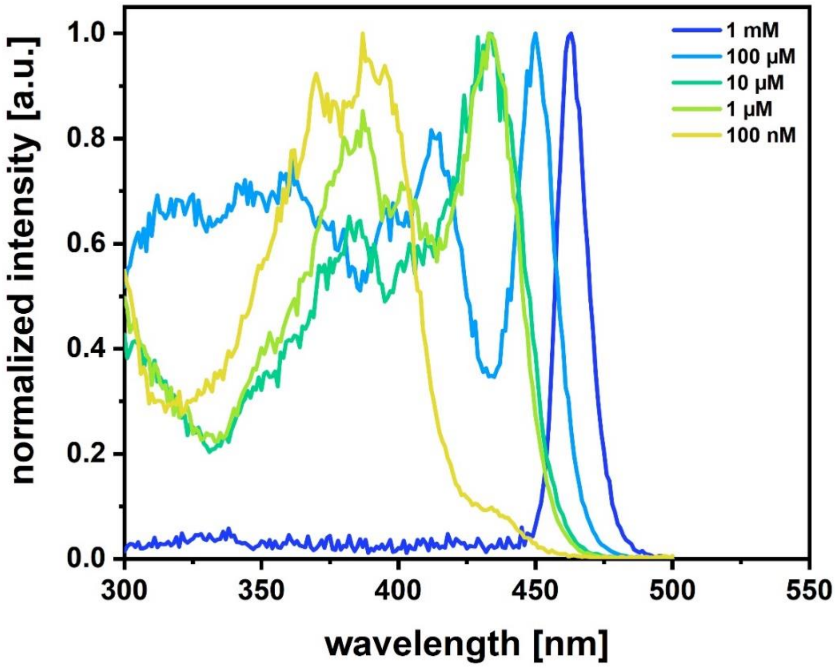

Figure S60. Concentration-dependent excitation spectra of complex **1** detected at 640 nm in MeTHF at 77 K.

Literature

(1) García Ruano, J. L.; Parra, A.; Alemán, J. Efficient synthesis of disulfides by air oxidation of thiols under sonication. *Green Chem.* **2008**, *10* (6), 706-711.
(2) Rodighiero, P.; Pastorini, G.; Chilin, A.; Marotto, A. Synthesis of methyl derivatives of linear and angular thienocoumarins and thiopyranocoumarins. *J. Heterocycl. Chem.* **1998**, *35* (4), 847-852.
(3) Irmler, P.; Gogesch, F. S.; Larsen, C. B.; Wenger, O. S.; Winter, R. F. Four different emissions from a Pt(Bodipy)$(PEt_3)_2$(S-Pyrene) dyad. *Dalton Trans.* **2019**, *48* (4), 1171-1174.
(4) Mangalum, A.; Gilliard Jr, R. J.; Hanley, J. M.; Parker, A. M.; Smith, R. C. Metal ion detection by luminescent 1,3-bis(dimethylaminomethyl) phenyl receptor-modified chromophores and cruciforms. *Org. Biomol. Chem.* **2010**, *8* (24), 5620-5627.
